# Discovery of YTHDF2 Ligands by Fragment-Based Design

**DOI:** 10.1021/acsbiomedchemau.5c00099

**Published:** 2025-06-27

**Authors:** Annalisa Invernizzi, Francesco Nai, Rajiv Kumar Bedi, Pablo Andrés Vargas-Rosales, Yaozong Li, Elena Bochenkova, Marcin Herok, František Zálešák, Amedeo Caflisch

**Affiliations:** Department of Biochemistry, 27217 University of Zurich, CH-8057 Zurich, Switzerland

**Keywords:** epitranscriptomics, m^6^A readers, docking, FRET binding assay, molecular dynamics, protein crystallography

## Abstract

*N* ^6^-Adenosine methylation is the most abundant modification of mRNA. The three members of the YTH domain family proteins (YTHDF1–3) recognize in the cytoplasm the m^6^A-RNA modification. We screened a library of about 500,000 fragments (i.e., molecules with 11–20 non-hydrogen atoms) by docking into YTHDF2, which resulted in the identification of six active compounds among 47 tested in vitro (hit rate of 13%). The acquisition of 28 analogues of the docking hits provided an additional set of 10 active compounds (IC_50_ < 100 μM). Protein crystallography-guided optimization of a ligand-efficient fragment by the synthesis of 32 derivatives culminated in a series of YTHDF2 ligands, which show low-micromolar affinity measured by a fluorescence polarization (FP) assay and a homogeneous time-resolved fluorescence-based (HTRF) assay. The series is characterized by very favorable ligand efficiency (of about 0.3–0.4 kcal/mol per non-hydrogen atom). Compound **23** binds to YTHDF2 according to the FP and HTRF assays with a *K*_d_ value of 1.3 μM and an IC_50_ value of 11 μM, respectively, and it is selective against all of the other YTH reader proteins. Several compounds of the series bind to the three YTHDF proteins with similar low-micromolar affinity, while they are less potent for YTHDC1 and YTHDC2. In contrast, compounds **17** and **30** bind also to YTHDC2, with *K*_d_ of 6.3 and 4.9 μM, respectively. We also disclose six crystal structures of YTHDF2 in the complex with the fragments identified by docking.

## Graphical abstract

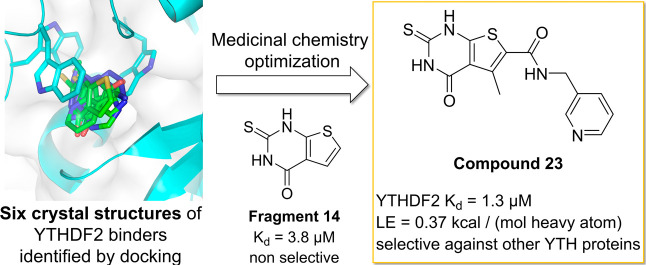

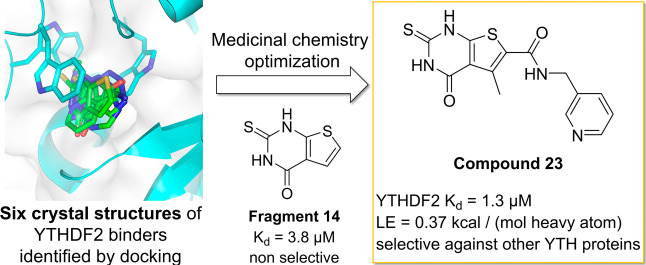

## Introduction

The YTH (YT521-B homology) domain-containing proteins are a family of RNA-binding proteins that specifically recognize *N* ^6^-methyladenosine (m^6^A), the most abundant internal modification in eukaryotic RNA.^1^ These proteins play critical roles in various biological processes, including mRNA metabolism, splicing, stability, and translation, influencing gene expression and cellular functions.^1–3^ The YTH family consists of five members: YTHDF1 (from now on termed DF1), YTHDF2 (DF2), YTHDF3 (DF3), YTHDC1 (DC1), and YTHDC2 (DC2).^4^ DC1 is primarily nuclear, where it participates in mRNA splicing, processing, and export.^5^ In contrast, the DF proteins (DF1, DF2, and DF3) are mainly cytoplasmic and play essential roles in mRNA translation, stability, and degradation.^3^ Finally, DC2 is the latest discovered member of this protein family and is mostly cytoplasmic, even though it has been found to interact with nuclear components, suggesting a dual role in RNA processing and regulation.^6,7^ Its functions appear to overlap with those of DF proteins, particularly in regulating RNA translation and stability. While other family members are broadly expressed across various cell types, DC2 is notably enriched in the testes, where it plays a critical role in germ cell development and maturation.^6^

While it has been established that each YTH protein possesses unique functions, there is evidence indicating that they can compensate for one another under certain conditions, leading to functional redundancy.^4,8,9^ Research has demonstrated that DF proteins can engage in context-dependent functional compensation. For instance, when one DF protein is knocked down, the others can partially compensate for the loss, maintaining the overall regulation of mRNA metabolism.^8,9^ This phenomenon highlights the complexity of the YTH protein family, where the precise roles of individual members may vary depending on the cellular context, the specific mRNA targets, and the presence of other regulatory factors.^4,8,9^

Given their crucial role in gene expression regulation, it is not surprising that YTH proteins and m^6^A regulation are heavily implicated in various diseases, especially cancer. Our study focuses on DF2, which is involved in multiple types of cancer, including prostate cancer,^10^ MYC-driven breast cancer,^11^ and acute myeloid leukemia (AML).^12^ This makes DF2 a highly attractive target for drug discovery and is gaining more and more attention. Even though we focus mainly on DF2, the highly conserved m^6^A binding site of the DF proteins hinders the development of a DF2-selective ligand.^13^ Furthermore, the discussed compensatory effects make a pan-DF ligand desirable.

Only a few small-molecule ligands have been identified for the DF protein family. Among them are Ebselen,^14^ Tegaserod,^15^ and Salvianolic Acid C,^16^ previously known compounds repurposed from other targets. Reviews of known inhibitors of the YTH proteins can be found in refs 17 and 18. In our earlier publication,^13^ we reported the first small-molecule binders of DF2; the X-ray crystal structure of one of them (compound **11**, IC_50_ = 174 μM)^13^ is the starting point of this work. Subsequently, Wang et al. reported the discovery of DC-Y13–27, a DF2 inhibitor with an IC_50_ of 21.8 μM (measured using an AlphaScreen assay) and a *K*_d_ of 38 μM (determined by microscale thermophoresis).^19^ The compound showed weaker activity on DF1 (IC_50_ = 165 μM in the AlphaScreen assay), but was not tested on DF3.^19^ A more recent study identified a series of functionalized pyrazoles as selective DF2 binders.^20^ The most potent compound, CK-75, exhibited an IC_50_ of 13.2 μM in an AlphaScreen assay and was found to be inactive against all other members of the YTH protein family. Notably, CK-75 induced cell cycle arrest and apoptosis in the K567 leukemia cell line, further supporting DF2 as a promising therapeutic target.^20^

Here we present a new series of DF2 binders identified by docking, followed by structure–activity relationship (SAR)-by-catalog. We discovered new chemotypes that compete with m^6^A-RNA for binding to DF2. Medicinal chemistry optimization of a ligand-efficient scaffold resulted in a series of low-micromolar binders of DF2. Most compounds of the series show a preference for DF proteins against DC1 and DC2. Compound **23** binds only to DF2, being selective against all of the other YTH proteins.

## Results and Discussion

### Docking of a Library of Fragments

The chemical diversity represented by a library of *N* fragments (with up to 18–20 non-hydrogen atoms) is substantially larger than the diversity of a library of *N* molecules with more than 25–30 heavy atoms.^21^ As a consequence, fragment screening by docking is an efficient alternative to the screening of ultralarge libraries of compounds.^22^ Thus, we decided to start with a fragment docking campaign in DF2. A library of 500,000 fragments was docked using the program SEED.^23,24^ The fragments were selected from the ZINC20 database^25^ according to the following rules: between 11 and 20 heavy atoms, at least one ring, and at least one sp^3^-hybridized carbon. The first two rules reflect the properties of the program SEED, which was developed for docking mainly rigid fragments. The third rule was selected as our first campaign had identified ligand fragments with methyl, ethyl, or cyclopropyl in the bottom of the aromatic pocket.^13^ For each of the extracted compounds, up to 20 conformers were generated using a distance geometry-based algorithm,^26^ and docked by SEED. Two structures of the m^6^A-RNA recognition domain of DF2 were used for docking (Figure 1). The crystal structure in the complex with 6-cyclopropylpyrimidine-2,4-diol (compound **11** in ref 13 PDB ID: 7R5W) and a snapshot obtained by molecular dynamics (MD) simulations started from the same crystal structure. The MD snapshot was selected by analysis of the time series of the volume of the recognition pocket. It has a more open recognition loop^27^ with a pocket volume of 600 Å^3^, which is significantly larger than the volume of 324 Å^3^ in the crystal structure 7R5W (Figure 1).

**Figure 1:**
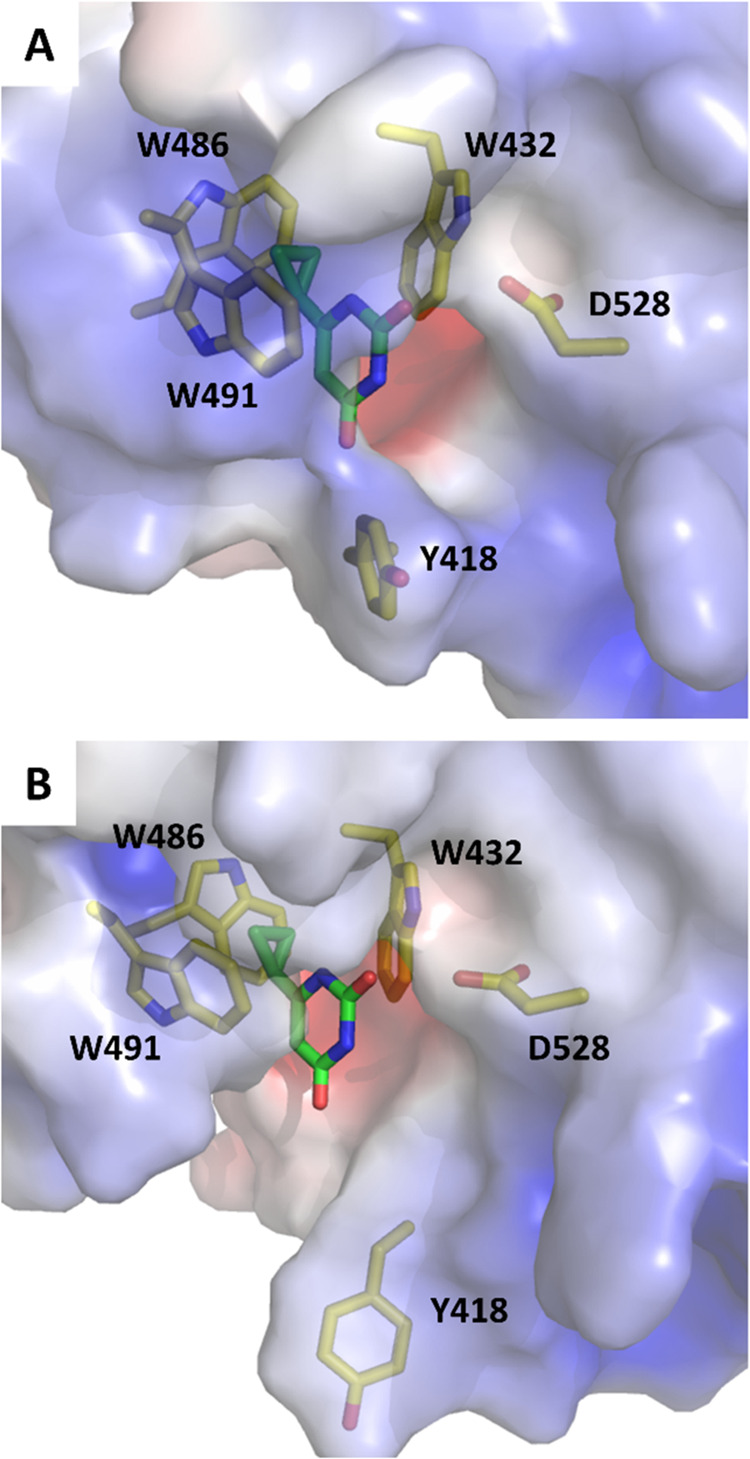
Two structures of the DF2 reader employed for docking. (A) X-ray crystal structure of DF2 in the complex with 6-cyclopropylpyrimidine-2,4-diol (compound **11** of ref 13 PDB: 7R5W). (B) MD-simulation snapshot with a larger aperture of the m^6^A-recognition pocket (see Materials and Methods). The surface of DF2 is colored by the electrostatic potential (red, negative; blue, positive), and 6-cyclopropylpyrimidine-2,4-diol (carbon atoms in green) and the binding site residues are shown by sticks (carbon atoms in yellow).

The two protein structures were kept rigid during docking and evaluation of the binding energy. SEED calculates the binding energy by a force field with implicit treatment of the electrostatic effects of the solvent. The docked compounds were ranked according to two energy terms, namely, the total binding energy and the difference between the electrostatic contribution to the intermolecular interaction energy in the solvent and the solvation energy of the ligand. The top-scoring compounds were then selected if they showed the crucial hydrogen bond with the backbone carbonyl of C433, which is the acceptor of the *N* ^6^ of the natural ligand m^6^A, and another polar interaction within the binding site (see Materials and Methods). If an interesting compound was not commercially available, then a structurally similar analogue was chosen. Finally, 25 and 22 compounds were selected from the docking campaigns that made use of the crystal structure and MD snapshot, respectively (Table S1).

### In Vitro Validation

A previously reported homogeneous time-resolved fluorescence (HTRF)-based assay was used to measure the binding affinity of the 47 ordered compounds (see Materials and Methods).^28^ For the nine compounds with residual signal at 1 mM smaller than 60% (with respect to dimethyl sulfoxide (DMSO) control), the IC_50_ value was determined by dose–response experiments (Table S1). Among these, the thiobarbiturate derivatives **1** and **2** were the strongest binders of DF2, with IC_50_ values of 19 and 170 μM, respectively (Table 1). The thiobarbiturate derivative **1** shows a very favorable ligand efficiency (LE) of 0.50 kcal/(mol HAC) [HAC = heavy atoms count, i.e., number of non-hydrogen atoms] while the toluene group of compound **2** does not seem to contribute to binding.

**Table 1:**
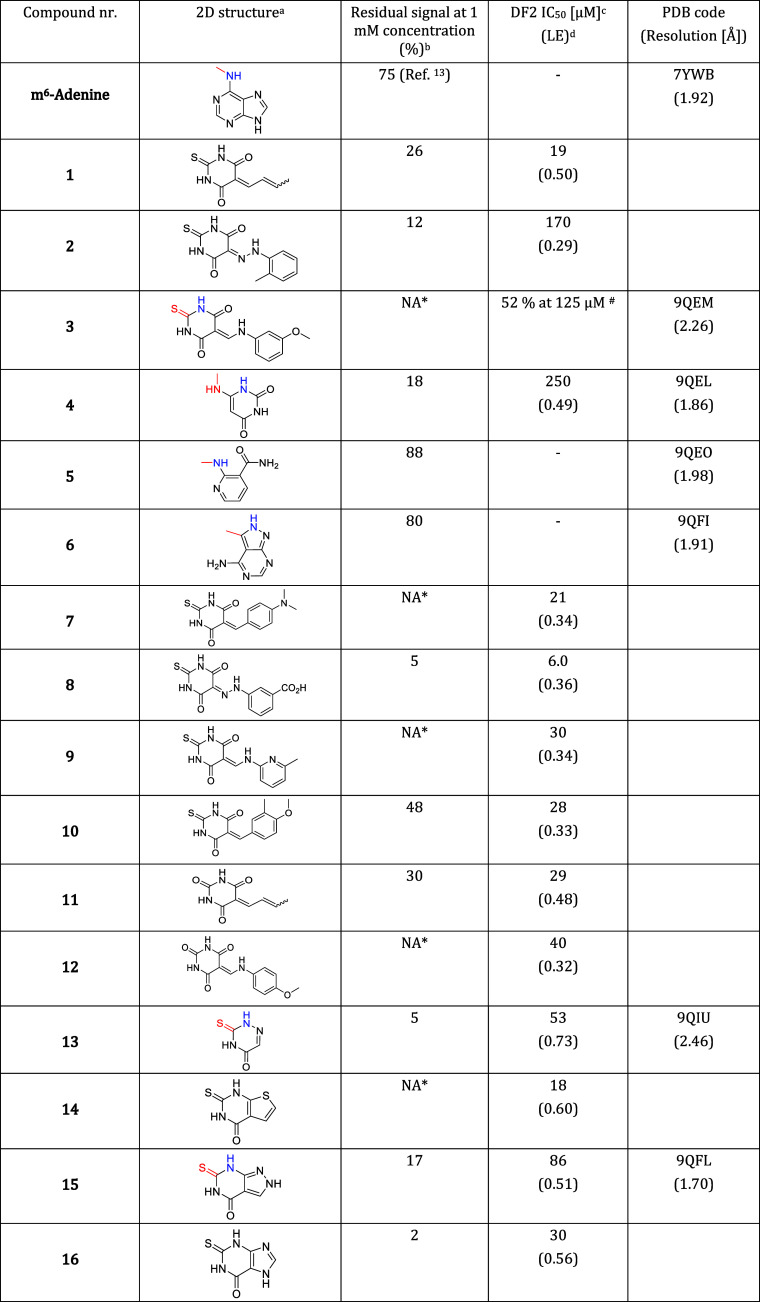
m^6^-Adenine and 16 Ligands of DF2 Identified by Docking, Followed by SAR by Catalogue^e^

a The NH group interacting as a hydrogen-bond donor with the backbone carbonyl of C433 (blue) and the group in the bottom of the tryptophan cage (red) are emphasized for the compounds with a crystal structure in the complex with DF2.

b The residual signal at 1 mM compound concentration is measured using an HTRF-based binding assay as previously reported.^28^ The signal decreases (with respect to buffer-only measurement) when the fragment competes with the binding of the natural ligand, i.e., m^6^A-oligoRNA. Thus, the lower the signal, the higher the affinity of the fragment. The reported values are the average of two technical replicates.

c We use the term IC_50_ for the concentration of the compound that reduces the signal by 50% with respect to buffer-only. YTH readers are not enzymes, but we still prefer the more frequently used inhibitory concentration (IC_50_) rather than effective concentration. The IC_50_ value for the DF2 reader domain was measured only for the fragments that, at a concentration of 1 mM, decrease the signal by more than 60%.

d The ligand efficiency is calculated as $\mathrm{LE}=-\frac{\Delta G}{HAC}\approx -\mathrm{RT}\frac{\ln{IC}_{50}}{HAC}$ and the values are reported in kcal/(mol HAC), HAC = heavy atom count.

e Compounds **1**–**6** were identified by docking, while compounds **7**–**16** were selected by SAR by catalogue. * Interference or poor solubility observed at 1 mM. ^#^ Interference or poor solubility observed at higher concentrations, IC_50_ could not be determined.

The X-ray crystal structure was solved for compounds **3**–**6** at high resolution (Table 1 and Figure 2B–E). The unique binding mode of compound **3** provided evidence that the ene-thiobarbiturate substructure of compounds **1**–**3** specifically and noncovalently binds to DF2 despite the potential Michael acceptor reactivity.

**Figure 2:**
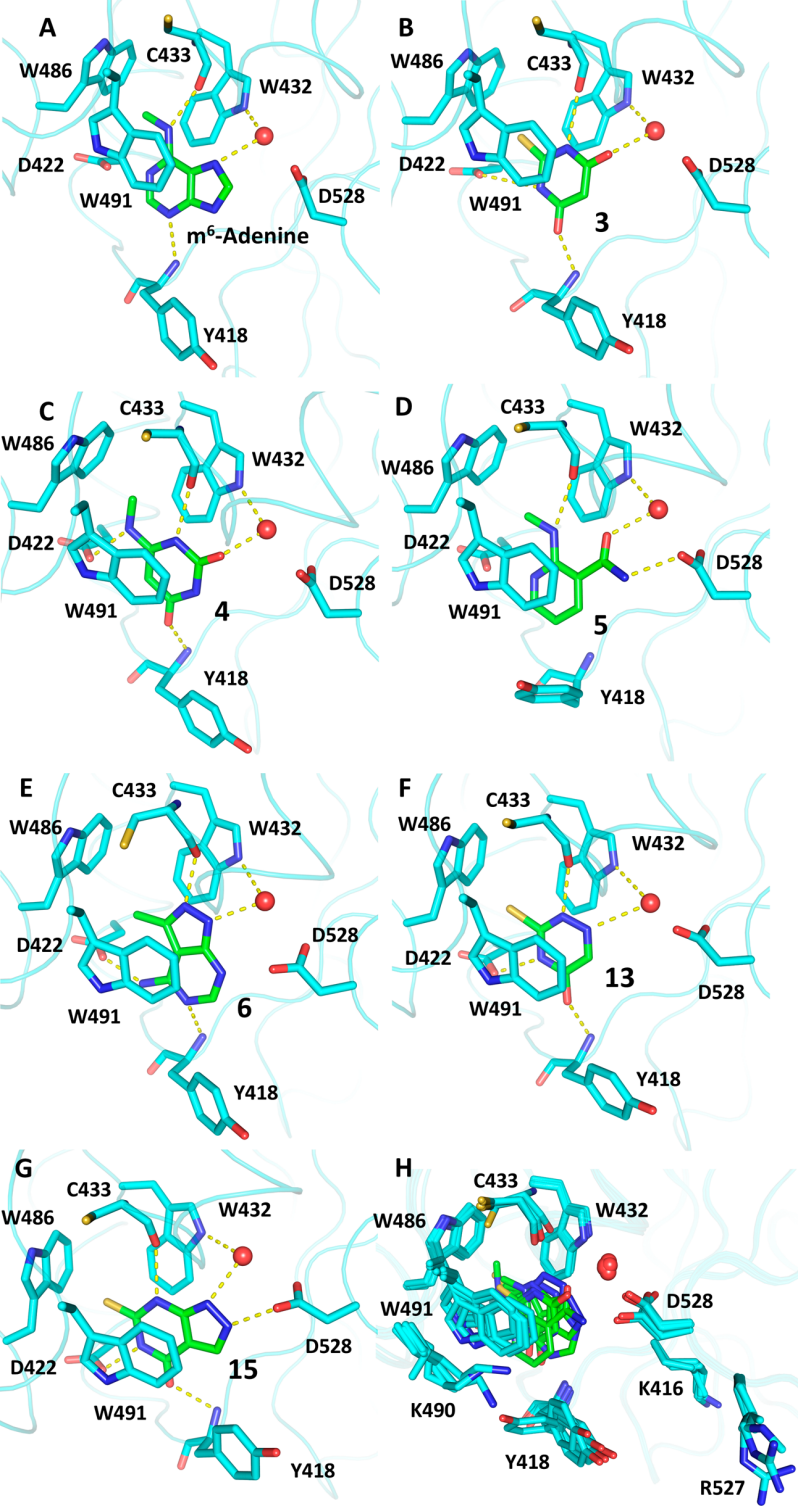
Crystal structures of DF2/fragment complexes. (A–G) Binding modes of m^6^-adenine (compound **1** in ref 13, PDB: 7YWB), compounds **3** (PDB: 9QEM), **4** (PDB: 9QEL), **5** (PDB: 9QEO), **6** (PDB: 9QFI), **13** (PDB: 9QIU), and **15** (PDB: 9QFL), respectively. The conserved water molecule (red sphere) and the hydrogen bonds (yellow dashed lines) are also shown. (H) Structural overlap of panels (A)–(G). The carbon atoms of the ligands are colored green and those of the protein in cyan.

The six binders **1**–**6** (Table 1) belong to four distinct chemotypes: thiobarbiturates (**1**–**3**), uracil (**4**), nicotinamide (**5**), and pyrazolopyrimidine (**6**). Despite their chemical diversity, these chemotypes feature an NH group that is involved as a hydrogen-bond donor with the backbone carbonyl of C433 (Figure 2B–E). Furthermore, they act as hydrogen-bond acceptor of the conserved water that forms hydrogen bonds with the side chains of W432 and D528. Notably, compounds **1**–**3** occupy the bottom of the aromatic cage with their sulfur atom, while compounds **4**–**6** have a methyl group as in the natural ligand. Compounds **1**–**4** and **6** act as hydrogen-bond acceptors for the backbone NH of Y418, and they also form a hydrogen bond with the side chain of D422. This interaction may offer selectivity against the nuclear reader DC1, which features the N367 hydrogen-bond donor NH_2_ in this position.^29^ In our first screening campaign,^13^ we described a series of uracil analogues; compound **4** is a new member of this series with a modest IC_50_ value of 250 μM. The amide nitrogen atom of compound **5** is involved in a hydrogen bond with the side chain of D528, but it does not form favorable interactions with D422 and the backbone NH of Y418. The 70° relative orientation and 4.8 Å distance between the ring centers indicate that the aromatic side chain of Y418 engages in an edge-to-face π-stacking interaction with **5**.^30,31^

### SAR by Catalogue

From the information gained in the first screening, a second set of 28 compounds was ordered (Table S2). This set consisted of 14 top-ranking docked molecules with a chemotype similar to that of compounds **1–3**, and other 14 molecules that are closely related to the discovered binders **1**–**6** but were not present in the library used for docking. A total of 16 of the ordered compounds belong to the thiobarbiturate chemotype, which was considered very promising from the previous results. Ten of the 28 compounds (ligands **7–16**) showed an IC_50_ < 100 μM.

Among the thiobarbiturate derivatives **7–10**, ligand **8** is the most potent (IC_50_ = 6 μM and LE = 0.36). The carboxylic acid substituent is likely to contribute to the binding affinity, as the solvent-exposed portion of the DF2 binding pocket is rich in positively charged residues (K416, K490, and R527; Figure 2H), which typically interact with the negatively charged phosphates of RNA, its natural substrate.

Other molecules closely related to the thiobarbiturates were identified as interesting binders: barbiturates **11** and **12**, triazine **13**, and condensed bicycles **14**–**16**. The X-ray crystal structures of compounds **13** and **15** confirm their binding (Figure 2F,G), again showing the sulfur atom interacting within the lipophilic tryptophan cage.

Compound **15** can establish all of the interactions previously discussed: in addition to the interactions of m^6^-adenine, it can interact with both D422 and D528. (Note though that m^6^-adenine is likely to be protonated in the bound state as suggested by the distance of 2.6 Å between its N1 atom and the nearest O atom of the D422 side chain (PDB entry 7YWB).) Unfortunately, the multiple interactions of compound **15** do not translate into a very strong binding (IC_50_ = 86 μM). This might be a consequence of the major tautomer represented in Table 1 being in equilibrium with other tautomeric forms, which are not ideal for efficient binding (∼67% of the desired tautomer, as calculated with the Chemaxon tautomers generator plugin https://plugins.calculators.cxn.io/tautomers/). Compounds **13** and **14** are characterized by very favorable LE values of 0.73 and 0.60, respectively. We decided to further explore the derivatization of compound **14** because of its favorable LE, the two possible vectors for substitution at the two carbon atoms of its thiophene ring, respectively, and the relatively accessible synthesis (see below). We could not determine the crystal structure of DF2 in complex with **14**. The choice of compound **14** was supported by its pose predicted by docking, which overlapped with the binding mode of compound **15** in the high-resolution crystal structure with DF2 (PDB entry 9QFL).

### Derivatization of Compound **14**

The exploration of analogues of **14** has been pursued by synthesizing new derivatives (Tables 2 and S4) and ordering commercially available variants (Table S3). The replacement of the exocyclic sulfur atom with oxygen was detrimental to the potency, from 18 to 290 μM (compound **S61**). Several 5- and 6-membered ring alternatives to the thiophene were explored (compounds **15**, **16**, **S5**, **S62–S64**), and only compound **16** resulted in an IC_50_ comparable to that of fragment **14** (30 vs 18 μM).

**Table 2:**
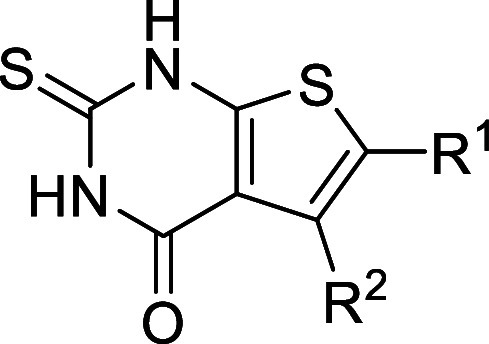

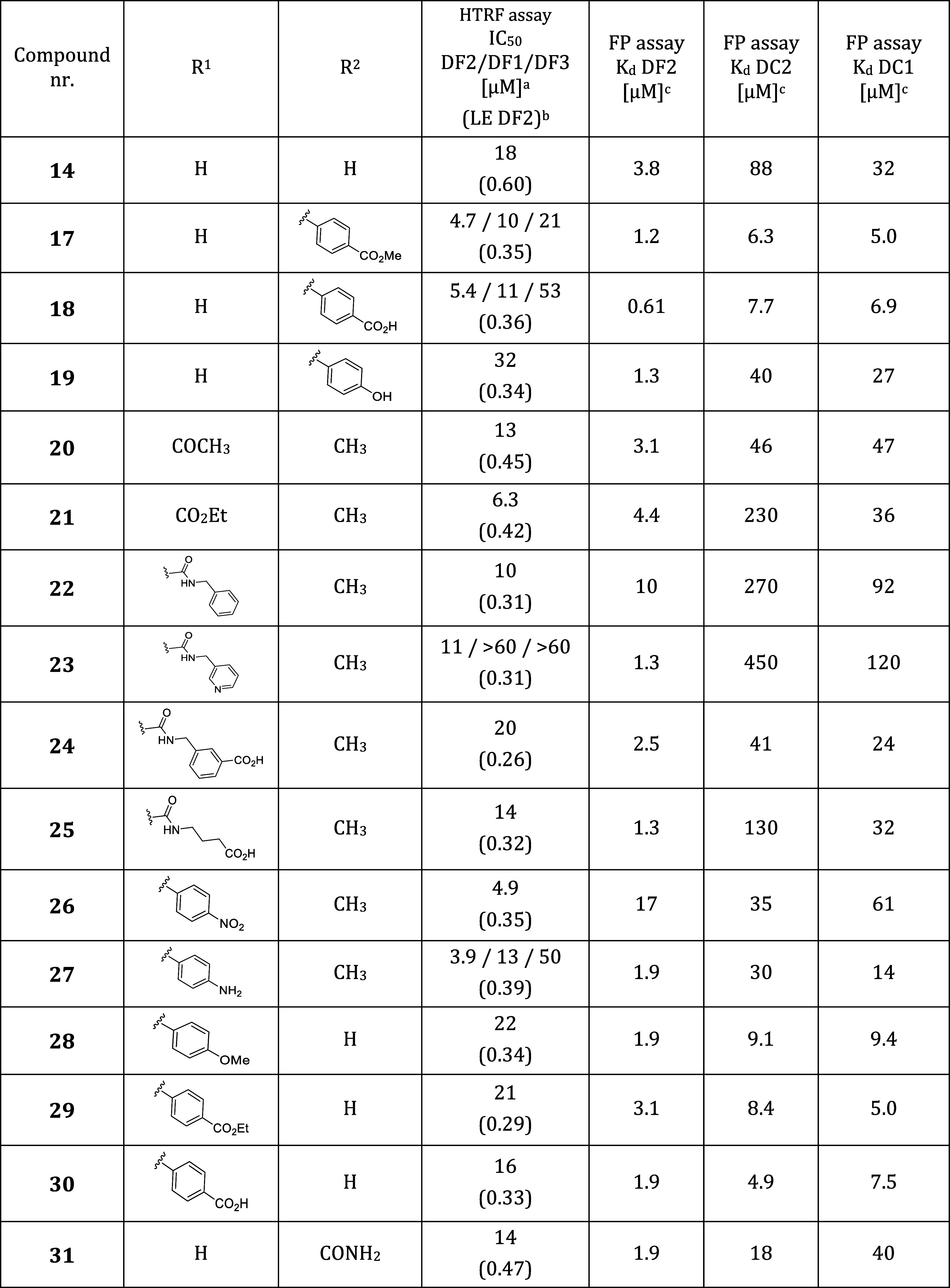
Expansion of Hit Fragment **14** ^d^

a For compounds with a single value, it refers to DF2. The dose–response curves and replicates are shown in the Figure S1.

b The LE formula is shown in the caption of Table 1.

c The *K*_d_ values were determined with the Cheng–Prusoff equation using a *K*_d_ value of 5 nM (DF2), 7 nM (DC2), and 5 nM (DC1) for the 5′-fluorescein-labeled m^6^A-DNA probe. The reported values are the averages of one (DC1) or two (DF2 and DC2) biological replicates, and each replicate is the average of four technical replicates. The dose–response curves are in Figures S2 (DF2), S3 (DC2), and S4 (DC1).

d Compounds **17**–**31** are more active for DF2 than compound **14** in the HTRF and/or the FP assays.

The medicinal chemistry campaign focused on derivatizing the thiophene ring of **14** through the addition of R^1^ and/or R^2^ groups (Table 2 and Table S4). A total of 32 molecules were synthesized (Table S4). Table 2 shows the molecules with higher affinity than compound **14** as measured by the HTRF-based binding assay and/or FP. As mentioned above, we were not able to determine the crystal structure of **14** in complex with DF2. Thus we hypothesized a similar binding mode as in the crystal structure with compound **15** (Figure 3A). The putative binding poses will be further analyzed and discussed in the next section.

**Figure 3:**
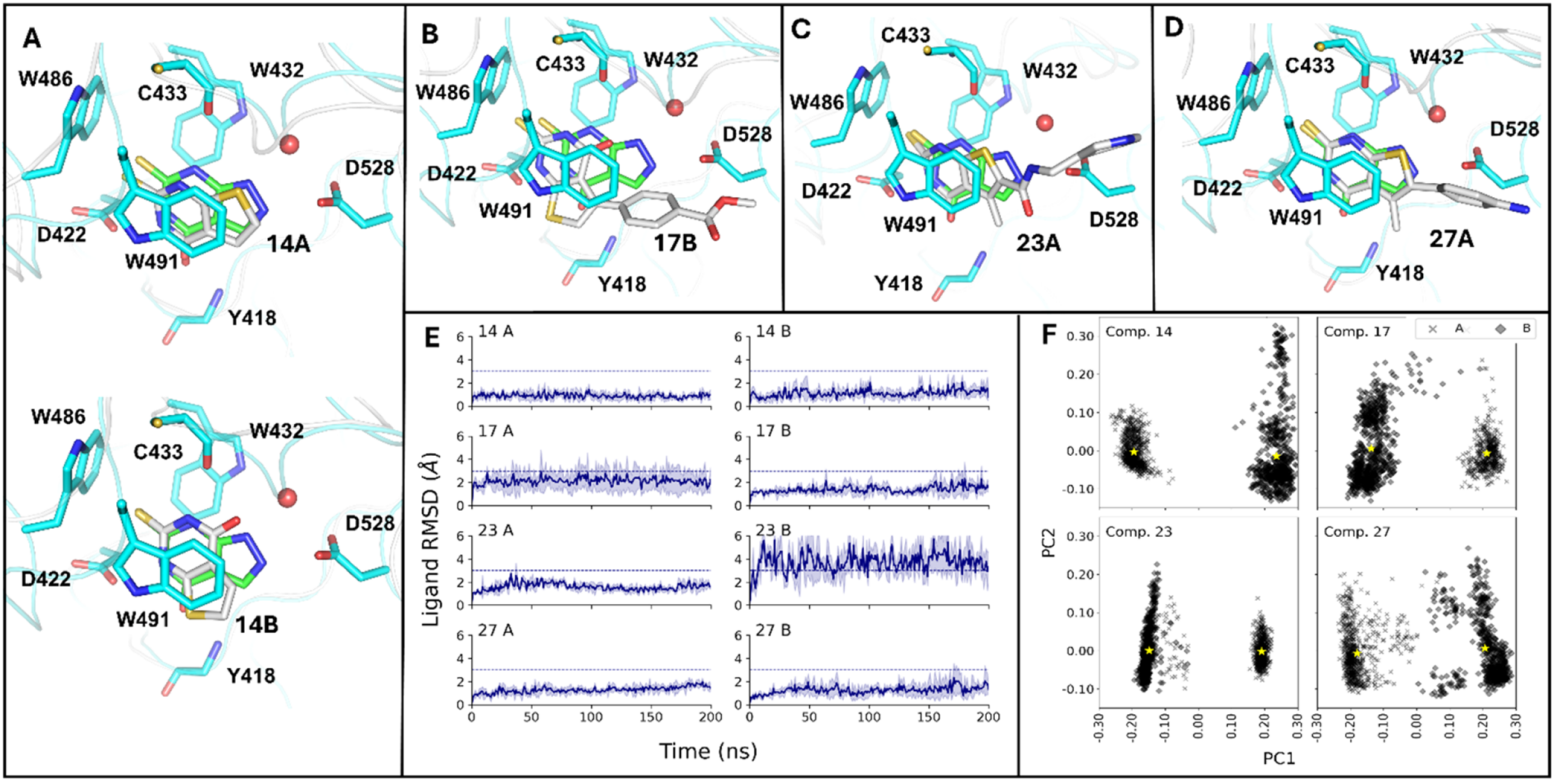
Predicted binding modes of compounds **14**, **17**, **23**, and **27** in DF2. (A) The pseudosymmetry of fragment 14 (carbon atoms in gray) results in two binding modes called here A (top) and B (bottom). The crystal structure of DF2 (cyan, PDB: 9QFL) in complex with fragment 15 (carbon atoms in green) is overlapped for comparison. (B–D) The most populated pose of compounds 17 (pose B), 23 (pose A), and 27 (pose A). Their alternative poses are shown in Figure S6. (E) Analysis of the MD simulations started from the two potential poses (pose A, left; pose B, right). The time series shows the median ligand root-mean-square deviation (RMSD) with respect to the first frame, with a colored band corresponding to one median absolute deviation around the median. (F) Principal component analysis (PCA) projections of the distances between atoms of the ligand and representative residues of the DF2 binding site. Data for MD snapshots saved every 1 ns are shown for the two poses (A, cross; B, diamond), and the centers of the two clusters are emphasized (yellow star).

Compound **S74** (R^2^ = phenyl) and its derivatives (**17**–**19**, **S75**, and **S76**) were synthesized to try to obtain a favorable π–π stacking interaction with Y418, as seen in the X-ray crystal structure of the *N*-methyl-3-phenyl-1*H*-pyrazolo­[4,3-*d*]­pyrimidin-7-amine (compound **7** in ref 13, PDB: 7YXE). Among these compounds, **17** and **18** resulted in a 3-fold improved IC_50_ in the HTRF-based binding assay (Table 2), possibly due to a hydrogen bond between the carbonyl group and the hydroxyl of Y418 or the −NH_2_ of N462. At R^1^, we started with small polar substituents to try to establish interactions with D528 and/or with the structural water molecule or to try to replace the water molecule (compounds **20**, **21**, **S79**, and **S80**). The ethyl ester of **21** at R^1^ enhanced the binding (IC_50_ = 6.3 μM), as also the bulkier benzyl (**22**, IC_50_ = 10 μM) and methylpyridine (**23**, IC_50_ = 11 μM) groups, both connected to the thiophene via an amide bond. We hypothesized that the −CH_2_– linker enables the aromatic ring to orient toward the solvent-exposed region of the pocket, which is enriched with positively charged residues that facilitate binding to the negatively charged RNA. Based on this, we explored modifications such as adding a carboxylic acid (**24**) and further increasing the flexibility by replacing the benzyl group with an alkyl chain (**25** and **S83**). However, none of these changes led to an improvement in the potency.

We continued the exploration of R^1^ with substituted benzyl and phenyl rings directly connected to the thiophene (compounds **26**–**30** and **S84**–**S87**). Compounds **26** and **27** resulted in an improvement of 3- to 4-fold compared to fragment **14**.

An FP competition assay was used to further validate the binding of the compounds to DF2. The main difference with respect to a previously published FP assay^32^ is the use of an oligo-DNA as competitor ligand (see Materials and Methods section). For most compounds, there is a factor of 2–5 difference between the IC_50_ values for DF2 measured by HTRF and FP (Table 2). The largest discrepancy is a factor of 16 for compound **19** (32 and 2 μM by HTRF and FP, respectively; note that Table 2 shows the *K*_d_ value for FP, which is equal to the IC_50_/1.6 for DF2). These differences might originate from the varying conditions in the two assays, such as the competitor mRNA (5′-biotin-AAGAACCGG­(m6A)­CUAAGCU-3′) in HTRF and DNA (5′-FAM-AAGAACCGG­(m6A)­CTAAGCT-3′) in FP, the salt concentration (150 mM NaCl and 100 mM KF in HTRF, and 150 mM NaCl in FP), and the pH (7.5 in HTRF and 7.4 in FP). The FP-based assay was also employed to assess the selectivity against DC1 and DC2 (see Selectivity section).

### Computational Analysis

We could not determine the crystal structure of the complex of DF2 with compound **14** or any of its derivatives by soaking the ligands into apo DF2 crystals or cocrystallization. Thus, we decided to run MD simulations to investigate the binding mode of **14** and its derivatives **17**, **23**, and **27** (Figure 3). As already observed during the docking campaign, the symmetry of the thiourea substructure is congruent with two distinct poses (A and B), which are flipped by a rotation of 180° around the S=C double bond. For each compound and pose, eight independent 0.2-μs MD simulations were performed for a cumulative sampling of 3.2 μs per compound, starting from the docked poses of compound **14**, or the alignment of the derivatives **17**, **23**, and **27** to it.

The analysis of the MD trajectories was carried out by adapting the protocol described in ref 33 (see Materials and Methods). Only the second half of each MD run, i.e., the trajectory segments from 100 to 200 ns, was used for the analysis to allow for sufficient ligand relaxation. From these segments, we extracted the simulation frames where the compound is bound, defined as having a distance lower than 5 Å between the exocyclic sulfur atom and the N atom of C433. Then we calculated a set of 10 distances between the heavy atoms of the compounds (all located in the scaffold, i.e., present in compound **14**) and the binding pocket. Finally, principal component analysis (PCA) was used to project the multidimensional space into two dimensions. The two-dimensional data were then clustered using the Gaussian Mixture algorithm. The trajectory frame closest to the center of each cluster (centroid) was extracted and used as the reference pose (Figure 3F).

Figure 3A shows the two reference poses obtained for compound **14** compared to the crystal structure of compound **15**. In both poses, the exocyclic sulfur atom is positioned within the lipophilic tryptophan cage. As mentioned above, compound **14** can adopt two distinct orientations, which are related by a 180-degree rotation. This flipping rearranges the hydrogen-bond interactions while preserving the same number of favorable polar contacts. In both poses, the two NH groups of the thiourea act as hydrogen-bond donors for the backbone carbonyl of C433 and the side chain of D422, respectively. Moreover, the carbonyl group and the thiophene sulfur atom are inverted between the two poses. In pose A, the carbonyl oxygen interacts with the backbone NH of Y418, as observed for the crystal structure with compound **15** (Figures 2G and 3A top). In pose B, it instead forms a hydrogen bond with the conserved water molecule (Figure 3A, bottom). The sulfur atom in the thiophene ring points toward the structural water in pose A and the backbone NH of Y418 in pose B. In both poses, it acts as a weak hydrogen-bond acceptor.^34–36^ The root-mean-square deviation (RMSD) analysis of the MD runs started from the two poses suggests that pose A is slightly more stable (Figure 3E, top).

A similar analysis was performed for compounds **17**, **23**, and **27** to investigate the impact of bulky R^1^ and/or R^2^ substituents on the stability of the two poses. For compound **17**, a smaller RMSD is observed for the B pose in comparison to A, and the opposite was observed for compound **23** (Figure 3E). A smaller difference emerges for compound **27**, with pose A being slightly more stable.

We also calculated the population of each cluster (Figure 3F). As expected, the most populated cluster corresponds to the most stable pose based on RMSD analysis. Specifically, 55% of the bound frames of compound **14** belong to the cluster of pose A (Figure 3A, top); 61% of the bound frames of compound **17** to pose B (Figure 3B); 56% of compound **23** to pose A (Figure 3C); and 53% of compound **27** to pose A (Figure 3D).

### Selectivity

The HTRF-based assay was employed to evaluate the selectivity of the series against other DF proteins. The compounds were tested at a concentration of 100 μM and most of them also inhibit DF1 and DF3 (Table S5).

A dose–response experiment on DF1 and DF3 was conducted for compounds **17**, **18**, **23**, and **27** (Figure S5). Compounds **17**, **18**, and **27** are active on three DF proteins. Their IC_50_ values for DF1 are approximately twice as high as those for DF2, while for DF3, they are 4–12 times higher. In contrast, compound **23** is selective for DF2, with residual signals (at the highest employed concentration of 62.5 μM) of 51 and 87% for DF1 and DF3, respectively.

The binding affinity for DC1 could not be evaluated by HTRF because of interference of the compounds with the assay, which has a smaller assay window when performed with DC1 compared to the DF proteins. Thus, the FP assay was used to evaluate binding to DC1 and DC2. The affinity of the compounds for DC2 varied significantly depending on the substituents, with *K*_d_ values ranging from 5 μM for compound **30** to over 400 μM for compounds **23**, **S74**, **S78**, **S79**, **S82**, **S83**, and **S89** (Tables 2 and S4).

The parent scaffold, compound **14**, exhibits approximately a 20-fold and 8-fold higher binding affinity for DF2 compared to DC2 and DC1, with *K*_d_ values of 3.8, 88, and 32 μM, respectively, as measured by FP (Table 2). Many derivatives maintain a strong preference for DF2. Notably, compound **23** shows high selectivity, being about 350 and 100 times more potent for DF2 than DC2 and DC1, respectively. MD simulations of compound **23** in complex with DC1 were performed following the same strategy described for DF2 (see the Computational Analysis section). The RMSD analysis (Figure S7) indicates a higher stability (lower RMSD) of both poses A and B in DF2 compared to DC1. These results are consistent with the selectivity of compound **23** for DF2 and against DC1.

In contrast, compounds with a substituted phenyl ring directly connected to the thiophene (R^1^ or R^2^) exhibited little to no selectivity against the DC proteins (e.g., ratio *K*_d_ DC1/DF2 and DC2/DF2 of only 2–4 for ligands **29** and **30**).

Compounds **17**, **18**, and **28**–**30** are the most potent DC binders of the series. They feature benzoic acid (at R^2^ in compound **18** and R^1^ in compound **30**), benzoic ester (at R^2^ in compound **17** and R^1^ in compound **29**), or para-methoxyphenyl (at R^1^ in compound **28**). Their similar behavior indicates that the previously discussed two poses (A and B, see previous section) may also be populated in the DC1 and DC2 binding sites. These results suggest that the substituent, at either R^1^ or R^2^, likely occupies the same region of the binding pocket. To the best of our knowledge, the only previously identified DC2-binder in the literature is the pan-YTH binder “N-7”, with a reported IC_50_ of 30 μM (as measured by FP).^32^ Thus, compounds **17** and **30** are the most potent DC2 ligands as of today (*K*_d_ values of 6.3 and 4.9 μM, respectively, measured by FP).

### Chemistry

The general synthetic approach begins with the synthesis of the ethyl 2-aminothiophene-3-carboxylate derivatives (**37a**–**g**), with the corresponding R^1^ and/or R^2^ substituents. For compounds **37a**–**e** this was achieved via a classic one-pot Gewald reaction (Scheme 1).^37,38^

**Scheme 1:**
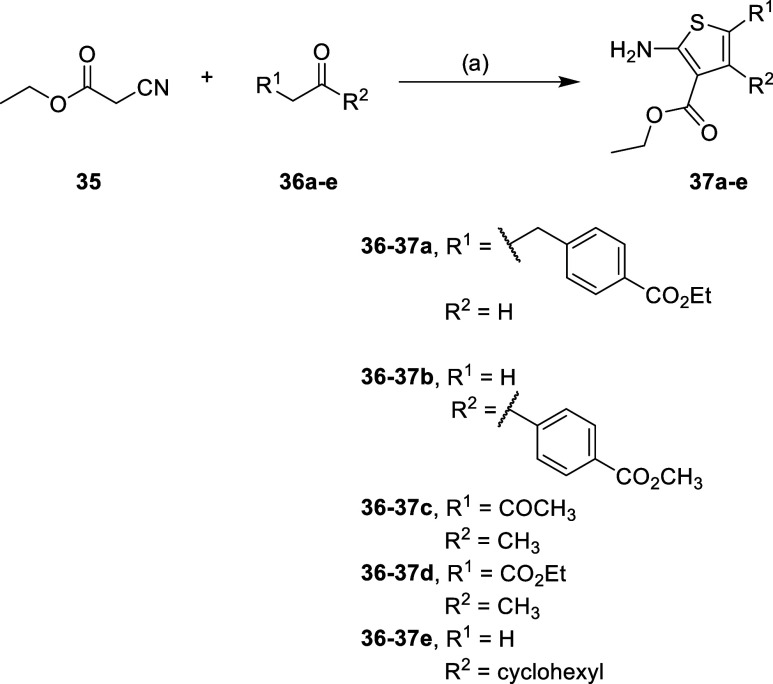
Synthesis Route for Compounds **37a**–**e** ^a^

a Reagents and conditions: (a) for **37a**–**d**: S_8_, morpholine, EtOH, 70 °C; for **37e**: S_8_, morpholine, room temperature (rt).

For the preparation of compounds **37g**–**m**, the substituted phenyl group was added via a Suzuki–Miyaura coupling from compound **39** (Scheme 2), prepared by bromination and protection of compound **38**.^39,40^ Finally, the amino group was deprotected, affording ethyl 2-aminothiophene-3-carboxylate intermediates **35g**–**i**.

**Scheme 2:**
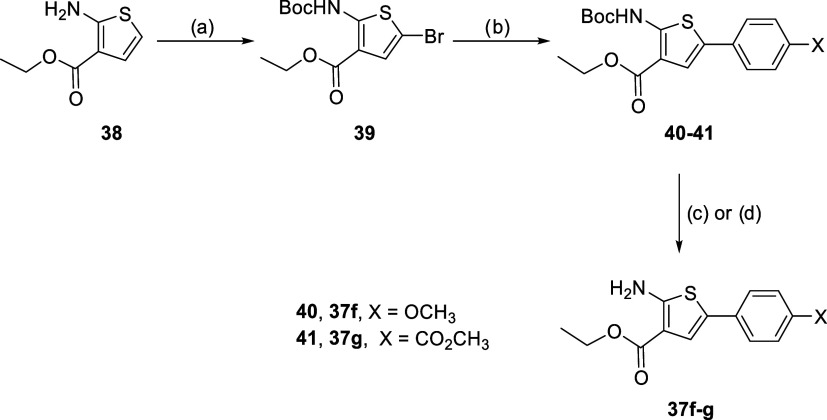
Synthesis Route for Compounds **37f**–**g** ^a^

a Reagents and conditions: (a) (i) Boc_2_O, 4-dimethylaminopyridine (DMAP), dioxane, 0–80 °C; (ii) *N*-bromosuccinimide (NBS), AcOH/dichloromethane (DCM), −15 °C (b) boronic acid, K_2_PO_3_, Pd­(PPh_3_)_4_, dimethylformamide (DMF)/H_2_O, 80 °C; (c) trifluoroacetic acid (TFA), DCM; (d) HCl 4 N in dioxane, MeOH.

Finally, the synthesized 2-aminothiophene-3-carboxylate intermediates (**37a**–**g**), and the commercially available ones (see Supporting Information (SI)), were reacted with benzoyl isothiocyanate **42** (Scheme 3). The ring closure was then performed in basic conditions under reflux.^41^ The synthesis of some final compounds required additional transformations, including hydrolysis (**18**, **24**, **25**, **30**, **S80**, and **S87**), reduction (**27** and **S79**), and amide coupling (**22–25**, **S81**, and **S83**). Full experimental details are provided in the Supporting Information.

**Scheme 3:**
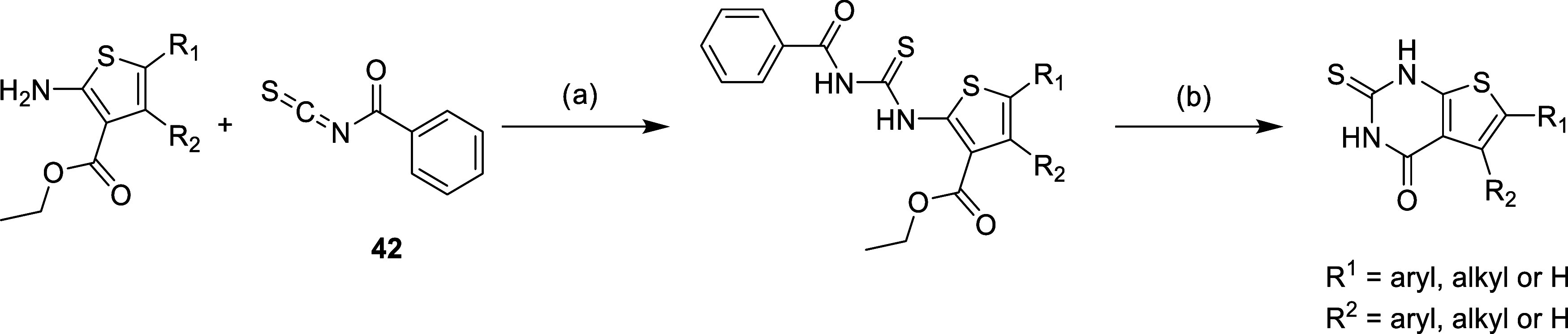
Synthesis Route for the Bicyclic Final Compounds^a^

a Reagents and conditions: (a) CH_3_CN, 45 °C; (b) EtONa, EtOH, reflux.

## Conclusions

We employed a fragment-based approach for identifying ligand-efficient small molecules that occupy the m^6^A-RNA recognition pocket of the DF2 reader domain. A crystal structure of a previously disclosed ligand-efficient fragment binder of DF2 (PDB 7R5W)^13^ and a molecular dynamics snapshot were used for fragment docking. Each of the two docking campaigns yielded three active compounds and, thus, an overall hit rate of 13% (6/47). SAR by catalogue and the synthesis of 32 derivatives of the thioxo-dihydrothienopyridinone scaffold **14** resulted in a series of ligand-efficient, low-micromolar binders of DF2. Most members of the series are selective against DC1 and DC2, while they bind with low-micromolar affinity also to DF1 and DF3. In contrast, compound **23** displays distinct selectivity as it binds exclusively to DF2 (*K*_d_ = 1.3 μM and IC_50_ = 11 μM measured by FP and HTRF, respectively). Using a similar fragment-based strategy, in our previous screening campaign for DF2 we identified 6-cyclopropyluracil as hit compound with an IC_50_ value of 170 μM.^13^ Compound **23** is more potent for DF2 than 6-cyclopropyluracil by a factor of 15, and is significantly more selective against DF1 and DF3. It is important to note that protein crystallography played a key role in both screening campaigns, as crystal structures were used for docking and molecular dynamics simulations. Moreover, in the present campaign the crystal structure of DF2 in the complex with compound **15** guided the derivatization of compound **14**, which has a similar scaffold and binding mode.

A few compounds (e.g., **17** and **18**) have a comparable affinity for the five YTH-containing proteins. Among them, compounds **17** and **30** are currently the most potent ligands of DC2 (*K*_d_ values of 6.3 and 4.9 μM, respectively, measured by FP). We have also presented the crystal structures of DF2 in complex with six ligands, which represent six distinct chemotypes (compounds **3**–**6**, **13**, and **15**). This structural data is useful for the development of a new series of ligands of the YTHDF m^6^A-RNA readers, and for the further training of machine learning models.^42^

## Materials and Methods

### Fragment Docking and Ranking

We used force field-based docking to identify small-molecule binders of the m^6^A reader YTHDF2. The structure of the YTHDF2 domain used for docking is the one in the complex with the ligand 6-cyclopropyl-1*H*-pyrimidine-2,4-dione (PDB code: 7R5W).^13^ We prepared the protein structure for docking using CAMPARIv5.^43^ The SEED^23,24,44^ docking program was used for rigid docking to the crystal structure itself and a snapshot obtained by MD simulations. These MD simulations were described in a previous study.^13^ After clustering the snapshots, a representative pose with a large aperture of the m^6^A binding site was selected (volume of 600 vs 324 Å^3^ in the crystal structure). The pocket volume was obtained by structural alignment of the crystal structure (PDB 7R5W) and the MD snapshot, and using the dpocket functionality of the fpocket tool with the ligand as reference.^45^ A library of 500,000 small molecules was considered for screening by SEED. The molecules were selected from the ZINC2020 database^25^ with the number of non-hydrogen atoms between 11 and 20, at least one ring and one sp^3^ carbon in the structure. For each of the extracted compounds, up to 20 conformers were generated using a distance geometry-based algorithm.^26^

The two protein structures were kept rigid during docking and evaluation of the binding energy. The residue C433 was selected for posing the fragments by SEED, which is similar to a pharmacophoric constraint. For the evaluation of the SEED energy, the binding site consisted of all of the DF2 residues within 20 Å of C433 and all charged side chains of DF2. A structural water molecule was also considered as part of the binding site because it is consistently resolved in all of the crystal structures obtained and is involved as a hydrogen-bond acceptor and donor with the side chains of W432 and D528, respectively.^13,27^ The partial charges and van der Waals parameters for the atoms in the protein and the small molecules were taken from the CHARMM36 all-atom force field^46–48^ and the CHARMM general force field (CGenFF),^49^ respectively. Importantly, the CHARMM36 force field and CGenFF are fully consistent in their partial charges and van der Waals parameters. The evaluation of the binding energy in the program SEED consists of a force field-based energy function with a continuum dielectric approximation of desolvation penalties by the generalized Born model.^50^ The values of the dielectric constant were 2.0 and 78.5 for the regions of the space occupied by the solute and solvent, respectively. Fragment screening by SEED requires about 1 s per fragment. SEED is available as an open-source code from GitLab (https://gitlab.com/CaflischLab).

From both docking campaigns, the compounds were ranked according to two energy terms calculated by SEED, namely, the total binding energy (SEED total) and the difference between the electrostatic contribution to the intermolecular interaction energy in the solvent and the solvation energy of the ligand (Delec). The top-scoring compounds were then selected if they showed the crucial hydrogen bond with the backbone carbonyl of C433 and a favorable interaction with the backbone NH of Y418, the side chain of D422, and/or the conserved water molecule. Finally, 47 compounds were purchased on the basis of commercial availability and structural diversity: 25 selected from the docking performed on the crystal structure and 22 from that on the MD snapshot with a large aperture of the binding site.

### HTRF Assay

GST-YTHDC1, GST-YTHDF1, GST-YTHDF2, and GST-YTHDF3 were purified as previously reported.^28^ The HTRF assay was assembled as detailed in ref 13 with the only difference being that the starting concentration of the dose–response experiments used for the IC_50_ determination was variated dependently from the tested compound. The same protocol was applied to the four proteins. The competitive inhibition data of GST-YTHDF1 (single dose experiment at 100 μM compound concentration and dose–response curves), GST-YTHDF3 (single dose experiment at 100 μM compound concentration and dose–response curves), and GST-YTHDF2 (single dose experiment at 100 μM compound concentration) were normalized as described in ref 29 to mitigate interference. The signal was measured as described in ref 29.

### GST-YTHDC2 Production

The N-terminally GST-tagged YTH domain of YTHDC2 (residues 1285–1424, cloned into the pGEX-6P-1 vector) was overexpressed in Rosetta (DE3) cells grown overnight at 20 °C following induction with 0.4 mM isopropyl-β-d-thiogalactopyranoside (IPTG) at an OD_600_ of 0.8. The cells were harvested and resuspended in a lysis buffer containing 100 mM Tris–HCl (pH 8.0) and 500 mM NaCl. After cell lysis, the lysate was clarified by centrifugation at 48,000*g* for 1 h, and the soluble proteins were loaded onto a column packed with Glutathione Sepharose 4B (GE Healthcare), then eluted with 20 mM reduced glutathione in lysis buffer. Finally, a size-exclusion chromatography step (HiLoad 16/600 Superdex 200 pg column, GE Healthcare) was performed to further purify the protein in 20 mM Tris–HCl (pH 7.4) and 150 mM NaCl.

### DNA-Fluorescence Polarization (FP) Assay

For fluorescence polarization (FP) experiments, a 5′-fluorescein-labeled m^6^A-DNA probe (5′-FAM-AAGAACCGG­(m6A)­CTAAGCT-3′) was synthesized by Microsynth AG.

The final concentration of the fluorescein-labeled DNA was kept constant at 3 nM. For YTHDC2 measurements, the protein concentration was set to 25 nM, while for YTHDF2 measurements, it was set to 10 nM, and for YTHDC1, it was set at 15 nM. The compound concentration was serially diluted to obtain the dose–response curve. The competition experiments were conducted in a final volume of 20 μL in a buffer containing 20 mM Tris–HCl (pH 7.4), 150 mM NaCl, and 0.01% bovine serum albumin (BSA), using a 384-well black flat-bottomed microplate (Corning 3575).

After incubating the mixture for 1 h, the anisotropy values were measured using a Tecan SPARK plate reader with 485/20 nm excitation and 535/25 nm emission polarization filters, suitable for fluorescein, at 25 °C. To obtain the equilibrium dissociation constant (*K*_d_), the IC_50_ values were first derived by fitting a dose–response curve to the data using nonlinear regression analysis in GraphPad Prism. These IC_50_ values were then converted to *K*_d_ by using the Cheng–Prusoff equation:

$$K_{d}=\frac{{\mathrm{IC}}_{50}}{1+\frac{\left[L\right]}{K_{d}^{\mathrm{probe}}}}$$

IC_50_ is the observed value from FP assay, [L] is the probe concentration used, and *K*_d_^probe^ is the binding affinity of probe for the target.

### YTHDF2 Protein Crystallography and Soaking

The YTH domain of YTHDF2 was expressed, purified, crystallized, and soaked as described in ref 13. The X-ray diffraction experiment was performed on the X06DA beamline of Paul Scherrer Institute’s Swiss Light Source. The resulting data were analyzed as described in ref 13.

### Molecular Dynamics Simulations and Clustering

We used molecular dynamics (MD) simulations to analyze the interactions between the YTHDF2 domain and compounds **14**, **17**, **23**, and **27**. Compound **14** (and its derivatives **17**, **23** and **27**) can, in principle, bind in two poses due to the symmetry of its thiourea ring. This was also observed in the docking results of compound **14**. Therefore, we defined two poses, A and B, depending respectively on the orientation of the exocyclic sulfur atom pointing toward the tryptophan cage or outward. We used the ParaLig^51^ software to modify the two docked poses of compound **14** to obtain derivatives **17** and **27**. The protein/ligand structures were then prepared using the software CAMPARIv5.^43^ Simulations were run using TIP3P (CHARMM) water model,^52^ with a 0.15 M concentration of Na^+^ and Cl^–^ ions. We equilibrated the systems first using an NPT ensemble to reach 300 K and 1 bar under 10 kJ/(mol Å^2^) positional restraints. We then applied four successive 1 ns NVT equilibrations with weakening restraints of 10, 5, 2.5, and 1.25 kJ/(mol Å^2^), respectively. All simulations were done using the CHARMM36m force field^53^ with the July 2022 GROMACS port. Production MD simulations consisted of eight independent runs for each compound and pose and a sampling of 200 ns per run. Production simulations were performed at the Swiss Supercomputing Center (CSCS) with the support of grant s1272 using GROMACS 2021.5.^54^

For clustering, we first subsampled the trajectories by selecting MD frames at every nanosecond of the simulation segments from 100 to 200 ns. In other words, we discarded the first half of each run for allowing the ligand to equilibrate in the binding pocket. The bound frames were selected according to the distance between the N atom of C433 and the thiourea sulfur atom and choosing frames with a distance lower than 5 Å. We applied PCA to a set of 10 protein–ligand distances to reduce the data to two dimensions. These 10 distances involved atoms of the thiourea ring and the residues C433 (four distances), D422 (two distances), and Y418, W432, W486, and W491 (one distance each). The Gaussian mixture algorithm with full covariance was employed for clustering the two-dimensional data in PC space. The MD snapshot closest to the center of the cluster (centroid) was used as a representative pose. The root-mean-square deviation (RMSD) of the ligand in the binding pocket was calculated for all of the trajectories. First, all MD snapshots were overlapped with the equilibrated starting structure using the Cα atoms of the protein. Then the coordinates of the heavy atoms of the ligand were employed for the calculation of the RMSD. All analyses were done using MDTraj^55^ and SciKit learn.^56^

## Supplementary Material



## Notes

Supplementary figures, tables, materials, synthetic procedures, characterization data, ^1^H and ^13^C NMR spectra, HPLC traces for all final compounds, and X-ray data collection and refinement statistics for the 6 complex YTH-YTHDF2 crystal structures (PDF)

## Abbreviations

YTHDF: YT521-B homology domain family
m^6^A: *N* ^6^-methyladenosine
YTHDC: YTH domain containing
oligoRNA: oligoribonucleotide
HTRF: homogeneous time-resolved fluorescence
LE: ligand efficiency
FP: fluorescence polarization
PCA: principal component analysis
RMSD: root-mean-square deviation
