# Supplementary material for: Discovery of YTHDF2 Ligands by Fragment-Based Design
Source: ACS Bio Med Chem Au. 2025 Jun 27;5(4):753–65. doi: 10.1021/acsbiomedchemau.5c00099 (PMC12371494; doi:10.1021/acsbiomedchemau.5c00099)

Supporting information for

Discovery of YTHDF2 ligands by fragment-based design

Annalisa Invernizzi, Francesco Nai, Rajiv Kumar Bedi, Pablo Andrés Vargas-Rosales, Yaozong Li, Elena Bochenkova, Marcin Herok, František Zálešák, Amedeo Caflisch*

Department of Biochemistry, University of Zurich, Winterthurerstrasse 190, CH-8057, Zurich, Switzerland

* Corresponding author

TABLE OF CONTENTS

Supplementary Tables 3

Table S1. Purchased compounds from the high-throughput docking campaign. 3

Table S2. Compounds purchased for SAR by catalogue. 7

Table S3. Purchased modifications and/or expansions of compound **14**. 10

Table S4. Synthesized expansions of compound **14**. 12

Table S5. Single-dose residual signal at 100 µM 14

Table S6. X-ray data collection and refinement statistics 15

Supplementary figures 17

Figure S1. YTHDF2 HTRF dose-response curves. 17

Figure S2. YTHDF2 fluorescence polarization (FP) dose-response curves 21

Figure S3. YTHDC2 fluorescence polarization (FP) dose-response curves. 23

Figure S4. YTHDC1 fluorescence polarization (FP) dose-response curves. 25

Figure S5. YTHDF1 and YTHDF3 HTRF dose-response curves. 27

Figure S6. Alternative poses of compounds **17**, **23** and **27**. 27

Figure S7. RMSD analysis of compound **23** in complex with DC1. 28

Chemistry 29

Material and Methods 29

Compounds source and purity 30

Experimental section 31

References 48

^{1}H and ^{13}C NMR spectra of final compounds 49

HPLC traces of final compounds 81

SUPPLEMENTARY TABLES

Table S1. Purchased compounds from the high-throughput docking campaign.

Compound nr.	2D Structure	Residual signal at 1 mM concentration (%)[a]	IC_{50} [µM][a]	PDB code Resolution [Å]	SEED total/Delec[b] [kcal/mol]
**1**	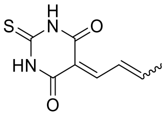	26	19		-15.9 -1.4
**2**	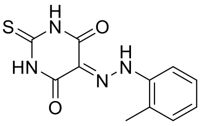	12	170		-23.0 -5.4
**3**	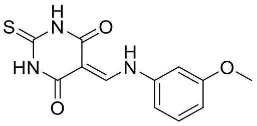	NA*	52 % at 125 µM #	9QEM (2.26)	-24.0 -3.5
**4**	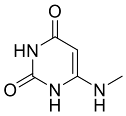	18	250	9QEL (1.86)	-17.5 -9.0
**5**	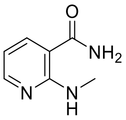	88		9QEO (1.98)	
**6**	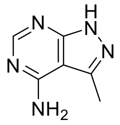	80		9QFI (1.91)	
**S1**	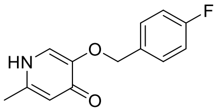	31	310		-12.4 -5.5
**S2**	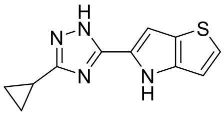	44	170		-20.5 -3.0
**S3**	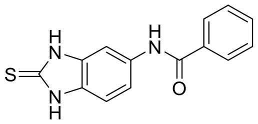	53	410		
**S4**	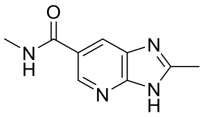	27	540		

**S5**	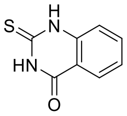	16*	610			
**S6**	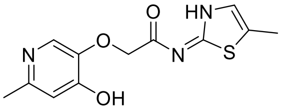	60			-11.6	-7.8
**S7**	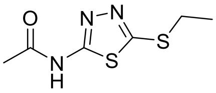	60			-13.5	1.4
**S8**	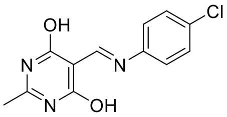	21*			-17.4	1.4
**S9**	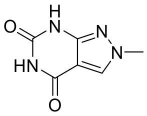	42*			-20.3	-4.1
**S10**	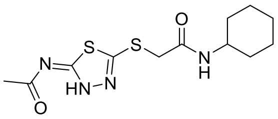	50*			-14.3	3.9
**S11**	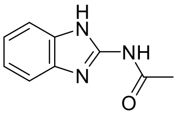	59*				
**S12**	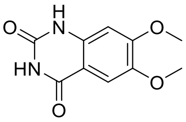	60*				
**S13**	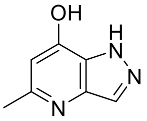	62			-15.5	-2.6
**S14**	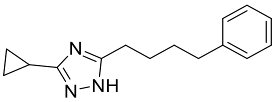	70				
**S15**	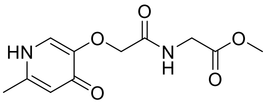	75			-16.9	-10.2
**S16**	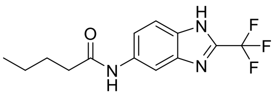	76				
**S17**	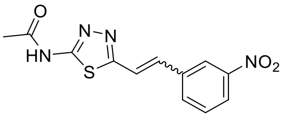	77			-23.0	-1.8

**S18**	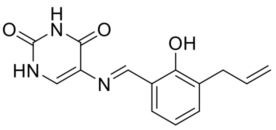	79			-10.7 9.5
**S19**	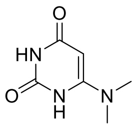	85			
**S20**	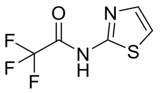	87			-14.1 -5.0
**S21**	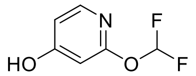	87			-6.8 -2.3
**S22**	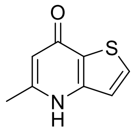	89			-13.2 -3.8
**S23**	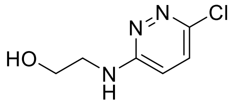	> 90			-15.0 -5.8
**S24**	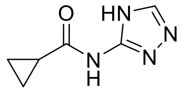	> 90			-10.8 0.7
**S25**	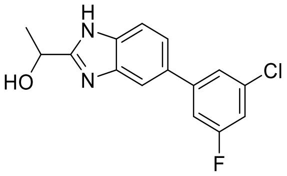	> 90			-19.0 -2.6
**S26**	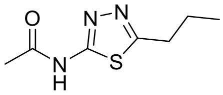	> 90			-13.5 1.4
**S27**	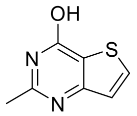	> 90			-12.9 3.6
**S28**	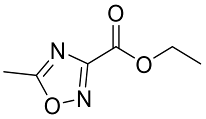	> 90			-12.7 -3.4
**S29**	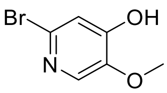	> 90			-14.4 -4.8
**S30**	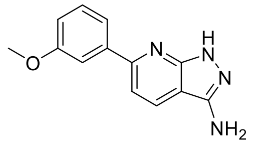	> 90			-18.1 -3.7

**S31**	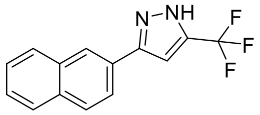	> 90			-19.3 -2.0
**S32**	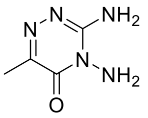	> 90			-9.1 -5.5
**S33**	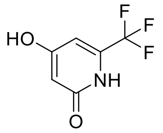	> 90			-9.5 -7.4
**S34**	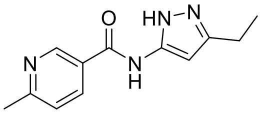	> 90			-10.7 10.1
**S35**	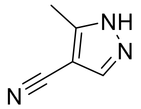	> 90			
**S36**	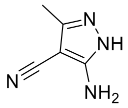	> 90			
**S37**	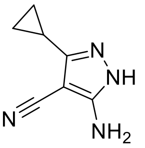	> 90			-17.4 -1.4
**S38**	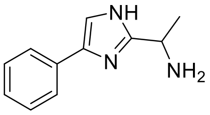	> 90			
**S39**	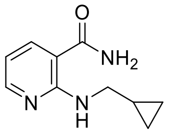	> 90			
**S40**	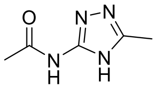	> 90			
**S41**	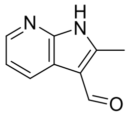	> 90			

[a]Measured using the HTRF-based binding assay reported previously.[1] [b]Energy values calculated by SEED (see Methods). Values are not reported when the docked compound was not commercially available and a close analogue was purchased instead (see below). *The compound shows interference with the assay at the tested concentration. # Interference or poor solubility observed at higher concentrations, IC_{50} could not be determined.

SMILES and energies (SEED total/Delec in kcal/mol) of the corresponding docked compounds, for each ordered analogue:
Compound 5: O=C1C2=C(N=CC=C2)NCN1 (-22.7/-11.2)
Compound 6: CC1=NNC2=C1C(N)=NC(C3=CC=NC=C3)=N2 (-17.0/-3.9)
Compound S3: O=C(COCC(F)(F)F)NC1=CC=C2C(NC(N2)=S)=C1 (-17.6/-0.5)
Compound S4: O=C(C1=CC2=C(NC(C)=N2)N=C1)NC3=NC(C)=NS3 (-17.0/-1.0)
Compound S5: S=C1NC2=C(C(N1)=O)C=C(C)C=C2 (-18.2/-2.4)
Compound S11: CC1=NC2=C(N1)C=CC(C(N)=O)=C2 (-16.9/-0.4)
Compound S12: CC(NC1=CC=C2C(C(NC(N2)=O)=O)=C1)=O (-16.0/-4.9)
Compound S14: C12=C(NC(C3=NNC(C4CC4)=N3)=C2)C=CS1 (-20.5/-3.0)
Compound S16: CC1=NC2=CC(NC(COC)=O)=CC=C2N1 (-18.9/-1.7)
BrC1=CC=C2NC(C(F)(F)F)=NC2=C1OC (-18.9/-4.3)
Compound S19: CNC1=CC(NC(N1)=O)=O (-17.5/-9.0)
Compound S35: N#CC1=C(NN=C1C2=COC=C2)CC (-18.4/-3.5)
Compound S36: NC1=C(C#N)C(C2CC2)=NN1 (-17.4/-1.4)
Compound S38: CC(C1=NC2=C(C=CC(C3=CC=CC=C3)=C2)N1)O (-19.0/-2.6)
Compound S39: Compound 5
Compound S40: O=C(NC1=NNC(C)=C1)C2=CC=NN2CC3=CSC=C3 (-20.2/-0.3),
O=C(NC1=NN=C(N1)CC)C2=CC(F)=CN2 (-18.2/0.8)
Compound S41: CC1=CC2=C(N=CC(C=O)=C2Cl)N1 (-15.2/-1.2)

Table S2. Compounds purchased for SAR by catalogue.

Compound nr.	2D Structure	Residual signal at 1 mM concentration (%)[a]	IC_{50} [µM][a]	PDB code
**S42**	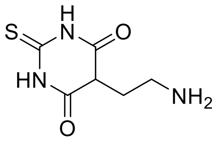		330	
**S43**	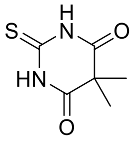	11		
**S44**	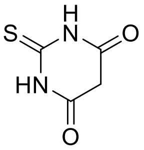	79		
**S45**	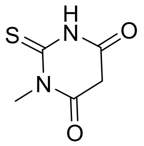	75		
**S46**	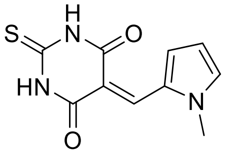	13	NA*	

**S47**	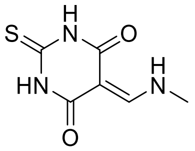	NA*	91	
**S48**	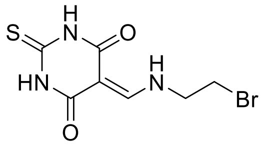		53% at 100 μM	
**S49**	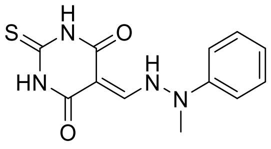	54		
**S50**	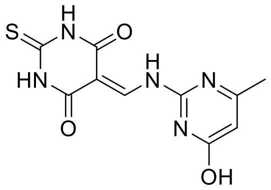	9	57	
**S51**	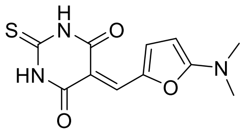	35		
**S52**	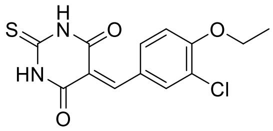	12	55 (0.29)	
**S53**	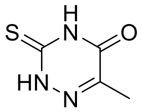	38		
**S54**	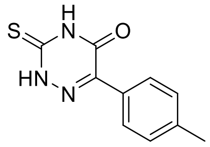		53% at 62.5 μM	
**S55**	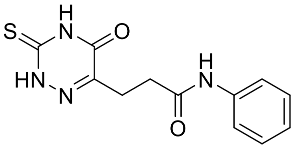		190	
**S56**	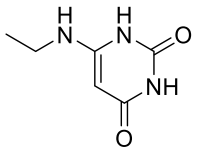	51		
**S57**	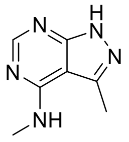	88		

**S58**	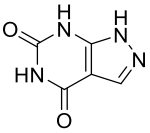	47		
**S59**	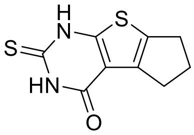	35	53	

Data for compounds **7-16** are shown in Table 1. [a] Measured using the HTRF-based binding assay reported previously.[1] * The compound shows interference with the assay at the tested concentration.

Table S3. Purchased modifications and/or expansions of compound **14**.

**Compound nr.**	**2D structure**	**Residual signal at 1 mM concentration (%)[a]**	**IC50 [µM][a]**
**S60**	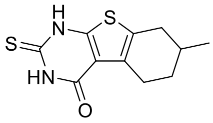	24	80
**S61**	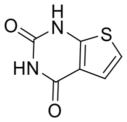	-	290
**S62**	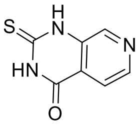	-	170
**S63**	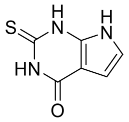	28	49% at 100 µM
**S64**	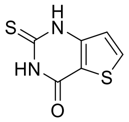	NA*	36% at 100 µM
**S65**	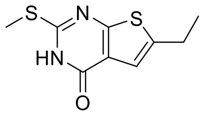	NA*	68% at 100 µM
**S66**	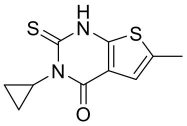	23	25% at 100 µM
**S67**	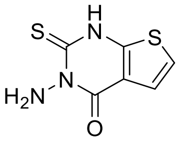	23	40% at 100 µM
**S68**	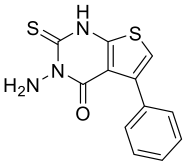	36	32% at 100 µM
**S69**	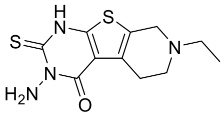	NA*	23
**S70**	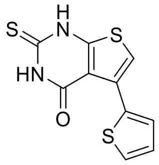	49	45% at 100 µM

**S71**	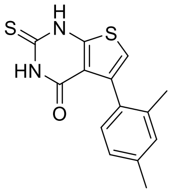	11	34% at 100 µM
**S72**	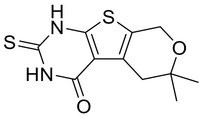	10	14<br>(0.39)
**S73**	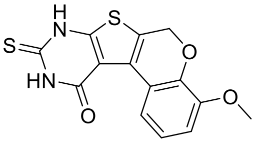	64	22% at 100 µM

[a] Measured using the HTRF-based binding assay reported previously.[1] * The compound shows interference with the assay at the tested concentration.

Table S4. Synthesized expansions of compound **14**.

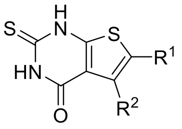

**Compound nr.**	**R^1**	**R^2**	**HTRF-assay IC$_{50}$ DF2 [μM]**	**FP K$_d$ DF2 [μM]**	**FP K$_d$ DC2 [μM]**
**14**	H	H	18	3.8	88
**S74**	H	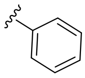	94	-*	> 1000
**17**	H	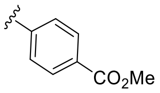	4.7	1.2	6.3
**18**	H	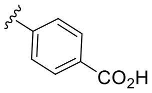	5.4	0.61	7.7
**S75**	H	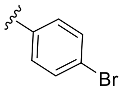	27	8.8	37
**S76**	H	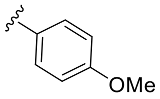	24	11	54
**19**	H	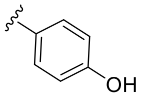	32	1.3	40
**S77**	H	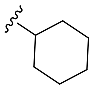	33	5.6	240
**S78**	CH_3	H	38	-*	> 1000
**20**	$COCH_3$	CH_3	13	3.1	46
**S79**	$CH(OH)CH_3$	CH_3	27	-*	> 1000
**21**	CO_2Et	CH_3	6.3	4.4	230
**S80**	CO_2H	CH_3	32	-*	-*
**22**	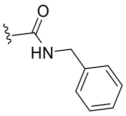	CH_3	10	10	270
**23**	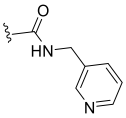	CH_3	11	1.3	450
**S81**	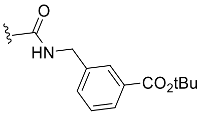	CH_3	34	5.0	22

**24**	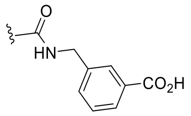	CH_3	20	2.5	41
**S82**	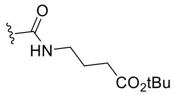	CH_3	22	-*	> 1000
**25**	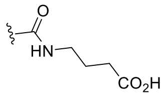	CH_3	14	1.3	130
**S83**	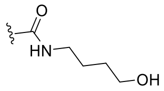	CH_3	25	-*	> 1000
**S84**	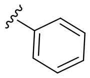	H	40	8.1	34
**26**	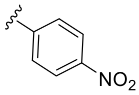	CH_3	4.9	17	35
**27**	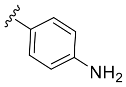	CH_3	3.9	1.9	30
**28**	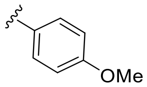	H	22	1.9	9.1
**29**	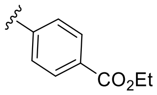	H	21	3.1	8.4
**30**	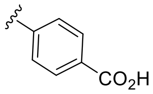	H	16	1.9	4.9
**S85**	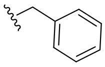	H	33	6.2	160
**S86**	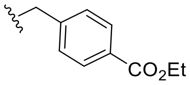	H	63% at 125 µM	-*	210
**S87**	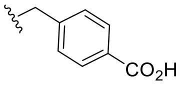	H	29	18	100
**S88**	H	CH_3	28	8.1	120
**S89**	H	CO_2Et	62	7.5	450
**S90**	H	CO_2H	60	10	240
**31**	H	$CONH_2$	14	1.9	18

* not determined.

Table S5. Single-dose residual signal at 100 µM.[a]

Compound nr.	DF1 (%)	DF2 (%)	DF3 (%)
**14**	15	8	23
**17**	7	4	7
**18**	3	4	19
**19**	8	5	13
**20**	8	3	12
**21**	12	5	10
**22**	17	5	18
**23**	20	9	33
**24**	5	3	12
**25**	6	7	26
**26**	12	7	15
**27**	6	0	5
**31**	27	13	36
**S75**	9	4	8
**S76**	12	6	12
**S77**	32	12	35
**S78**	17	8	22
**S79**	31	9	30
**S80**	15	9	29
**S81**	15	4	10
**S82**	18	5	20
**S83**	17	9	40
**S84**	32	11	25
**S85**	34	13	35
**S89**	16	8	16
**S90**	36	14	34

[a] Measured using the HTRF-based binding assay reported previously.[1]

Table S6. X-ray data collection and refinement statistics for the 6 complex YTH-YTHDF2 crystal structures as generated by Phenix "Generate "Table 1" for journal". Deposited PDB and MTZ files were used as input, along with unmerged intensities. Numbers in parentheses refer to the highest resolution shell.

	9QEM	9QEL	9QEO
**Wavelength**	1	1	1
**Resolution range**	43.28 - 2.262 (2.38 - 2.26)	44.1 - 1.857 (1.91 - 1.86)	40.28 - 1.977 (2.05 - 1.98)
**Space group**	P 65	P 65	P 65
**Unit cell**	80.06 80.06 110.8 90 90 120	80.47 80.47 113.89 90 90 120	80.56 80.56 114.37 90 90 120
**Total reflections**	170974 (25582)	240177 (20246)	266741 (27767)
**Unique reflections**	37091 (5319)	63911 (5275)	57778 (5800)
**Multiplicity**	4.6 (4.8)	3.8 (3.8)	4.6 (4.8)
**Completeness (%)**	99.98 (100.00)	99.01 (99.80)	98.76 (99.73)
**Mean I/sigma(I)**	5.99	10.74	15.58
**Wilson B-factor**	58.55	40.14	34.25
**R-merge**	0.1379 (1.325)	0.0681 (1.462)	0.05135 (0.3709)
**R-meas**	0.156 (1.485)	0.07909 (1.687)	0.05802 (0.4178)
**R-pim**	0.07218 (0.6664)	0.03964 (0.8327)	0.02673 (0.1906)
**CC1/2**	0.992 (0.552)	0.996 (0.413)	0.998 (0.908)
**CC***	0.998 (0.843)	0.999 (0.764)	1 (0.976)
**Reflections used in refinement**	18830 (2689)	34875 (2935)	29041 (2923)
**Reflections used for R-free**	943 (135)	1744 (147)	1451 (147)
**R-work**	0.2186 (0.2941)	0.2174 (0.3233)	0.2124 (0.2459)
**R-free**	0.2598 (0.3607)	0.2484 (0.3848)	0.2582 (0.3082)
**Number of non-hydrogen atoms**	2342	2520	2594
**macromolecules**	2255	2387	2411
**ligands**	56	35	49
**solvent**	31	98	134
**Protein residues**	288	297	300
**RMS(bonds)**	0.008	0.006	0.007
**RMS(angles)**	0.89	0.79	0.84
**Ramachandran favored (%)**	96.83	96.93	96.62
**Ramachandran allowed (%)**	3.17	3.07	3.38
**Ramachandran outliers (%)**	0.00	0.00	0.00
**Rotamer outliers (%)**	1.76	1.24	0.82
**Clashscore**	4.08	2.15	2.75
**Average B-factor**	59.09	42.59	39.56
**macromolecules**	58.69	42.31	39.21
**ligands**	76.81	53.94	53.93
**solvent**	56.15	45.36	40.59

	9QFI	9QIU	9QFL
**Wavelength**	1	1	1
**Resolution range**	42.96 - 1.905 (1.97 - 1.91)	44.3 - 2.462 (2.65 - 2.46)	44.05 - 1.705 (1.74 - 1.7)
**Space group**	P 65	P 65	P 65
**Unit cell**	79.14 79.14 110.27 90 90 120	79.44 79.44 115.8 90 90 120	79.72 79.72 114.43 90 90 120
**Total reflections**	227761 (20711)	102855 (21464)	404801 (23221)
**Unique reflections**	56170 (5012)	29444 (5851)	88577 (5387)
**Multiplicity**	4.1 (4.1)	3.5 (3.7)	4.6 (4.3)
**Completeness (%)**	99.23 (99.78)	99.37 (98.80)	99.82 (97.47)
**Mean I/sigma(I)**	14.21	5.62	11.64
**Wilson B-factor**	45.22	50.95	32.44
**R-merge**	0.03999 (0.7234)	0.1768 (1.021)	0.05521 (1.445)
**R-meas**	0.04603 (0.8282)	0.2102 (1.19)	0.0624 (1.643)
**R-pim**	0.02254 (0.3988)	0.1119 (0.6037)	0.02884 (0.7727)
**CC1/2**	0.999 (0.689)	0.987 (0.656)	0.999 (0.452)
**CC***	1 (0.903)	0.997 (0.89)	1 (0.789)
**Reflections used in refinement**	30344 (2774)	14935 (2969)	44778 (2734)
**Reflections used for R-free**	1518 (139)	746 (148)	2241 (136)
**R-work**	0.2245 (0.2972)	0.2230 (0.2723)	0.2038 (0.3668)
**R-free**	0.2558 (0.3809)	0.2801 (0.3624)	0.2222 (0.4425)
**Number of non-hydrogen atoms**	2432	2459	2577
**macromolecules**	2303	2359	2386
**ligands**	46	47	43
**solvent**	83	53	148
**Protein residues**	295	297	299
**RMS(bonds)**	0.008	0.008	0.006
**RMS(angles)**	0.91	0.90	0.74
**Ramachandran favored (%)**	96.56	94.54	97.97
**Ramachandran allowed (%)**	3.44	5.46	2.03
**Ramachandran outliers (%)**	0.00	0.00	0.00
**Rotamer outliers (%)**	0.88	1.67	0.00
**Clashscore**	4.48	5.21	3.85
**Average B-factor**	50.62	52.98	35.36
**macromolecules**	50.58	52.80	34.88
**ligands**	58.02	67.17	48.41
**solvent**	47.79	48.45	39.29

SUPPLEMENTARY FIGURES

Figure S1. YTHDF2 HTRF dose-response curves.

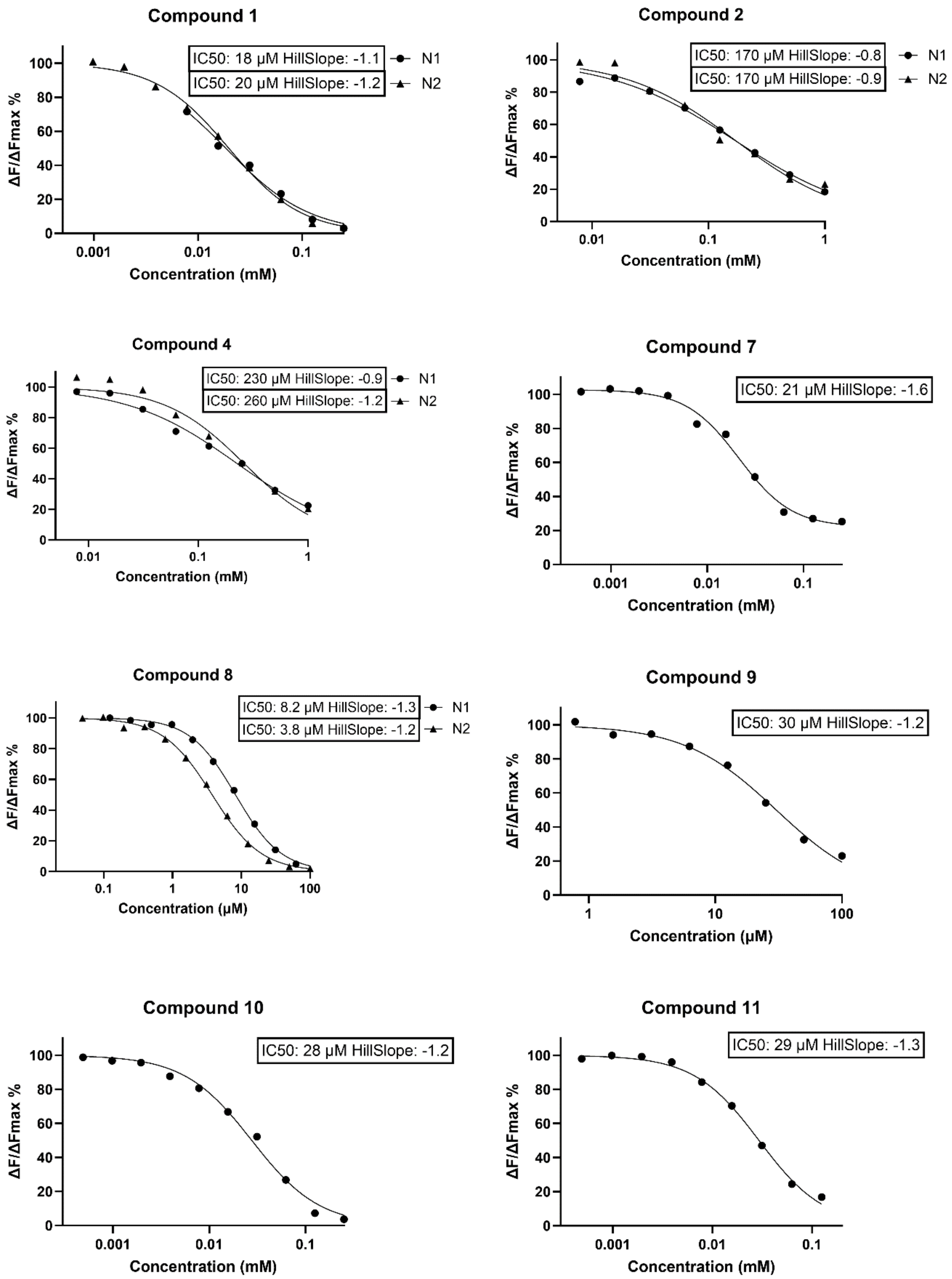

Compound 12

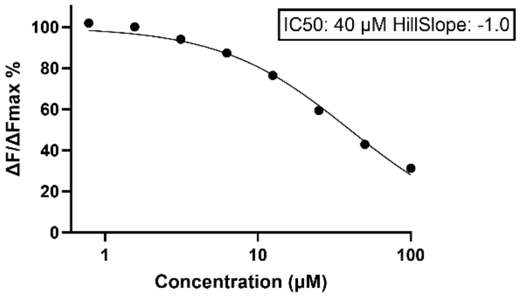

Compound 13

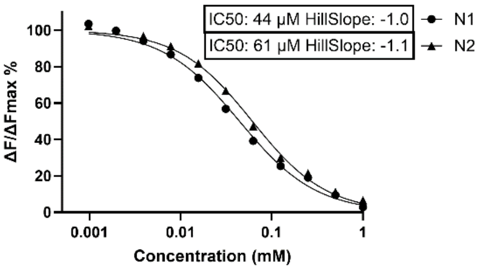

Compound 14

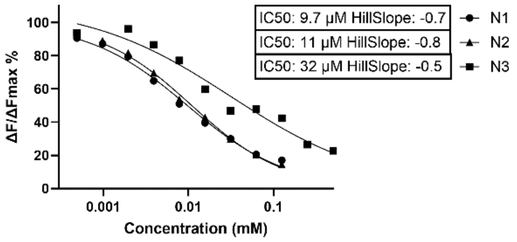

Compound 15

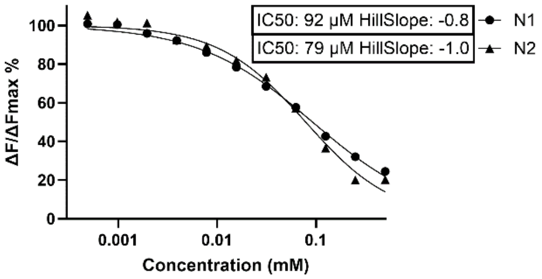

Compound 16

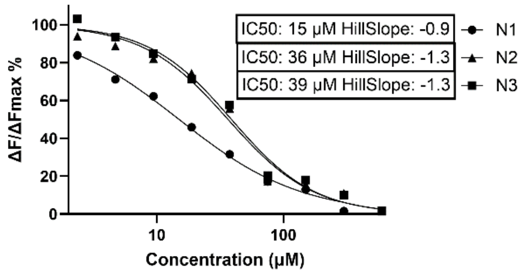

Compound 17

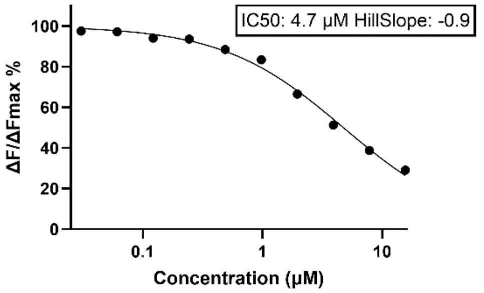

Compound 18

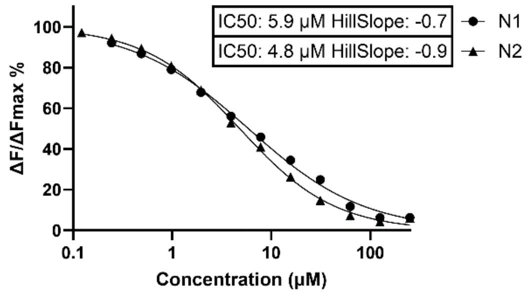

Compound 19

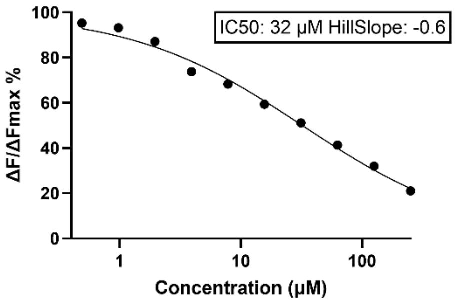

Compound 20

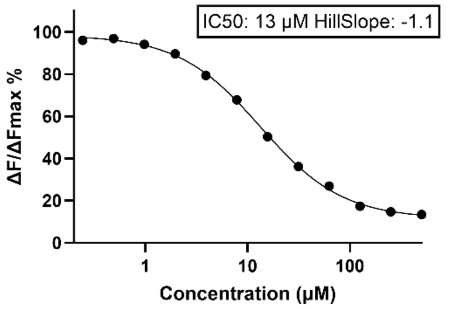

Compound 21

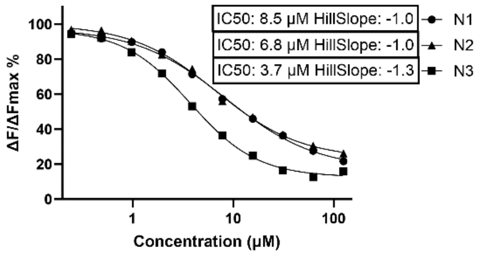

Compound 22

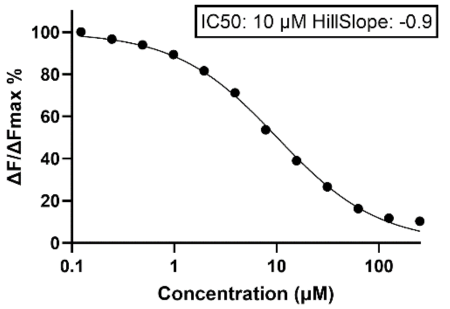

Compound 23

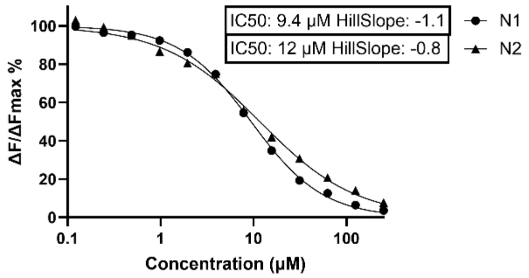

Compound 24

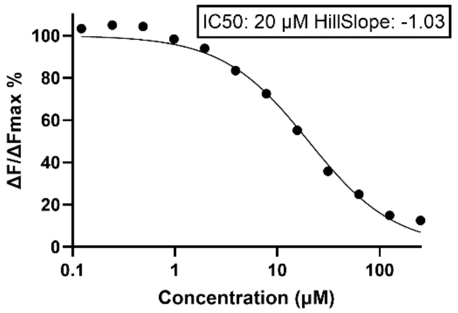

Compound 25

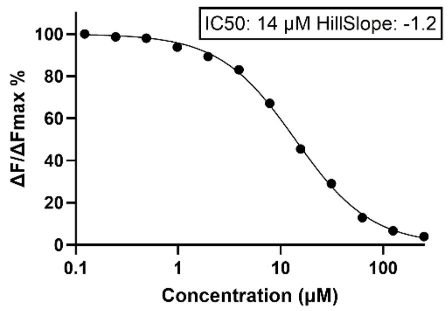

Compound 26

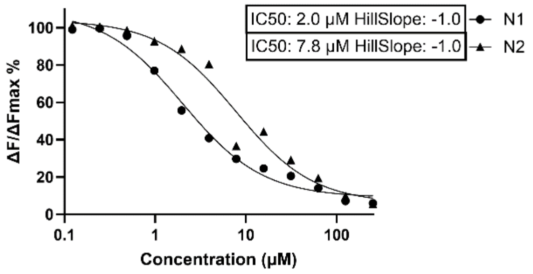

Compound 27

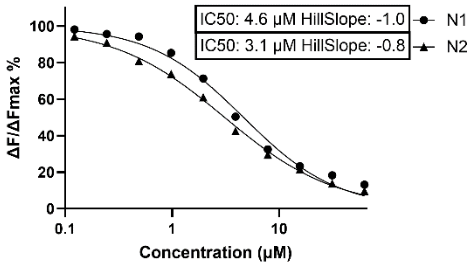

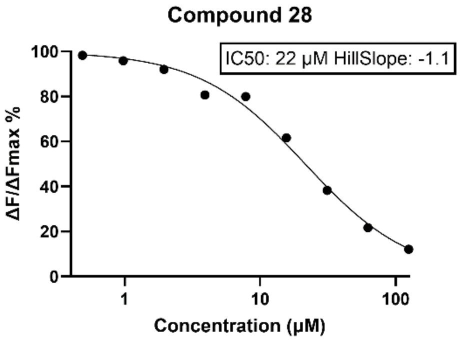

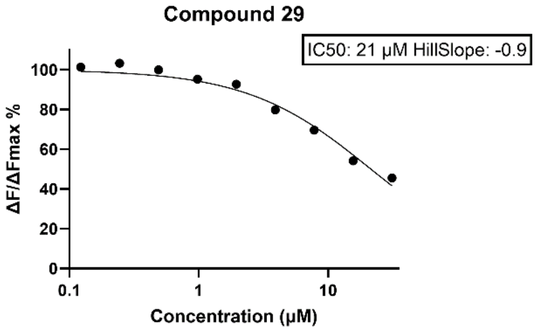

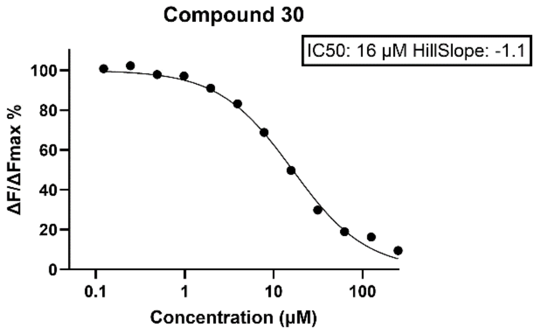

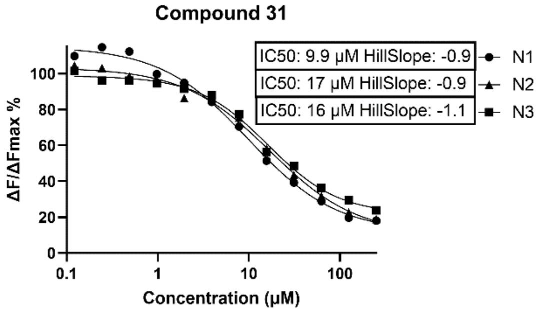

Each curve represents one biological replicate, and each biological replicate is the average of two technical replicates. The final IC_{50} value (Tables 1 and 2) is calculated as the average of the IC_{50} values obtained from each curve.

Figure S2. YTHDF2 fluorescence polarization (FP) dose-response curves.

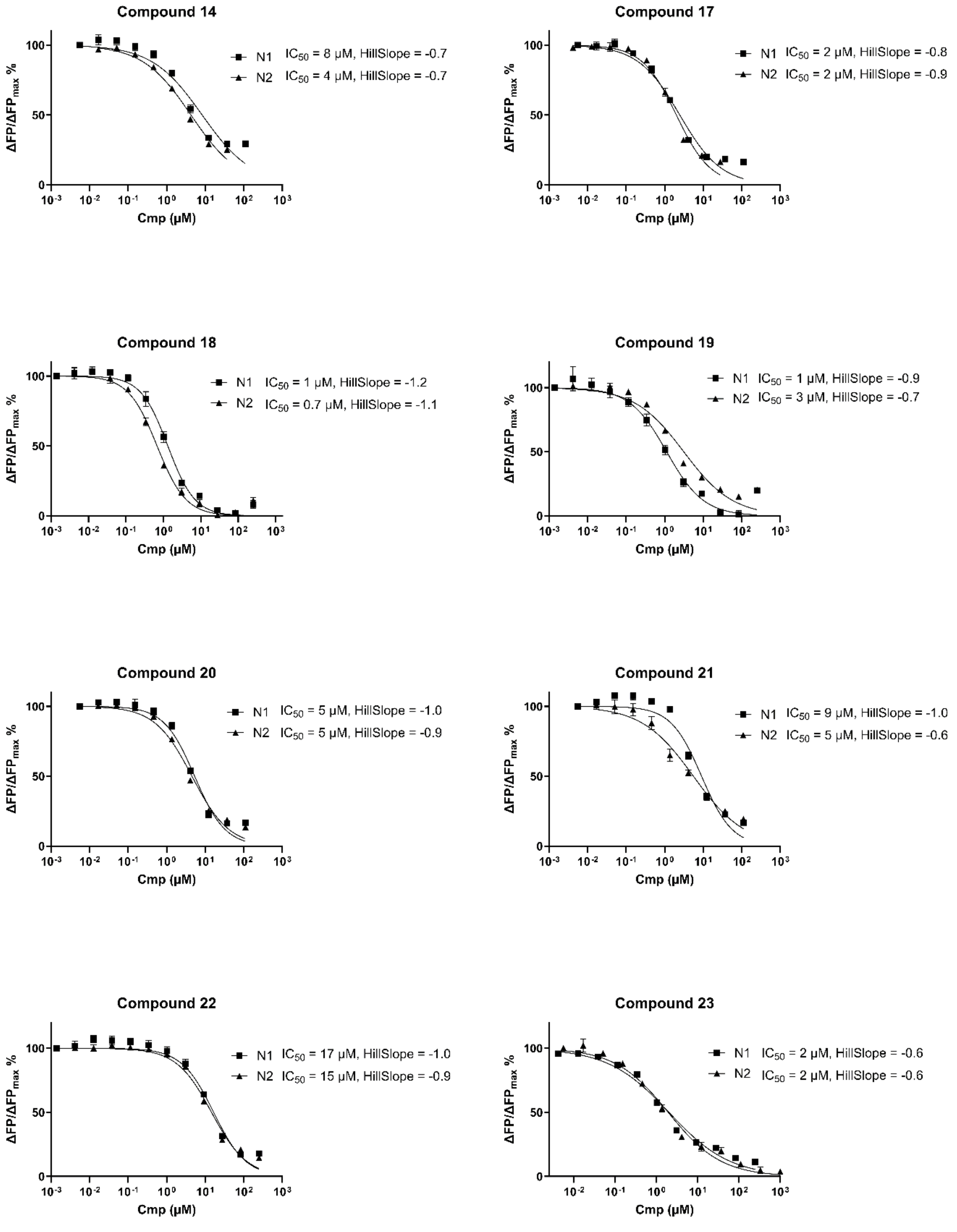

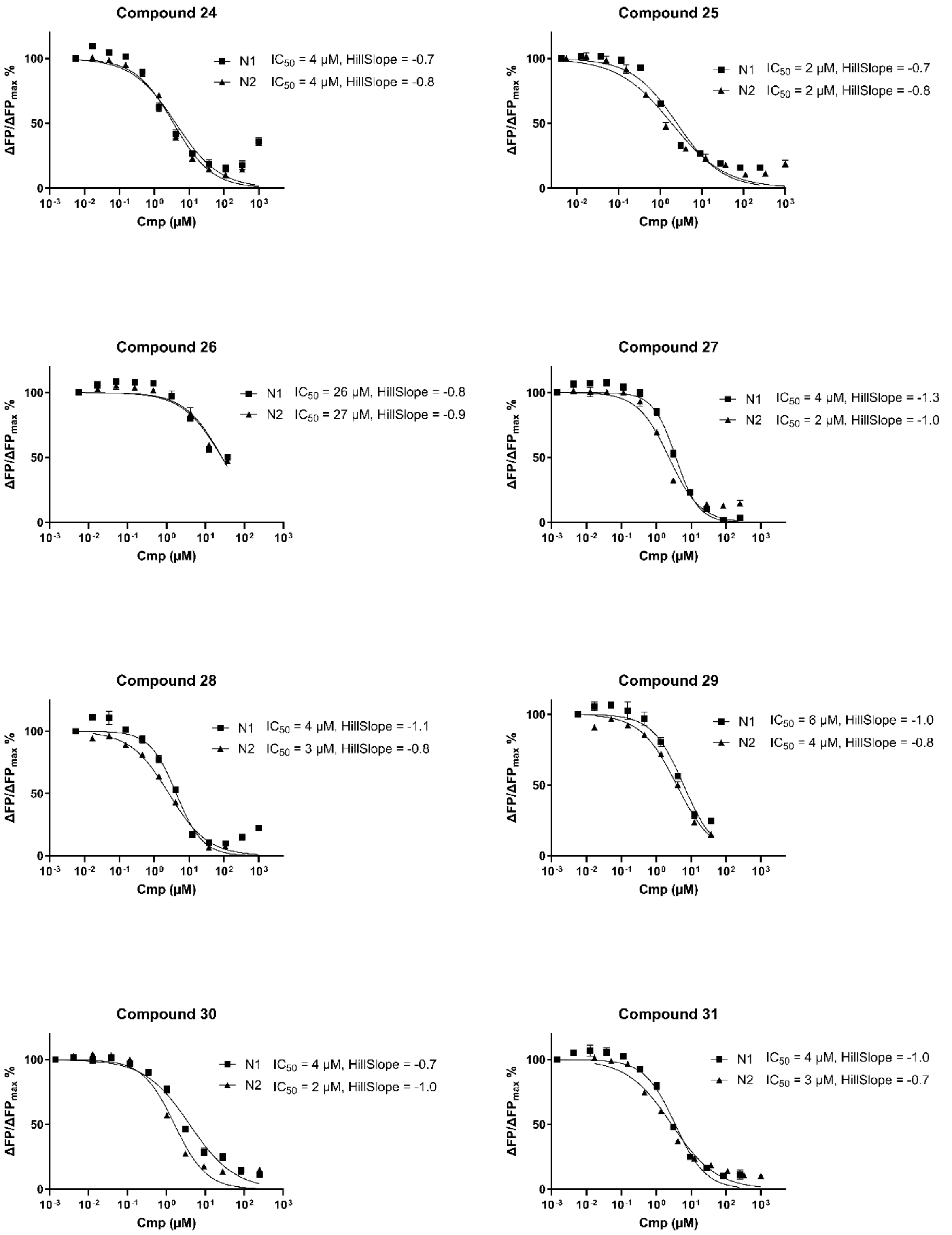

For each compound two curves are shown corresponding to two biological replicates, respectively. Each biological replicate is the average of four technical replicates. The error bars represent the standard deviation for the technical replicates. The final IC_{50} value is calculated as the average of the two IC_{50} values obtained from the two curves. The value is then used to calculate the K_d reported in Table 2 (see Methods)

Figure S3. YTHDC2 fluorescence polarization (FP) dose-response curves.

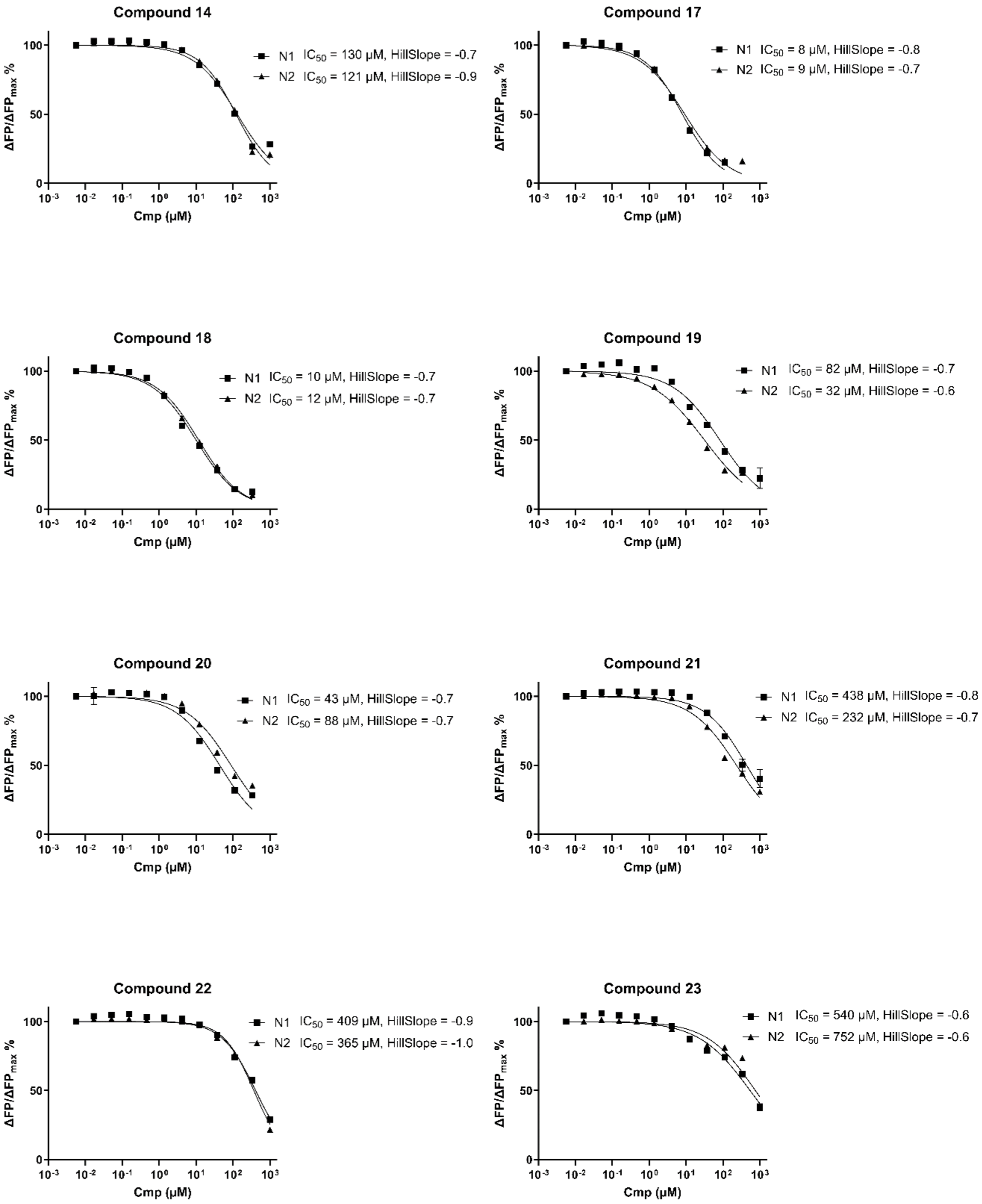

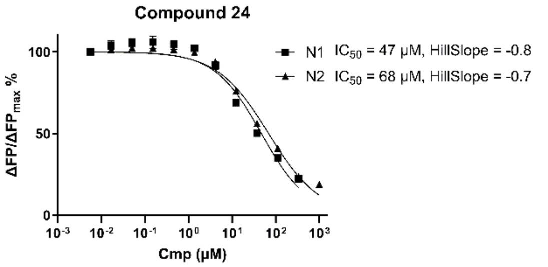

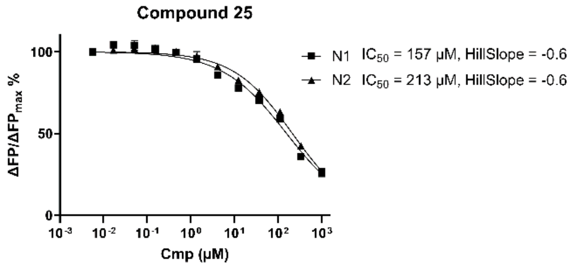

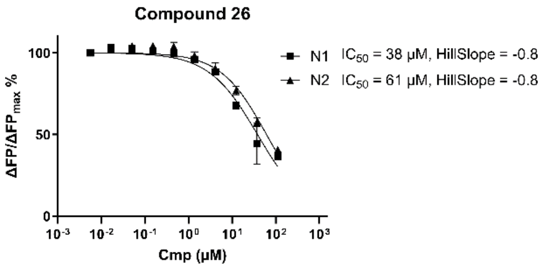

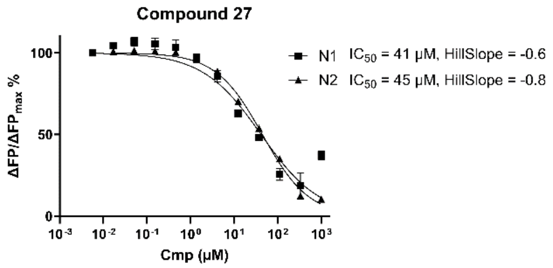

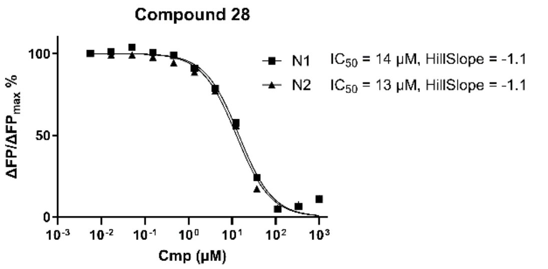

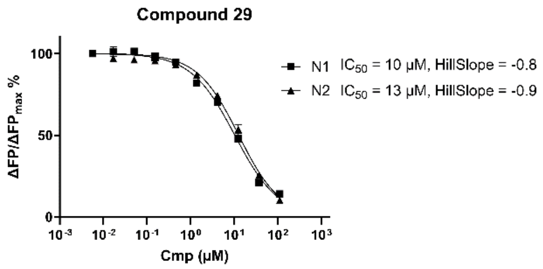

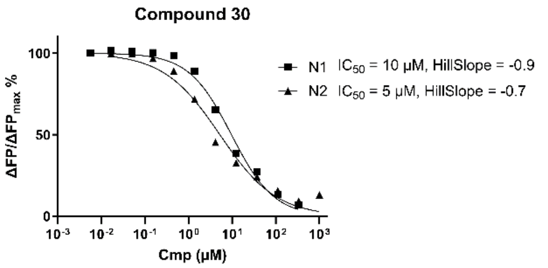

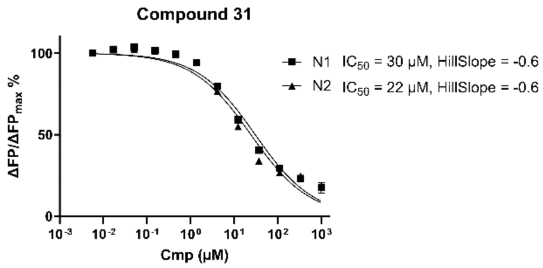

For each compound two curves are shown corresponding to two biological replicates, respectively. Each biological replicate is the average of four technical replicates. The error bars represent the standard deviation for the technical replicates. The final IC_{50} value is calculated as the average of the two IC_{50} values obtained from the two curves. The value is then used to calculate the K_d reported in Table 2 (see Methods)

Figure S4. YTHDC1 fluorescence polarization (FP) dose-response curves.

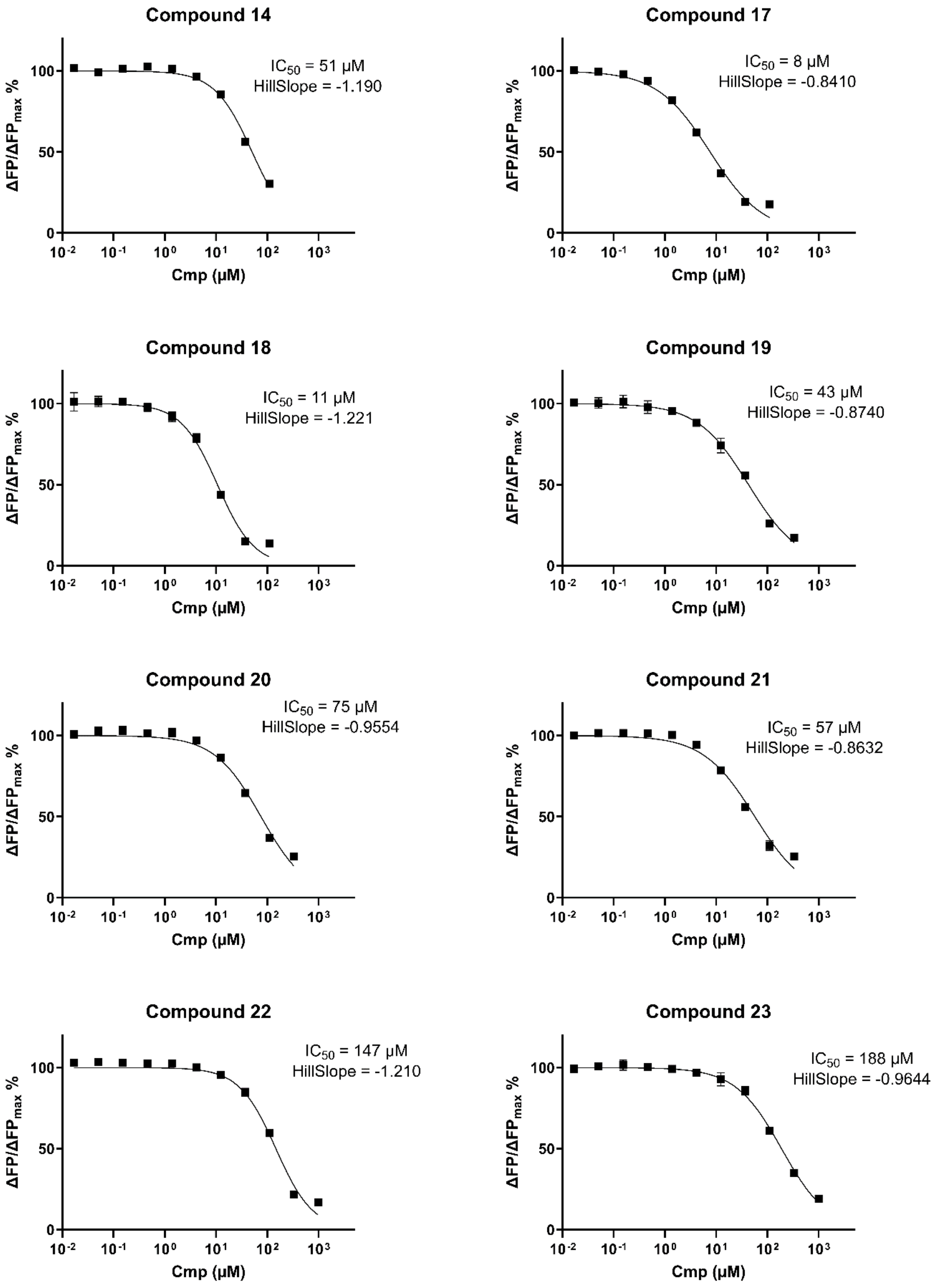

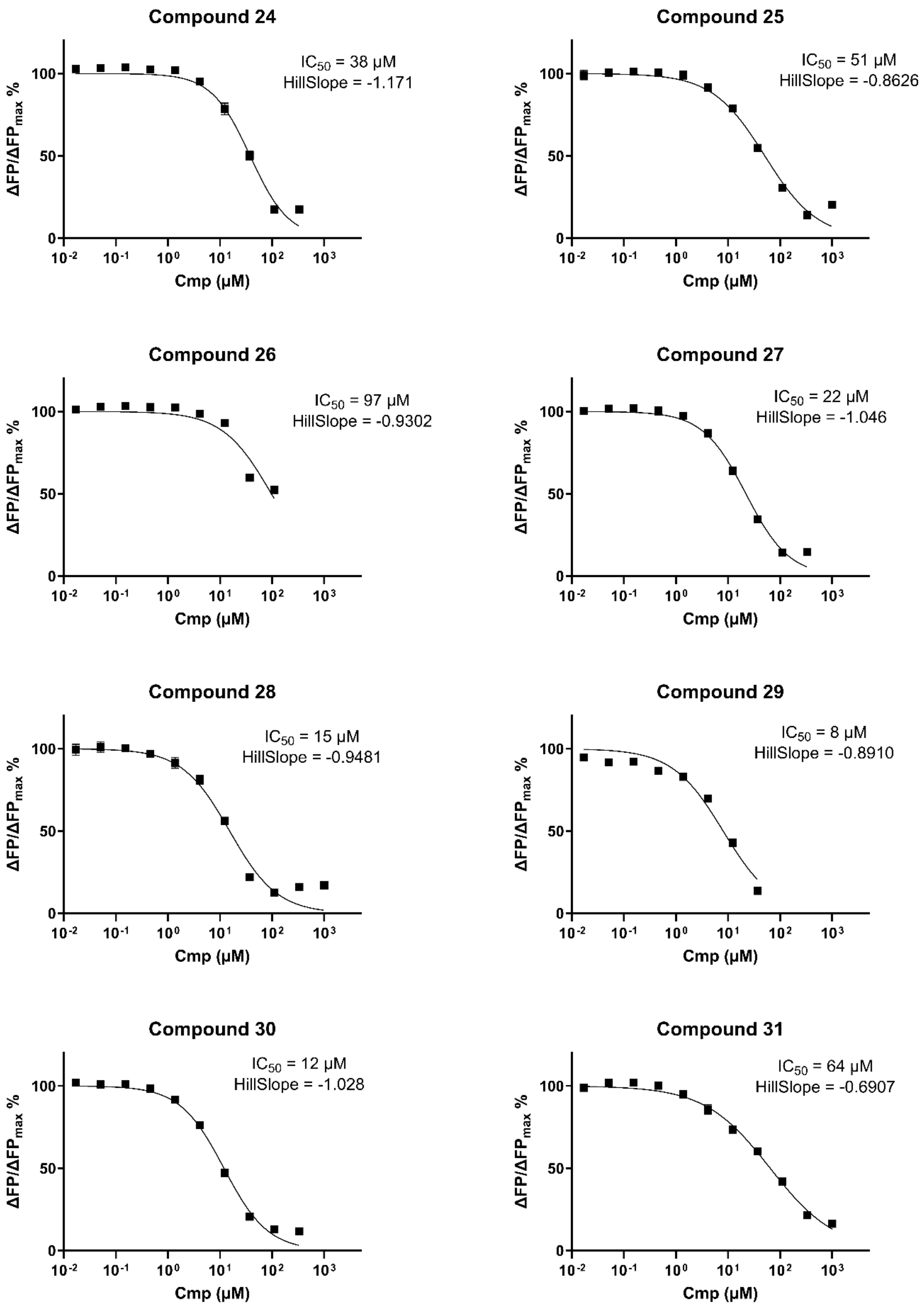

For each compound two curves are shown corresponding to two biological replicates, respectively. Each biological replicate is the average of four technical replicates. The error bars represent the standard deviation for the technical replicates. The final IC_{50} value is calculated as the average of the two IC_{50} values obtained from the two curves. The value is then used to calculate the K_d reported in Table 2 (see Methods)

Figure S5. YTHDF1 and YTHDF3 HTRF dose-response curves.

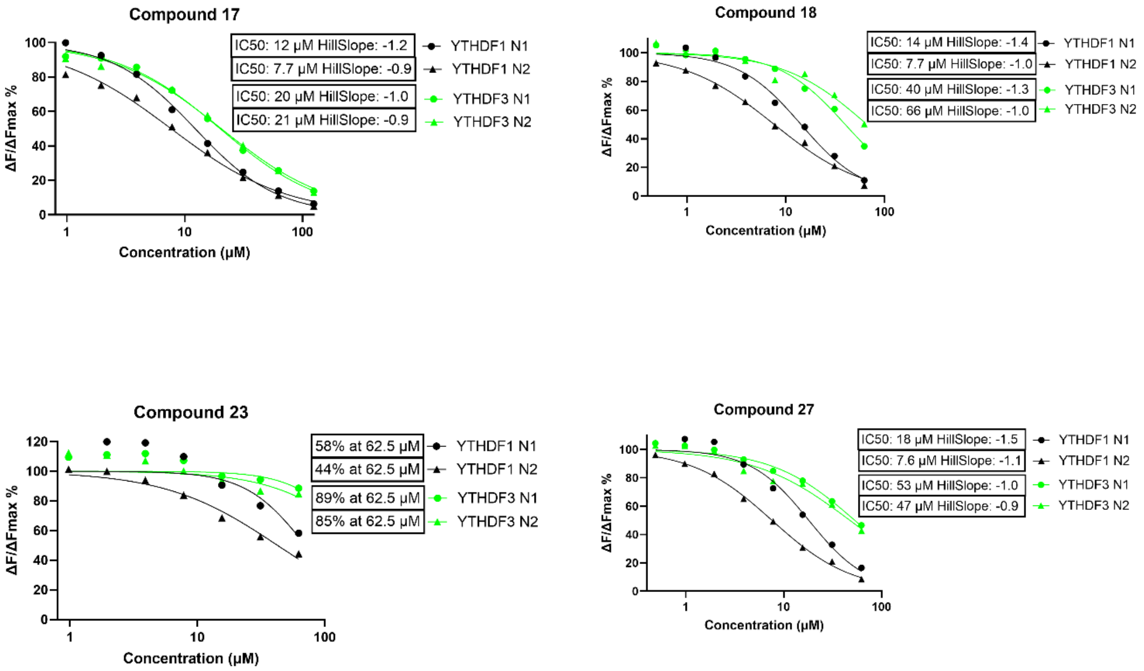

Each curve represents one biological replicate, and each biological replicate is the average of two technical replicates. The final IC_{50} value (Table 2) is calculated as the average of the IC_{50} values obtained from each curve.

Figure S6. Alternative poses of compounds **17**, **23** and **27**.

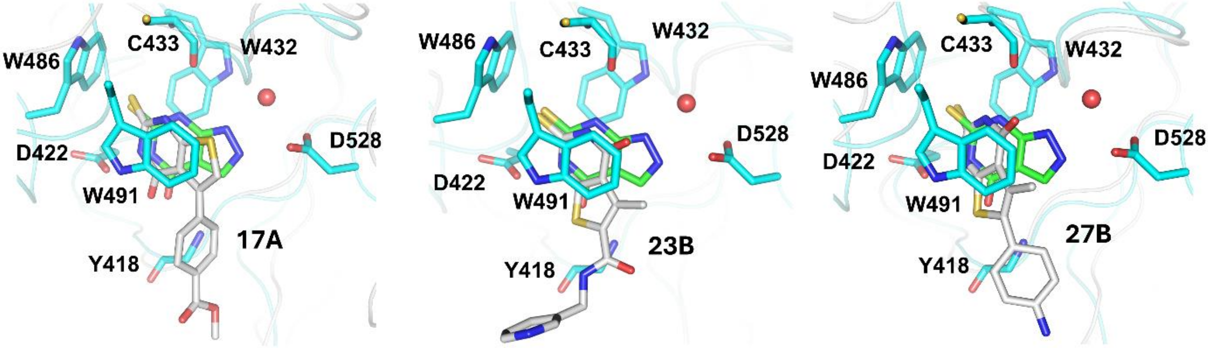

From left to right: compound **17** pose A, compound **23** pose B and compound **27** pose B (carbon atoms shown in grey). The crystal structure of DF2 (cyan) in complex with fragment **15** (carbon atoms in green, PDB: 9QFL) is overlapped for comparison. The main poses are shown in Figure 3b-d.

Figure S7. RMSD analysis of compound **23** in complex with DC1.

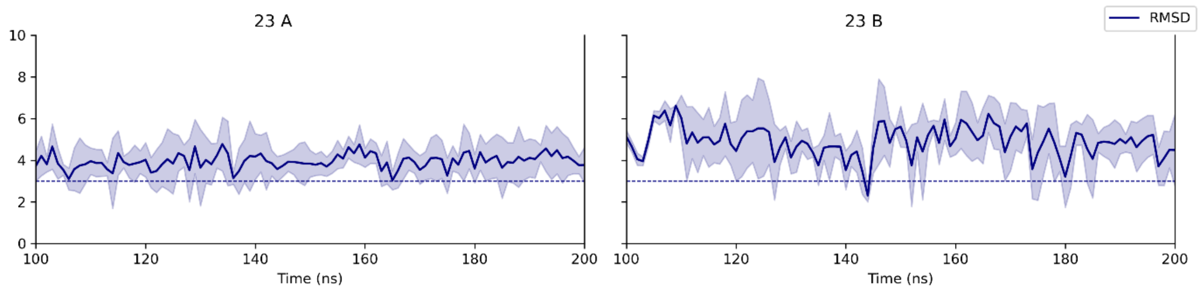

Analysis of the molecular dynamics simulations (MD) started from the two potential poses (pose A, left; pose B, right). The time series show the median ligand root mean square deviation (RMSD) with respect to the first frame, with a colored band corresponding to one median absolute deviation around the median.

CHEMISTRY

Material and Methods

All reagents were purchased from commercial suppliers and used as received. Reactions run at elevated temperatures were carried out in the oil bath. All reactions were monitored by thin-layer chromatography (Aluminium plates coated with silica gel 60 F_{254}). Flash column chromatography was carried out over silica gel (0.040-0.063 mm) or aluminium oxide (0.050-0.200 mm). SiliaMetS® TAAcONa (or SiliaMetS® Triaminetetraacetate, sodium salt) is a silica-bound metal scavenger for Pd(II), Ni(II) and Cu. It is the supported version of EDTA in its sodium salt form. ^{1}H and ^{13}C {^{1}H} NMR spectra were recorded on AV2-400 MHz, AV2-500 and AV-600 Bruker spectrometers (400 MHz, 101 MHz, 500 MHz, 126 MHz, and 600 MHz, 150 MHz, respectively) in DMSO-d_6, $CDCl_3$ or MeOD-d_4. Chemical shifts are given in ppm and their calibration was performed to the residual ^{1}H and ^{13}C signals of the deuterated solvents. Multiplicities are abbreviated as follows: singlet (s), doublet (d), triplet (t), multiplet (m), and broad signal (bs). The purity was acquired by Liquid chromatography high-resolution electrospray ionization mass spectrometry (LC-HR-ESI-MS): *Acquity UPLC* (*Waters*, Milford, USA) connected to an *Acquity* eλ diode array detector and a *Synapt G2* HR-ESI-QTOF-MS (*Waters*, Milford, USA); injection of 1 µL sample (c = ca. 10-100 µg/mL in the indicated solvent); *Acquity BEH C18* HPLC column (1.7 µm particle size, 2.1 × 50 mm, *Waters*) kept at 30°C; elution at a flow rate of 400 µL/min with A: H_2O + 0.02% TFA and B: CH_3CN + 0.02% TFA, linear gradient from 10–95% B within 3 min, then isocratic 95% B for 2 min; UV spectra recorded from 190–300 nm at 1.2 nm resolution and 20 points s^{-1}; ESI: positive ionization mode, capillary voltage 3.0 kV, sampling cone 40V, extraction cone 4V, N_2 cone gas 4 L/h, N_2 desolvation gas 800 L/min, source temperature 120°C; mass analyzer in resolution mode: mass range 100–2'000 *m/z* with a scan rate of 1 Hz; mass calibration to <2 ppm within 50–2'500 m/z with a 5mM aq. soln. of HCO_2Na, lockmasses: *m/z* 195.0882 (caffein, 0.7 ng/mL) and 556.2771 (Leucine-enkephalin, 2 ng/mL). The HPLC analyses were performed on a Shimadzu LC − 9A HPLC system equipped with a Shimadzu SPD − 6A VP UV − Vis detector; Phenomenex RP-HPLC on a Phenomenex InertClone (5 µm particle size, 4.6 mm × 150 mm i.d.). HPLC purifications were performed on a Shimadzu LC − 8A HPLC system equipped with a Shimadzu SCL − 10A VP System control and a Shimadzu SPD − 10A VP UV − Vis detector on a Phenomenex Gemini C18-110A preparative column (10-µm particle size, 250 mm × 21.2 mm i.d.).

For all isolated intermediates and final compounds ^{1}H, ^{13}C NMR and LCMS characterization is included to the synthetic procedures, if they were not previously reported in the literature. For all final compounds ^{1}H, ^{13}C NMR spectra and HPLC traces are shown in the Supporting Information.

Compounds source and purity

Compounds **5-6, 13, 14, 16, S3-5, S11, S12, S19, S20, S22, S33-42, S45, S54-58, S61, S63-S73** were purchased from Chemspace and have a guaranteed purity ≥ 95%. Compounds **1-4, 7-12, 15, S1, S2, S6-S10, S13-S18, S21, S23-S32, S43, S44, S46-S53, S59, S60, S62** were purchased from Mcule and have a guaranteed purity ≥ 90%. The synthesized final compounds **17-31** and **S74-S90** have a purity ≥ 95% assessed by HPLC, HPLC traces can be found further on in the Supporting Information.

Experimental section

GENERAL PROCEDURE A: Gewald reaction (synthesis of compounds 37a-d)

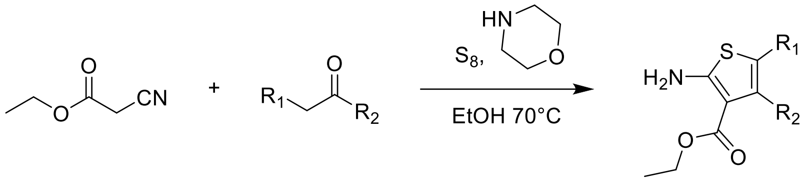

To a solution of ethyl cyanoacetate (1 equivalent) in absolute ethanol (0.5 M), the corresponding carbonyl compound (1 equivalent), sulfur powder (1 equivalent), and morpholine (1 equivalent) were added sequentially. The resulting mixture was stirred and heated at 70 °C (oil bath) until full reaction completion (as monitored by TLC). Volatiles were removed under reduced pressure, and the crude product was purified by flash column chromatography (SiO_2), resulting in the corresponding 2-aminothiophene derivative.

GENERAL PROCEDURE B

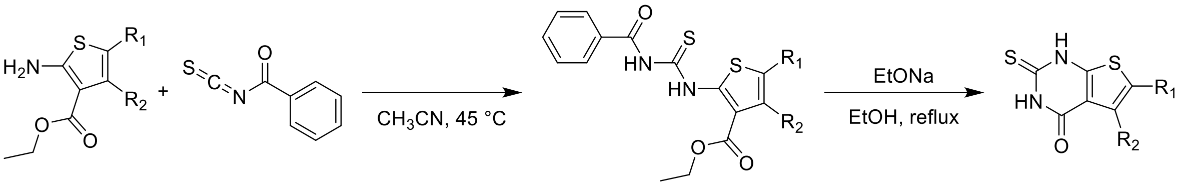

To a heated solution (45 °C) of ethyl 2-aminothiophene-3-carboxylate derivative in MeCN (0.5 M) was added benzoyl isothiocyanate. The reaction mixture was maintained at 45 °C with continuous stirring for 3 hours. After completion, the resulting precipitate was collected by filtration, washed with CH_3CN, and air-dried.

The thiourea derivative was used in the next step without additional purification and suspended in absolute ethanol, and sodium ethoxide (2 equivalents) was added (if not mentioned differently). The reaction mixture was refluxed for 24 hours. Afterward, the mixture was cooled to room temperature and acidified to pH 3 by adding 38% HCl. The resulting precipitate was collected by filtration, washed thoroughly with water, and air-dried. Adapted from Ref.[2]

GENERAL PROCEDURE C: Suzuki coupling (synthesis of compounds 40 and 41)

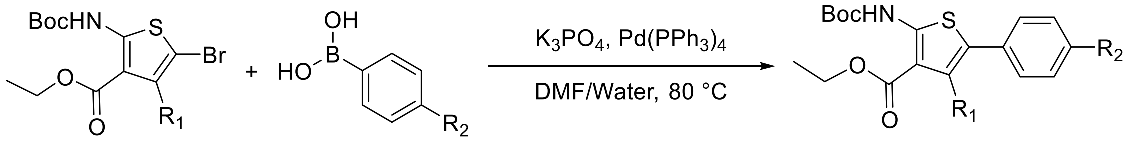

The starting material was dissolved in a 4:1 mixture of DMF and aqueous 2M K_3PO_4 under N_2-atmosphere. The appropriate boronic acid (1.2 eq.) and $Pd(PPh_3)_4$ (3 mol%) were added and the reaction mixture was stirred at 80 °C for 24 h. The solution was cooled to room temperature, diluted

with water and extracted with EtOAc. The combined organic layers were washed with a 10% LiCl solution 3 times, dried with $MgSO_4$, filtered, and the solvent was removed *in vacuo*. The crude product was purified by flash column chromatography (SiO_2) providing the corresponding product. Procedure from Ref.[3]

ethyl 2-amino-5-(4-(ethoxycarbonyl)benzyl)thiophene-3-carboxylate ***37a***

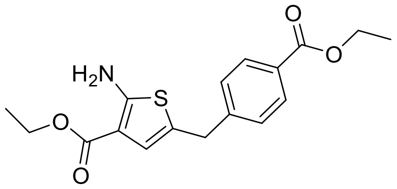

Compound **37a** was prepared according to General procedure A using ethyl 4-(3-oxopropyl)benzoate (500 mg, 2.42 mmol) in absolute ethanol (5 mL). The reaction was stirred at reflux for 2 hours. The volatiles were removed *in vacuo* and purified via flash column chromatography (SiO_2; Hept/EtOAc 3:1 to 2:1) affording 547 mg of desired product (68 % yield). ^{1}H NMR (400 MHz, DMSO) δ 7.92 – 7.88 (m, 2H), 7.41 – 7.33 (m, 2H), 7.16 (s, 2H), 6.61 (s, 1H), 4.30 (q, J = 7.1 Hz, 2H), 4.14 (q, J = 7.1 Hz, 2H), 3.95 (s, 2H), 1.31 (t, J = 7.1 Hz, 1H), 1.23 (t, J = 7.1 Hz, 1H). As reported in lit.[4]

ethyl 2-amino-4-(4-(methoxycarbonyl)phenyl)thiophene-3-carboxylate ***37b***

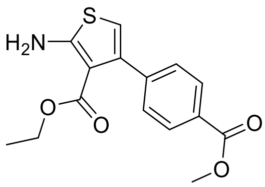

Compound **37b** was prepared according to General procedure A using methyl 4-acetylbenzoate (400 mg, 2.24 mmol) in absolute EtOH (8 mL). The reaction was stirred for 48 hours at reflux. The reaction was stirred at reflux for 2 hours. The volatiles were removed *in vacuo* and purified via flash column chromatography (SiO_2; 9:1 Hept/EtOAc) affording 336 mg of desired product (49 % yield). ^{1}H NMR (400 MHz, $CDCl_3$) δ 7.99 (dd, J = 8.1, 1.4 Hz, 2H), 7.37 (dd, J = 8.1, 1.4 Hz, 2H), 6.10 (s, 1H), 4.08 – 3.98 (m, 2H), 0.98 – 0.89 (m, 3H).

ethyl 5-acetyl-2-amino-4-methylthiophene-3-carboxylate ***37c***

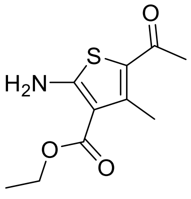

Compound **37c** was prepared according to General procedure A using pentane-2,4-dione (354 mg, 3.54 mmol) in absolute ethanol (8 mL). The reaction was stirred for 16 hours at 70 °C. The volatiles were removed *in vacuo* and purified via flash column chromatography (SiO_2; from 100% Hept to 3:2

Hept/EtOAc) affording 458 mg of desired product (56 % yield). ^{1}H NMR (400 MHz, $CDCl_3$) δ 6.60 (bs, 2H), 4.32 (q, *J* = 7.0 Hz, 2H), 2.70 (s, 3H), 2.43 (s, 3H), 1.38 (t, *J* = 7.1 Hz, 3H).

diethyl 5-amino-3-methylthiophene-2,4-dicarboxylate ***37d***

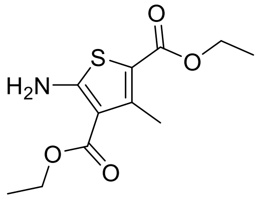

Compound **37d** was prepared according to General procedure A using ethyl 3-oxobutanoate (920 mg, 7.07 mmol) in absolute ethanol (15 mL). The reaction mixture was stirred at 70 °C for 16 hours. The volatiles were then removed *in vacuo* and purified via flash column chromatography (SiO_2; from 100% Hept to 3:2 Hept/EtOAc) affording 1.79 mg of desired product (98 % yield). ^{1}H NMR (400 MHz, $CDCl_3$) δ 6.46 (bs, 2H), 2.70 (s, 3H), 4.31 (q, *J* = 7.0 Hz, 2H), 4.25 (q, *J* = 7.0 Hz, 2H), 1.37 (t, *J* = 7.0 Hz, 3H), 1.33 (t, *J* = 7.0 Hz, 3H).

ethyl 2-amino-4-cyclohexylthiophene-3-carboxylate ***37e***

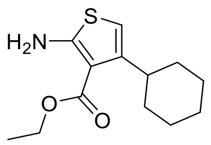

Ethyl cyanoacetate (470 µL, 4.42 mmol) and 1-cyclohexylethan-1-one (608 µL, 4.42 mmol) were added sequentially to a suspension of sulfur powder (1.13 g, 4.42 mmol) in morpholine (1 mL). The reaction mixture was stirred for 24 hours at room temperature. The solvent was then evaporated under reduced pressure and the crude was purified via flash column chromatography (SiO_2; 9:1 Hept/EtOAc). The obtained solid was then washed with heptane to afford 483 mg of desired product (43% yield). ^{1}H NMR (400 MHz, DMSO) δ 7.28 (s, 2H), 5.90 (s, 1H), 4.17 (q, *J* = 7.1 Hz, 2H), 2.94 (t, *J* = 11.5 Hz, 1H), 1.89 – 1.82 (m, 2H), 1.71 (dd, *J* = 24.7, 12.6 Hz, 3H), 1.29 – 1.24 (m, 5H), 1.23 – 1.08 (m, 3H).

ethyl 2-((tert-butoxycarbonyl)amino)thiophene-3-carboxylate ***S91***

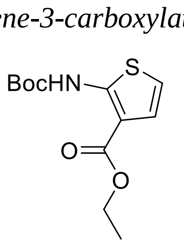

To a cooled solution (water-ice bath) of ethyl 2-aminothiophene-3-carboxylate (1 g, 5.8 mmol) in dioxane (10 mL) were added di-*tert*-butyl dicarbonate (1.59 g, 7.3 mmol) and DMAP (71 mg, 0.58 mmol). The reaction mixture was stirred at 80 °C for 2 hours. Upon completion (monitored by TLC), the mixture was concentrated under reduced pressure. The residue was then diluted with water and extracted with diethyl ether. The organic layers were combined and washed with water and brine, then dried over $MgSO_4$ and concentrated under reduced pressure. The crude product was purified via flash

column chromatography (SiO_2; from 5% to 8% EtOAc in Hept) providing 1.15 g of desired product (72% yield). ^{1}H NMR (400 MHz, $CDCl_3$) δ 10.08 (s, 1H), 7.16 (d, *J* = 5.8 Hz, 1H), 6.64 (d, *J* = 5.8 Hz, 1H), 4.32 (q, *J* = 7.1 Hz, 2H), 1.53 (s, 9H), 1.37 (t, *J* = 7.1 Hz, 3H). Procedure from Ref.[5]

*ethyl 5-bromo-2-((tert-butoxycarbonyl)amino)thiophene-3-carboxylate **39***

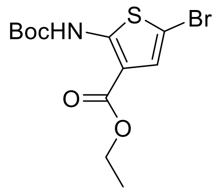

To a solution of **S91** (500 mg, 1.8 mmol) in DCM (2 mL) and acetic acid (2 mL) was added NBS (361 mg, 2 mmol, 1.1 eq.) at -15 °C (ice-salt bath). The reaction was stirred at this temperature for 1 hour, then it was diluted with water and extracted with diethyl ether. The organic layers were combined and washed with saturated $NaHCO_3$ solution (until gas evolution ceased) and brine. The organic layer was then dried with $MgSO_4$ and concentrated under reduced pressure, affording 571 mg of desired product which was used in the next step without further purification (88% yield). ^{1}H NMR (400 MHz, $CDCl_3$) δ 10.06 (s, 1H), 7.14 (s, 1H), 4.30 (q, *J* = 7.1 Hz, 2H), 1.52 (s, 9H), 1.36 (t, *J* = 7.1 Hz, 3H). Procedure from Ref.[3]

*ethyl 2-((tert-butoxycarbonyl)amino)-5-(4-methoxyphenyl)thiophene-3-carboxylate **40***

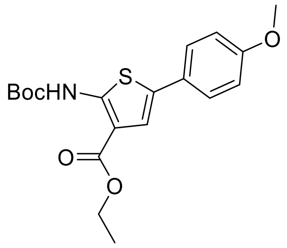

Compound **40** was prepared following General procedure C using **39** (135 mg, 0.37 mmol) and (4-methoxyphenyl)boronic acid (72 mg, 0.47 mmol). The reaction was stirred at 80 °C for 24 hours under N_2-atmosphere. The purification was performed via flash column chromatography (SiO_2; 5% EtOAc in Hept) providing 80 mg of desired product (60% yield). ^{1}H NMR (400 MHz, $CDCl_3$) δ 10.07 (s, 1H), 7.52 – 7.46 (m, 2H), 7.24 (s, 1H), 6.93 – 6.86 (m, 2H), 4.34 (q, *J* = 7.1 Hz, 2H), 3.83 (s, 3H), 1.55 (s, 9H), 1.39 (t, *J* = 7.1 Hz, 3H). LRMS (ESI) m/z: $[M – C_5H_8O_2]^{+\cdot}$ calcd for $C_{14}H_{15}NO_3S$; 277.077 found, 277.077.

*ethyl 2-amino-5-(4-methoxyphenyl)thiophene-3-carboxylate **37f***

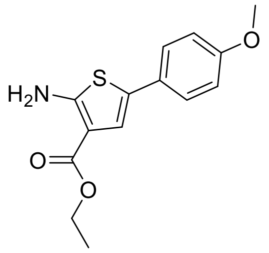

Compound **40** (50 mg, 0.13 mmol) was dissolved in EtOH (1 mL), followed by addition of 0.2 m4N HCl in dioxane (0.2 mL), and the reaction mixture was stirred at room temperature for 5 hours, and then heated at 50 °C overnight. Upon completion, the reaction was concentrated under reduced pressure and the crude residue was used in the following steps without further purification.

*ethyl 2-((tert-butoxycarbonyl)amino)-5-(4-(ethoxycarbonyl)phenyl)thiophene-3-carboxylate **41***

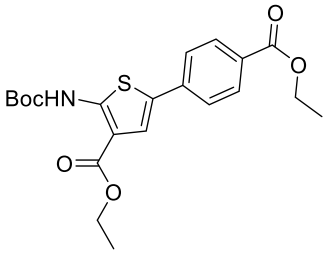

Compound **41** was prepared following General procedure C from **39** (200 mg, 0.57 mmol) and (4-(ethoxycarbonyl)phenyl)boronic acid (133 mg, 0.69 mmol). The reaction was stirred at 80 °C for 24 hours under N_2-atmosphere. The purification was performed via flash column chromatography (SiO_2; 5% EtOAc in Hept) providing 150 mg of desired product (62% yield). ^{1}H NMR (400 MHz, DMSO) δ 10.08 (s, 1H), 7.95 (d, *J* = 8.4 Hz, 2H), 7.78 (d, *J* = 8.4 Hz, 2H), 7.67 (s, 1H), 4.32 (q, *J* = 7.1 Hz, 4H), 1.52 (s, 9H), 1.33 (d, *J* = 7.1 Hz, 6H).

*ethyl 2-amino-5-(4-(ethoxycarbonyl)phenyl)thiophene-3-carboxylate **37g***

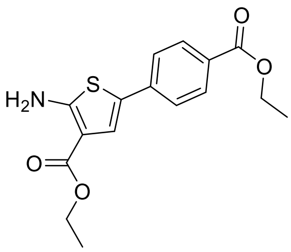

Compound **41** (133 mg, 0.32 mmol) was dissolved in EtOH (1 mL), followed by addition of 4N HCl in dioxane (0.3 mL), and the reaction mixture was stirred at 50 °C for 4 hours. Upon completion, the reaction was concentrated under reduced pressure and the crude product was used following steps without further purification. ^{1}H NMR (400 MHz, DMSO) δ 7.89 (d, *J* = 8.3 Hz, 2H), 7.57 (d, *J* = 8.3 Hz, 2H), 7.44 (s, 1H), 4.30 (q, *J* = 7.1 Hz, 2H), 4.22 (q, *J* = 7.1 Hz, 2H), 1.31 (t, *J* = 7.1 Hz, 3H), 1.29 (d, *J* = 7.1 Hz, 3H).

*5-phenyl-2-thioxo-2,3-dihydrothieno[2,3-d]pyrimidin-4(1H)-one **S74***

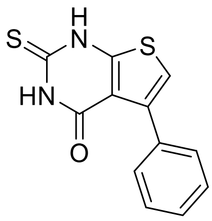

Benzoyl chloride (188 µL, 1.62 mmol) and ammonium thiocyanate (277 mg, 3.64 mmol) was added to MeCN and the reaction mixture was stirred at 80 °C for 30 min. Ethyl 2-amino-4-phenylthiophene-3-carboxylate (200 mg, 0.808 mmol) was added and the reaction mixture was stirred at 80 °C for additional 6 hours. After the reaction completion, the reaction mixture was cooled down to 0 °C and extracted into EtOAc. Dried over $MgSO_4$ and the solvent was removed *in vacuo*. The thiourea intermediate (35 mg, 0.85 mmol) was subsequently dissolved in EtOH (1.5 mL) and KOH (24 mg, 0.42 mmol) was added. The reaction mixture was heated at 80 °C for 14 hours. After the reaction completion, the reaction mixture was cooled down to room temperature and acidified with HCl 38%. The resulting precipitate was filtered off and the crude desired product was purified via flash column chromatography (SiO_2; Heptane/EtOAc 1:1) providing 7 mg of the desired product (33 % yield). ^{1}H NMR (400 MHz, DMSO) δ 13.67 – 13.22 (bs, 1H), 12.36 (s, 1H), 7.49 – 7.45 (m, 2H), 7.38 – 7.33 (m, 3H), 7.18 (s, 1H). ^{13}C NMR (101 MHz, DMSO) δ 173.3, 156.6, 153.7, 138.6, 134.5, 129.3, 127.7, 116.7, 115.1. LRMS (ESI) m/z: [M + H]+ calcd for $C_{12}H_9N_2OS_2$; 261.015 found, 261.015.

methyl 4-(4-oxo-2-thioxo-1,2,3,4-tetrahydrothieno[2,3-d]pyrimidin-5-yl)benzoate ***17***

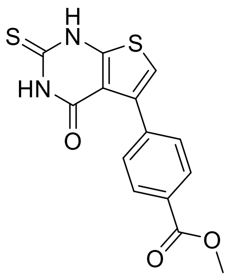

Compound **17** was prepared according to General procedure B using intermediate **37b** (230 mg, 0.75 mmol) and benzoyl isothiocyanate (184 mg, 1.13 mmol, 1.5 eq.). The cyclization was performed in EtOH (1.5 mL) and EtONa (102 mg, 1.5 mmol). After the filtration, the solid was purified via flash column chromatography (SiO_2; 10% MeOH in DCM), affording 80 mg of desired product (33 % yield after 2 steps). ^{1}H NMR (400 MHz, DMSO) δ 13.54 (s, 1H), 12.42 (s, 1H), 7.95 (d, J = 8.1 Hz, 2H), 7.64 (d, J = 8.0 Hz, 2H), 7.34 (s, 1H), 3.88 (s, 3H). ^{13}C NMR (101 MHz, DMSO) δ 173.3, 166.1, 165.8, 156.5, 153.6, 139.1, 137.3, 129.5, 128.5, 128.4, 118.1, 52.2. LRMS (ESI) m/z: [M + H]+ calcd for $C_{14}H_{11}N_2O_3S_2$; 319.021 found, 319.021.

4-(4-oxo-2-thioxo-1,2,3,4-tetrahydrothieno[2,3-d]pyrimidin-5-yl)benzoic acid ***18***

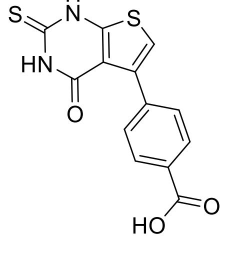

17 (50 mg, 0.16 mmol) was suspended in 10 % aqueous solution of NaOH (0.5 mL) and stirred at room temperature for 2 hours. Upon reaction completion (monitored by TLC), the mixture acidified to pH 3

by adding 38% HCl. The resulting precipitate was collected by filtration, washed thoroughly with water, and air-dried, providing 8 mg of desired product (17 % yield). ^{1}H NMR (400 MHz, DMSO) δ 13.54 (s, 1H), 12.96 (s, 1H), 12.42 (s, 1H), 7.93 (d, *J* = 7.9 Hz, 2H), 7.60 (d, *J* = 7.9 Hz, 2H), 7.31 (s, 1H). ^{13}C NMR (126 MHz, DMSO) δ 173.3, 167.2, 156.5, 153.6, 138.7, 137.5, 129.7, 129.4, 128.6, 117.9, 115.0. LRMS (ESI) m/z: [M + H]+ calcd for $C_{13}H_9N_2O_3S_2$; 305.004 found, 305.006.

*5-(4-bromophenyl)-2-thioxo-2,3-dihydrothieno[2,3-d]pyrimidin-4(1H)-one **S75***

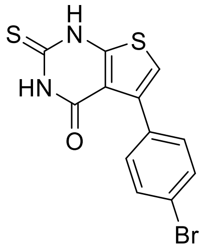

Compound **S75** was prepared according to General procedure B using the corresponding 2-aminothiophene derivative (110 mg, 0.338 mmol) and benzoyl isothiocyanate (58 mg, 0.355 mmol, 1.05 eq.). The cyclization was performed in EtOH (2 mL) and KOH (38 mg, 0.677 mmol, 2 eq.), providing 90 mg of desired compound (78 % yield over 2 steps). ^{1}H NMR (400 MHz, DMSO) δ 13.51 (s, 1H), 12.42 (s, 1H), 7.59 – 7.54 (m, 2H), 7.45 – 7.41 (m, 2H), 7.24 (s, 1H). ^{13}C NMR (101 MHz, DMSO) δ 173.2, 156.5, 153.5, 137.2, 133.6, 131.3, 130.5, 121.1, 117.2, 115.0. LRMS (ESI) m/z: [M + H]+ calcd for $C_{12}H_8N_2OS_2$; 338.926 found, 338.926.

*ethyl 2-(3-benzoylthioureido)-4-(4-methoxyphenyl)thiophene-3-carboxylate **S76***

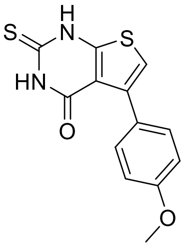

Compound **S76** was prepared according to General procedure B using the corresponding 2-aminothiophene derivative (200 mg, 0.721 mmol) and benzoyl isothiocyanate (123 mg, 0.757 mmol, 1.05 eq.). The cyclization was performed in EtOH/H_2O (1:1) and KOH (80 mg, 1.44 mmol, 2 eq.), providing 160 mg of derider product (76 % yield over 2 steps). ^{1}H NMR (400 MHz, DMSO) δ 13.47 (s, 1H), 12.35 (s, 1H), 7.44 – 7.38 (m, 2H), 7.09 (s, 1H), 6.96 – 6.90 (m, 2H), 3.78 (s, 3H). ^{13}C NMR (101 MHz, DMSO) δ 173.1, 158.9, 156.5, 153.3, 138.3, 130.4, 126.9, 115.5, 115.1, 113.0, 55.1. LRMS (ESI) m/z: [M + H]+ calcd for $C_{13}H_{11}N_2O_2S_2$; 291.026 found, 291.026.

5-(4-hydroxyphenyl)-2-thioxo-2,3-dihydrothieno[2,3-d]pyrimidin-4(1H)-one **19**

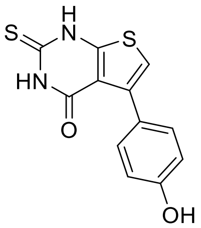

A solution of compound **S76** (30 mg, 0.1 mmol) in dry-DCM (5 mL) was cooled down to 0 °C (ice bath), followed by a dropwise addition of BBr_3 (1eq, 0.1 mL, 0.1 mmol). Ten minutes after the addition, the ice bath was removed and the reaction mixture was stirred overnight at room temperature. Upon completion the reaction mixture was quenched with water and extracted with EtOAc. Combined organic layers were dried over $MgSO_4$ and concentrated under reduced pressure. The crude product was purified using flash column chromatography (SiO_2; from 10% to 20% MeOH in DCM) providing 23 mg of desired product (79 % yield). ^{1}H NMR (400 MHz, DMSO) δ 13.46 (s, 1H), 12.33 (s, 1H), 9.50 (s, 1H), 7.33 – 7.25 (m, 2H), 7.03 (s, 1H), 6.78 – 6.70 (m, 2H). ^{13}C NMR (101 MHz, DMSO) δ 173.1, 157.1, 156.5, 153.3, 138.8, 130.4, 125.3, 115.1, 115.0, 114.4. LRMS (ESI) m/z: [M + H]+ calcd for $C_{12}H_9N_2O_2S_2$; 277.010 found, 277.009.

*5-cyclohexyl-2-thioxo-2,3-dihydrothieno[2,3-d]pyrimidin-4(1H)-one **S77***

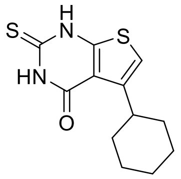

Compound **S80** was prepared according to General procedure B using intermediate **37e** (68 mg, 0.268 mmol) and benzoyl isothiocyanate (46 mg, 0.282 mmol). The cyclization was performed in EtOH and KOH (30 mg, 0.537 mmol, 2 eq.), providing 58 mg of desired product (81 % yield over 2 steps). ^{1}H NMR (400 MHz, DMSO) δ 13.44 (bs, 1H), 12.22 (s, 1H), 6.82 (s, 1H), 3.10 (tt, J = 11.3, 3.2 Hz, 1H), 1.96 – 1.65 (m, 5H), 1.40 – 1.11 (m, 5H). ^{13}C NMR (101 MHz, DMSO) δ 173.0, 157.1, 144.8, 115.9, 111.6, 37.6, 33.0, 26.2, 25.7. LRMS (ESI) m/z: [M + H]+ calcd for $C_{12}H_{15}N_2OS_2$; 267.062 found, 267.062.

*6-methyl-2-thioxo-2,3-dihydrothieno[2,3-d]pyrimidin-4(1H)-one **S78***

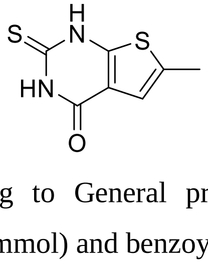

Compound **S78** was prepared according to General procedure B using the corresponding 2-aminothiophene derivative (95 mg, 0.558 mmol) and benzoyl isothiocyanate (91 mg, 0.558 mmol, 1.05 eq.). The cyclization was performed in MeOH and KOH (92 mg, 1.12 mmol, 2 eq.), providing 35 mg of desired product (33 % yield over 2 steps). ^{1}H NMR (400 MHz, DMSO) δ 13.33 (s, 1H), 12.37 (s, 1H),

6.92 (s, 1H), 2.41 (s, 3H). ^{13}C NMR (101 MHz, DMSO) δ 173.5, 156.8, 150.7, 133.6, 119.6, 119.0, 15.2. LRMS (ESI) m/z: [M + H]+ calcd for $C_7H_7N_2OS_2$; 198.999 found, 198.999.

*6-acetyl-5-methyl-2-thioxo-2,3-dihydrothieno[2,3-d]pyrimidin-4(1H)-one **20***

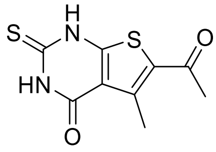

Compound **20** was prepared according to General procedure B using compound **37c** (100 mg, 0.440 mmol) and benzoyl isothiocyanate (79 mg, 0.484 mmol). The cyclization was performed in EtOH (1 mL) and EtONa (60 mg, 0.880 mmol), providing 89 mg of desired product (84 % yield over 2 steps). ^{1}H NMR (400 MHz, DMSO) δ 13.60 (s, 1H), 12.55 (s, 1H), 3.17 (s, 3H), 2.76 (s, 3H), 2.53 (s, 3H).^{13}C NMR (101 MHz, DMSO) δ 191.4, 174.3, 157.7, 154.6, 141.5, 130.6, 117.9, 30.1, 14.7. LRMS (ESI) m/z: [M + H]+ calcd for $C_9H_9N_2O_2S_2$; 241.010 found, 241.010.

*6-(1-hydroxyethyl)-5-methyl-2-thioxo-2,3-dihydrothieno[2,3-d]pyrimidin-4(1H)-one **S79***

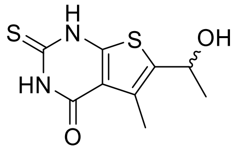

20 (50 mg, 0.2 mmol) was suspended in dry-MeOH (0.5 mL) under N_2-atmosphere. The reaction mixture was brought down to 0 °C and $NaBH_4$ (55 mg, 1.44 mmol) was added portion wise and the mixture was stirred for 2 hours. Upon completion (monitored by TLC), the reaction was quenched with saturated aq. solution of NH_4Cl. The reaction mixture was then extracted into EtOAc. The combined organic layers were dried over $MgSO_4$ and the solvent was removed *in vacuo*. The crude product was purified via flash column chromatography (SiO_2; from 6% to 8% MeOH in DCM) affording 3 mg of desired product (6 % yield). ^{1}H NMR (400 MHz, DMSO) δ 13.33 (bs, 1H), 12.24 (s, 1H), 5.74 (d, J = 3.7 Hz, 1H), 5.04 (qd, J = 6.3, 3.6 Hz, 1H), 2.31 (s, 3H), 1.33 (d, J = 6.3 Hz, 3H). ^{13}C NMR (126 MHz, DMSO) δ 172.9, 157.6, 150.7, 138.6, 126.3, 117.1, 62.3, 25.1, 12.8. LRMS (ESI) m/z: [M + H]+ calcd for $C_9H_{11}N_2O_2S_2$; 243.026 found, 243.026.

*ethyl 5-methyl-4-oxo-2-thioxo-1,2,3,4-tetrahydrothieno[2,3-d]pyrimidine-6-carboxylate **21***

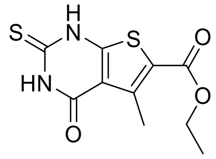

Compound **21** was prepared according to General procedure B using compound **37d** (850 mg, 3.30 mmol) and benzoyl isothiocyanate (593 mg, 3.63 mmol). The cyclization was performed in EtOH (12 mL) and EtONa (450 mg, 6.61 mmol), providing 700 mg of desired product (78 % yield over 2 steps). ^{1}H NMR (400 MHz, DMSO) δ 13.56 (s, 1H), 12.53 (s, 1H), 4.27 (q, J = 7.1 Hz, 2H), 2.71 (s, 3H), 1.29

(t, J = 7.0 Hz, 3H). ^{13}C NMR (101 MHz, DMSO) δ 174.3, 161.7, 157.3, 143.9, 117.8, 117.6, 61.1, 14.3, 14.2. LRMS (ESI) m/z: [M + H]+ calcd for $C_{10}H_{11}N_2O_3S_2$; 271.021 found, 271.020.

*5-methyl-4-oxo-2-thioxo-1,2,3,4-tetrahydrothieno[2,3-d]pyrimidine-6-carboxylic acid **S80***

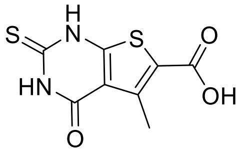

21 (614 mg, 2.3 mmol) was suspended in 10 % aqueous solution of NaOH (5 mL) and stirred at room temperature for 2 hours. Upon completion (monitored by TLC), the mixture acidified to pH 3 by adding 38% HCl. The resulting precipitate was collected by filtration, washed thoroughly with water, and air-dried affording 402 mg of desired product (73 % yield). ^{1}H NMR (500 MHz, DMSO) δ 13.62 (s, 1H), 13.38 (bs, 1H), 12.47 (s, 1H), 2.69 (s, 3H). ^{13}C NMR (126 MHz, DMSO) δ 174.1, 163.4, 157.4, 153.7, 142.8, 119.7, 117.7, 14.2. LRMS (ESI) m/z: [M + H]+ calcd for $C_8H_7N_2O_3S_2$; 242.989 found, 242.989.

N-benzyl-5-methyl-4-oxo-2-thioxo-1,2,3,4-tetrahydrothieno[2,3-d]pyrimidine-6-carboxamide **22**

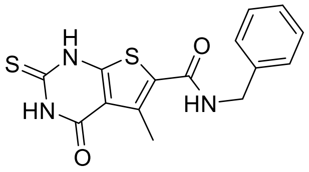

To a cooled solution (water-ice bath) of compound **S80** (30 mg, 0.124 mmol) in DMF (1 mL) was added DIPEA (64.7 µL, 0.37 mmol, 3 eq.), and the reaction mixture was stirred at the same temperature for 10 minutes. After the addition of 1.2 eq. of HATU (56.5 mg, 0.15 mmol), the solution was stirred for an additional 20 minutes after which 2 eq. of benzylamine (30 µL, 0.25 mmol) were added. The resulting reaction mixture was stirred at room temperature until completion (monitored by TLC), concentrated *in vacuo*, and purified using flash column chromatography (SiO_2; 4% MeOH in DCM) affording 17 mg of desired product (41 % yield). ^{1}H NMR (400 MHz, DMSO) δ 13.48 (s, 1H), 12.38 (s, 1H), 8.64 (t, J = 6.0 Hz, 1H), 7.37 – 7.28 (m, 4H), 7.28 – 7.21 (m, 1H), 4.42 (d, J = 5.9 Hz, 2H), 2.63 (s, 3H). ^{13}C NMR (126 MHz, DMSO) δ 173.9, 161.7, 157.5, 152.2, 139.3, 136.8, 128.3, 127.3, 126.9, 124.2, 117.4, 42.9, 14.5. LRMS (ESI) m/z: [M + H]+ calcd for $C_{15}H_{14}N_3O_2S_2$; 332.052 found, 332.051.

*5-methyl-4-oxo-N-(pyridin-3-ylmethyl)-2-thioxo-1,2,3,4-tetrahydrothieno[2,3-d]pyrimidine-6-carboxamide **23***

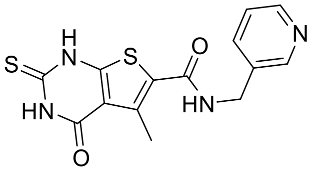

To a cooled solution (water-ice bath) of compound **S80** (20 mg, 0.085 mmol) in DMF (0.5 mL) was added DIPEA (43 µL, 0.25 mmol, 3 eq.), and the reaction mixture was stirred at the same temperature for 10 minutes. After the addition of 1.2 eq. of HATU (38 mg, 0.1 mmol), the solution was stirred for an additional 20 minutes after which 2 eq. of pyridin-3-ylmethanamine (17 µL, 0.17 mmol) were added. The resulting reaction mixture was stirred at room temperature until completion (monitored by TLC), concentrated *in vacuo*, and purified using flash column chromatography (SiO_2; 4% MeOH in DCM) affording 15 mg of desired product (54 % yield). ^{1}H NMR (400 MHz, DMSO) δ 13.47 (bs, 1H), 12.37 (s, 1H), 8.68 (t, J = 5.9 Hz, 1H), 8.54 (d, J = 2.3 Hz, 1H), 8.46 (dd, J = 4.8, 1.6 Hz, 1H), 7.72 (dt, J = 7.9, 2.0 Hz, 1H), 7.37 (dd, J = 7.9, 4.8 Hz, 1H), 4.44 (d, J = 5.9 Hz, 2H), 2.63 (s, 3H). ^{13}C NMR (101 MHz, DMSO) δ 161.9, 157.6, 148.9, 148.2, 137.2, 135.2, 134.8, 123.7, 123.5, 117.4, 40.7, 14.5. LRMS (ESI) m/z: [M + H]+ calcd for $C_{14}H_{13}N_4O_2S_2$; 333.047 found, 333.044.

tert-butyl 3-((5-methyl-4-oxo-2-thioxo-1,2,3,4-tetrahydrothieno[2,3-d]pyrimidine-6-carboxamido)methyl)benzoate ***S81***

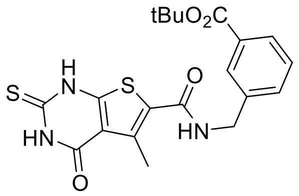

To a stirred solution of **S80** (20 mg, 0.08 mmol) in DMF (0.5 mL), were added sequentially TEA (350 µL, 0.25 mmol), EDC HCl (19 mg, 0.1 mmol) and HOBt hydrate (15 mg, 0.1 mmol.). Lastly, *tert*-butyl 3-(aminomethyl)benzoate (34 mg, 0.165 mmol) was added and the reaction was stirred at room temperature for 3 hours. The mixture was then concentrated under reduced pressure and purified using flash column chromatography (SiO_2; 5% MeOH in DCM) affording 15 mg of desired product (42% yield). ^{1}H NMR (500 MHz, DMSO) δ 13.50 (s, 1H), 12.47 (s, 1H), 8.74 (t, J = 5.9 Hz, 1H), 7.85 (s, 1H), 7.78 (d, J = 7.7 Hz, 1H), 7.56 (d, J = 7.7 Hz, 1H), 7.46 (t, J = 7.7 Hz, 1H), 4.47 (d, J = 5.9 Hz, 2H), 2.64 (s, 3H), 1.53 (s, 9H). ^{13}C NMR (126 MHz, DMSO) δ 173.9, 165.0, 161.8, 157.5, 152.2, 139.9, 136.9, 131.9, 131.4, 128.7, 127.7, 127.6, 124.1, 117.5, 80.8, 42.7, 27.8, 14.5. LRMS (ESI) m/z: [M + H – C_4H_7]+ calcd for $C_{16}H_{14}N_3O_4S_2$; 376.042 found, 376.042.

3-((5-methyl-4-oxo-2-thioxo-1,2,3,4-tetrahydrothieno[2,3-d]pyrimidine-6-carboxamido)methyl)benzoic acid ***24***

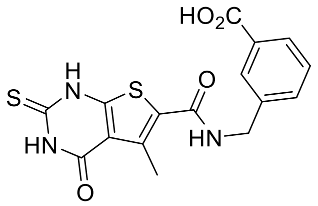

To a stirred solution of **S80** (11 mg, 0.025 mmol) in DCM (0.4 mL) was added TFA (20 µL, 0.25 mmol), and the resulting reaction mixture was stirred at room temperature until full completion (monitored by

TLC). The volatiles were then removed *in vacuo* and the solid was dried at the high-vacuum pump for 24 hours, affording 9 mg of desired compound (99 %). ^{1}H NMR (400 MHz, DMSO) δ 13.49 (s, 1H), 12.47 (s, 1H), 8.74 (t, *J* = 6.0 Hz, 1H), 7.90 (s, 1H), 7.82 (d, *J* = 7.6 Hz, 1H), 7.56 (d, *J* = 7.5 Hz, 1H), 7.46 (t, *J* = 7.6 Hz, 1H), 4.47 (d, *J* = 6.0 Hz, 2H), 2.63 (s, 3H). ^{13}C NMR (101 MHz, DMSO) δ 173.9, 167.3, 161.8, 157.5, 152.2, 139.8, 137.0, 131.9, 130.9, 128.7,128.1, 127.9, 124.1, 117.5, 42.7, 14.5. LRMS (ESI) m/z: [M + H]+ calcd for $C_{16}H_{14}N_3O_4S_2$; 376.042 found, 376.042.

tert-butyl 4-(5-methyl-4-oxo-2-thioxo-1,2,3,4-tetrahydrothieno[2,3-d]pyrimidine-6-carboxamido)butanoate ***S82***

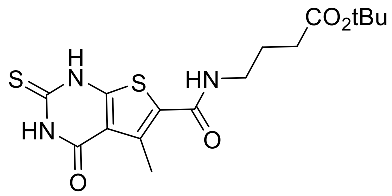

To a stirred solution of **S80** (20 mg, 0.08 mmol) in DMF (0.5 mL), were added sequentially TEA (350 µL, 0.25 mmol), EDC HCl (19 mg, 0.1 mmol) and HOBt hydrate (15 mg, 0.1 mmol). Lastly, *tert*-butyl 4-aminobutanoate (26 mg, 0.165 mmol) was added and the reaction was stirred at room temperature for 3 hours. The mixture was concentrated under reduced pressure and purified using flash column chromatography (SiO_2; 5% MeOH in DCM) affording 21 mg of desired product (66 % yield). ^{1}H NMR (500 MHz, DMSO) δ 13.47 (bs, 1H), 12.41 (s, 1H), 8.10 (t, *J* = 5.7 Hz, 1H), 3.25 – 3.17 (m, 2H), 2.59 (s, 3H), 2.23 (t, *J* = 7.4 Hz, 2H), 1.71 (p, *J* = 7.3 Hz, 2H), 1.39 (s, 9H). ^{13}C NMR (126 MHz, DMSO) δ 173.9, 172.1, 172.0, 161.7, 157.6, 136.3, 124.5, 117.4, 79.7, 38.8, 32.3, 27.8, 24.6, 14.5. LRMS (ESI) m/z: [M – C_4H_7]+ calcd for $C_{16}H_{22}N_3O_4S_2$; 328.042 found, 328.042.

4-(5-methyl-4-oxo-2-thioxo-1,2,3,4-tetrahydrothieno[2,3-d]pyrimidine-6-carboxamido)butanoic acid ***25***

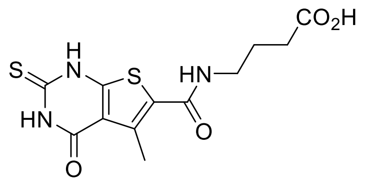

To a stirred solution of **S80** (15 mg, 0.039 mmol) in DCM (0.5 mL) was added TFA (30 µL, 0.39 mmol, 10 eq.), and the resulting reaction mixture was stirred at room temperature until full completion (monitored by TLC). The volatiles were then removed *in vacuo* and the solid was dried at the high-vacuum pump for 24 hours, affording 12 mg of desired compound (99 %). ^{1}H NMR (400 MHz, DMSO) δ 13.48 (s, 1H), 12.46 (s, 1H), 8.12 (t, *J* = 5.8 Hz, 1H), 3.22 (q, *J* = 6.5 Hz, 2H), 2.59 (s, 3H), 2.25 (t, *J* = 7.3 Hz, 2H), 1.72 (p, *J* = 7.0 Hz, 2H). ^{13}C NMR (126 MHz, DMSO) δ 174.2, 174.2, 173.8, 161.6, 157.5, 136.2, 124.6, 117.3, 31.1, 27.8, 24.4, 14.4. LRMS (ESI) m/z: [M + H]+ calcd for $C_{16}H_{22}N_3O_4S_2$; 328.042 found, 328.042.

*N-(4-hydroxybutyl)-5-methyl-4-oxo-2-thioxo-1,2,3,4-tetrahydrothieno[2,3-d]pyrimidine-6-carboxamide **S83***

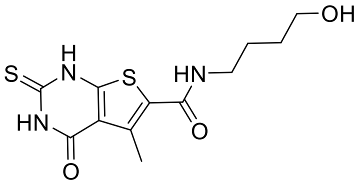

To a stirred solution of **S80** (20 mg, 0.08 mmol) in DMF (0.5 mL), were added sequentially TEA (350 µL, 0.25 mmol), EDC HCl (19 mg, 0.1 mmol) and HOBt hydrate (15 mg, 0.1 mmol). Lastly, 4-aminobutan-1-ol (15 mg, 0.165 mmol) was added and the reaction was stirred at room temperature. After 3 hours the reaction mixture was concentrated under reduced pressure and purified using flash column chromatography (SiO_2; 5% MeOH in DCM) affording 9 mg of desired product (35 % yield). ^{1}H NMR (400 MHz, DMSO) δ 13.47 (bs, 1H), 12.43 (s, 1H), 8.09 (t, *J* = 5.7 Hz, 1H), 4.45 (s, 1H), 3.20 (q, *J* = 6.6 Hz, 2H), 2.59 (s, 3H), 1.58 – 1.39 (m, 4H). ^{13}C NMR (101 MHz, DMSO) δ 173.9, 161.5, 157.6, 152.2, 136.0, 124.9, 117.4, 60.5, 37.1, 30.0, 25.8, 14.5. LRMS (ESI) m/z: [M + H]+ calcd for $C_{12}H_{16}N_3O_3S_2$; 314.062 found, 314.063.

*6-phenyl-2-thioxo-2,3-dihydrothieno[2,3-d]pyrimidin-4(1H)-one **S84***

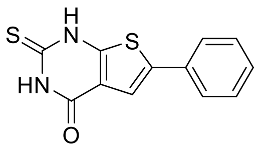

Compound **S84** was prepared according to General procedure B using the corresponding 2-aminothiophene derivative (98 mg, 0.396 mmol) and benzoyl isothiocyanate (68 mg, 0.416 mmol, 1.05 eq.). The cyclization was performed in EtOH (2 mL) and KOH (44 mg, 0.794 mmol, 2 eq.), providing 79 mg of desired product (76 % yield over 2 steps). ^{1}H NMR (400 MHz, DMSO) δ 13.62 (s, 1H), 12.50 (s, 1H), 7.70 (d, *J* = 7.7 Hz, 2H), 7.64 (s, 1H), 7.43 (t, *J* = 7.5 Hz, 2H), 7.35 (t, *J* = 7.3 Hz, 1H). ^{13}C NMR (101 MHz, DMSO) δ 173.4, 156.5, 135.9, 132.3, 129.3, 128.3, 125.4, 119.7, 117.5. LRMS (ESI) m/z: [M + H]+ calcd for $C_{12}H_9N_2OS_2$; 216.015 found, 216.015.

*5-methyl-6-(4-nitrophenyl)-2-thioxo-2,3-dihydrothieno[2,3-d]pyrimidin-4(1H)-one **26***

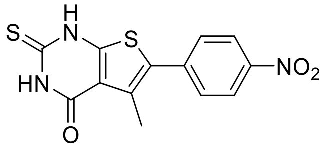

Compound **26** was prepared according to General procedure B using the corresponding 2-aminothiophene derivative (100 mg, 0.326 mmol) and benzoyl isothiocyanate (58 mg, 0.359 mmol). The cyclization was performed in EtOH (1 mL) and EtONa (44 mg, 0.653 mmol), affording 107 mg of desired compound (99 %). ^{1}H NMR (400 MHz, DMSO) δ 13.54 (bs, 1H), 12.45 (s, 1H), 8.36 – 8.26 (m, 2H), 7.83 – 7.71 (m, 2H), 2.52 (s, 3H). ^{13}C NMR (126 MHz, DMSO) δ 173.4, 157.4, 152.0, 146.5, 139.0,

132.4, 129.9, 126.5, 124.2, 118.0, 14.2. LRMS (ESI) m/z: [M + H]+ calcd for $C_{13}H_{10}N_3O_3S_2$; 320.015 found, 320.016.

6-(4-aminophenyl)-5-methyl-2-thioxo-2,3-dihydrothieno[2,3-d]pyrimidin-4(1H)-one ***27***

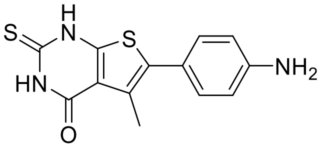

To 0.4 mL of a 1:1 EtOH/H_2O solution, NH_4Cl (33 mg, 0.626 mmol) and compound **26** (40 mg, 0.125 mmol) were added sequentially. Iron powder (35 mg, 0.626 mmol) was added and the mixture was stirred for 2 hours at 80 °C. Upon completion (monitored by TLC), the reaction was extracted with EtOAc 3 times, dried with $MgSO_4$ and concentrated under reduced pressure. The crude product was purified via flash column chromatography (SiO_2; from 10% to 20% MeOH in DCM) providing 12 mg of desired product (33 % yield). ^{1}H NMR (400 MHz, DMSO) δ 13.25 (bs, 1H), 12.29 (s, 1H), 7.17 – 7.07 (m, 2H), 6.67 – 6.57 (m, 2H), 5.42 (bs, 2H), 2.38 (s, 3H). ^{13}C NMR (126 MHz, DMSO) δ 172.7, 157.5, 149.8, 149.0, 130.8, 129.9, 126.8, 118.8, 117.9, 113.9, 14.0. LRMS (ESI) m/z: [M + H]+ calcd for $C_{13}H_{12}N_3OS_2$; 290.041 found, 290.042.

6-(4-methoxyphenyl)-2-thioxo-2,3-dihydrothieno[2,3-d]pyrimidin-4(1H)-one ***28***

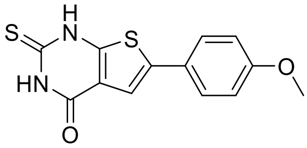

Compound **28** was prepared according to General procedure B using compound **37f** (37 mg, 0.13 mmol) and benzoyl isothiocyanate (24 mg, 0.15 mmol). The cyclization was performed in EtOH (1 mL) and EtONa (18 mg, 0.27 mmol). The yield was 14 mg (36 % yield after two steps). ^{1}H NMR (400 MHz, DMSO) δ 13.51 (s, 1H), 12.49 (s, 1H), 7.68 – 7.59 (m, 2H), 7.50 (s, 1H), 7.03 – 6.94 (m, 2H), 3.79 (s, 3H). ^{13}C NMR (101 MHz, DMSO) δ 173.1, 159.4, 156.5, 150.0, 136.1, 126.9, 124.8, 119.8, 116.0, 114.7, 55.3.LRMS (ESI) m/z: [M + H]+ calcd for $C_{13}H_{11}N_2O_2S_2$; 291.025 found, 291.026.

ethyl 4-(4-oxo-2-thioxo-1,2,3,4-tetrahydrothieno[2,3-d]pyrimidin-6-yl)benzoate ***29***

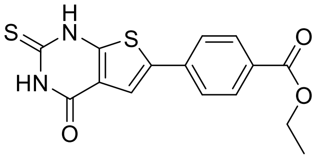

Compound **29** was prepared according to General procedure B using compound **37g** (100 mg, 0.31 mmol) and benzoyl isothiocyanate (56 mg, 0.34 mmol). The cyclization was performed in EtOH (2 mL) and EtONa (42 mg, 0.63 mmol), affording 65 mg of desired product (62% yield after two steps). ^{1}H NMR (400 MHz, DMSO) δ 13.61 (s, 1H), 12.58 (s, 1H), 8.01 – 7.92 (m, 2H), 7.90 – 7.83 (m, 2H), 7.84 (s, 1H), 4.32 (q, J = 7.1 Hz, 2H), 1.33 (t, J = 7.1 Hz, 3H). ^{13}C NMR (101 MHz, DMSO) δ 173.6, 165.2,

156.4, 151.5, 136.7, 134.3, 130.0, 129.0, 125.4, 119.9, 119.8, 60.8, 14.2. LRMS (ESI) m/z: [M + H]+ calcd for $C_{15}H_{13}N_2O_3S_2$; 333.036 found, 333.037.

*4-(4-oxo-2-thioxo-1,2,3,4-tetrahydrothieno[2,3-d]pyrimidin-6-yl)benzoic acid **30***

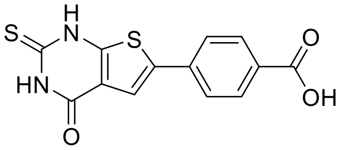

29 (30 mg, 0.1 mmol) were suspended in 10% aq. solution of NaOH (1 mL), and stirred at room temperature for 4 hours. Upon completion (monitored by TLC), the mixture was concentrated under reduced pressure and the resulting solid was suspended in 1M HCl (0.5 mL) and stirred for 1 hour. The solid was collected by filtration, washed thoroughly with water, and air-dried affording 17 mg of desired compound (60 % yield) ^{1}H NMR (400 MHz, DMSO) δ 13.60 (s, 1H), 13.02 (s, 1H), 12.56 (s, 1H), 8.00 – 7.89 (m, 2H), 7.87 – 7.80 (m, 2H), 7.82 (s, 1H). ^{13}C NMR (101 MHz, DMSO) δ 173.6, 166.8, 156.4, 151.5, 136.4, 134.5, 130.2, 130.0, 125.3, 119.9, 119.5. LRMS (ESI) m/z: [M + H]+ calcd for $C_{13}H_9N_2O_3S_2$; 305.004 found, 305.006.

*6-benzyl-2-thioxo-2,3-dihydrothieno[2,3-d]pyrimidin-4(1H)-one **S85***

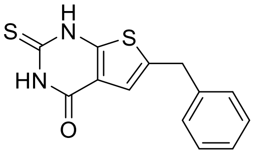

Compound **S85** was prepared according to General procedure B using the corresponding 2-aminothiophene derivative (71 mg, 0.271 mmol) and benzoyl isothiocyanate (47 mg, 0.285 mmol, 1.05 eq.). The cyclization was performed in EtOH (1.5 mL) and KOH (30 mg, 0.794 mmol, 2 eq.), providing 62 mg of desired product (83 % yield over 2 steps). ^{1}H NMR (400 MHz, DMSO) δ 13.44 (s, 1H), 12.36 (s, 1H), 7.36 – 7.21 (m, 5H), 6.98 (d, *J* = 1.0 Hz, 1H), 4.11 (s, 2H). ^{13}C NMR (101 MHz, DMSO) δ 173.1, 156.5, 150.9, 139.5, 138.0, 128.7, 128.6, 126.7, 118.9, 118.2, 35.0. LRMS (ESI) m/z: [M + H]+ calcd for $C_{13}H_{11}N_2OS_2$; 275.030 found, 275.031.

*ethyl 4-((4-oxo-2-thioxo-1,2,3,4-tetrahydrothieno[2,3-d]pyrimidin-6-yl)methyl)benzoate **S86***

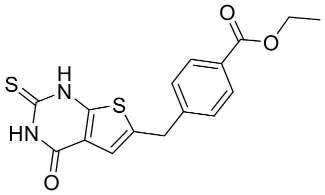

Compound **S86** was prepared according to General procedure B using the corresponding 2-aminothiophene derivative (340 mg, 0.685 mmol) and benzoyl isothiocyanate (58 mg, 0.359 mmol). The cyclization was performed in EtOH (1 mL) and EtONa (93 mg, 1.37 mmol), providing 218 mg of desired product (48 % yield over 2 steps). ^{1}H NMR (500 MHz, DMSO) δ 13.37 (s, 1H), 12.40 (s, 1H),

7.92 (d, J = 8.0 Hz, 2H), 7.44 (d, J = 8.0 Hz, 2H), 7.03 (s, 1H), 4.30 (q, J = 7.1 Hz, 2H), 4.21 (s, 2H), 1.31 (t, J = 7.1 Hz, 3H). ^{13}C NMR (126 MHz, DMSO) δ 173.2, 165.5, 156.5, 151.0, 145.1, 136.8, 129.6, 129.0, 128.4, 119.6, 118.3, 60.7, 34.7, 18.6, 14.2. LRMS (ESI) m/z: [M + H]+ calcd for $C_{16}H_{15}N_2O_3S_2$; 347.052 found, 347.052.

4-((4-oxo-2-thioxo-1,2,3,4-tetrahydrothieno[2,3-d]pyrimidin-6-yl)methyl)benzoic acid ***S87***

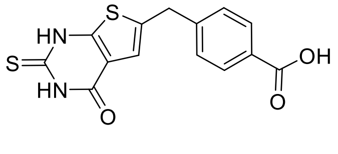

Compound **S86** (40 mg, 0.115 mmol) was suspended in a mixture of dioxane/water (1:1) (0.5 mL), and 38% HCl (0.25 mL) was added. The reaction mixture was refluxed for 24 hours. The precipitate was filtered off, washed with water and dried on air, providing 25 mg of the carboxylic acid derivative (68 % yield). ^{1}H NMR (500 MHz, DMSO) δ 13.45 – 13.21 (bs, 1H), 13.01 – 12.69 (bs, 1H), 12.38 (s, 1H), 7.90 (d, J = 8.0 Hz, 2H), 7.41 (d, J = 8.0 Hz, 2H), 7.03 (s, 1H), 4.20 (s, 2H). ^{13}C NMR (126 MHz, DMSO) δ 173.3, 167.1, 156.5, 151.2, 144.7, 136.8, 129.7, 129.7, 129.3, 128.8, 128.7, 119.5, 118.2, 34.7. LRMS (ESI) m/z: [M + H]+ calcd for $C_{14}H_{11}N_2O_3S_2$; 319.021 found, 319.020.

5-methyl-2-thioxo-2,3-dihydrothieno[2,3-d]pyrimidin-4(1H)-one ***S88***

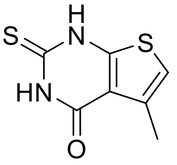

Benzoyl chloride (678 µL, 5.84 mmol) and ammonium thiocyanate (666 mg, 8.76 mmol) was added to MeCN and the reaction mixture was stirred at 80 °C for 30 min. Methyl 2-amino-4-methylthiophene-3-carboxylate (500 mg, 2.92 mmol) was added and the reaction mixture was stirred at 80 °C for additional 6 hours. After the reaction completion, the reaction mixture was cooled down to 0 °C and extracted into EtOAc. Dried over $MgSO_4$ and the solvent was removed *in vacuo*. The thiourea intermediate (436 mg, 1.3 mmol) was subsequently dissolved in EtOH (5 mL) and KOH (417 mg, 7.44 mmol) was added. The reaction mixture was heated at 80 °C for 14 hours. After the reaction completion, the reaction mixture was cooled down to room temperature and acidified with HCl 38%. The resulting precipitate was filtered off and the crude desired product was purified via flash column chromatography (SiO_2; Heptane/EtOAc 1:1) providing 190 mg of the desired product (33 % yield after two steps). ^{1}H NMR (500 MHz, DMSO) δ 13.36 (s,1H), 12.32 (s,1H), 6.85 (s,1H), 2.35(s,3H). ^{13}C NMR (126 MHz, DMSO) δ 173.4, 157.4, 152.4, 133.8, 116.8, 114.1, 15.5. LRMS (ESI) m/z: [M + H]+ calcd for $C_7H_7N_2OS_2$; 198.999 found, 198.999.

ethyl 4-oxo-2-thioxo-1,2,3,4-tetrahydrothieno[2,3-d]pyrimidine-5-carboxylate ***S89***

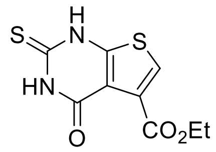

Compound **S89** was prepared according to General procedure B using the corresponding 2-aminothiophene derivative (500 mg, 2.06 mmol) and benzoyl isothiocyanate (370 mg, 2.26 mmol). The cyclization was performed in EtOH (5 mL) and EtONa (0.28 g, 4.11 mmol), affording 274 mg of desired compound (52% yield over 2 steps). ^{1}H NMR (400 MHz, DMSO) δ 13.54 (s, 1H), 12.45 (s, 1H), 7.71 (s, 1H), 4.24 (q, *J* = 7.1 Hz, 2H), 1.27 (t, *J* = 7.1 Hz, 3H). ^{13}C NMR (101 MHz, DMSO) δ 173.7, 162.4, 155.2, 153.0, 129.2, 123.9, 115.3, 61.0, 14.0. LRMS (ESI) m/z: [M + H]+ calcd for $C_{18}H_{18}N_2O_5S_2$; 257.004 found, 257.004.

4-oxo-2-thioxo-1,2,3,4-tetrahydrothieno[2,3-d]pyrimidine-5-carboxylic acid ***S90***

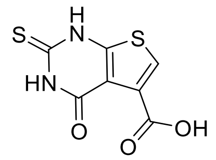

50 mg of **S89** (0.2 mmol) were suspended in 1 mL of 10% NaOH solution and stirred at room temperature for 2 hours. Upon completion (monitored by TLC), the mixture acidified to pH 3 by adding 38% HCl. The resulting precipitate was collected by filtration, washed thoroughly with water, and air-dried, affording 25 mg of desired product (56% yield). ^{1}H NMR (400 MHz, DMSO) δ 14.62 (s, 1H), 13.30 (s, 1H), 8.08 (s, 1H). ^{13}C NMR (101 MHz, DMSO) δ 173.0, 160.7, 160.3, 129.9, 128.9, 113.5. LRMS (ESI) m/z: [M + H]+ calcd for $C_7H_5N_2O_3S_2$; 228.973 found, 228.974.

4-oxo-2-thioxo-1,2,3,4-tetrahydrothieno[2,3-d]pyrimidine-5-carboxamide ***31***

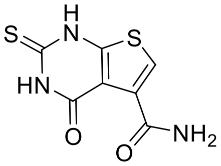

50 mg of **S89** (0.2 mmol) were suspended in 0.5 mL of ammonia 7N solution in MeOH, upon heating the starting material was dissolved. The mixture was stirred for 4 hours at reflux, during which a white precipitate appeared. Upon completion (monitored by TLC), the reaction was cooled down to room temperature, and the solvent was evaporated under reduced pressure. The residue was stirred for 1 hour in 0.5 mL of 1 M aqueous HCl, then the solid was collected by filtration, washed thoroughly with water, and air-dried affording 40 mg of desired product (88 % yield). ^{1}H NMR (400 MHz, DMSO) δ 13.76 (s, 1H), 12.84 (s, 1H), 9.72 – 9.67 (m, 1H), 7.94 (s, 1H), 7.64 (s, 1H). ^{13}C NMR (126 MHz, DMSO) δ 172.8, 161.3, 158.8, 155.0, 132.9, 127.1, 113.5. LRMS (ESI) m/z: [M + H]+ calcd for $C_7H_6N_3O_2S_2$; 227.989 found, 228.974.

REFERENCES

(1) Wiedmer, L.; Eberle, S. A.; Bedi, R. K.; Śledź, P.; Caflisch, A. A Reader-Based Assay for M6A Writers and Erasers. *Anal. Chem.* **2019**, 91 (4), 3078–3084. DOI: 10.1021/acs.analchem.8b05500.

(2) Wang, T.; Zheng, C. H.; Liu, S.; Chen, H. Z. Synthesis and Biological Activity of a Series of New Thieno[2,3-d]Pyrimidines. *Phosphorus Sulfur Silicon Relat. Elem.* **2010**, 185 (7), 1543–1549. DOI: 10.1080/10426500903127565.

(3) Aurelio, L.; Figler, H.; Flynn, B. L.; Linden, J.; Scammells, P. J. 5-Substituted 2-Aminothiophenes as A1 Adenosine Receptor Allosteric Enhancers. *Bioorg. Med. Chem.* **2008**, 16 (3), 1319-1327. DOI: 10.1016/j.bmc.2007.10.065.

(4) Gangjee, A.; Qiu, Y.; Kisliuk, R. L. Synthesis of Classical and Nonclassical 2-Amino-4-Oxo-6-Benzylthieno-[2,3-d]Pyrimidines as Potential Thymidylate Synthase Inhibitors. *J. Heterocycl. Chem.* **2004**, 41 (6), 941–946. DOI: 10.1002/jhet.5570410613.

(5) Dong, Y.; Navarathne, D.; Bolduc, A.; McGregor, N.; Skene, W. G. α,A′-N-Boc-Substituted Bi- and Terthiophenes: Fluorescent Precursors for Functional Materials. *J. Org. Chem.* **2012**, 77 (12), 5429–5433. DOI: 10.1021/jo300687d.

^{1}H AND ^{13}C NMR SPECTRA OF FINAL COMPOUNDS

5-phenyl-2-thioxo-2,3-dihydrothieno[2,3-d]pyrimidin-4(1H)-one S74

^{1}H-NMR

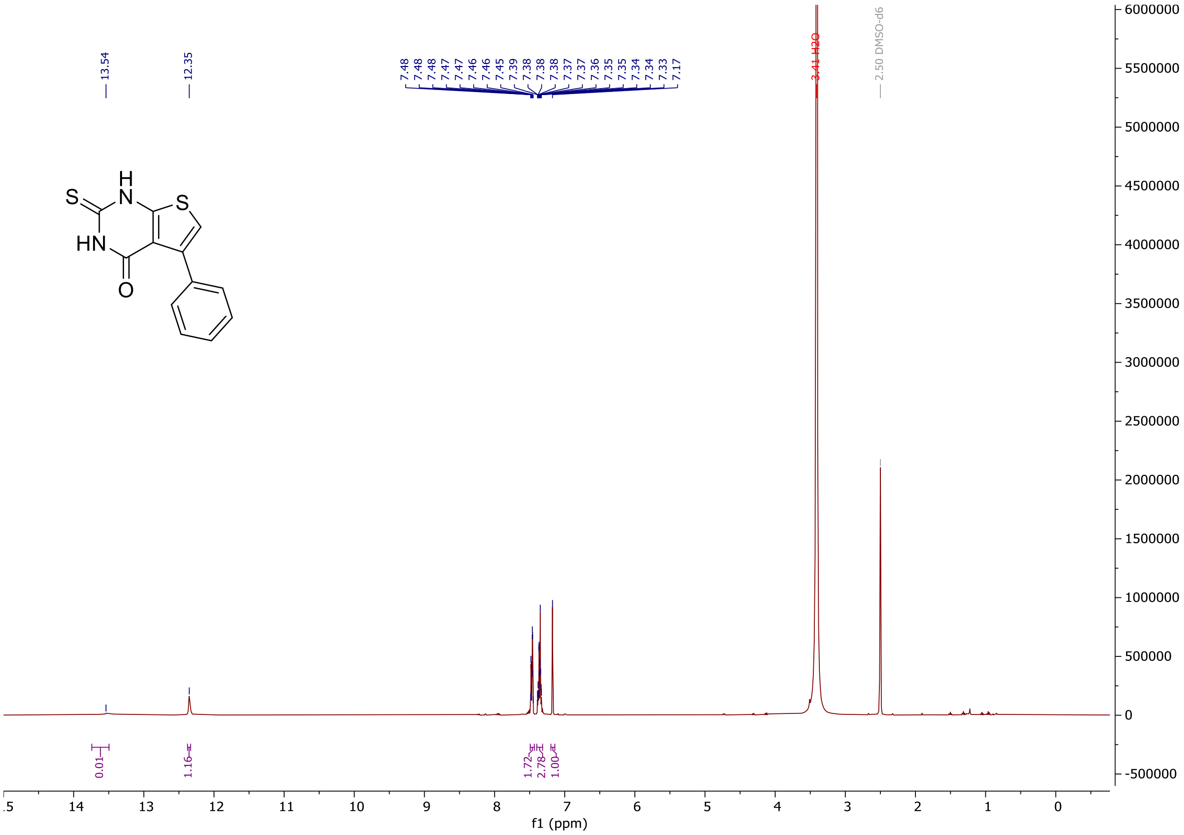

^{13}C-NMR

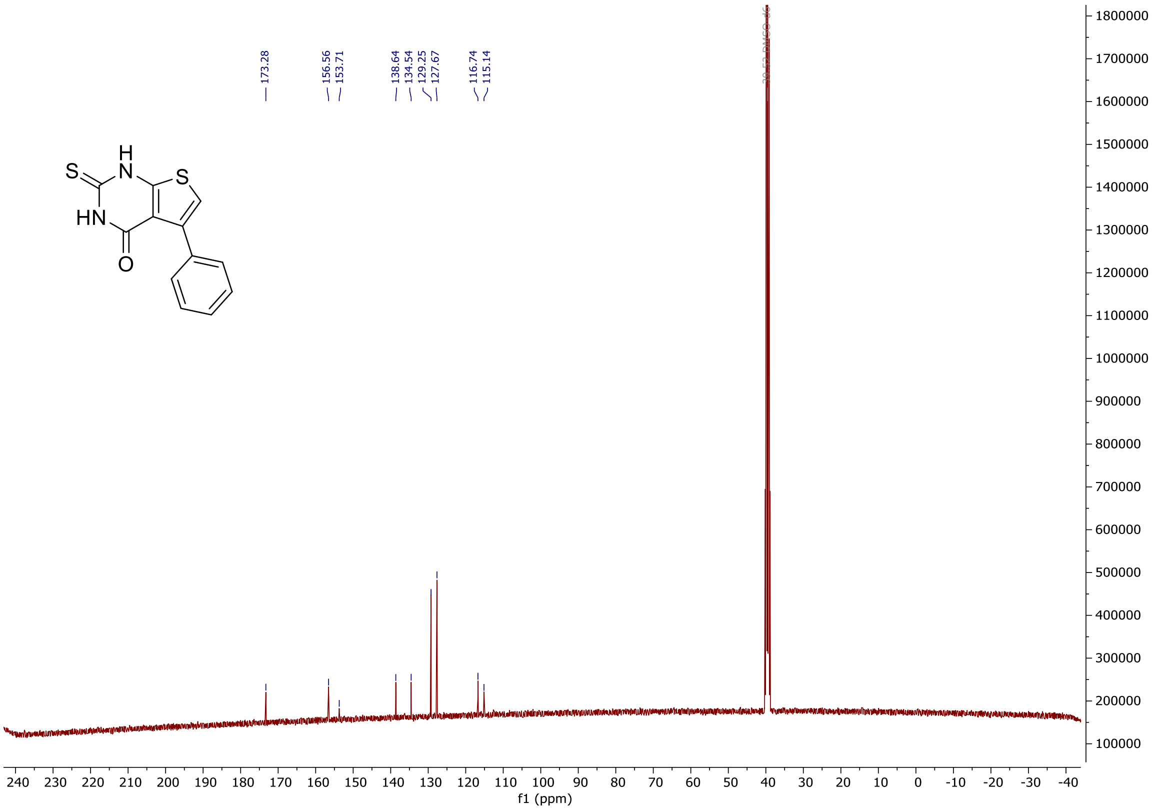

methyl 4-(4-oxo-2-thioxo-1,2,3,4-tetrahydrothieno[2,3-d]pyrimidin-5-yl)benzoate 17

^{1}H-NMR

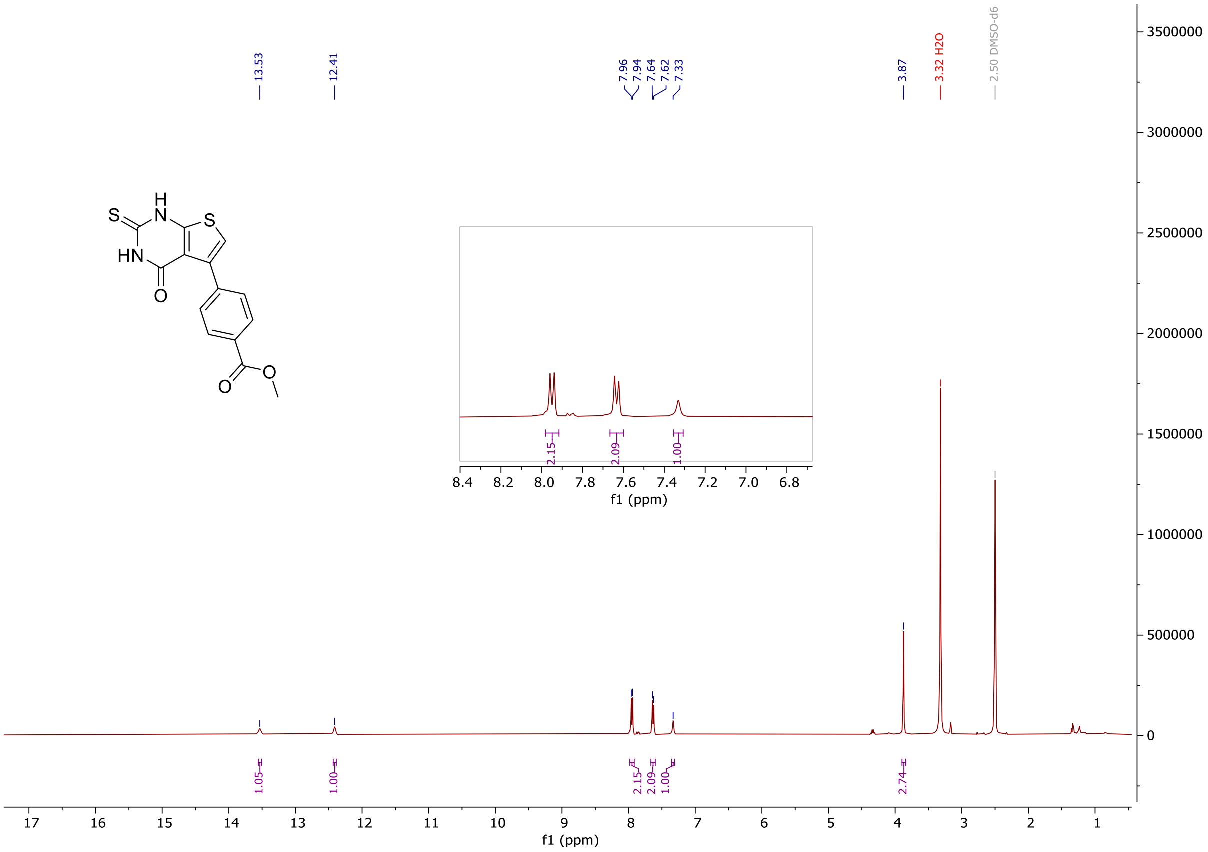

^{13}C-NMR

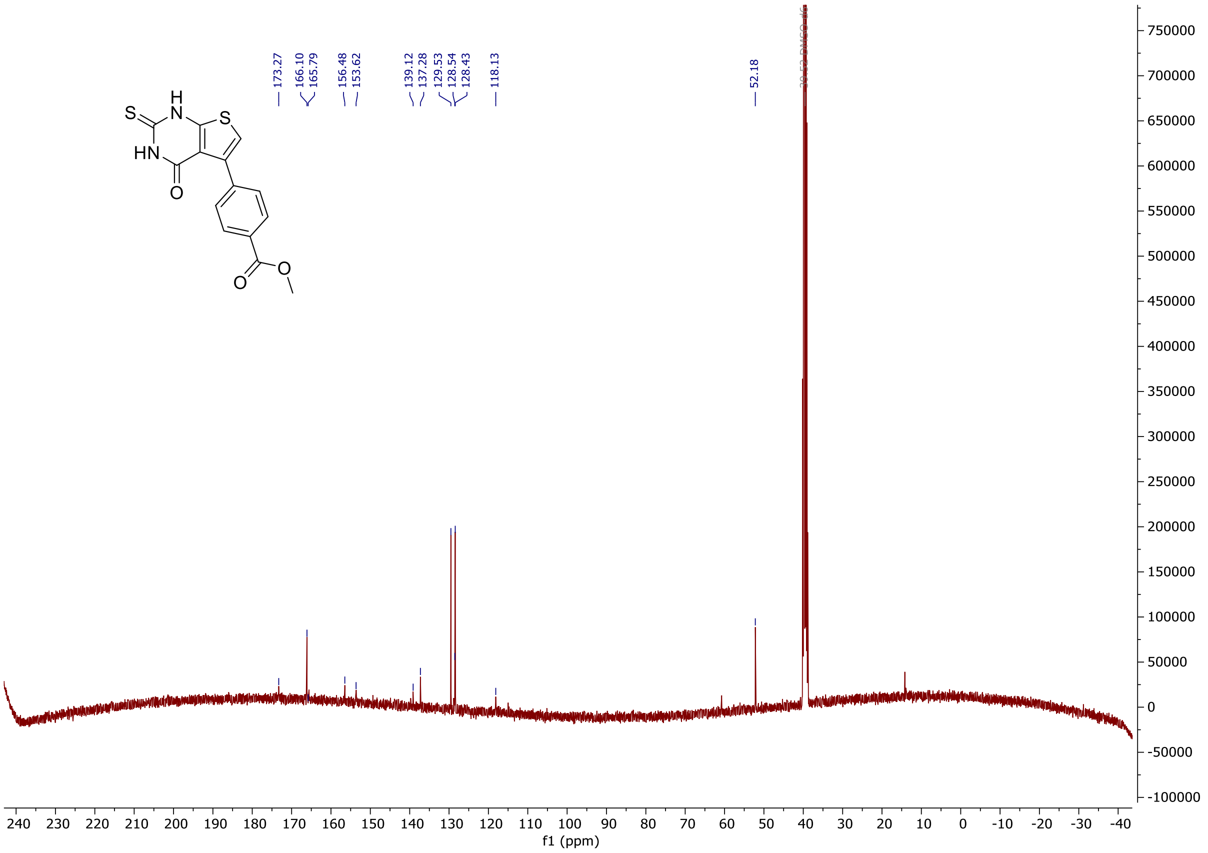

4-(4-oxo-2-thioxo-1,2,3,4-tetrahydrothieno[2,3-d]pyrimidin-5-yl)benzoic acid 18

^{1}H-NMR

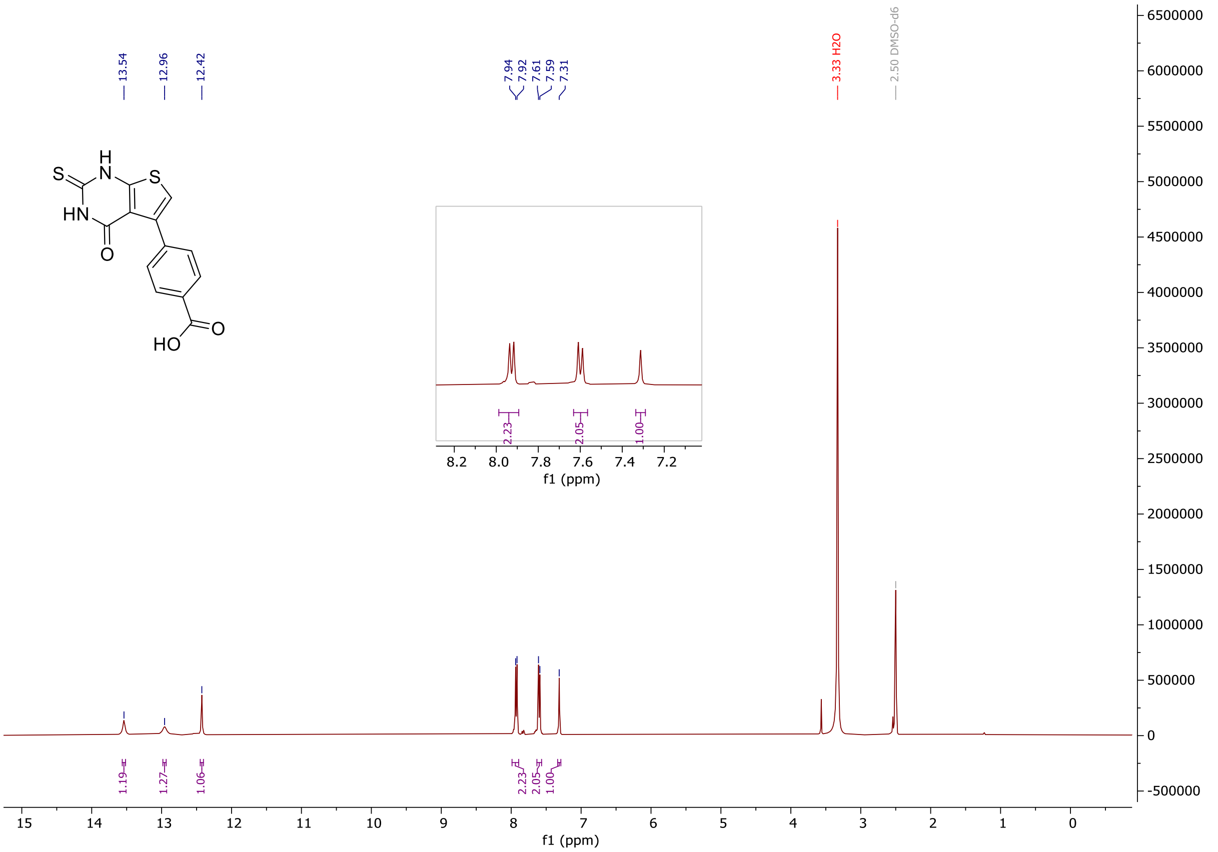

^{13}C-NMR

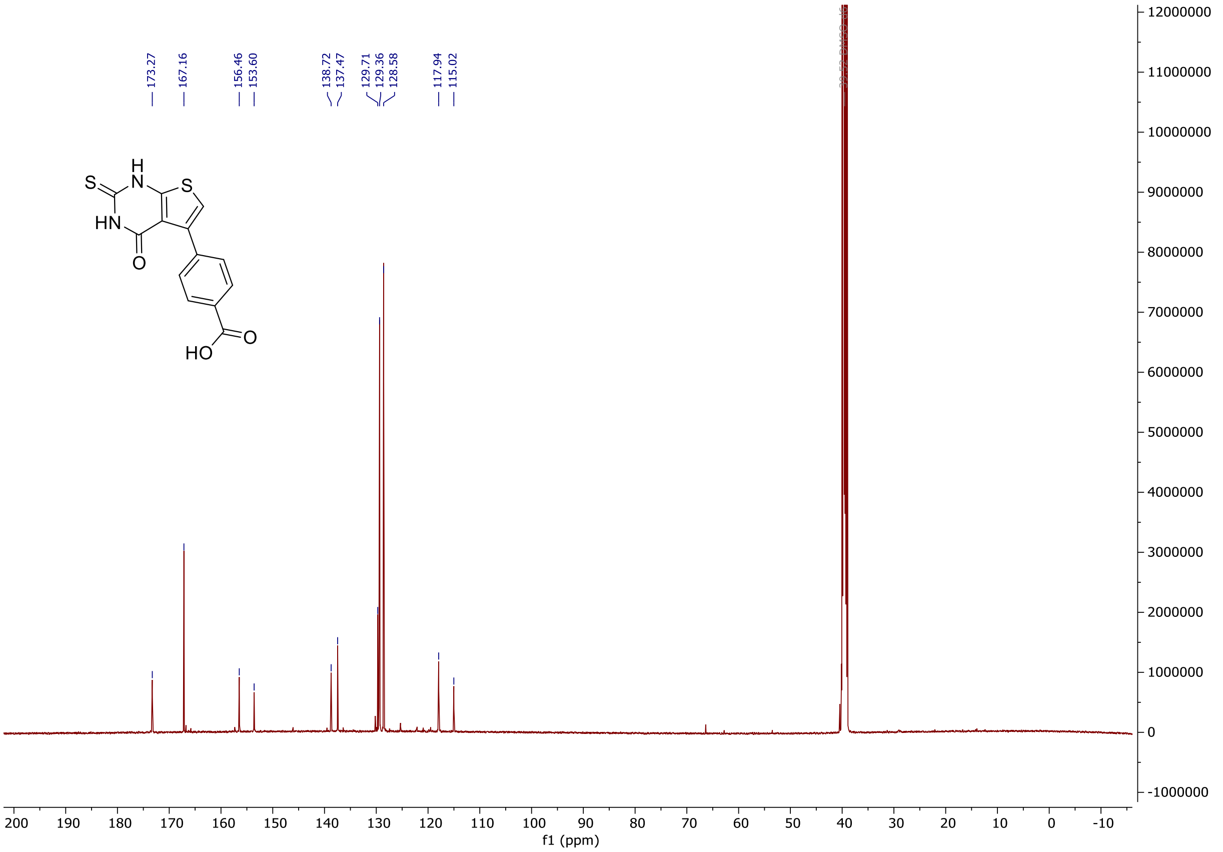

5-(4-bromophenyl)-2-thioxo-2,3-dihydrothieno[2,3-d]pyrimidin-4(1H)-one S75

^{1}H-NMR

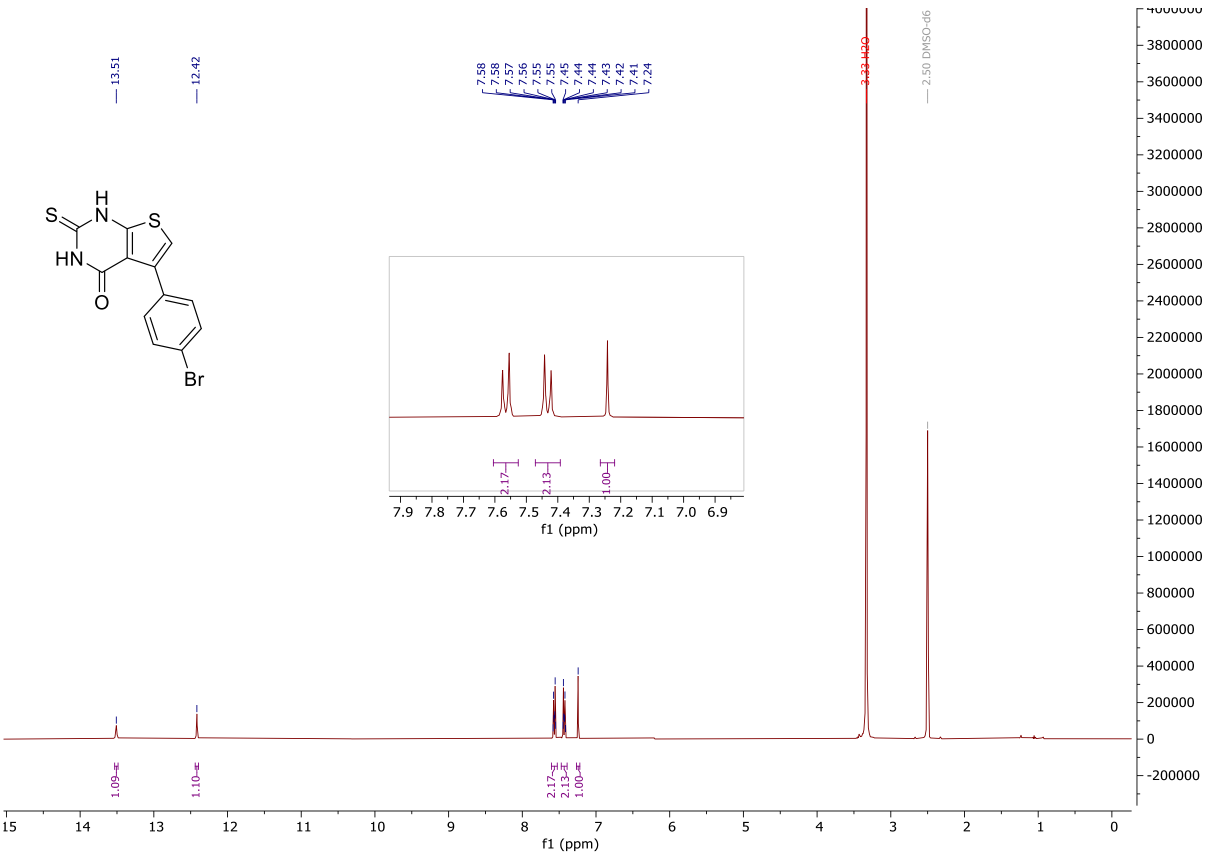

^{13}C-NMR

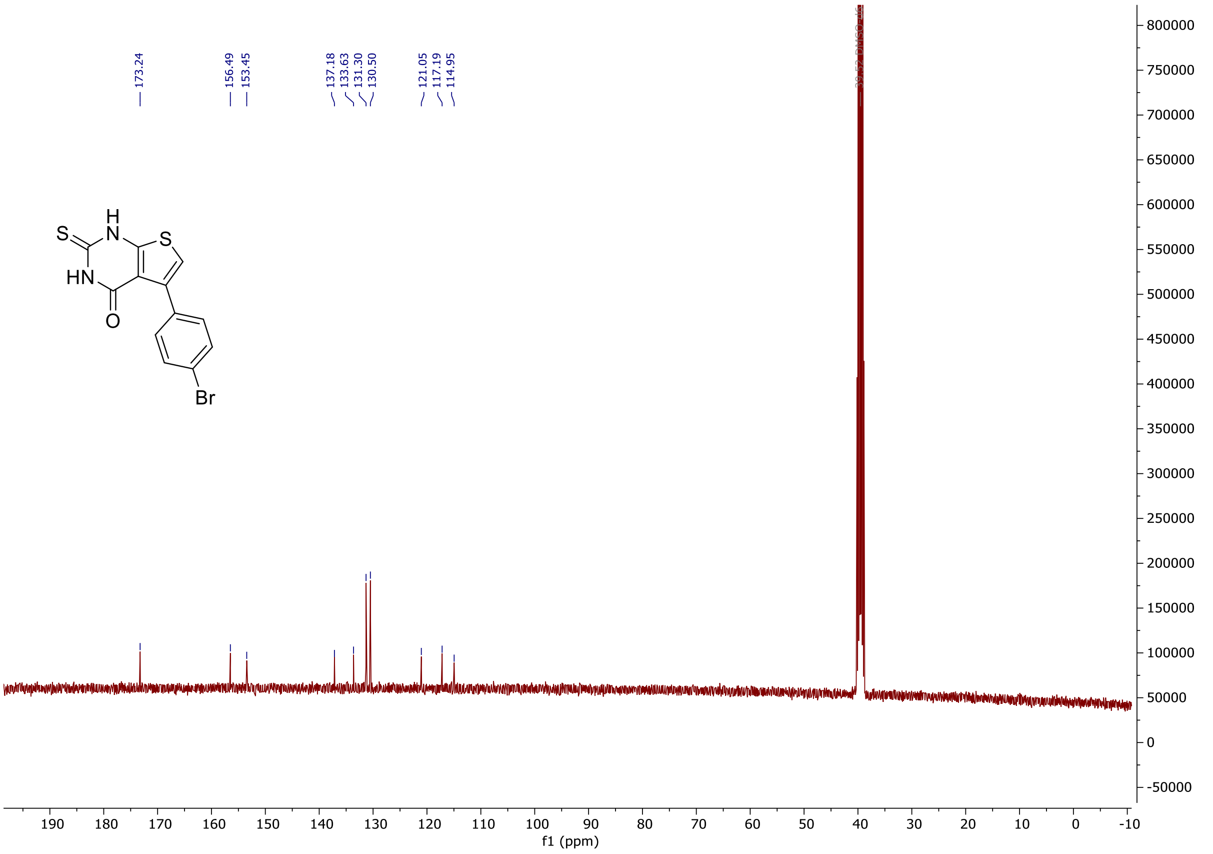

ethyl 2-(3-benzoylthioureido)-4-(4-methoxyphenyl)thiophene-3-carboxylate S76

^{1}H-NMR

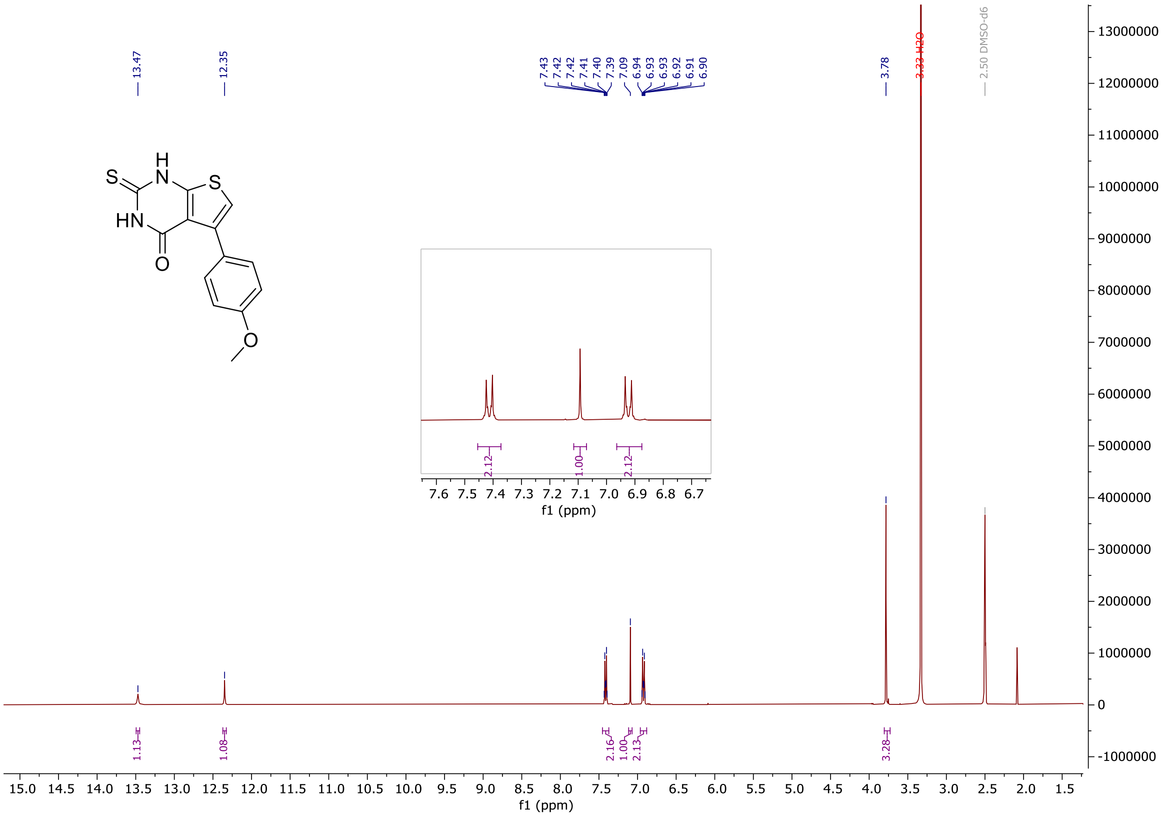

^{13}C-NMR

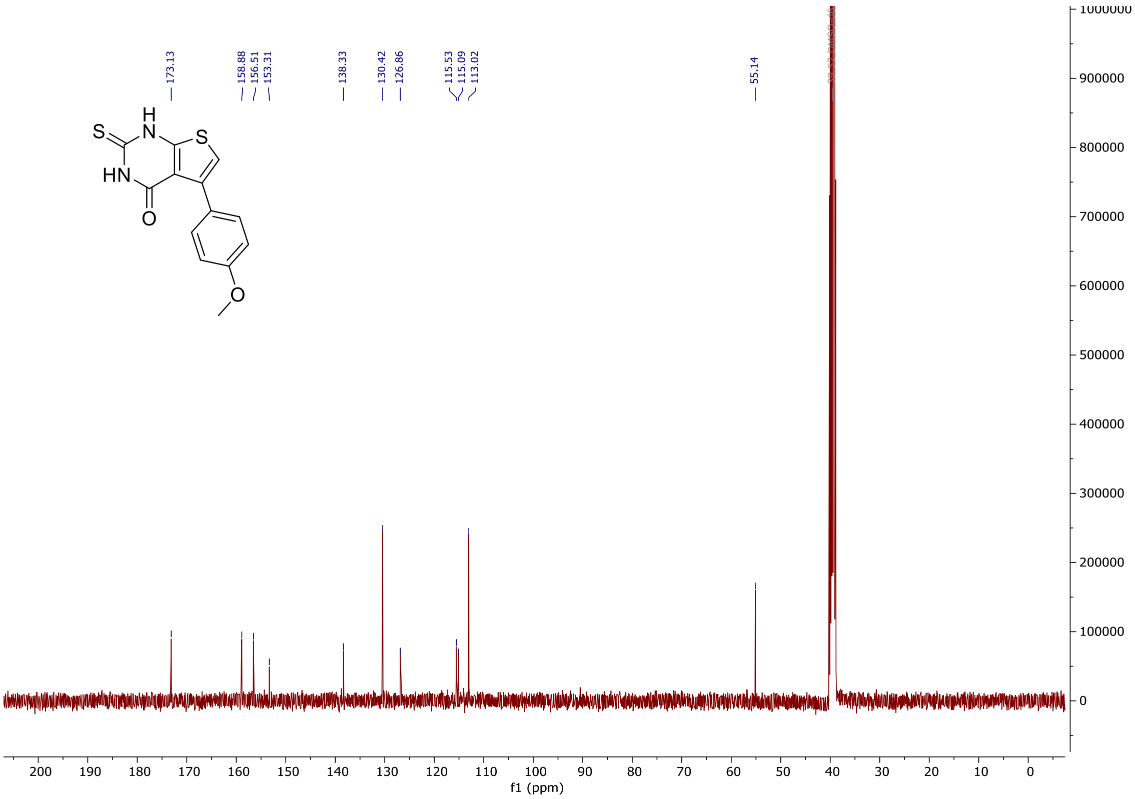

5-(4-hydroxyphenyl)-2-thioxo-2,3-dihydrothieno[2,3-d]pyrimidin-4(1H)-one 19

^{1}H-NMR

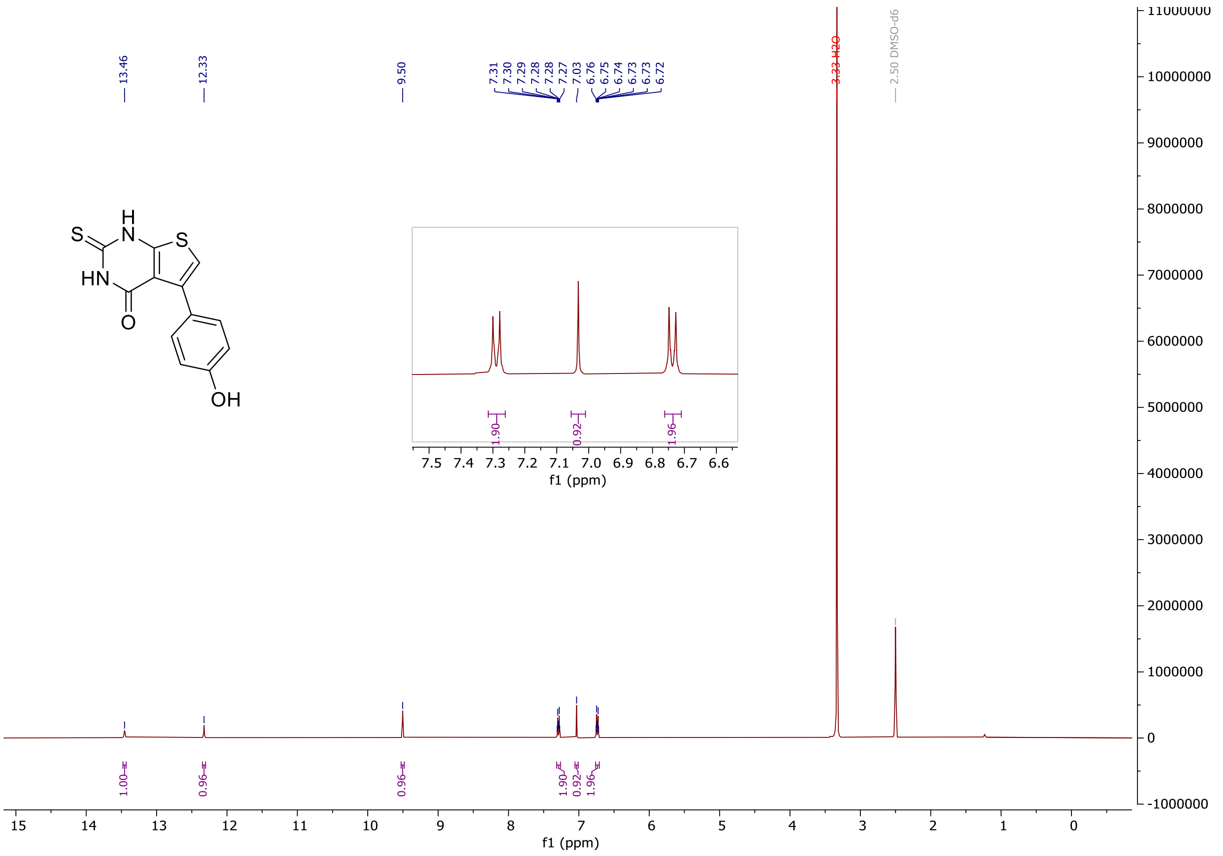

^{13}C-NMR

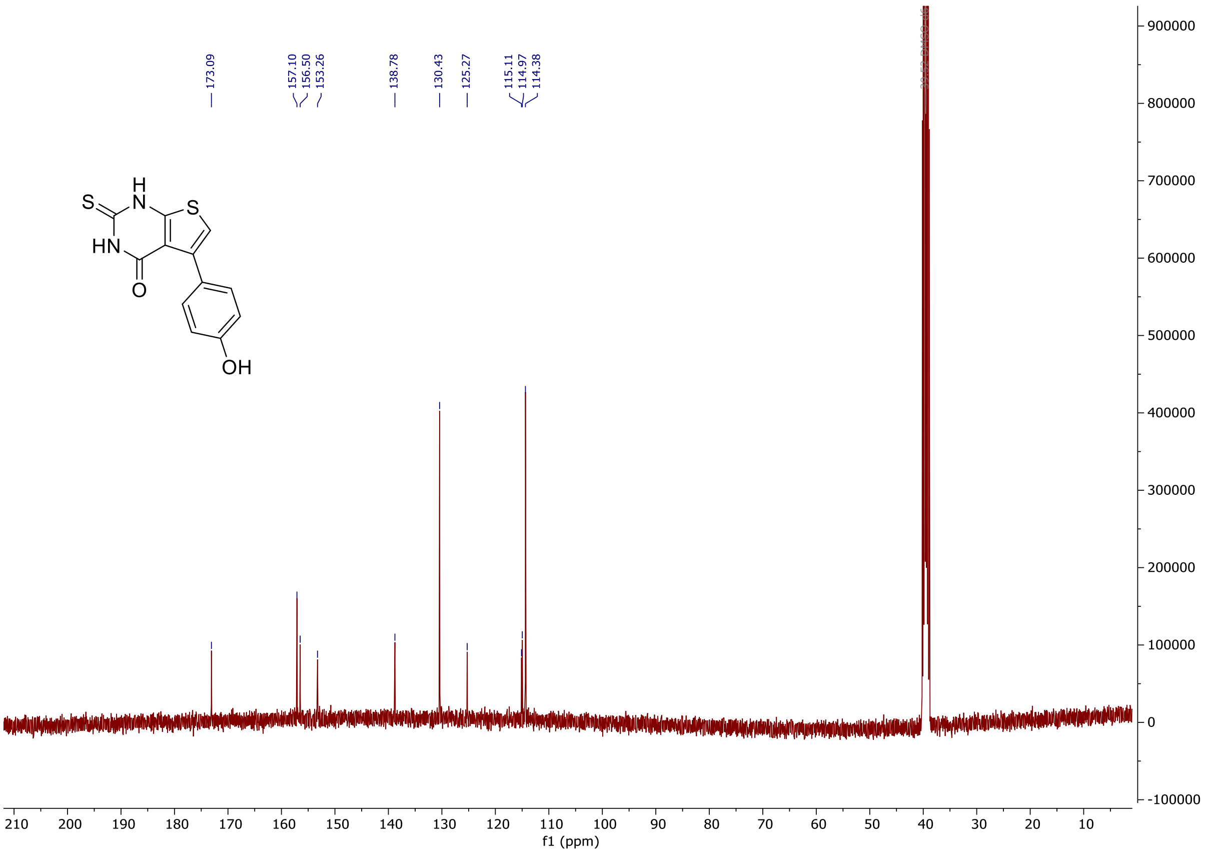

5-cyclohexyl-2-thioxo-2,3-dihydrothieno[2,3-d]pyrimidin-4(1H)-one S77

^{1}H-NMR

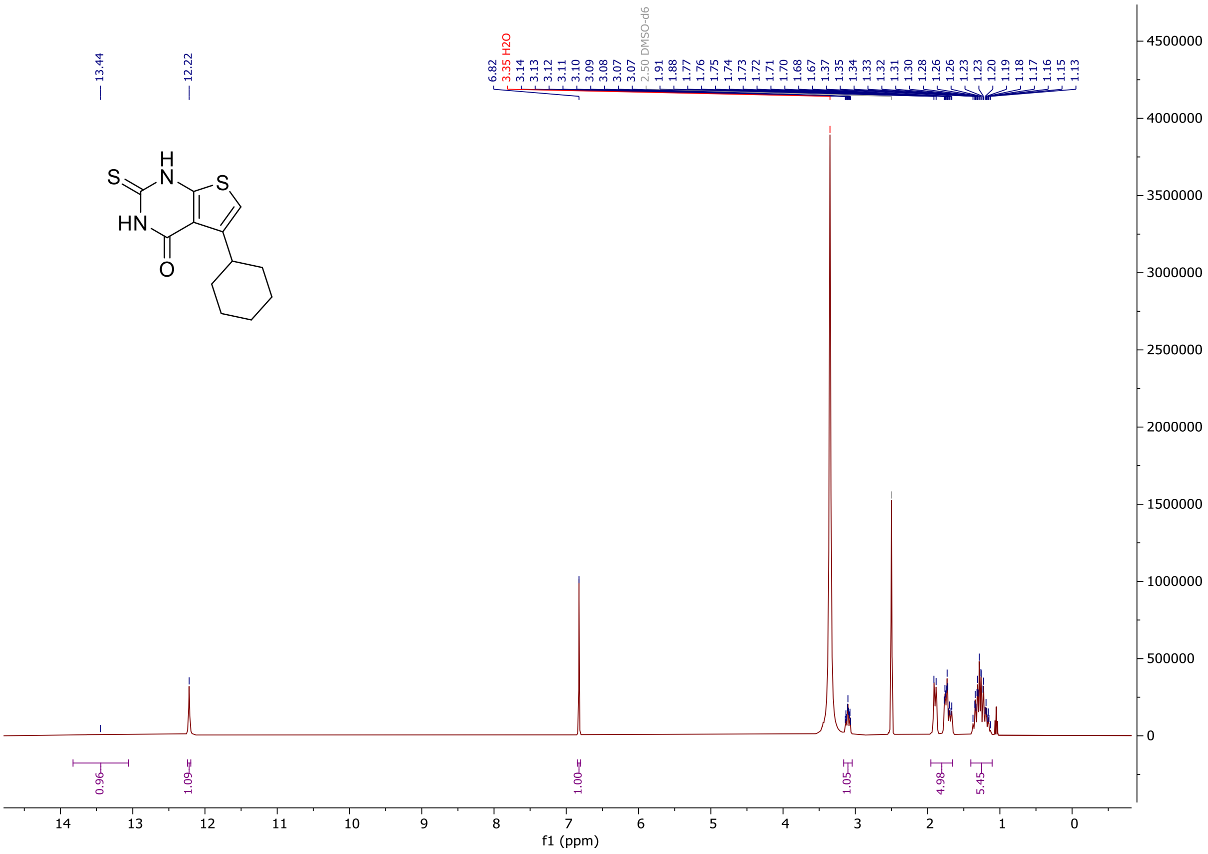

^{13}C-NMR

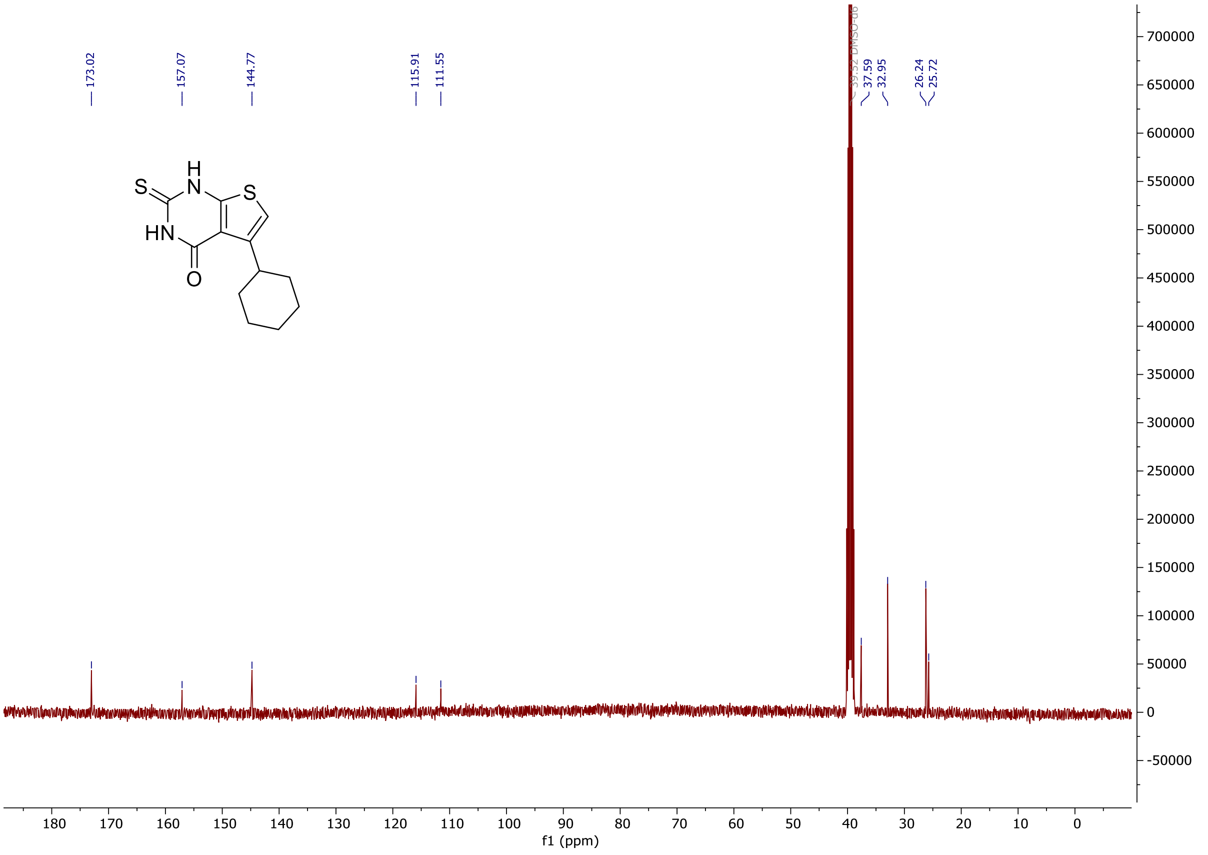

6-methyl-2-thioxo-2,3-dihydrothieno[2,3-d]pyrimidin-4(1H)-one S78

^{1}H-NMR

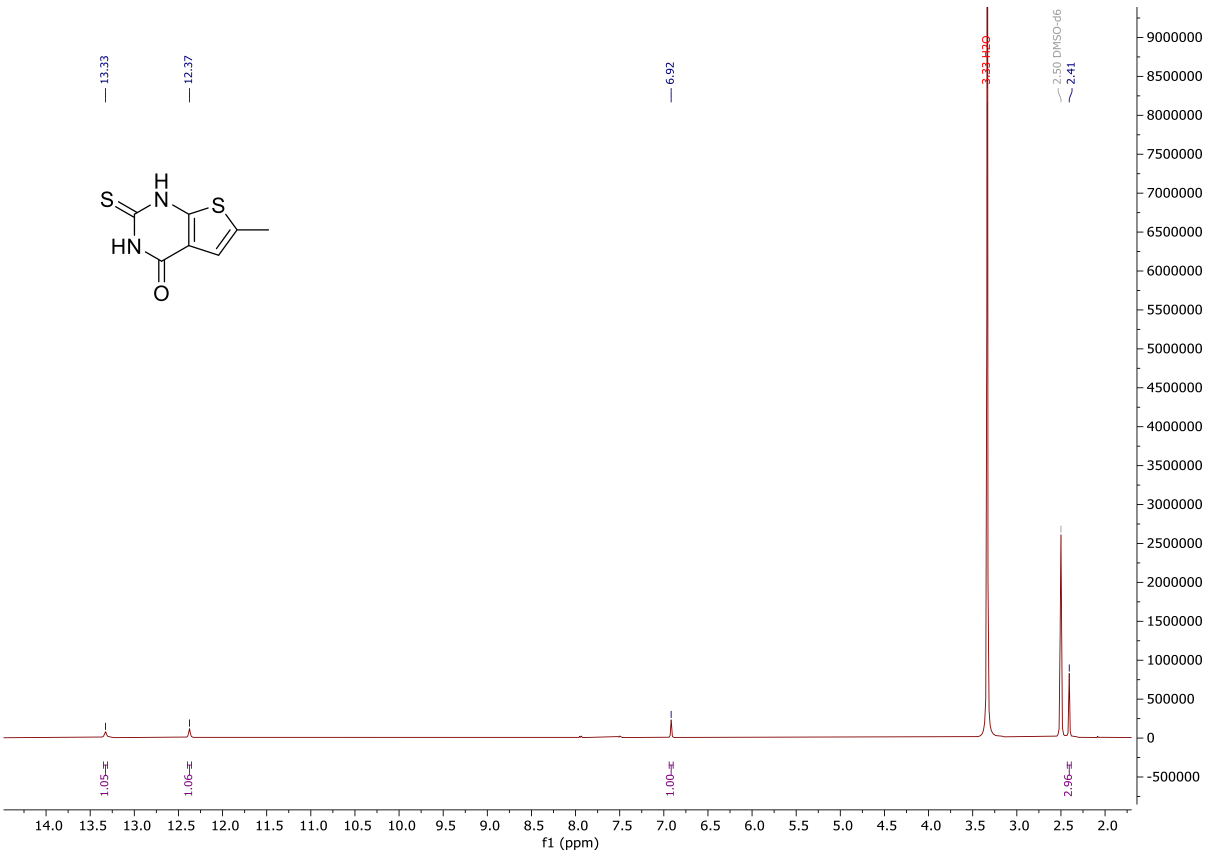

^{13}C-NMR

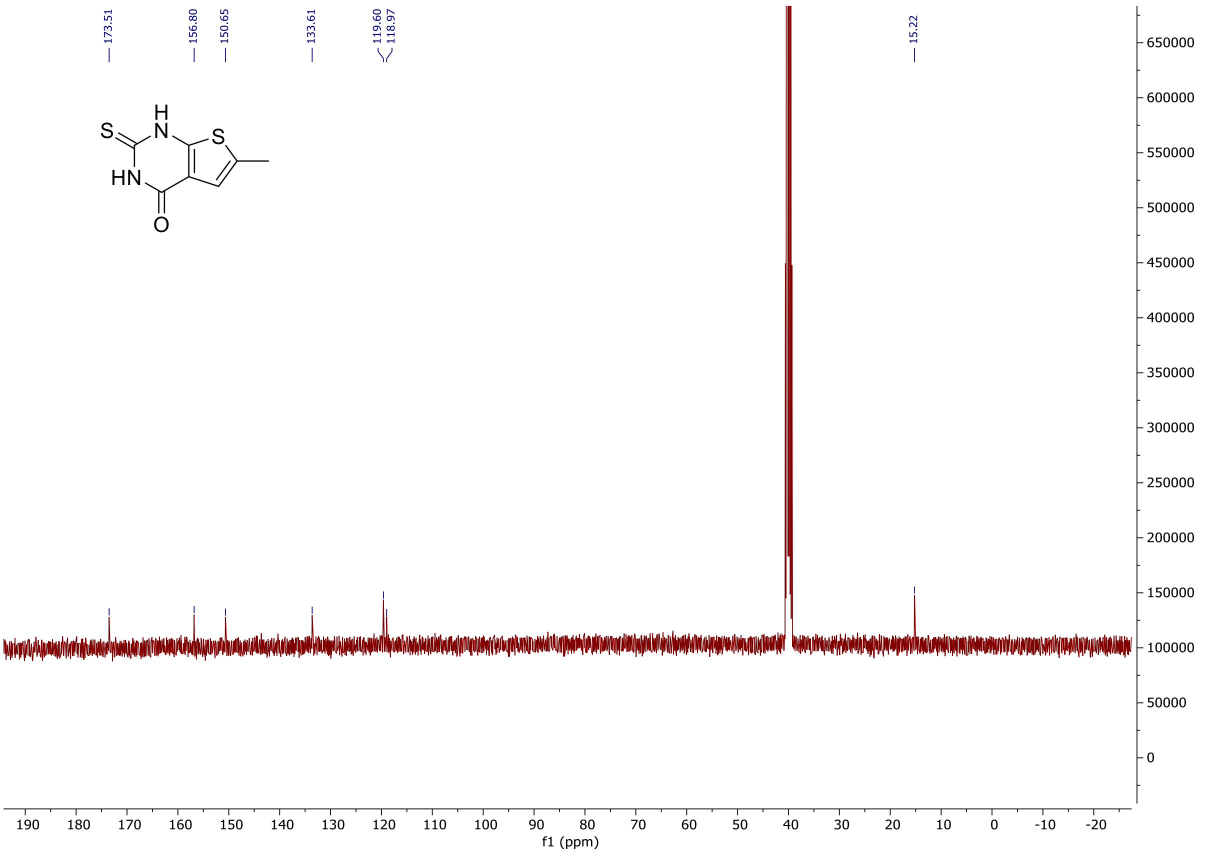

6-acetyl-5-methyl-2-thioxo-2,3-dihydrothieno[2,3-d]pyrimidin-4(1H)-one 20

^{1}H-NMR

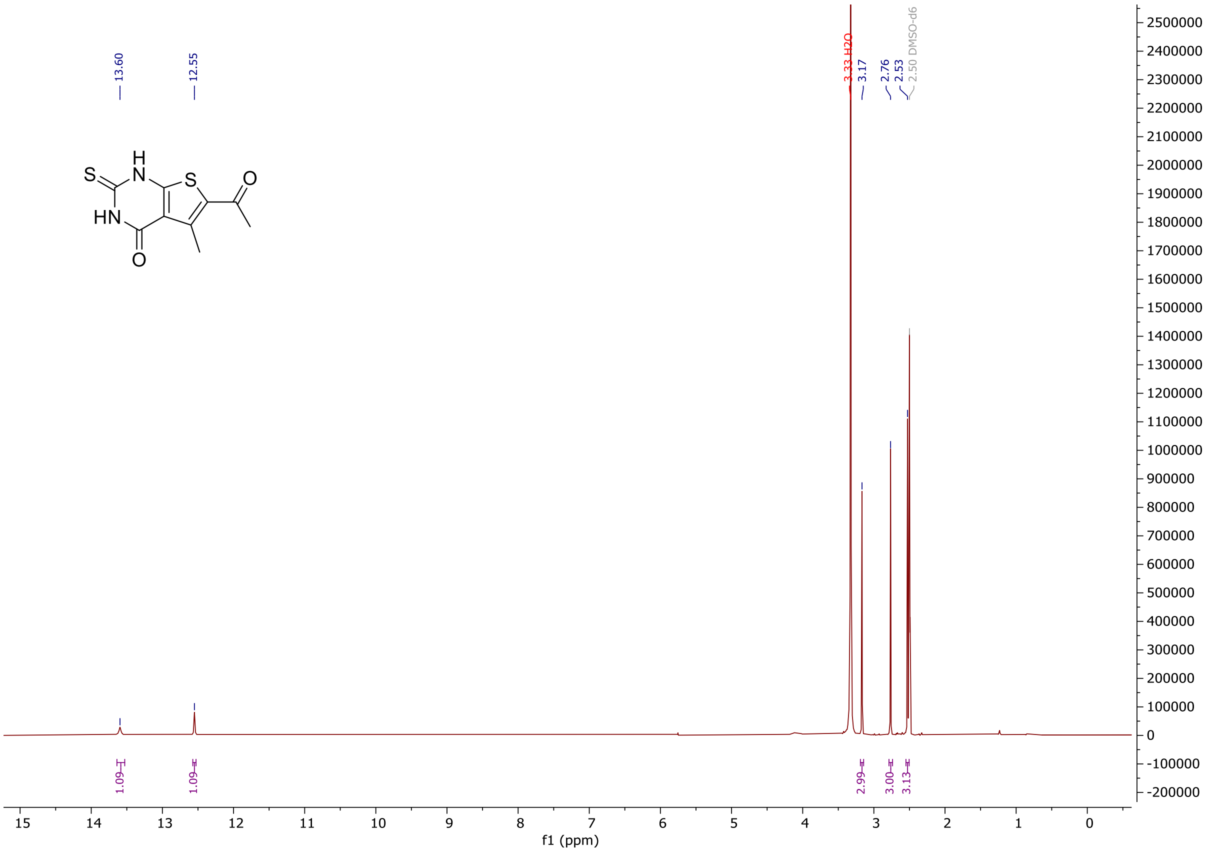

^{13}C-NMR

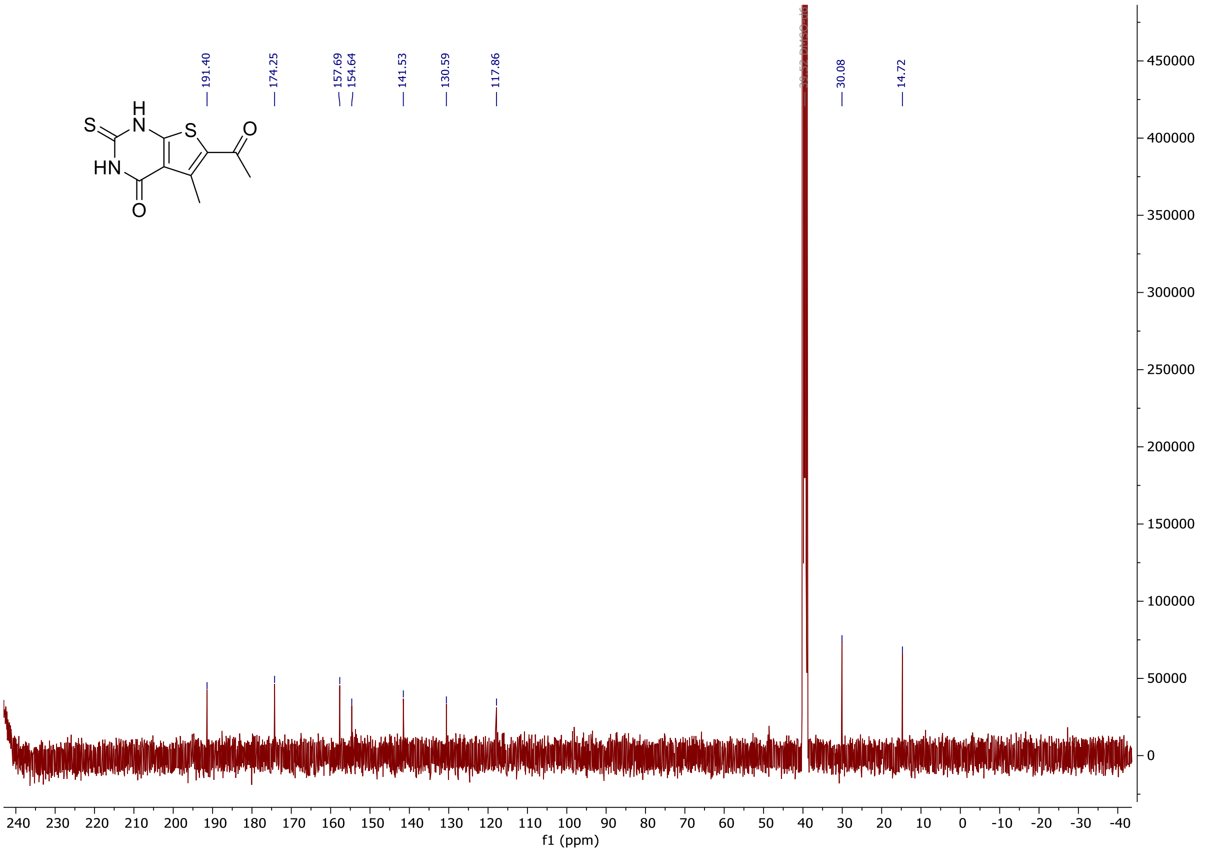

6-(1-hydroxyethyl)-5-methyl-2-thioxo-2,3-dihydrothieno[2,3-d]pyrimidin-4(1H)-one S79

^{1}H-NMR

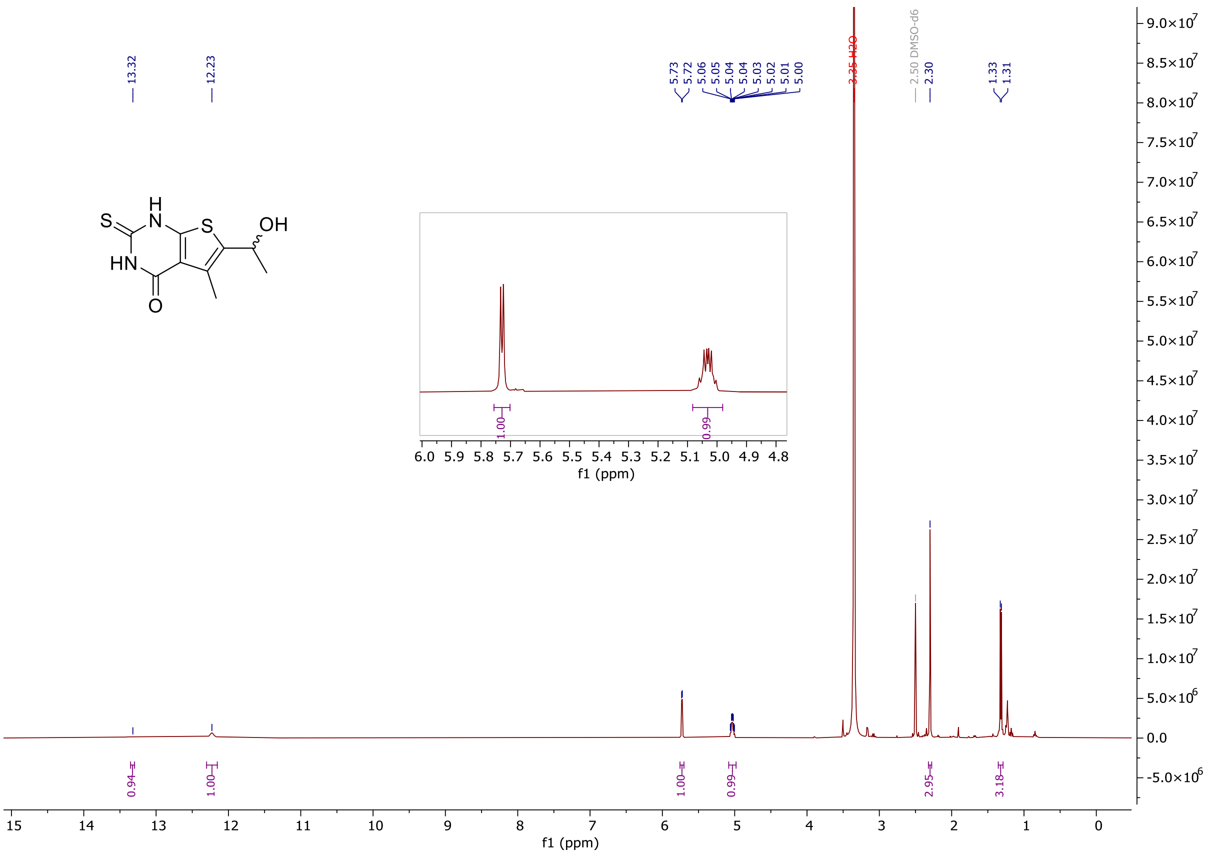

^{13}C-NMR

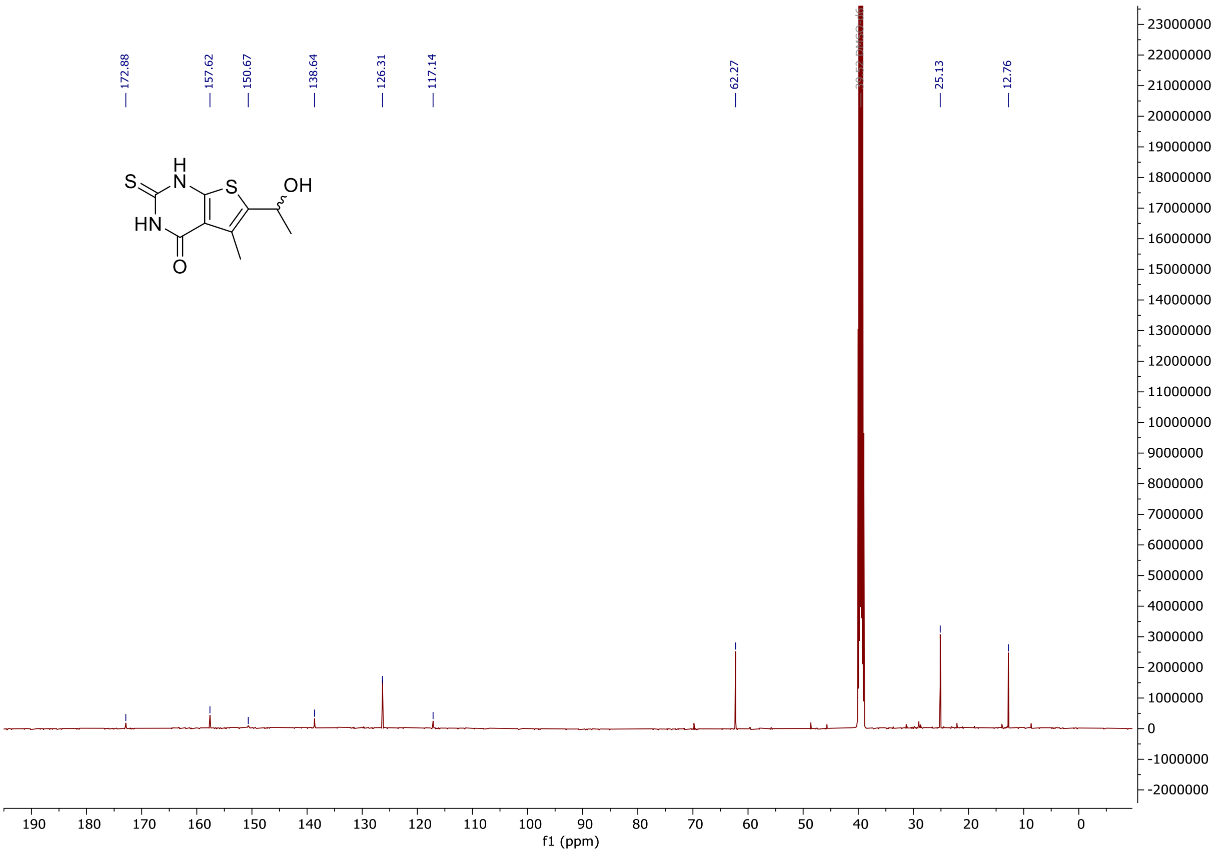

ethyl 5-methyl-4-oxo-2-thioxo-1,2,3,4-tetrahydrothieno[2,3-d]pyrimidine-6-carboxylate 21

^{1}H-NMR

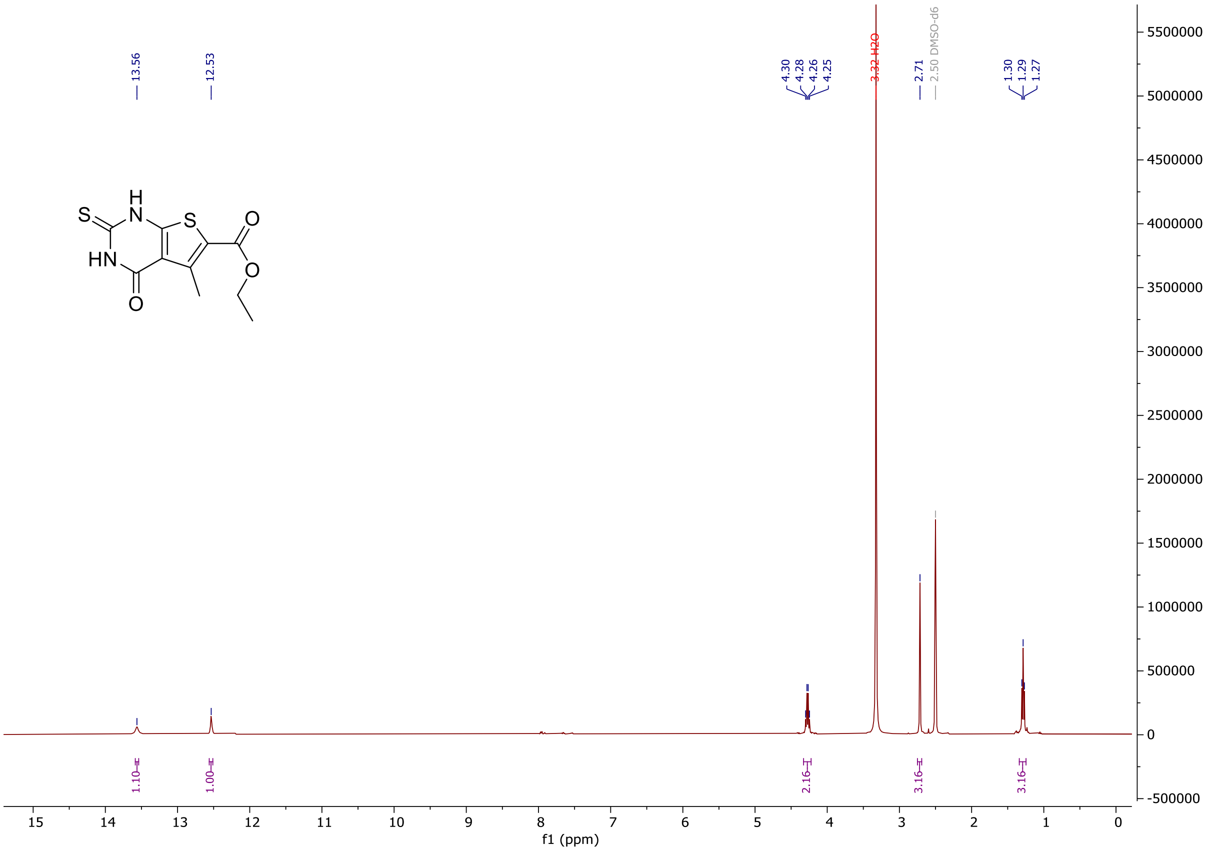

^{13}C-NMR

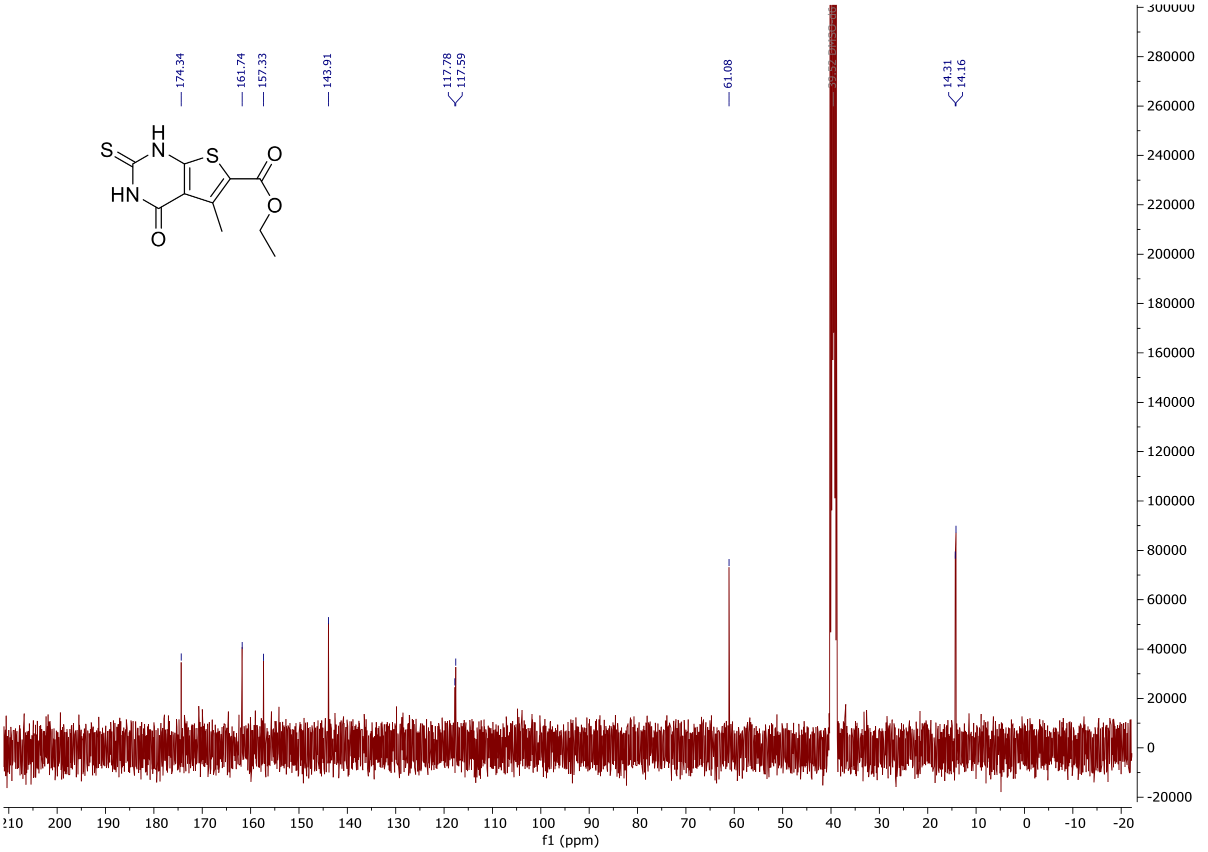

5-methyl-4-oxo-2-thioxo-1,2,3,4-tetrahydrothieno[2,3-d]pyrimidine-6-carboxylic acid S80

^{1}H-NMR

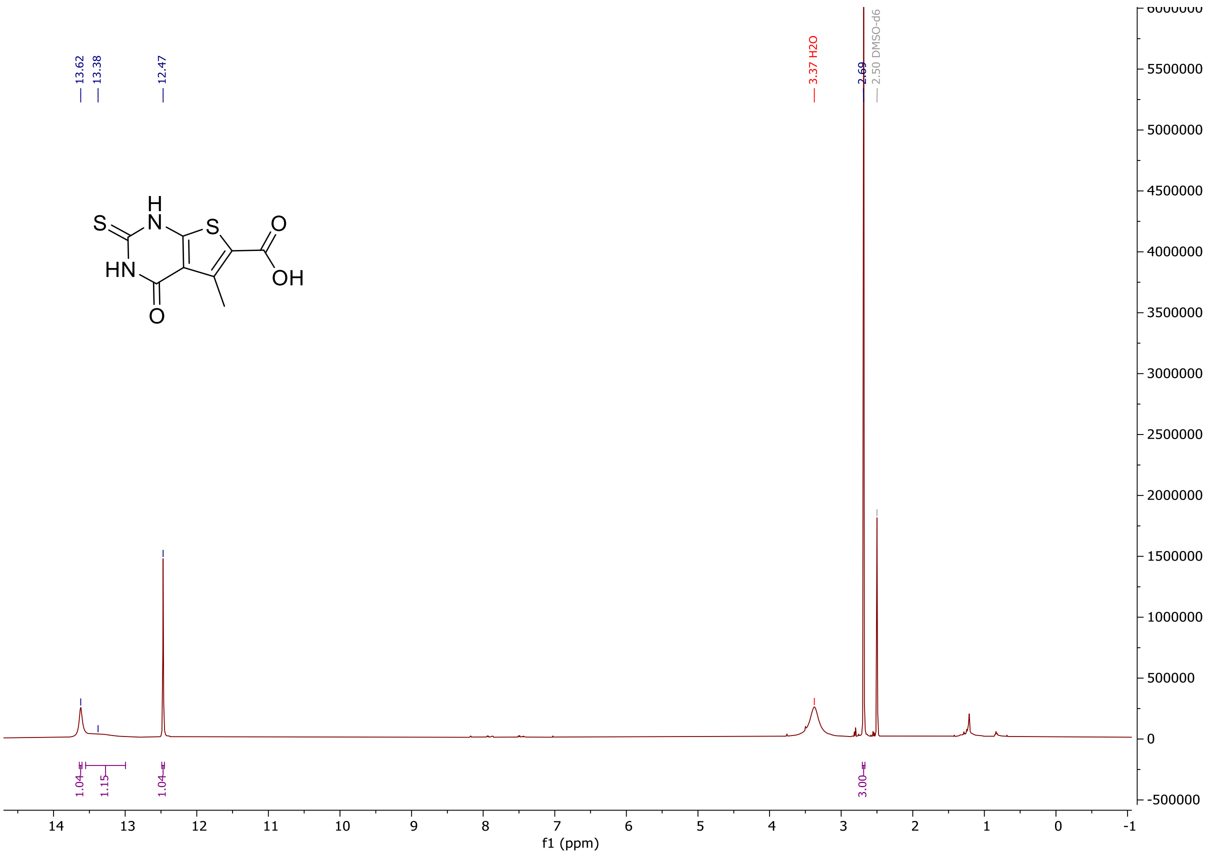

^{13}C-NMR

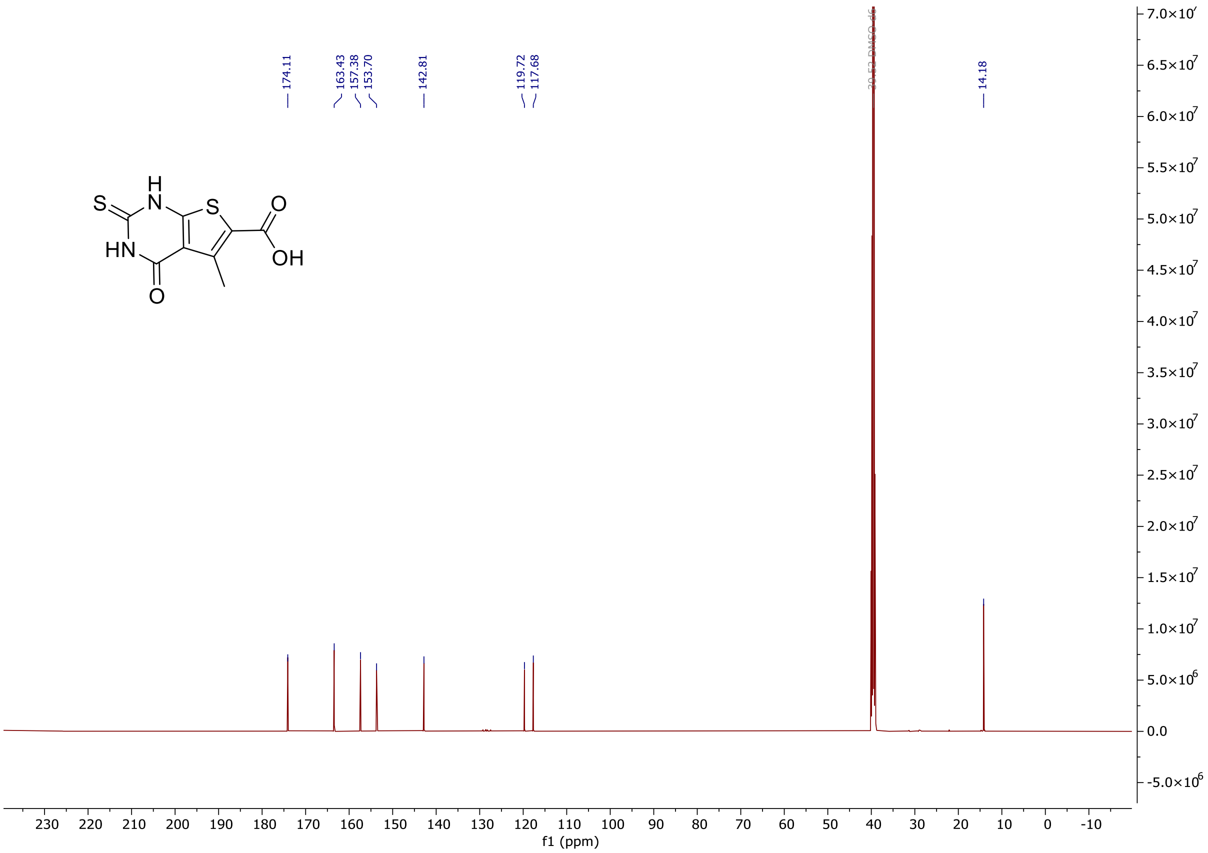

N-benzyl-5-methyl-4-oxo-2-thioxo-1,2,3,4-tetrahydrothieno[2,3-d]pyrimidine-6-carboxamide 22

^{1}H-NMR

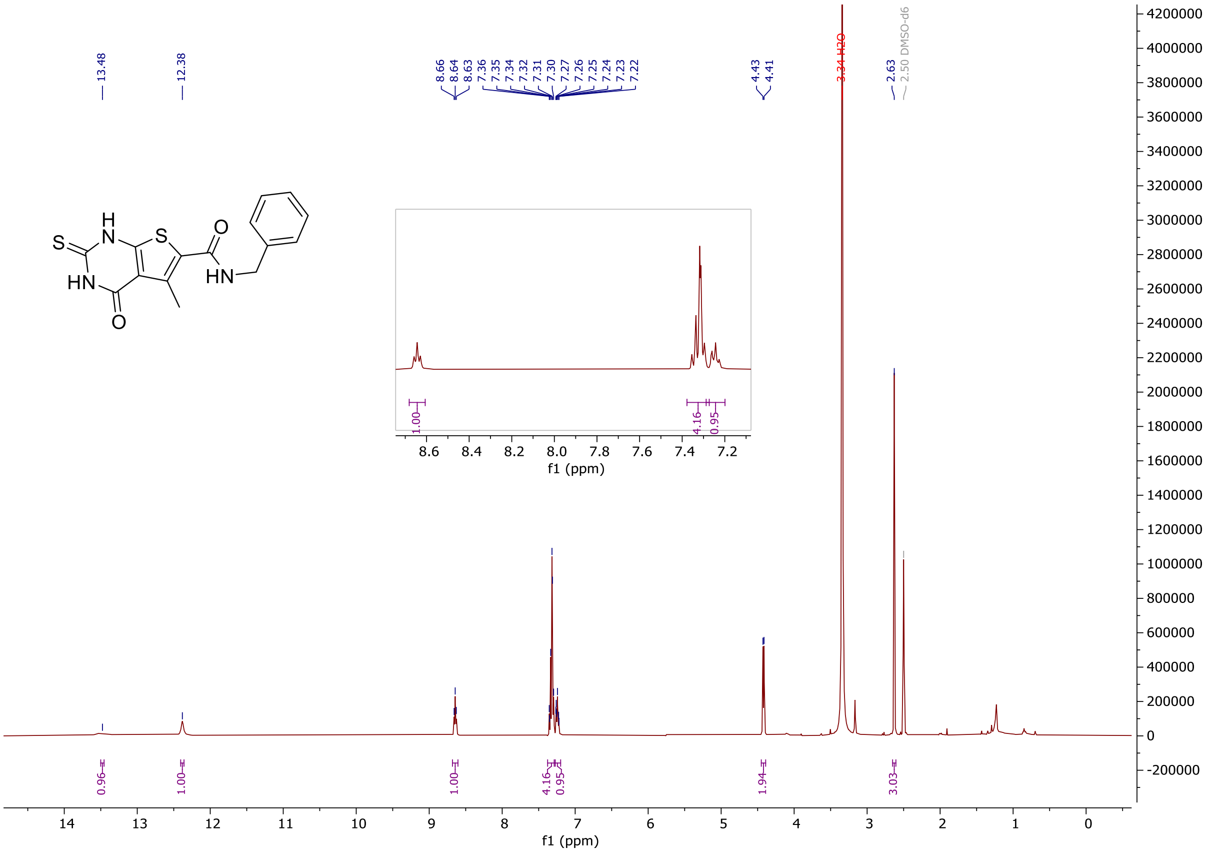

^{13}C-NMR

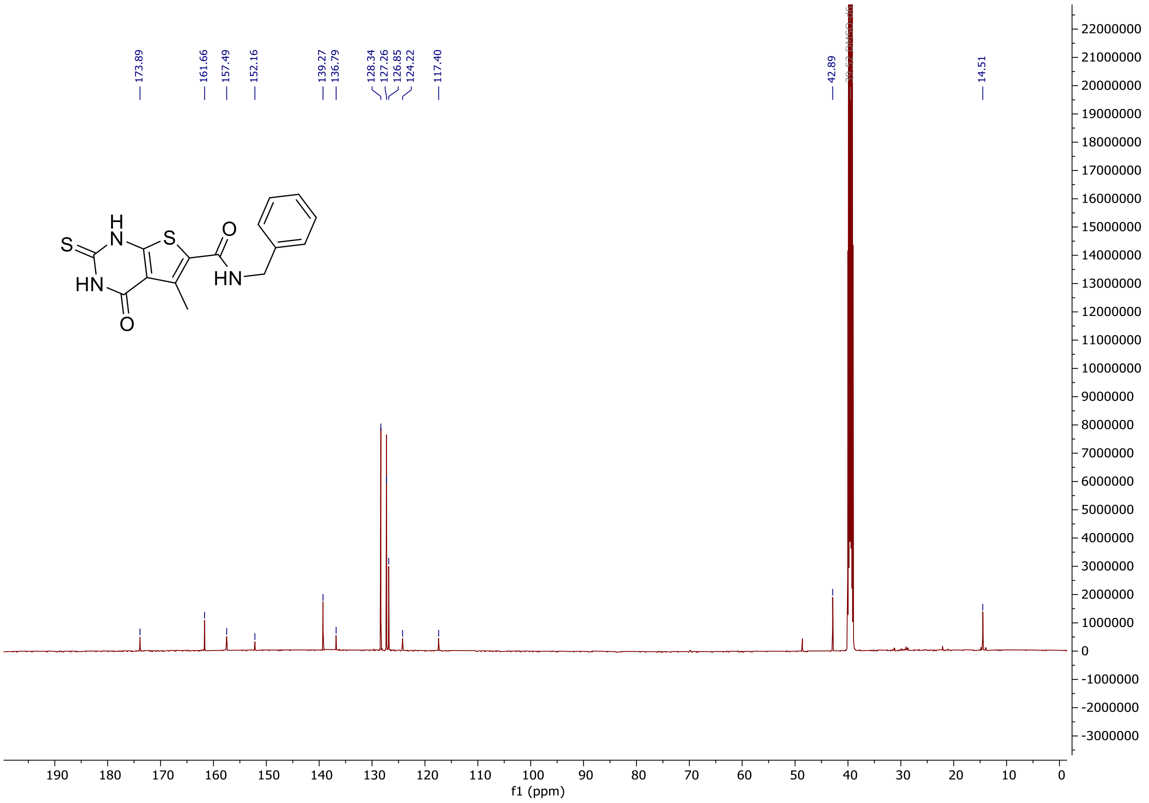

5-methyl-4-oxo-N-(pyridin-3-ylmethyl)-2-thioxo-1,2,3,4-tetrahydrothieno[2,3-d]pyrimidine-6-carboxamide 23

^{1}H-NMR

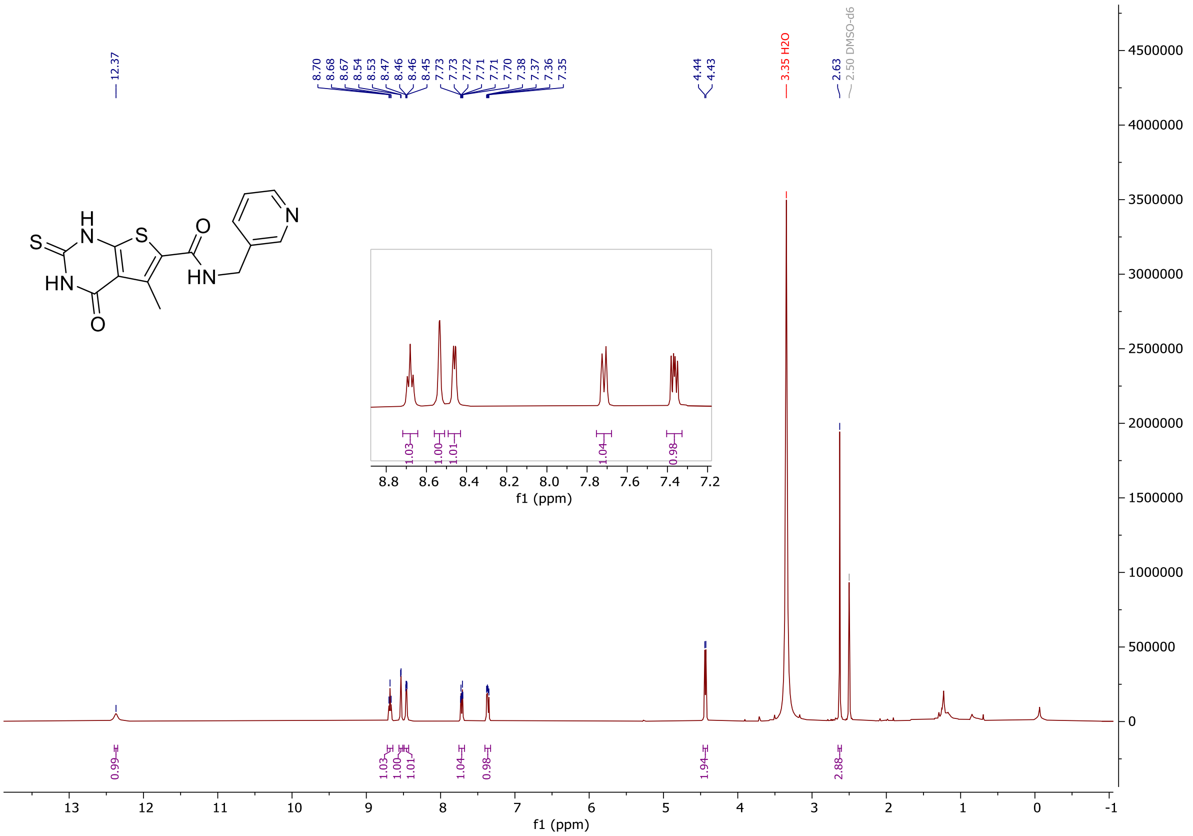

^{13}C-NMR

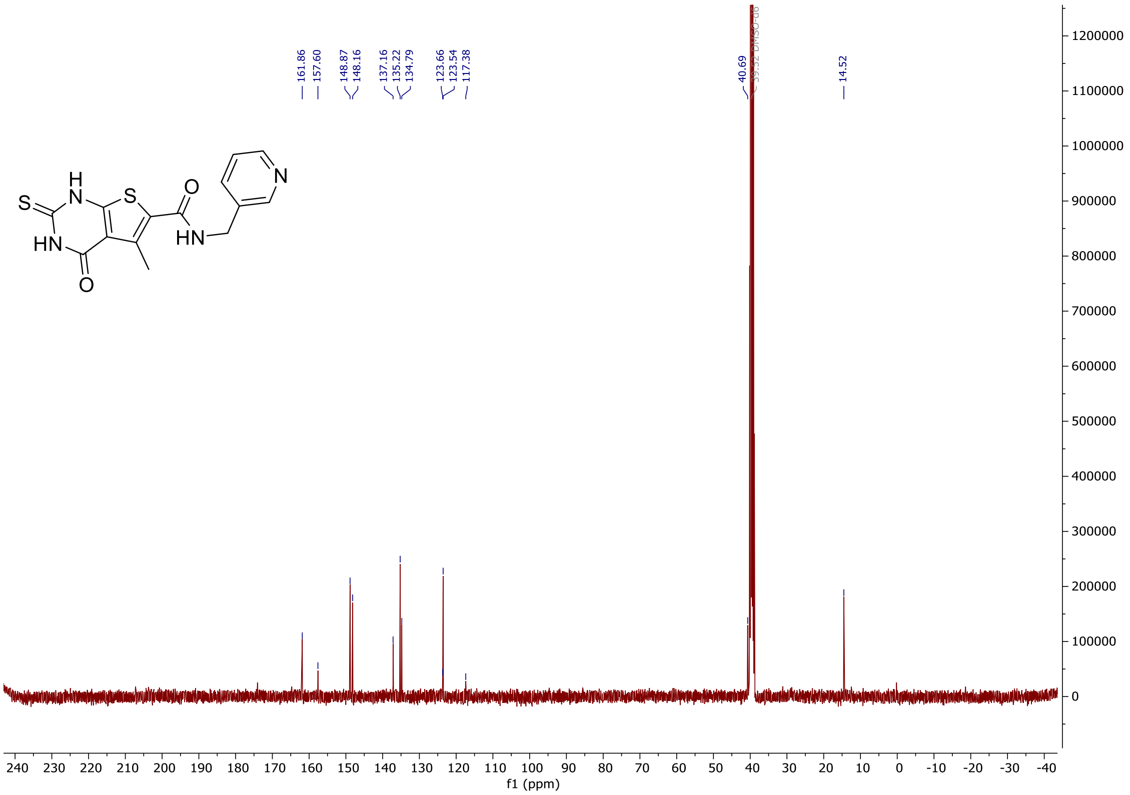

***tert*-butyl 3-((5-methyl-4-oxo-2-thioxo-1,2,3,4-tetrahydrothieno[2,3-d]pyrimidine-6-carboxamido)methyl)benzoate S81**

^{1}H-NMR

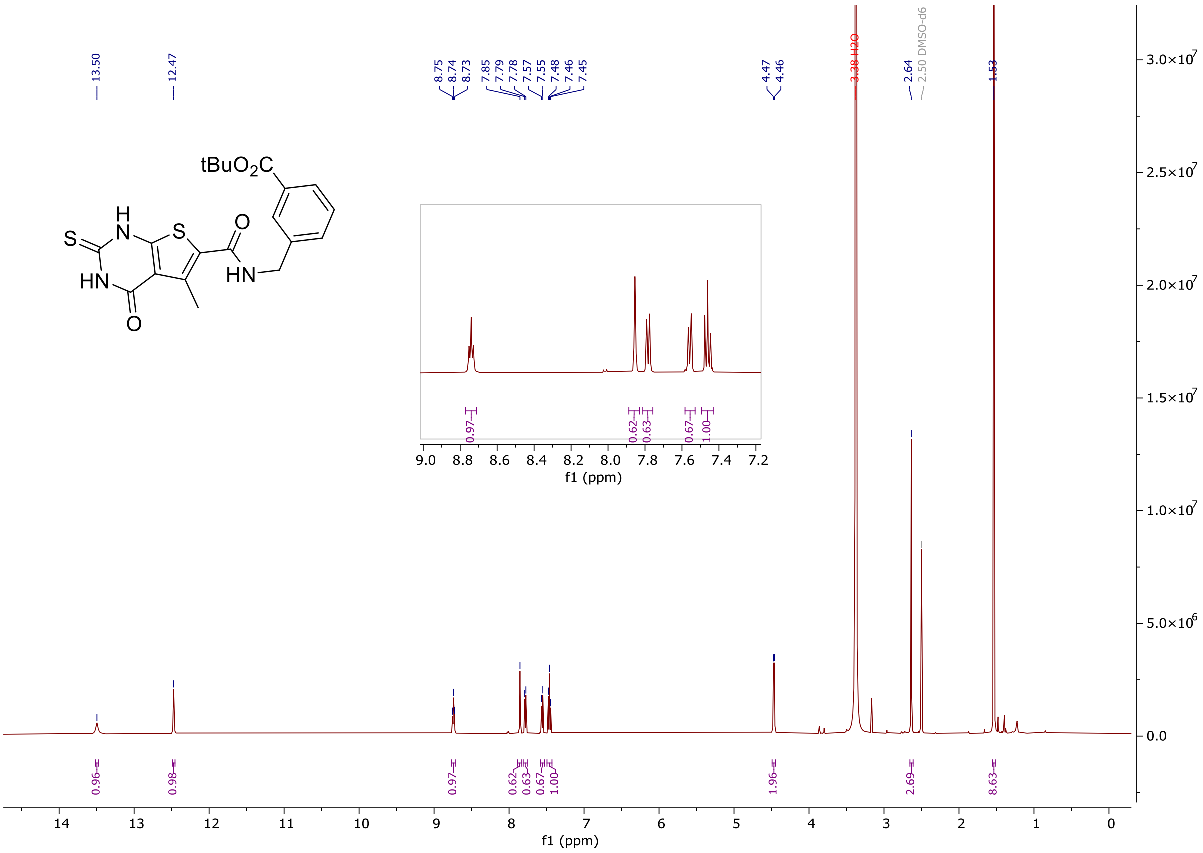

^{13}C-NMR

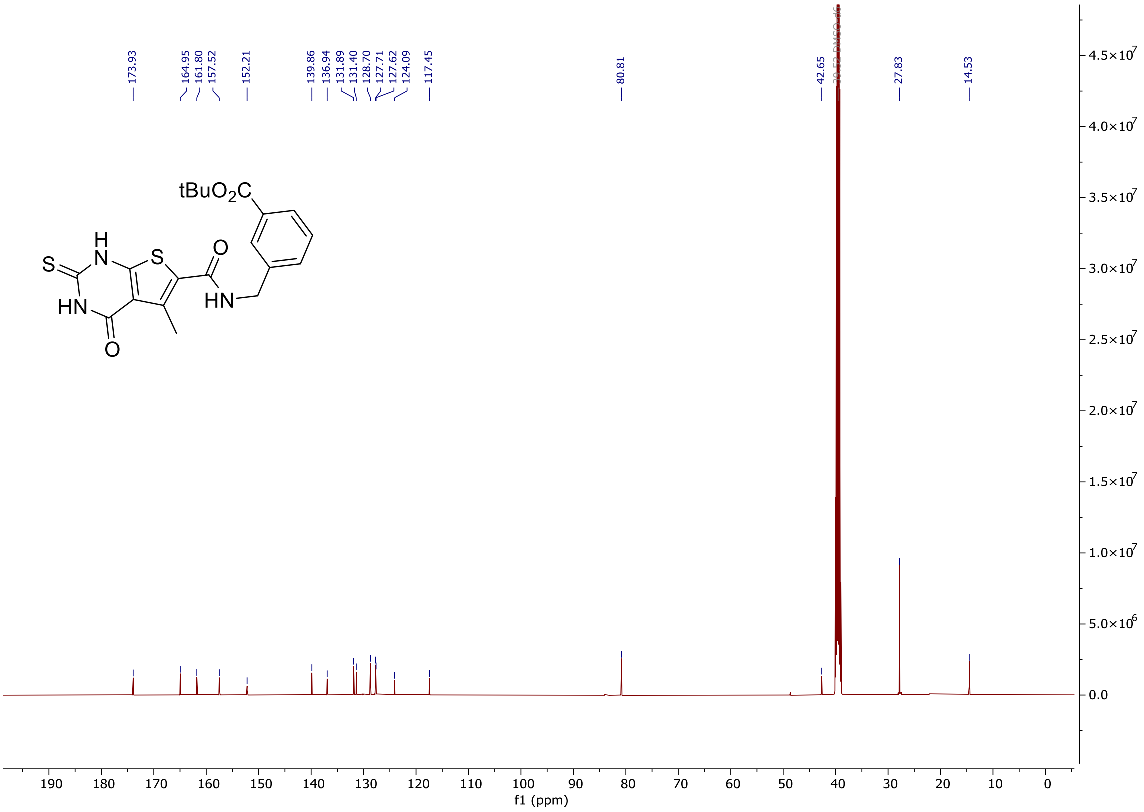

3-((5-methyl-4-oxo-2-thioxo-1,2,3,4-tetrahydrothieno[2,3-d]pyrimidine-6-carboxamido)methyl)benzoic acid 24

^{1}H-NMR

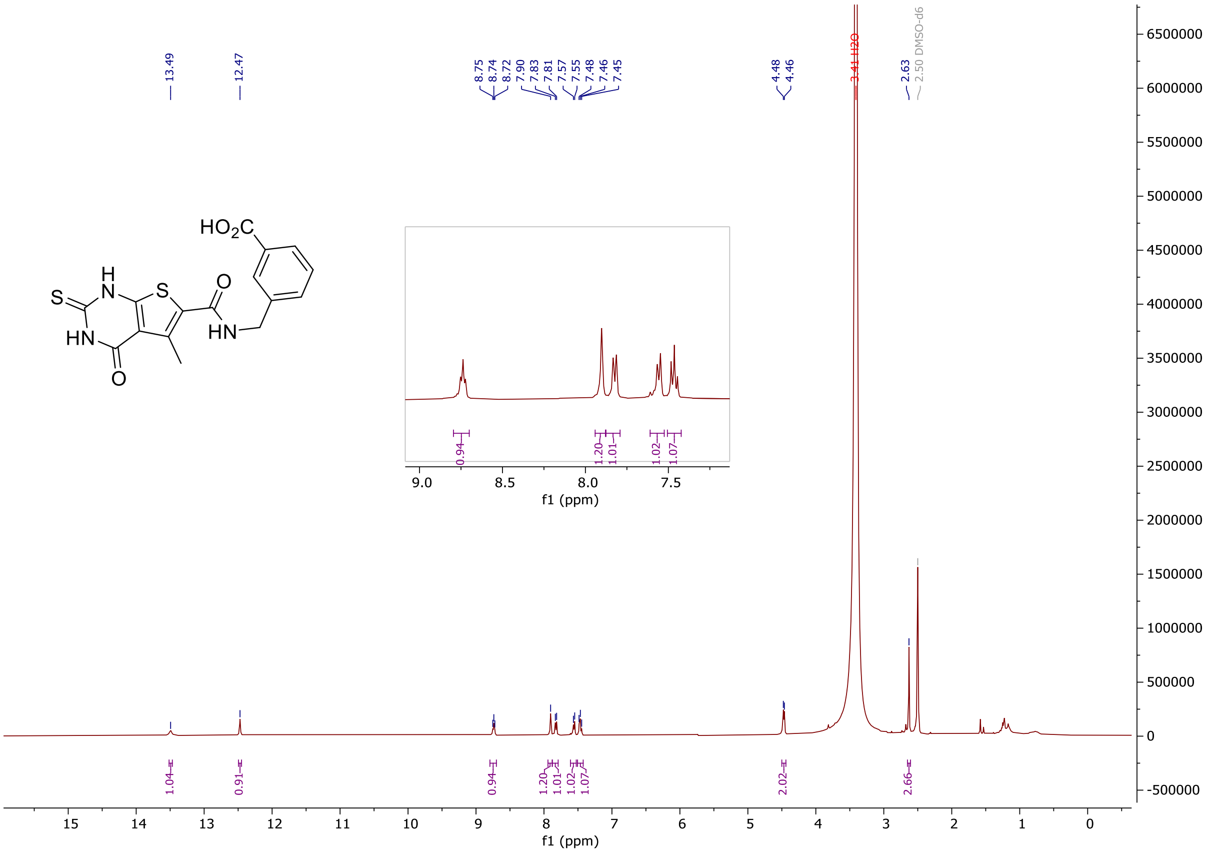

^{13}C-NMR

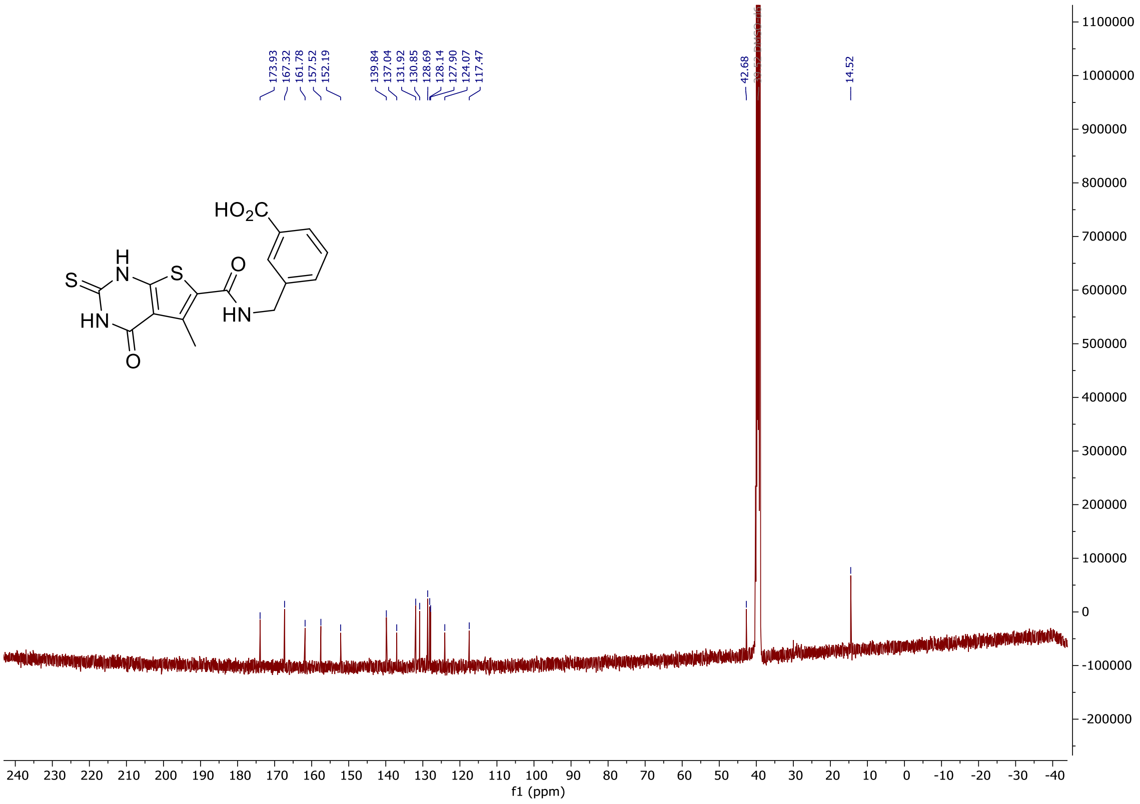

***tert*-butyl 4-(5-methyl-4-oxo-2-thioxo-1,2,3,4-tetrahydrothieno[2,3-d]pyrimidine-6-carboxamido)butanoate S82**

^{1}H-NMR

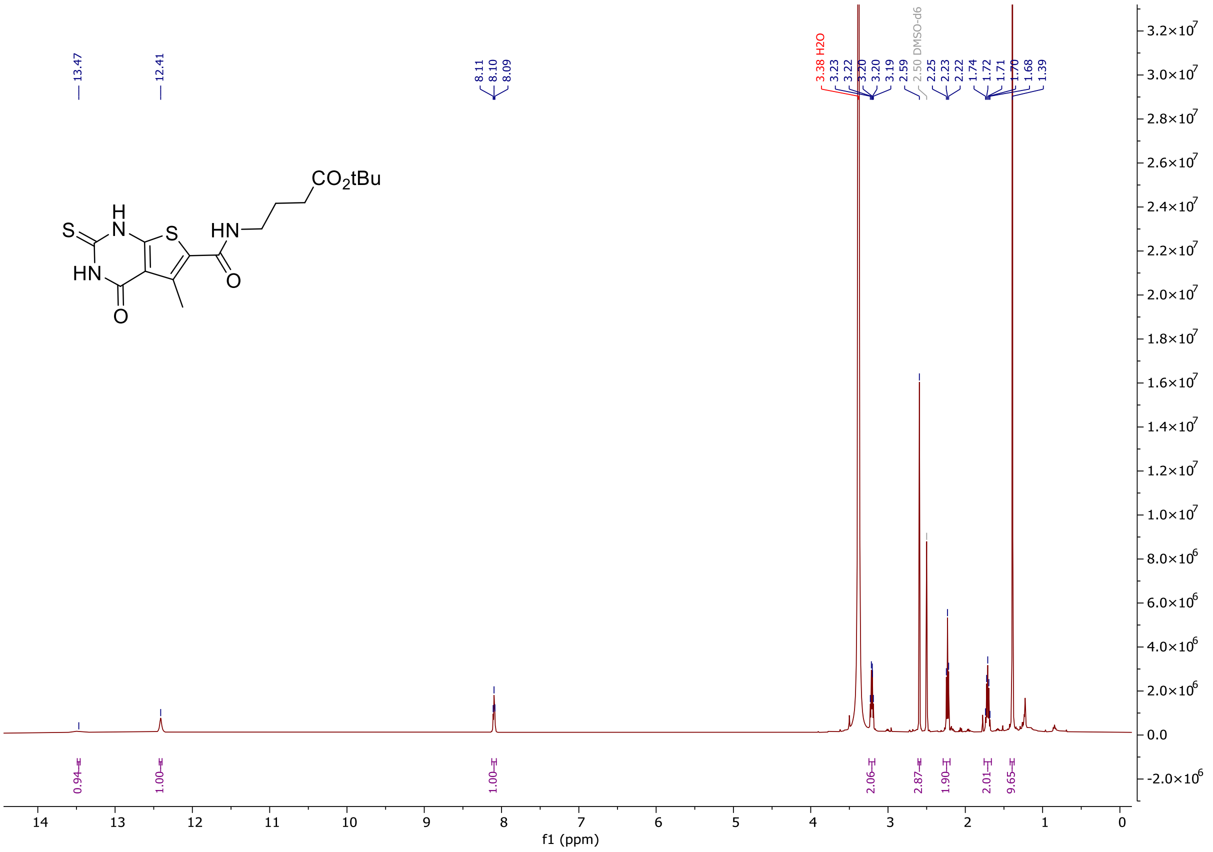

^{13}C-NMR

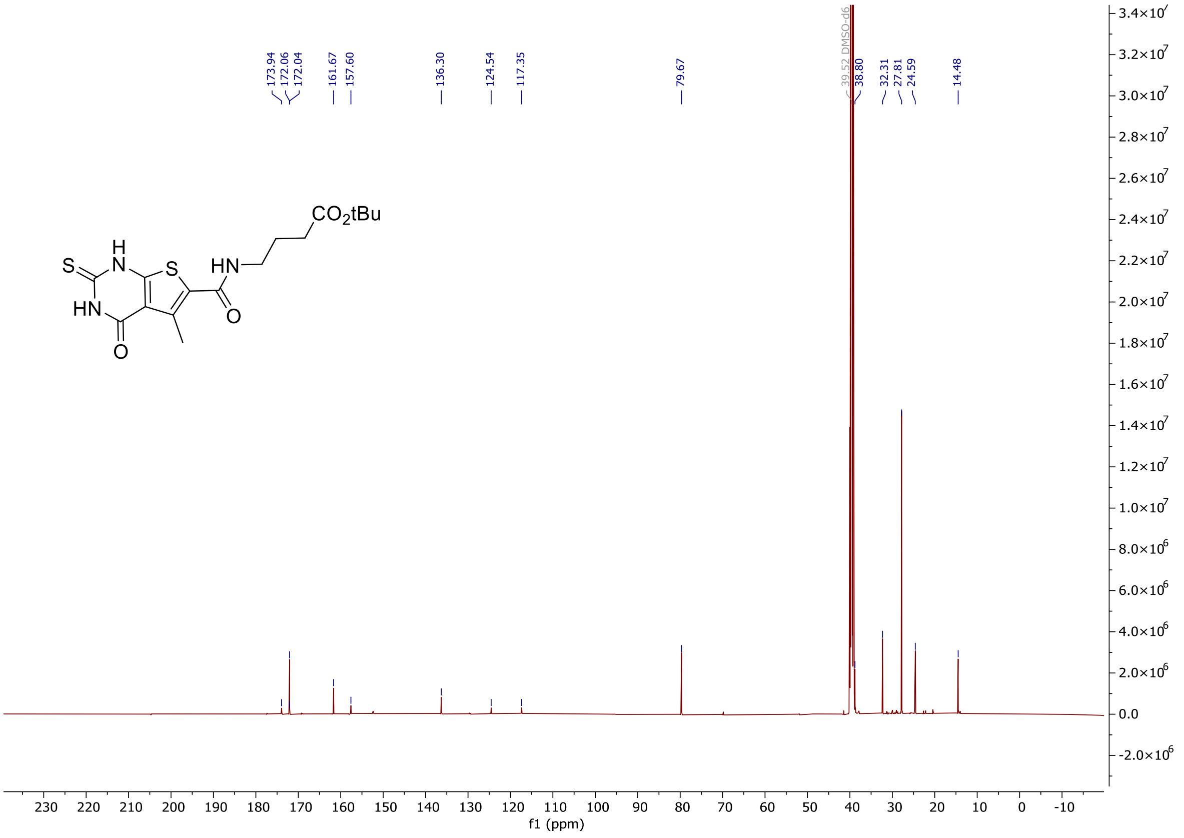

4-(5-methyl-4-oxo-2-thioxo-1,2,3,4-tetrahydrothieno[2,3-d]pyrimidine-6-carboxamido)butanoic acid 25

^{1}H-NMR

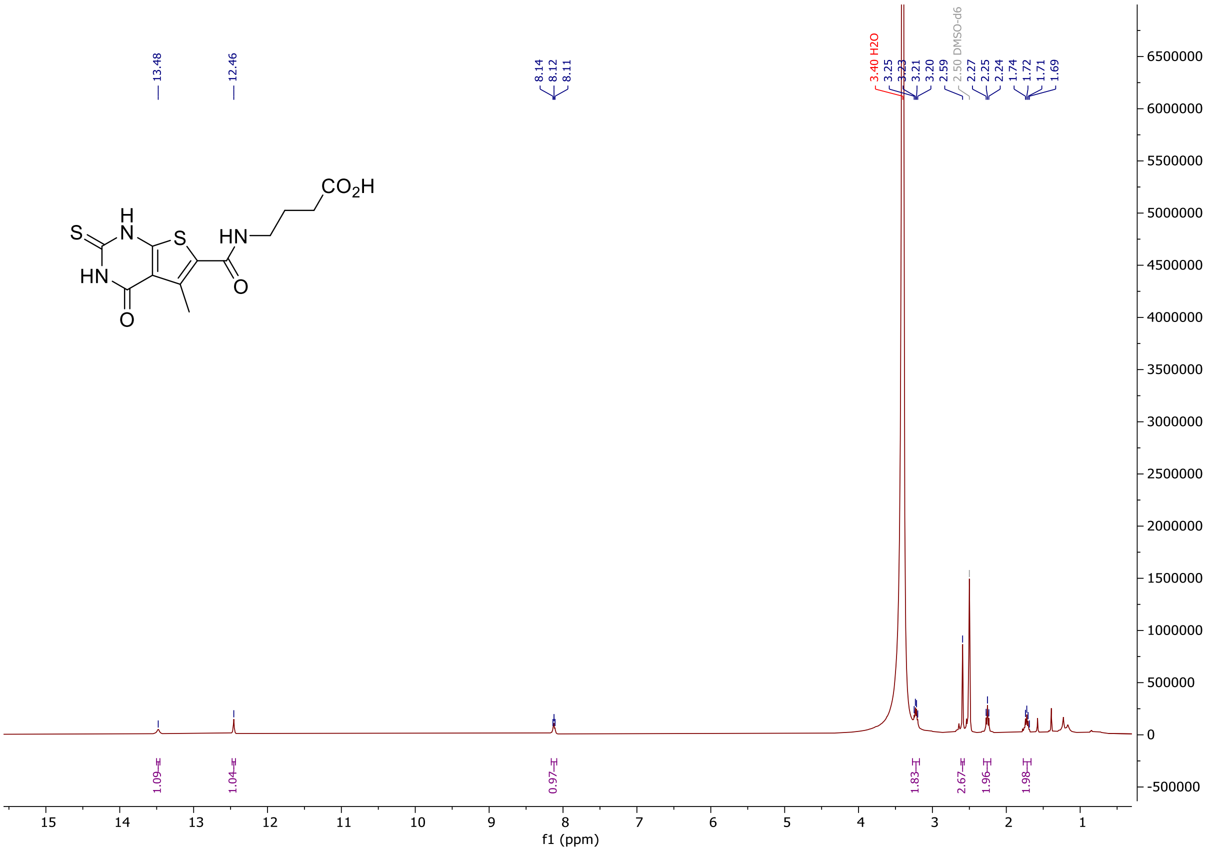

^{13}C-NMR

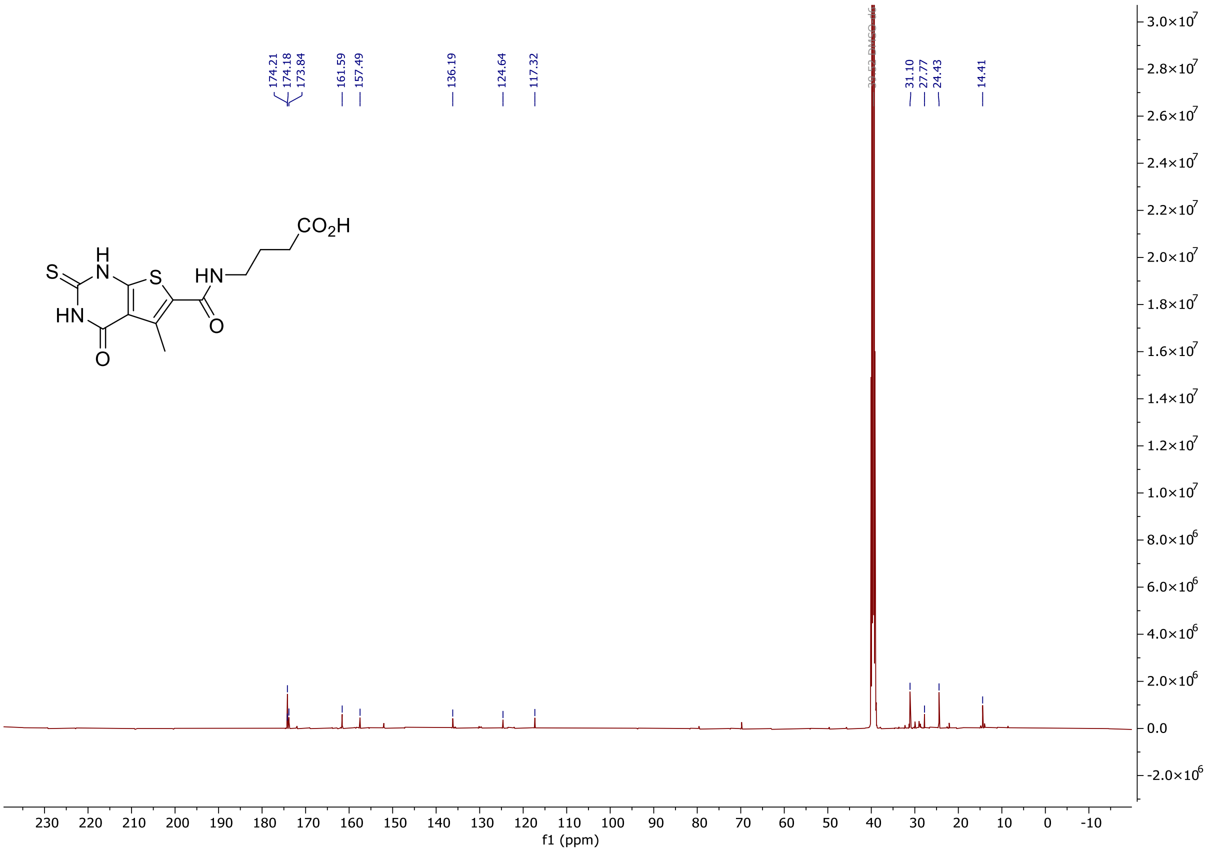

N-(4-hydroxybutyl)-5-methyl-4-oxo-2-thioxo-1,2,3,4-tetrahydrothieno[2,3-d]pyrimidine-6-carboxamide S83

^{1}H-NMR

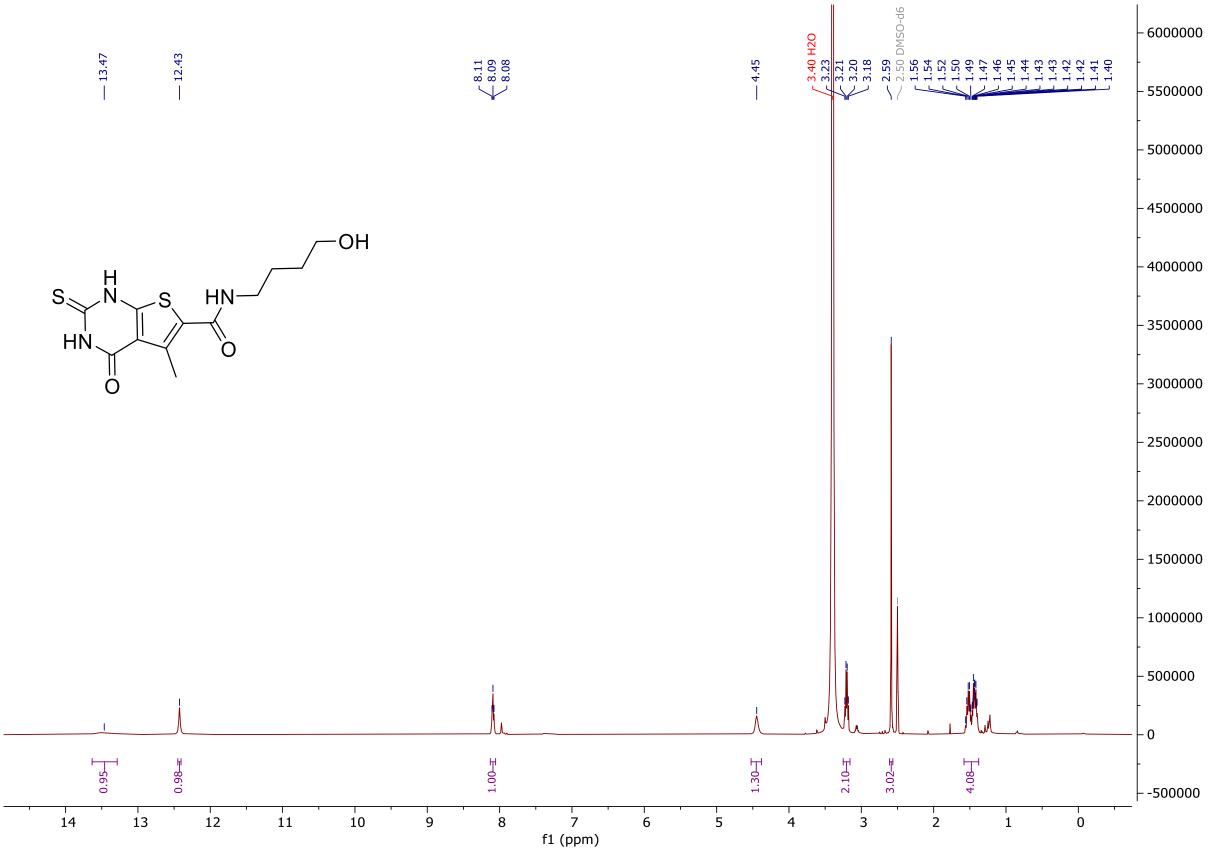

^{13}C-NMR

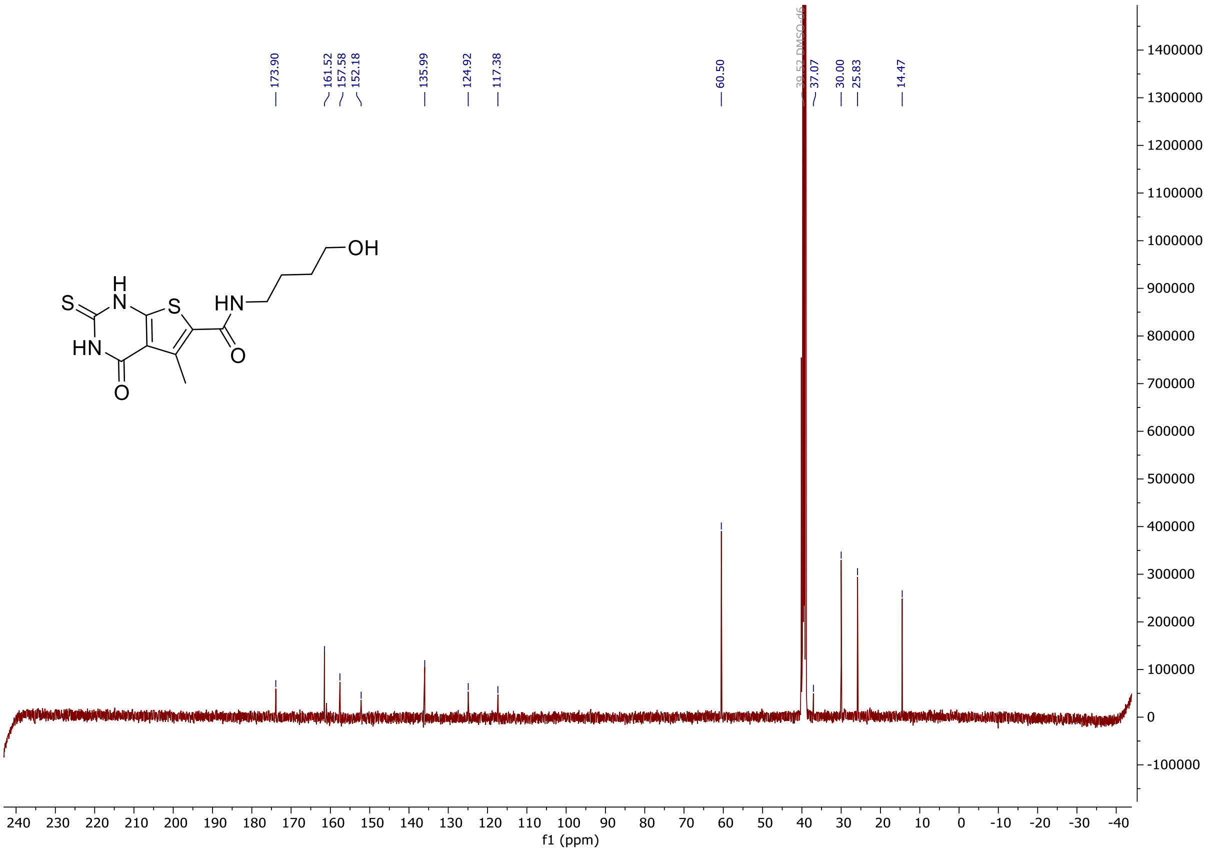

6-phenyl-2-thioxo-2,3-dihydrothieno[2,3-d]pyrimidin-4(1H)-one S84

^{1}H-NMR

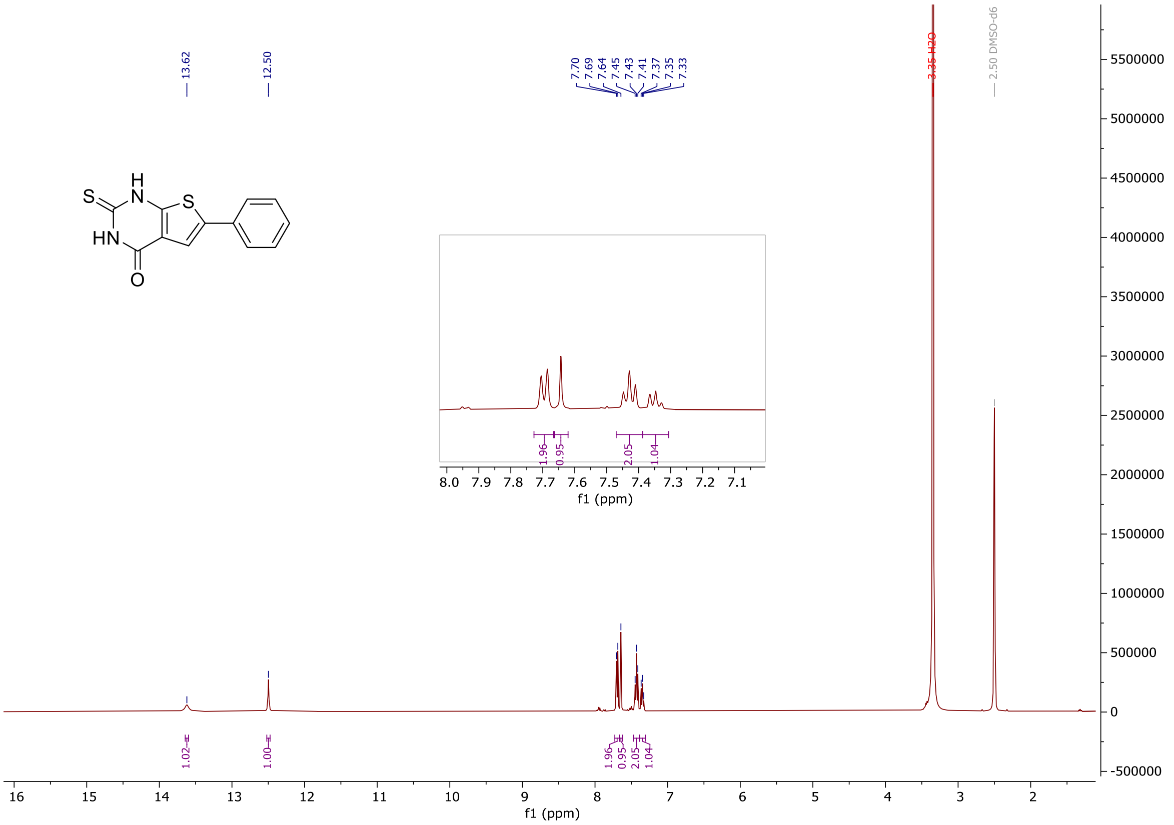

^{13}C-NMR

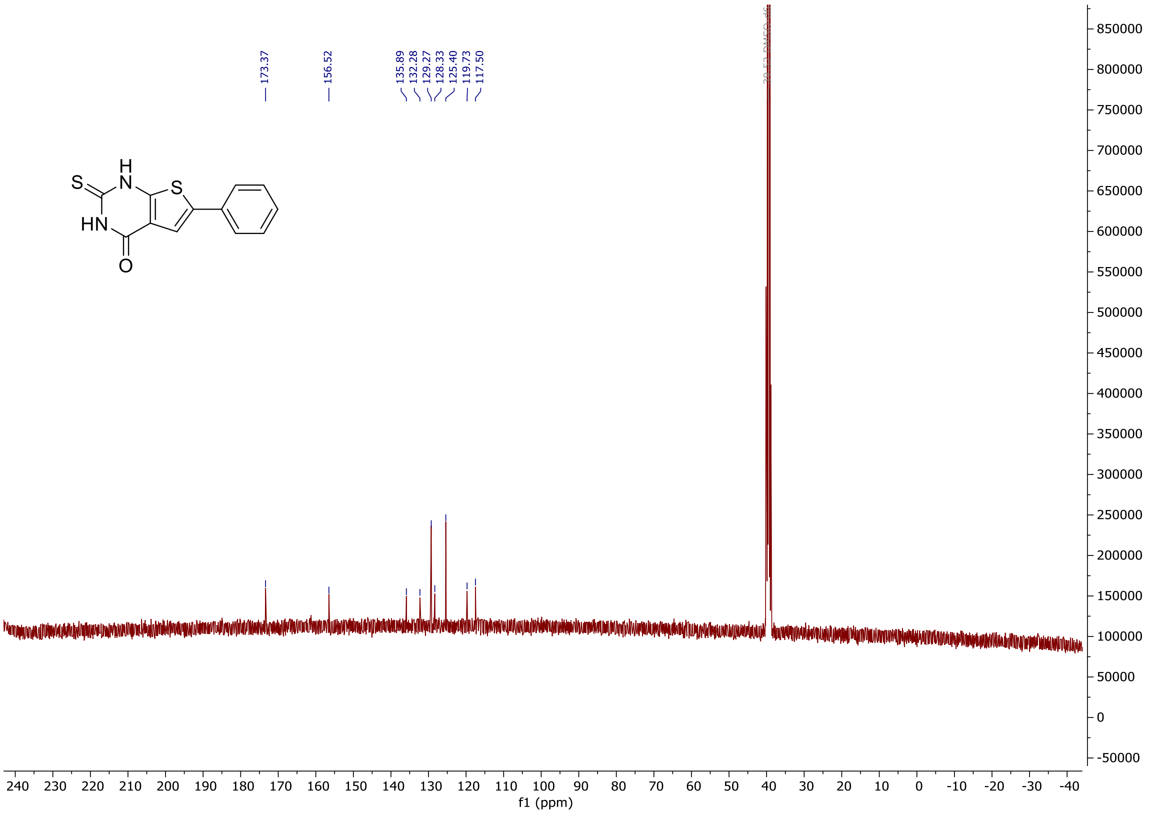

5-methyl-6-(4-nitrophenyl)-2-thioxo-2,3-dihydrothieno[2,3-d]pyrimidin-4(1H)-one 26

^{1}H-NMR

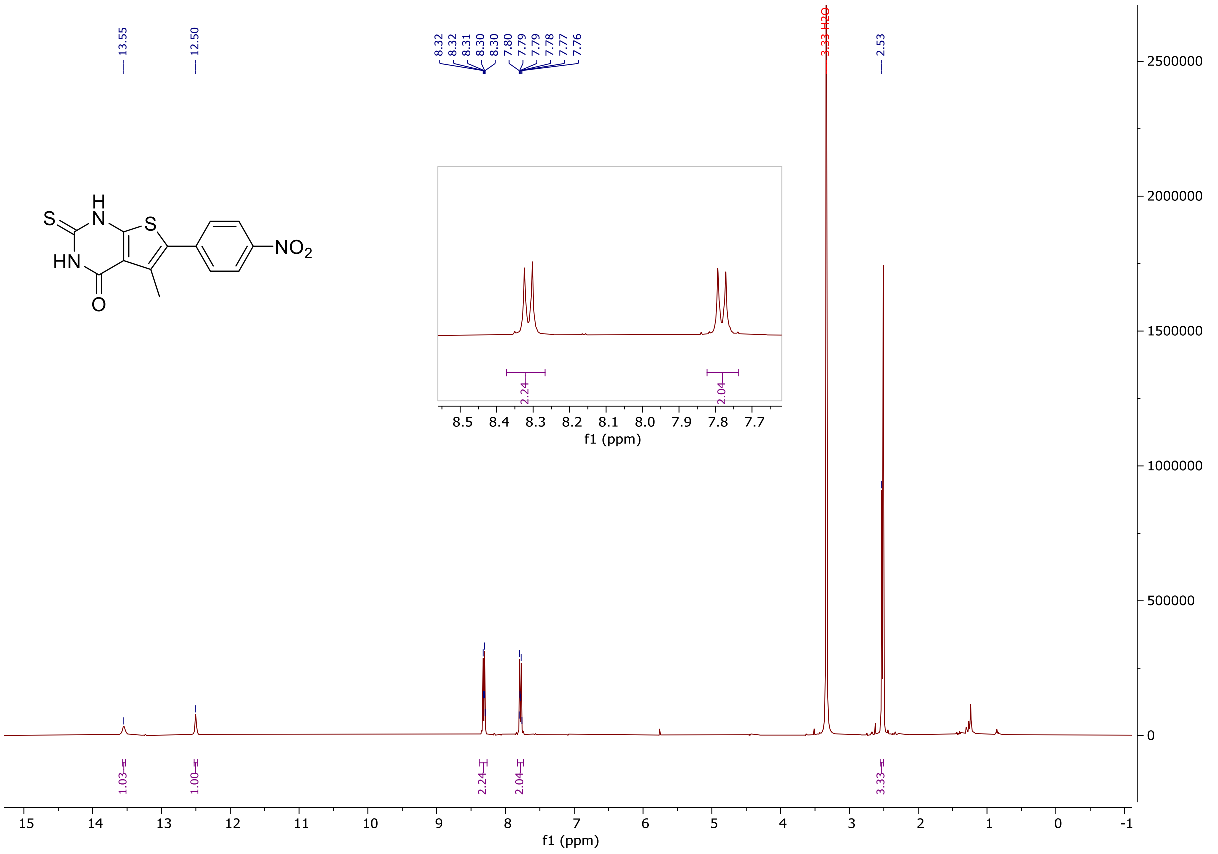

^{13}C-NMR

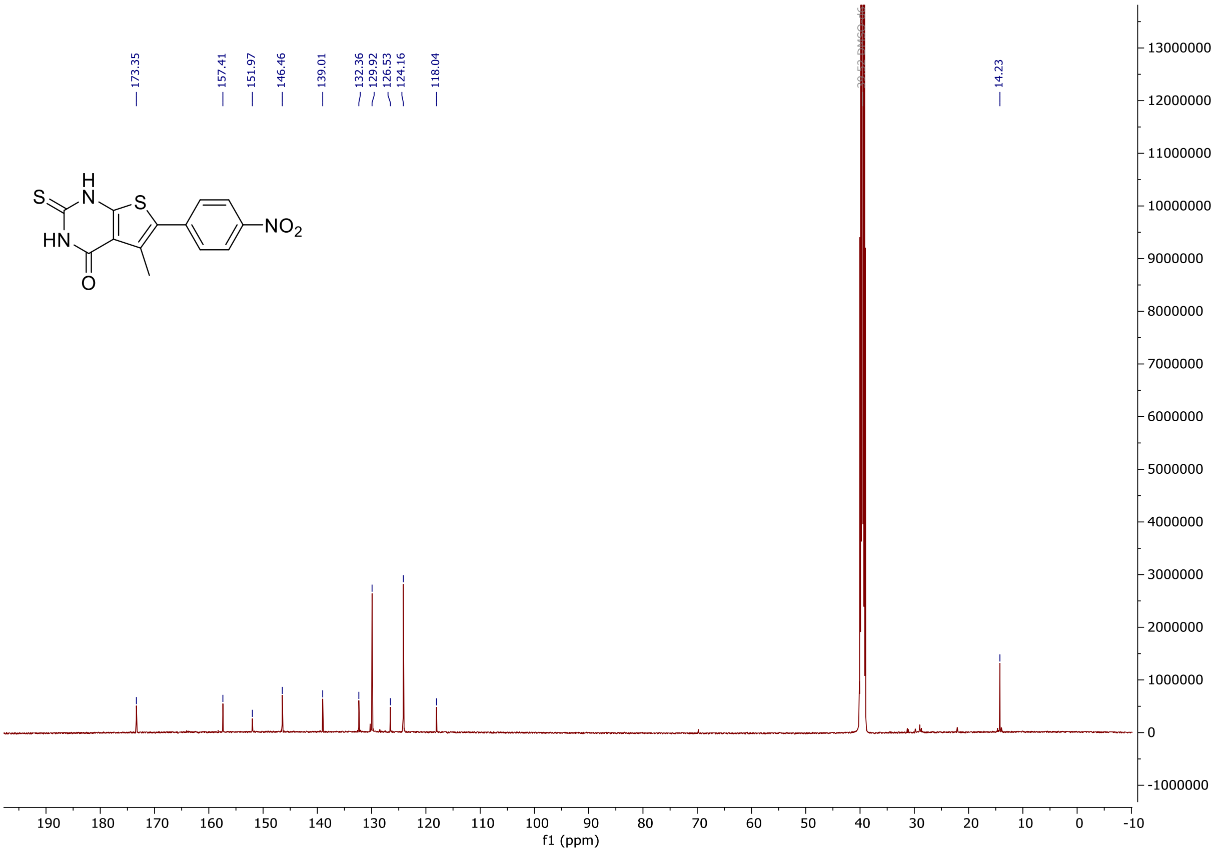

6-(4-aminophenyl)-5-methyl-2-thioxo-2,3-dihydrothieno[2,3-d]pyrimidin-4(1H)-one 27

^{1}H-NMR

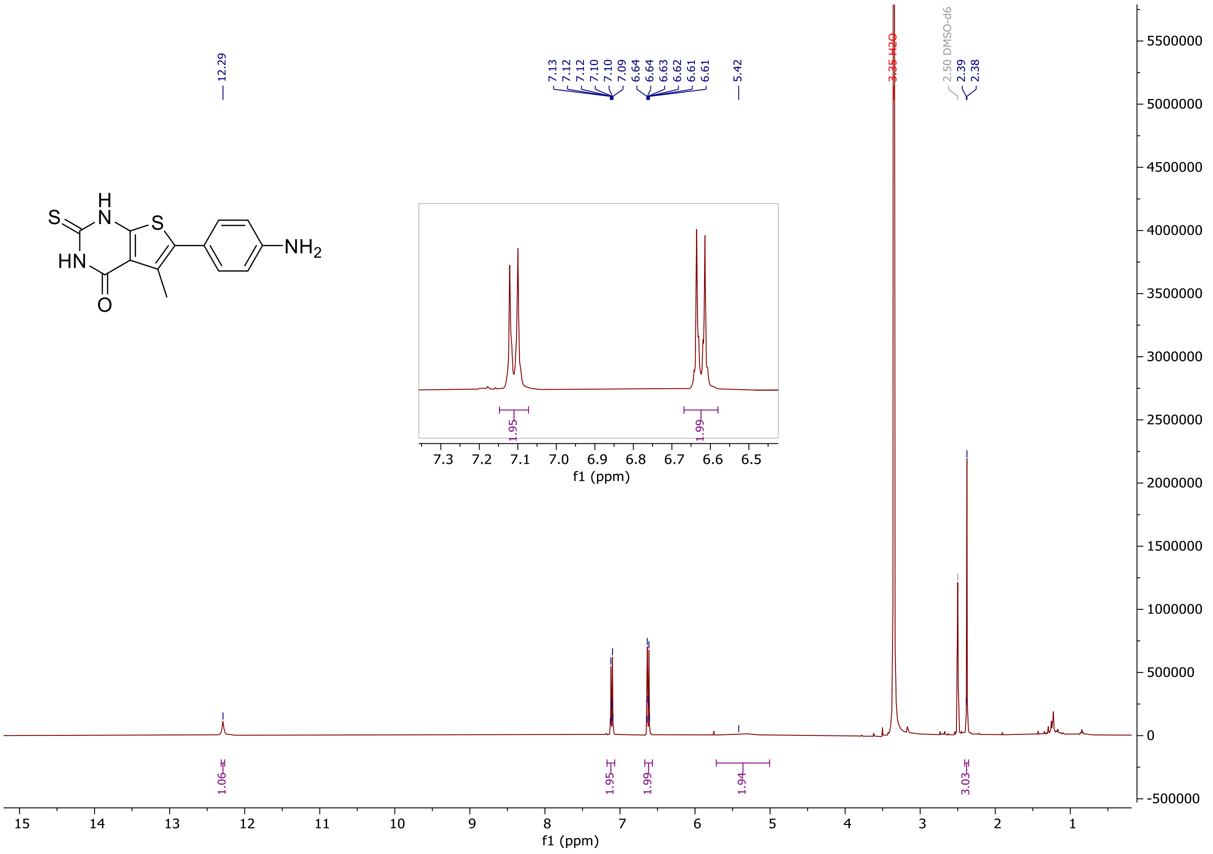

^{13}C-NMR

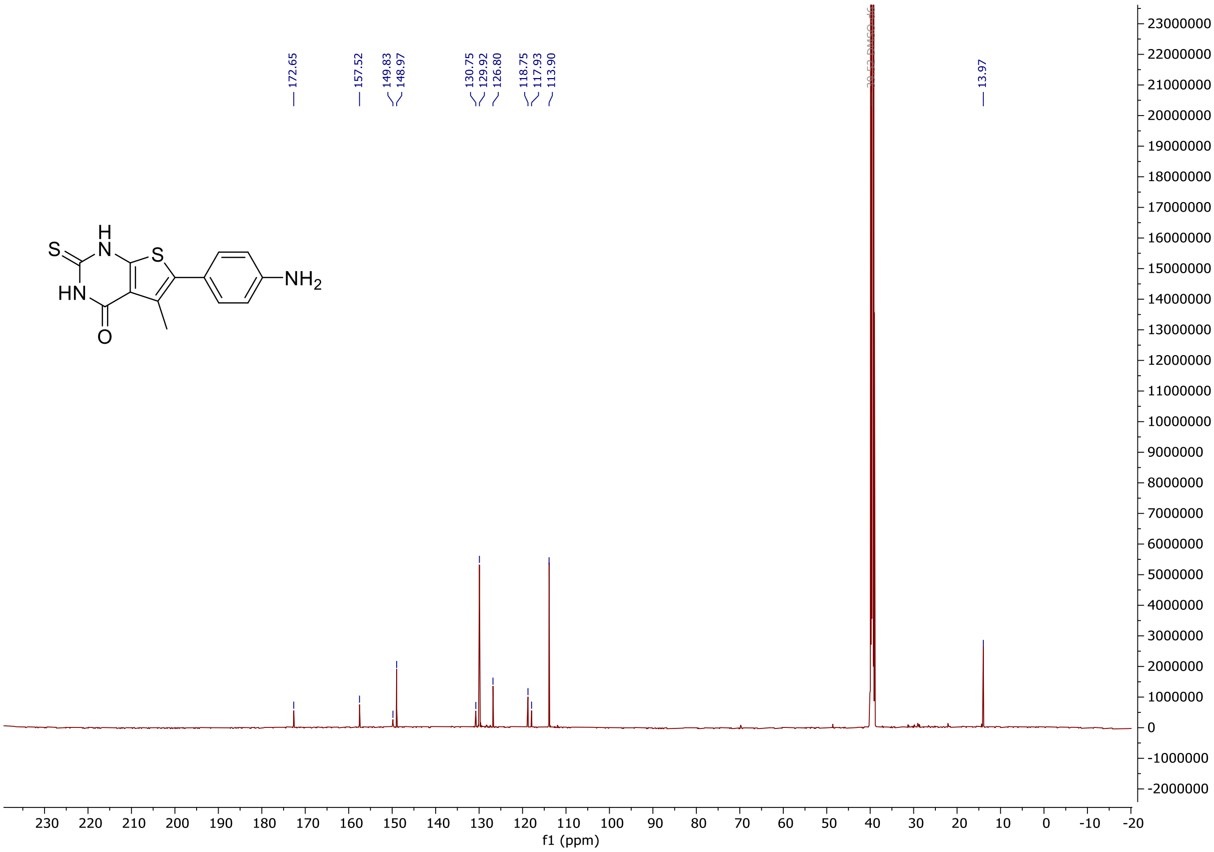

6-(4-methoxyphenyl)-2-thioxo-2,3-dihydrothieno[2,3-d]pyrimidin-4(1H)-one 28

^{1}H-NMR

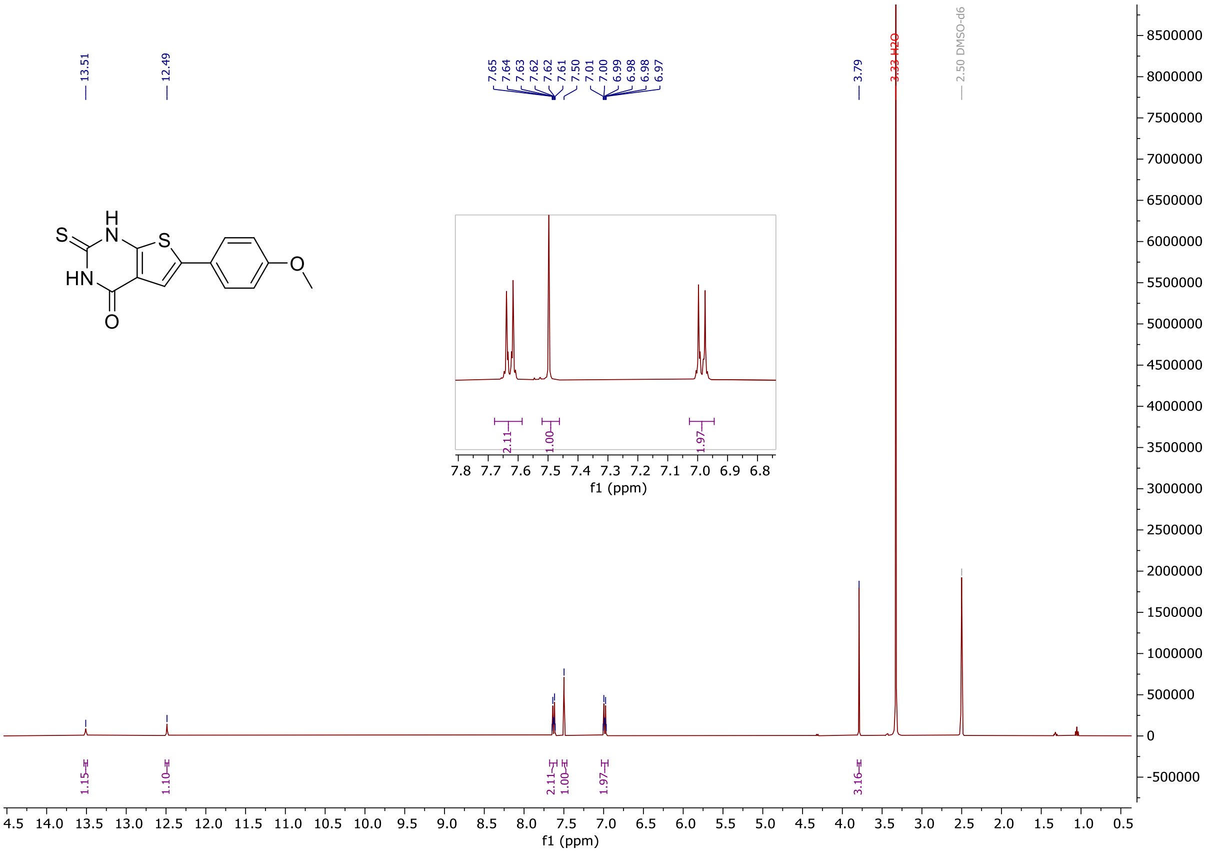

^{13}C-NMR

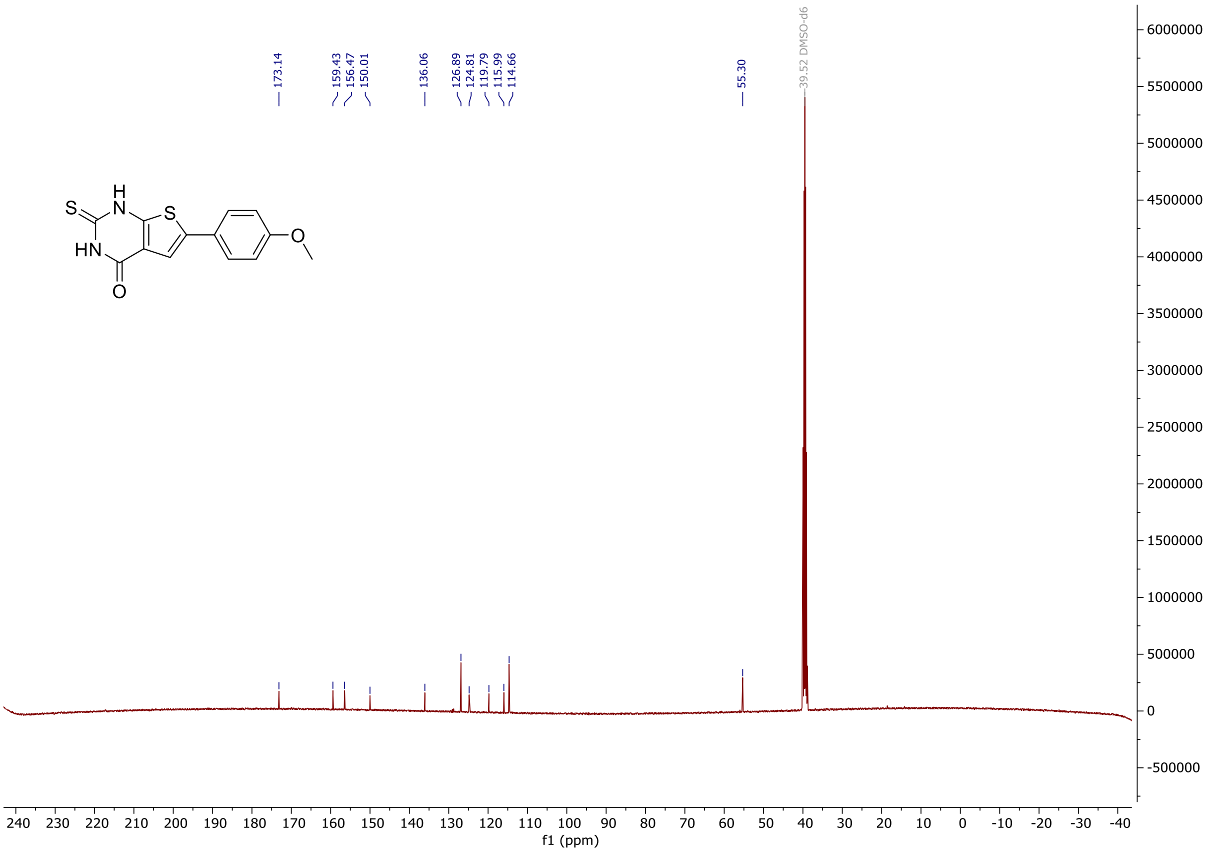

ethyl 4-(4-oxo-2-thioxo-1,2,3,4-tetrahydrothieno[2,3-d]pyrimidin-6-yl)benzoate 29

^{1}H-NMR

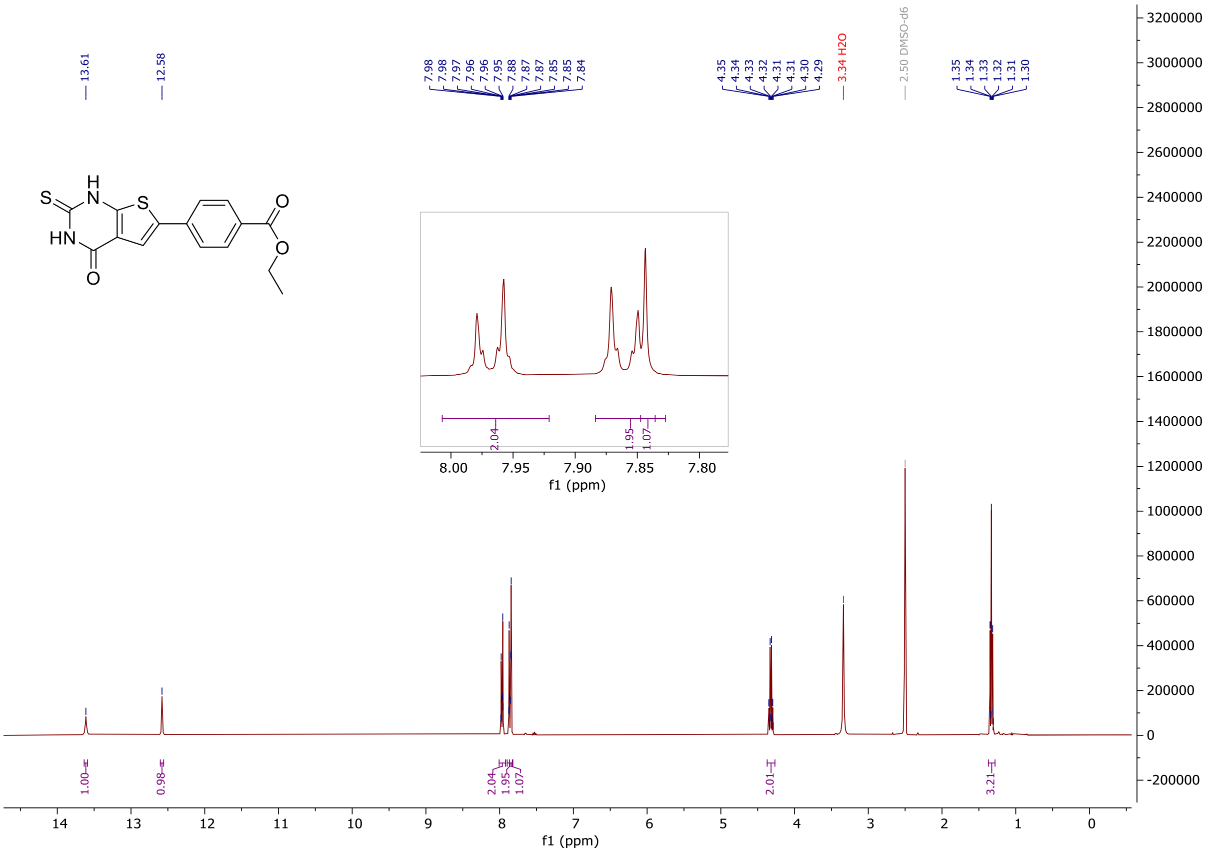

^{13}C-NMR

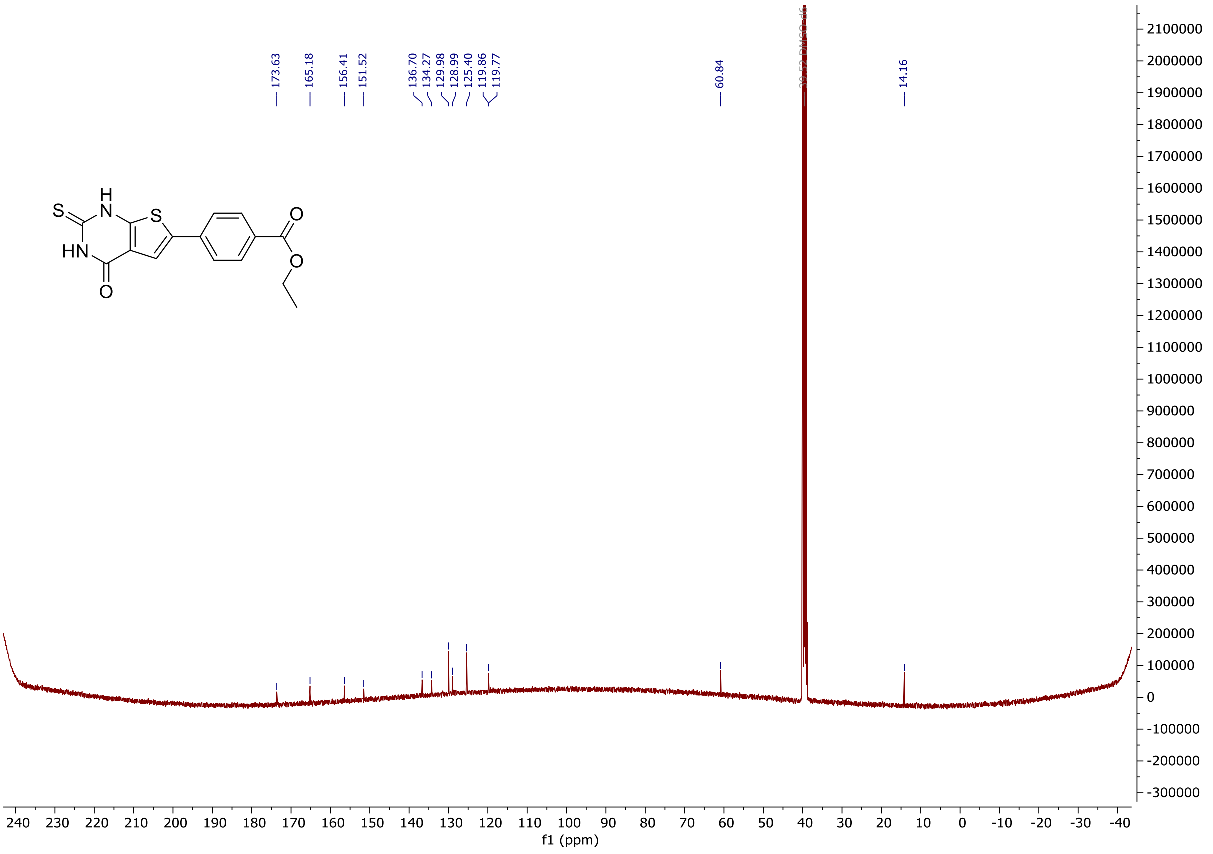

4-(4-oxo-2-thioxo-1,2,3,4-tetrahydrothieno[2,3-d]pyrimidin-6-yl)benzoic acid 30

^{1}H-NMR

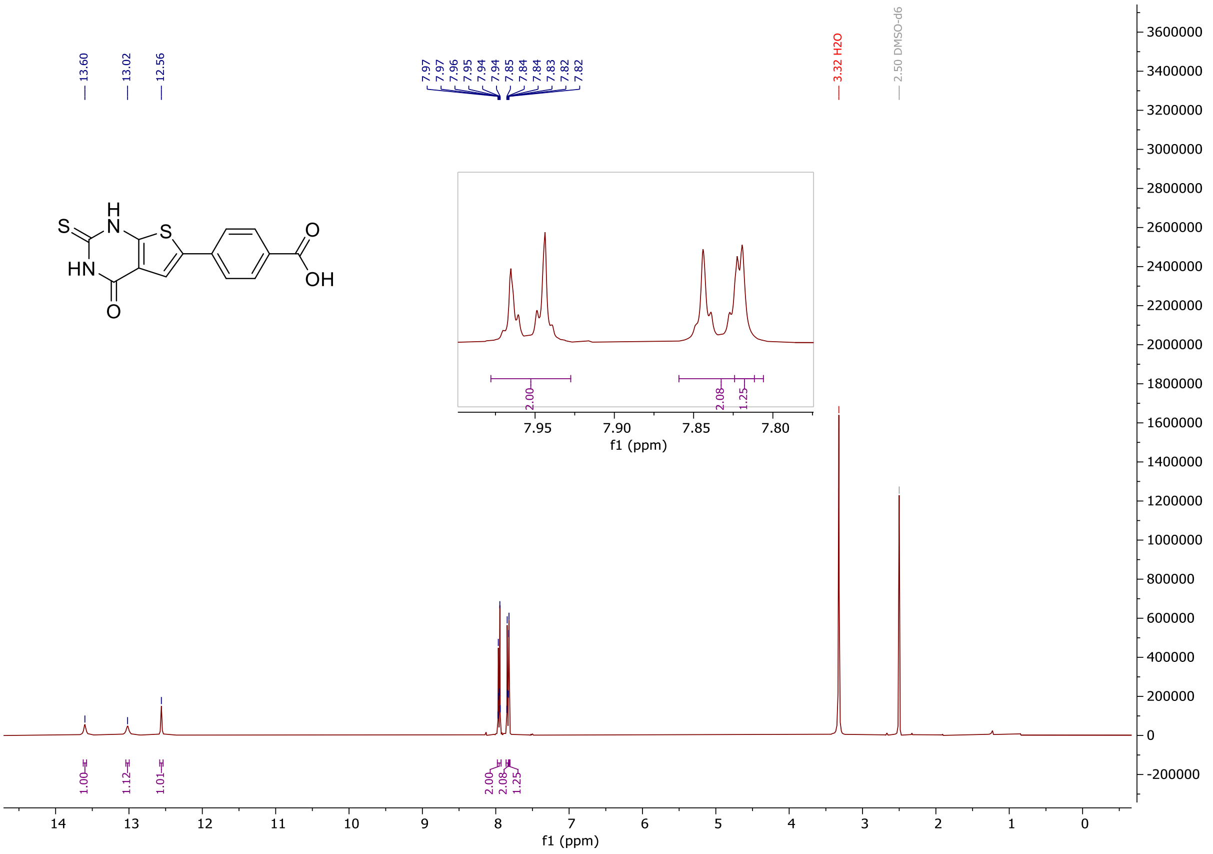

^{13}C-NMR

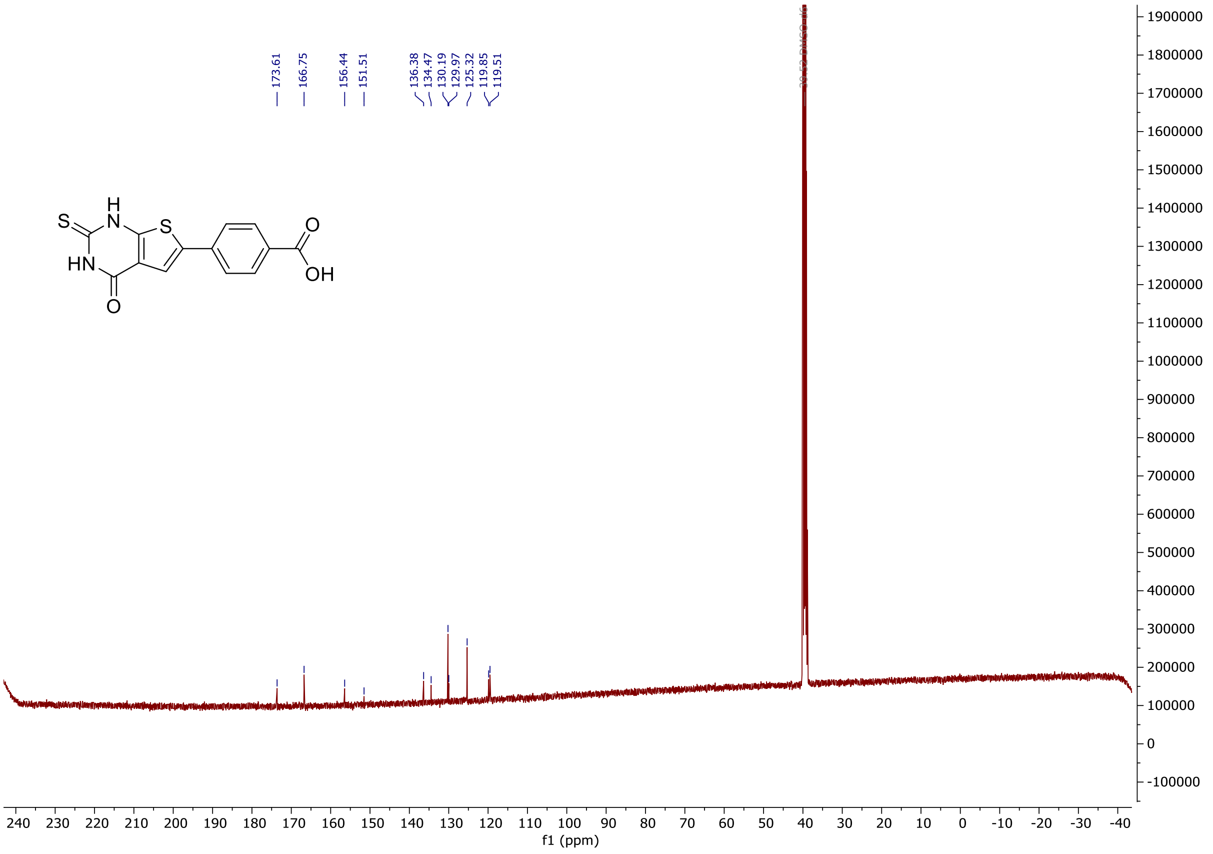

6-benzyl-2-thioxo-2,3-dihydrothieno[2,3-d]pyrimidin-4(1H)-one S85

^{1}H-NMR

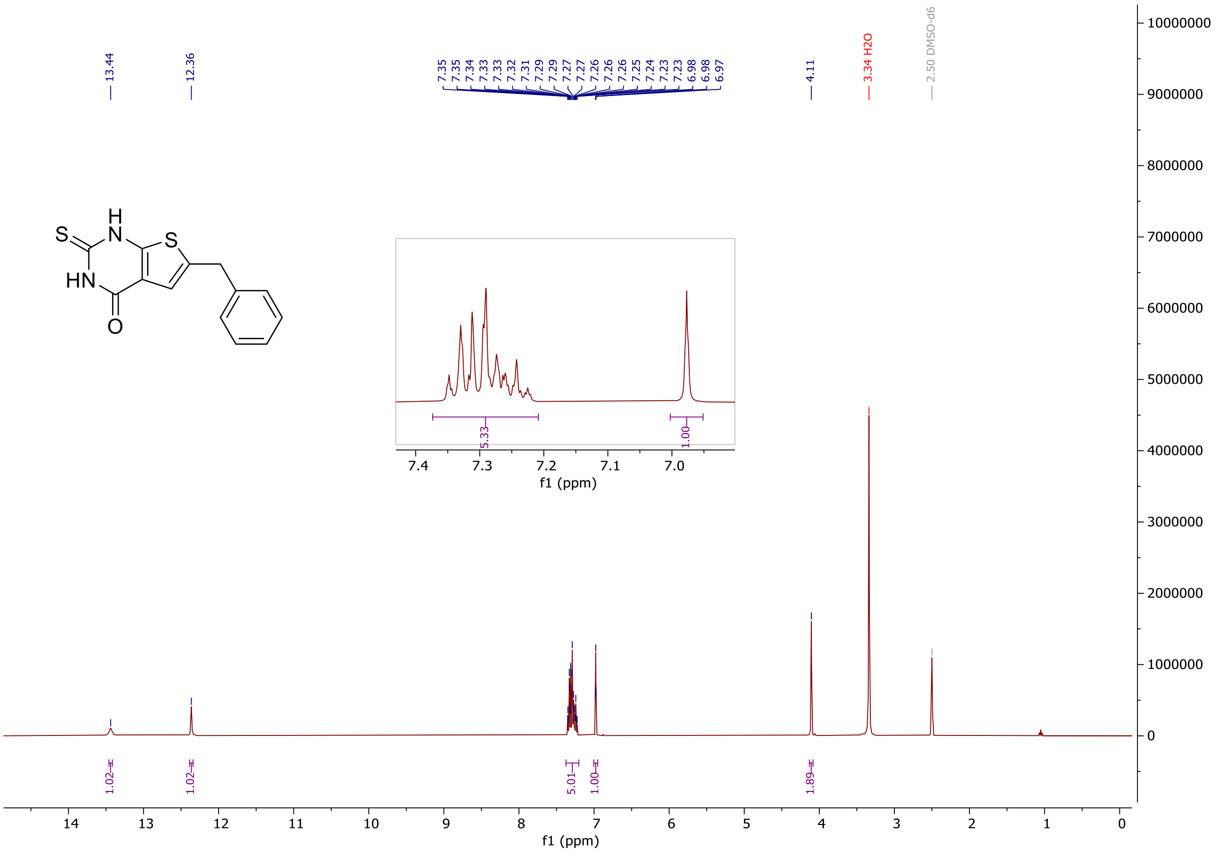

^{13}C-NMR

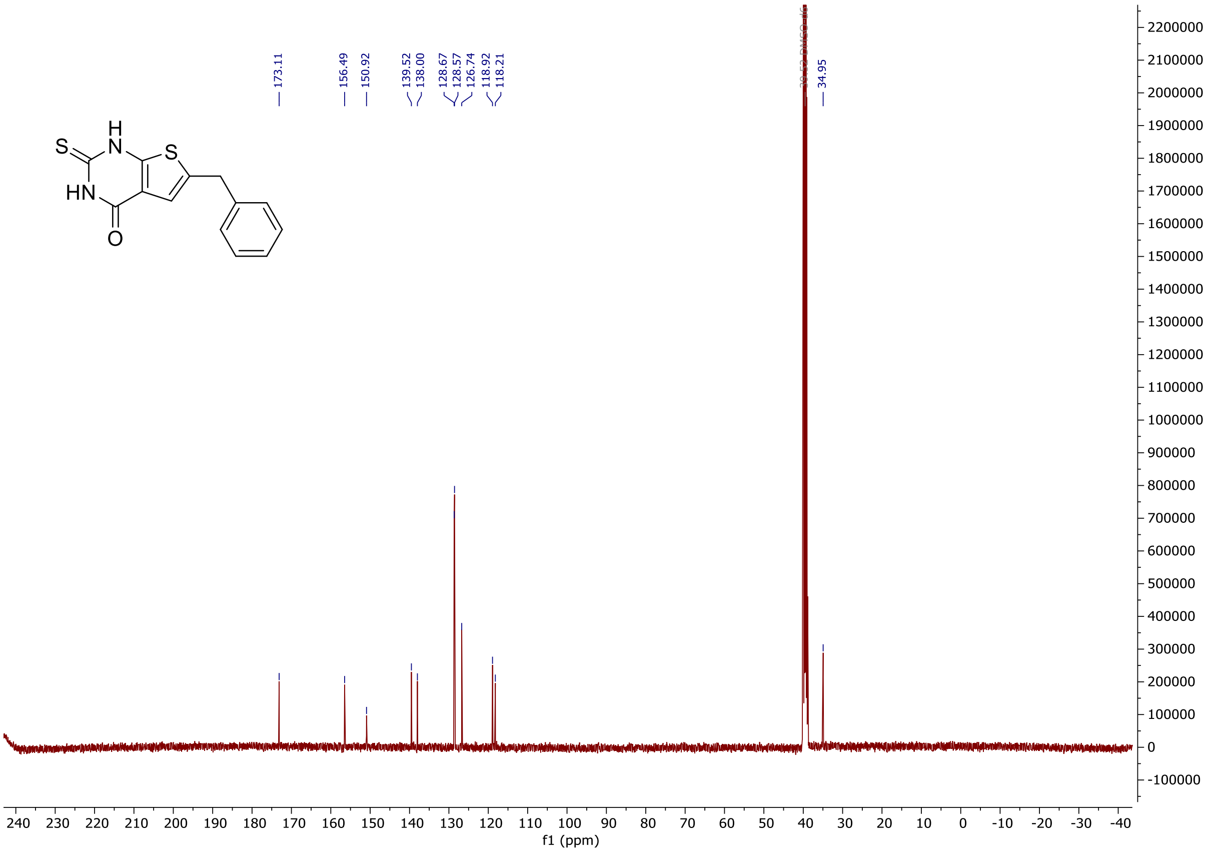

ethyl 4-((4-oxo-2-thioxo-1,2,3,4-tetrahydrothieno[2,3-d]pyrimidin-6-yl)methyl)benzoate S86

^{1}H-NMR

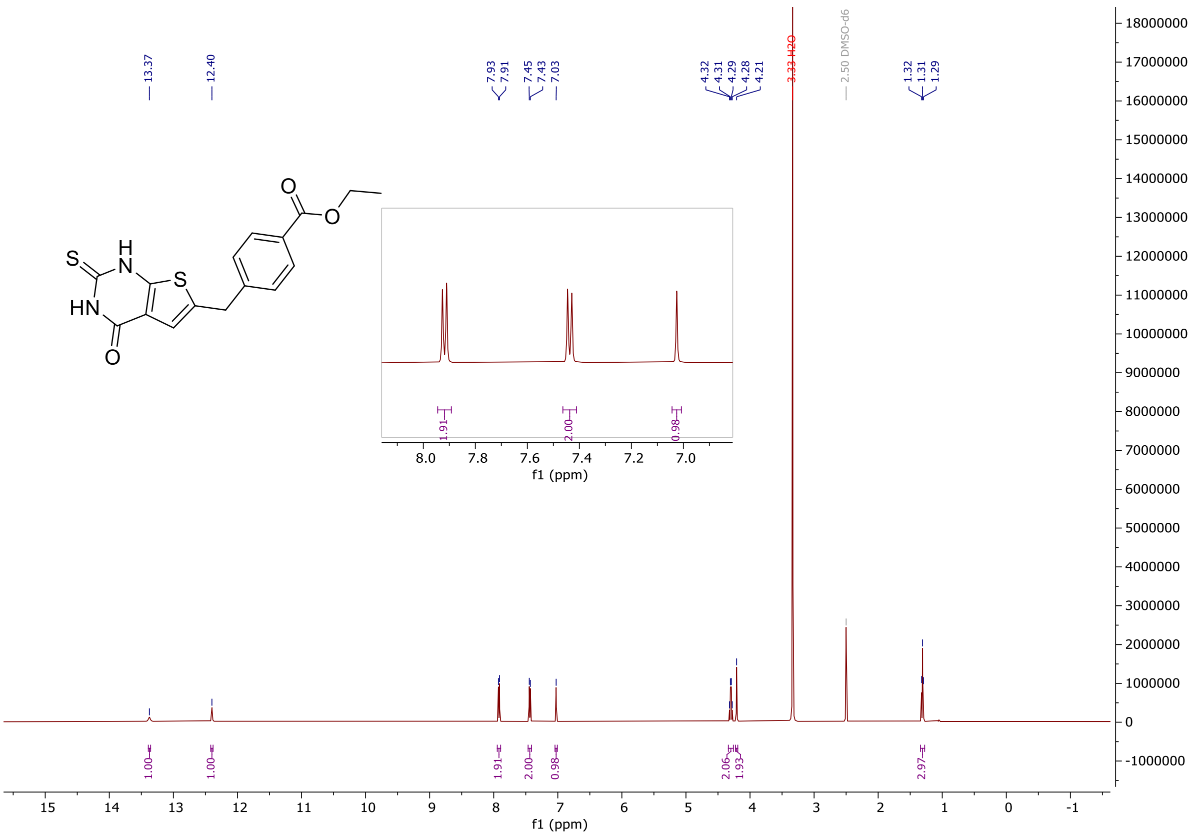

^{13}C-NMR

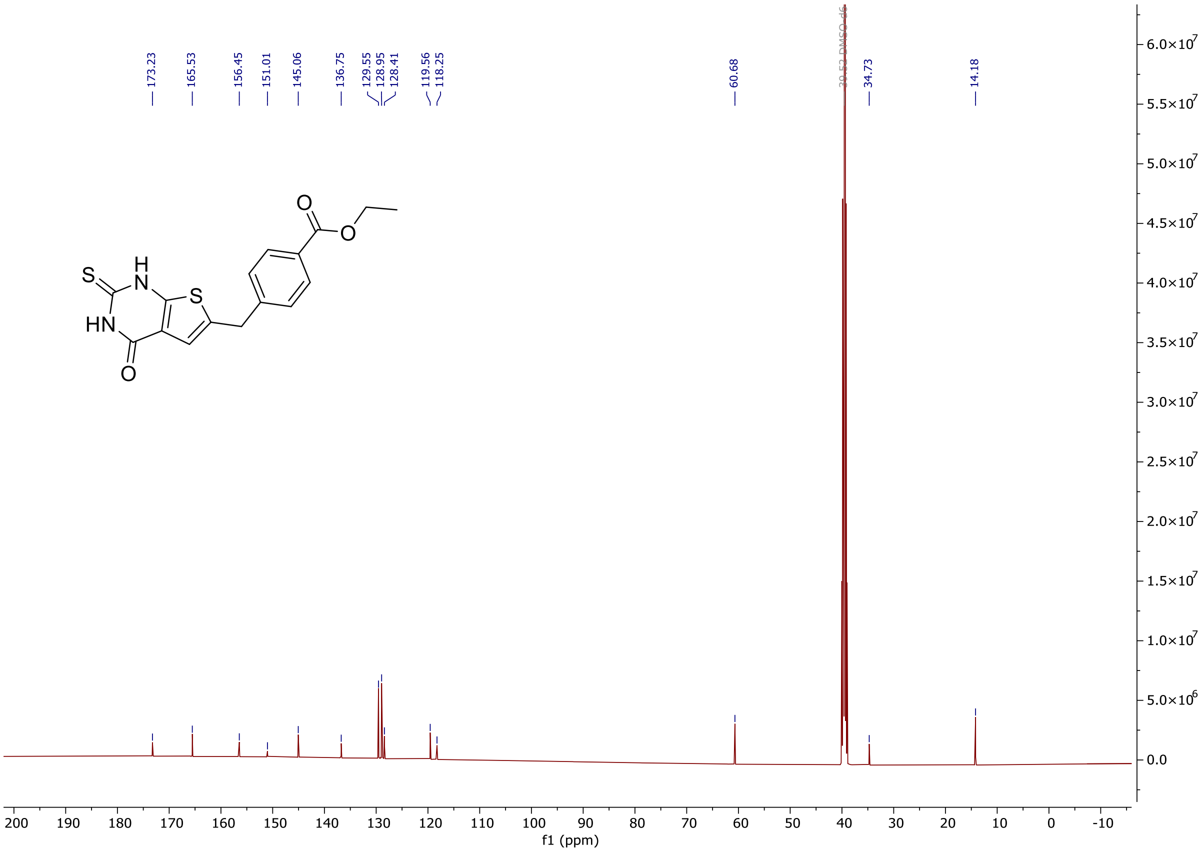

4-((4-oxo-2-thioxo-1,2,3,4-tetrahydrothieno[2,3-d]pyrimidin-6-yl)methyl)benzoic acid S87

^{1}H-NMR

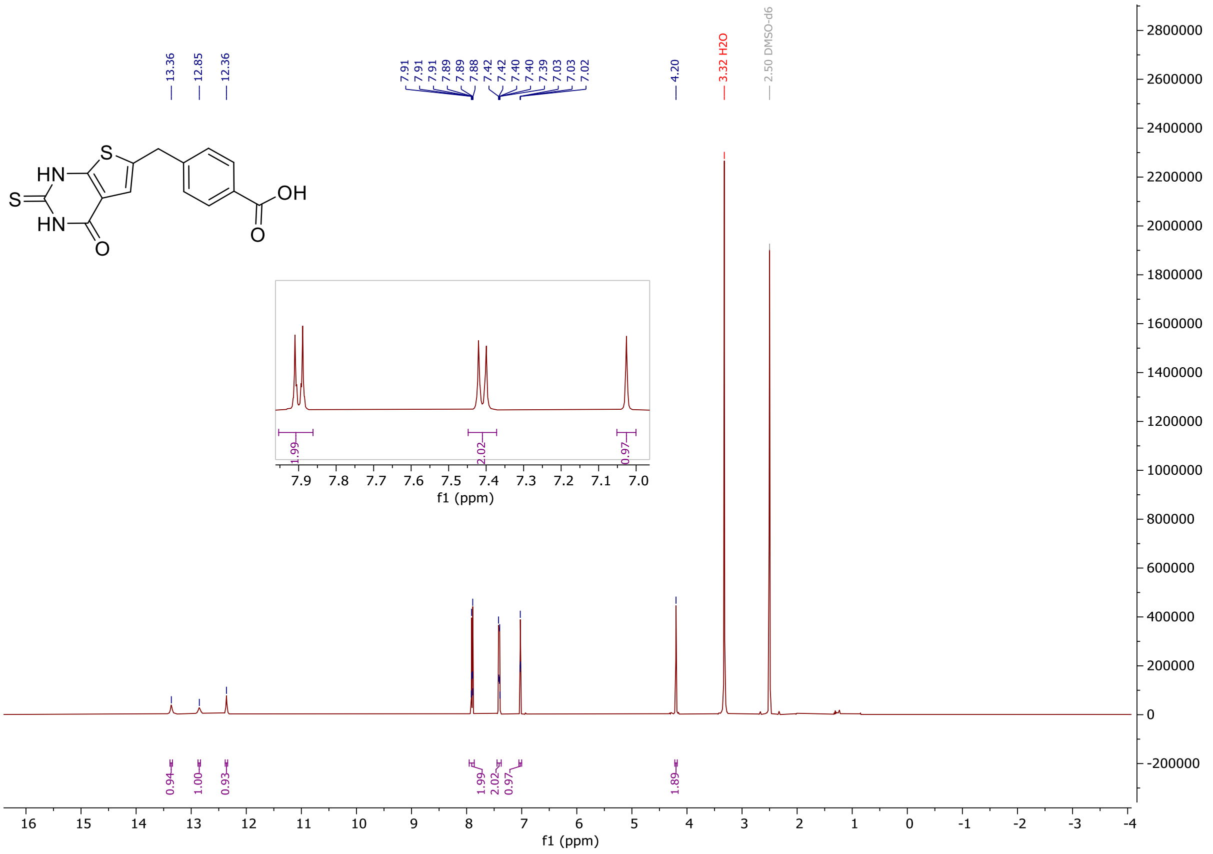

^{13}C-NMR

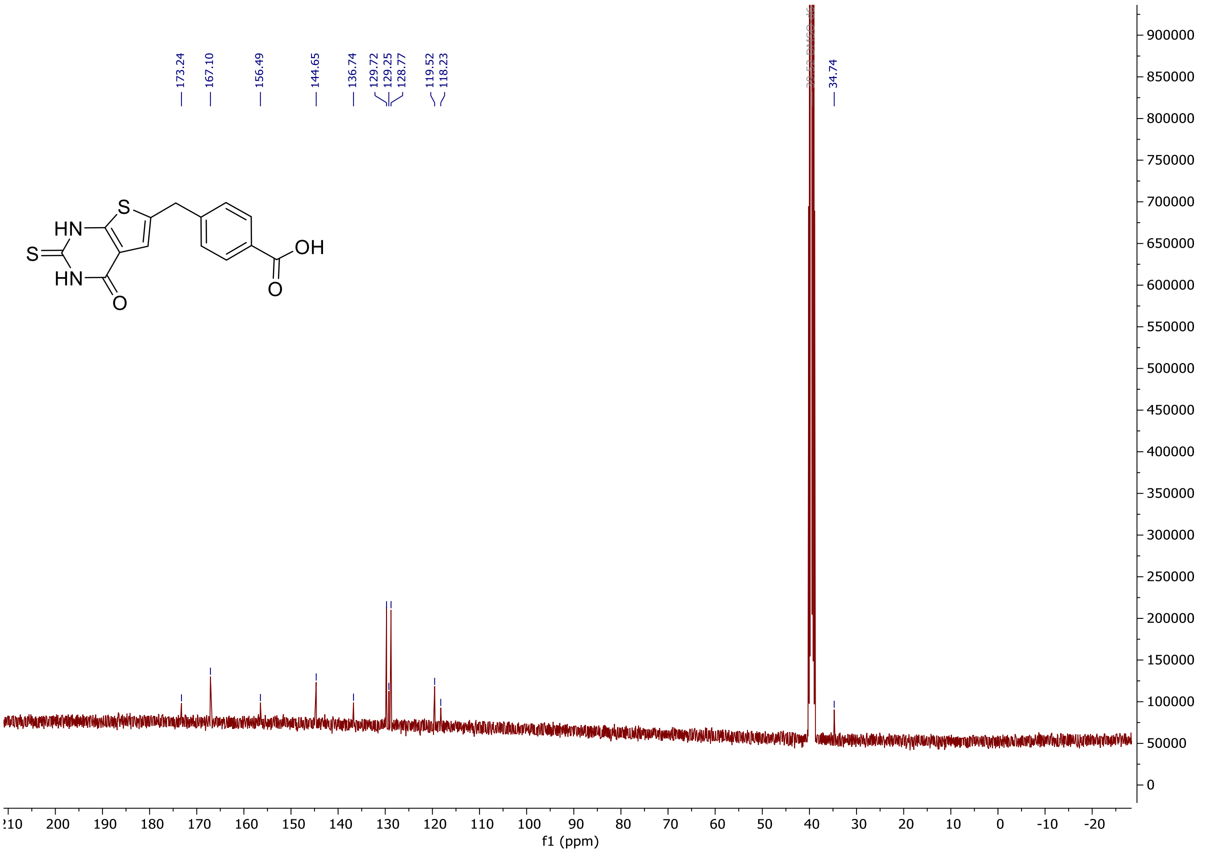

5-methyl-2-thioxo-2,3-dihydrothieno[2,3-d]pyrimidin-4(1H)-one S88

^{1}H-NMR

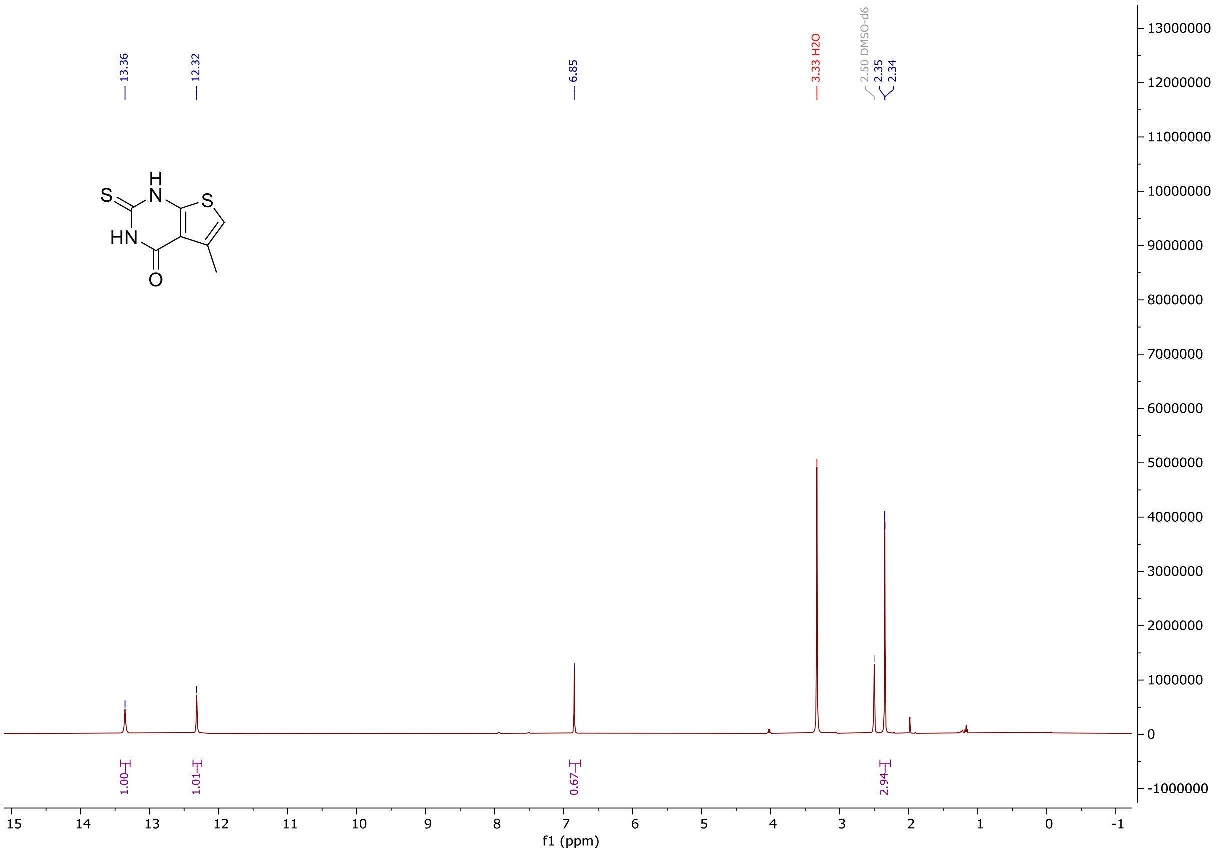

^{13}C-NMR

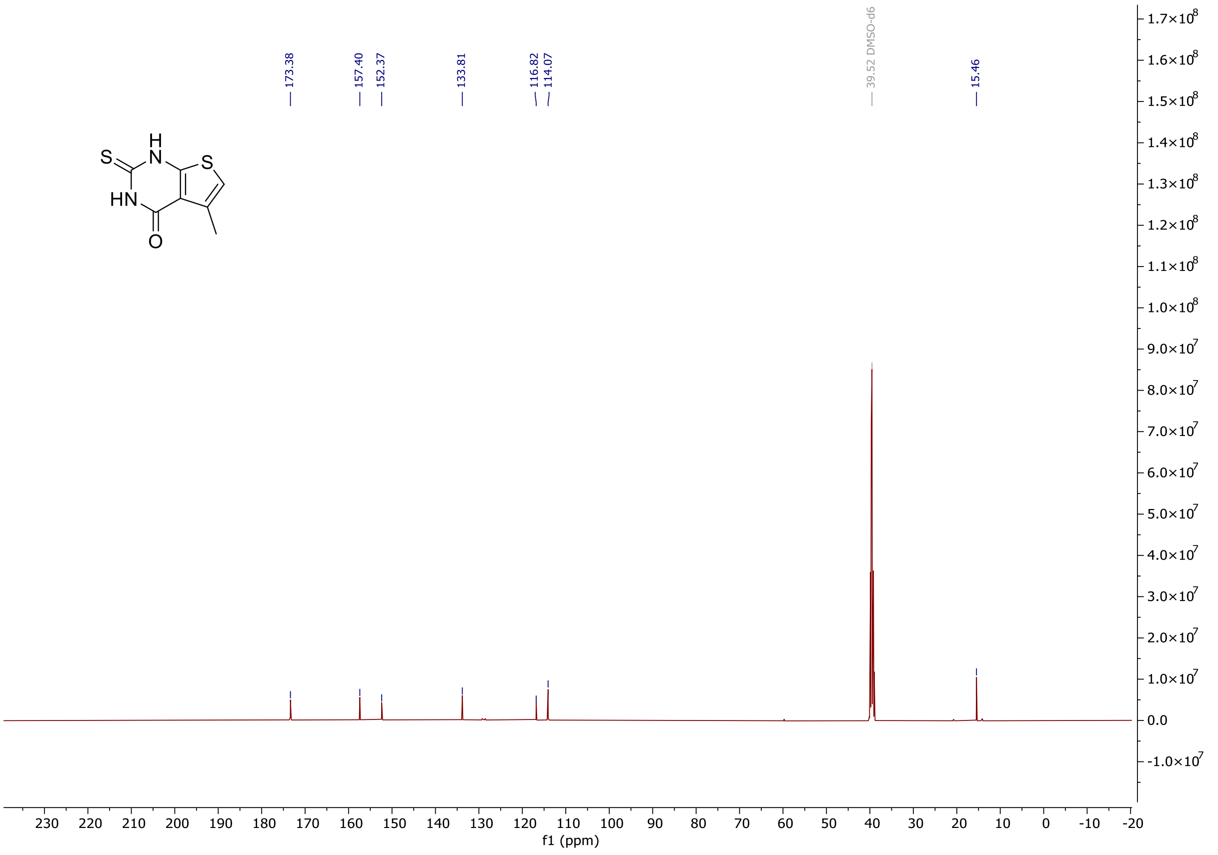

ethyl 4-oxo-2-thioxo-1,2,3,4-tetrahydrothieno[2,3-d]pyrimidine-5-carboxylate S89

^{1}H-NMR

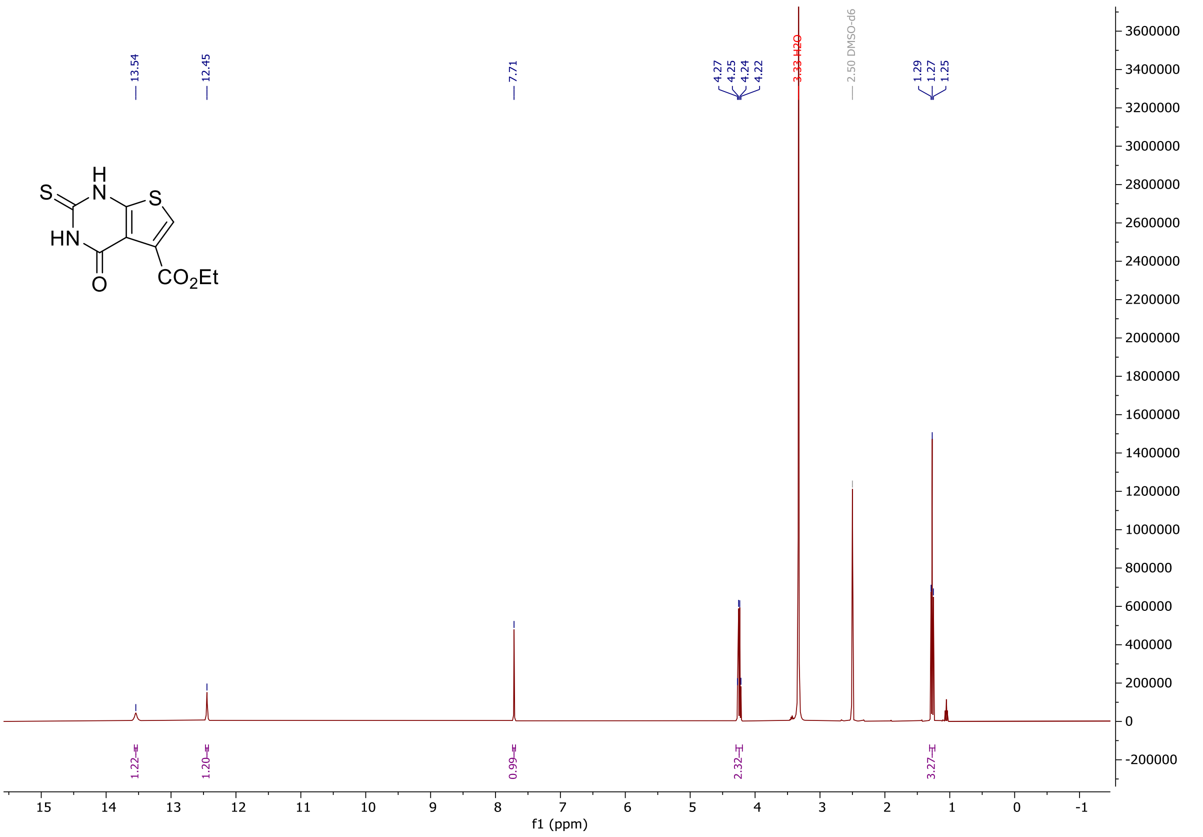

^{13}C-NMR

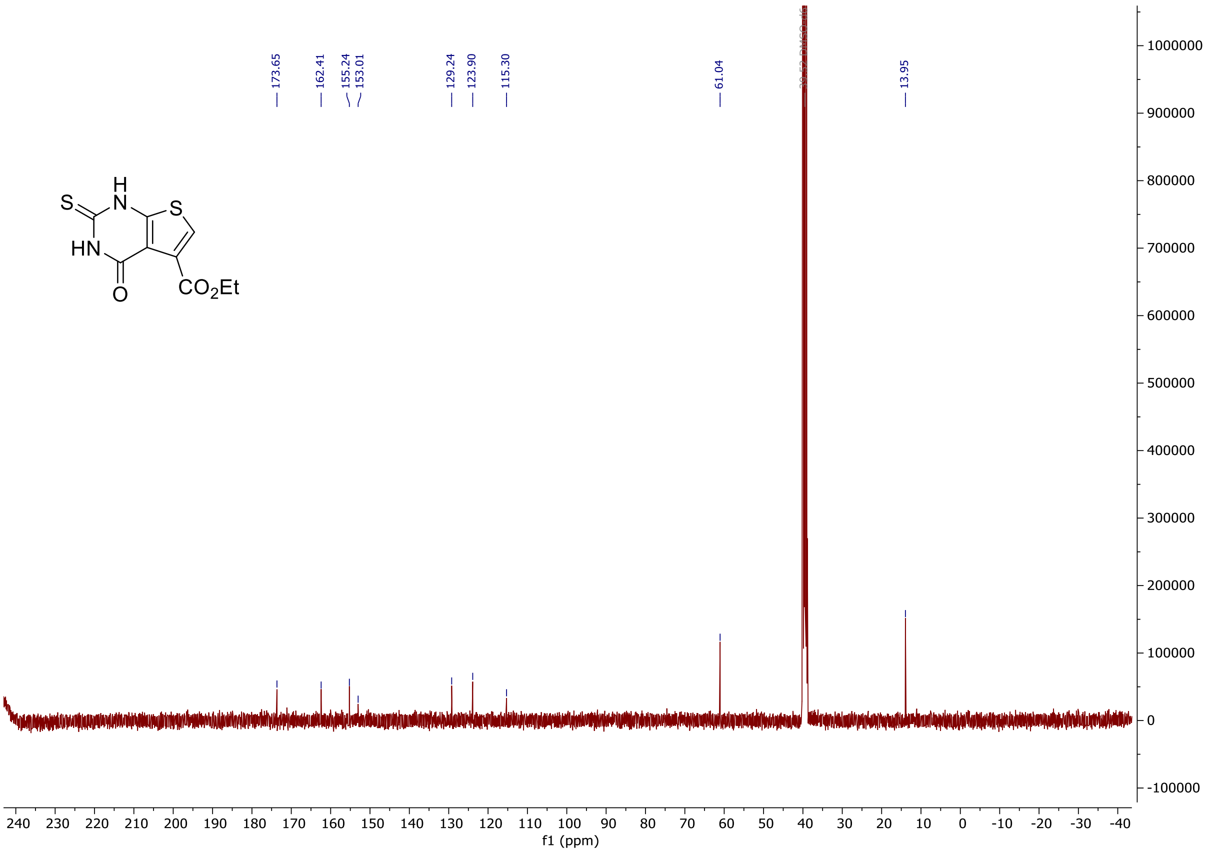

4-oxo-2-thioxo-1,2,3,4-tetrahydrothieno[2,3-d]pyrimidine-5-carboxylic acid S90

^{1}H-NMR

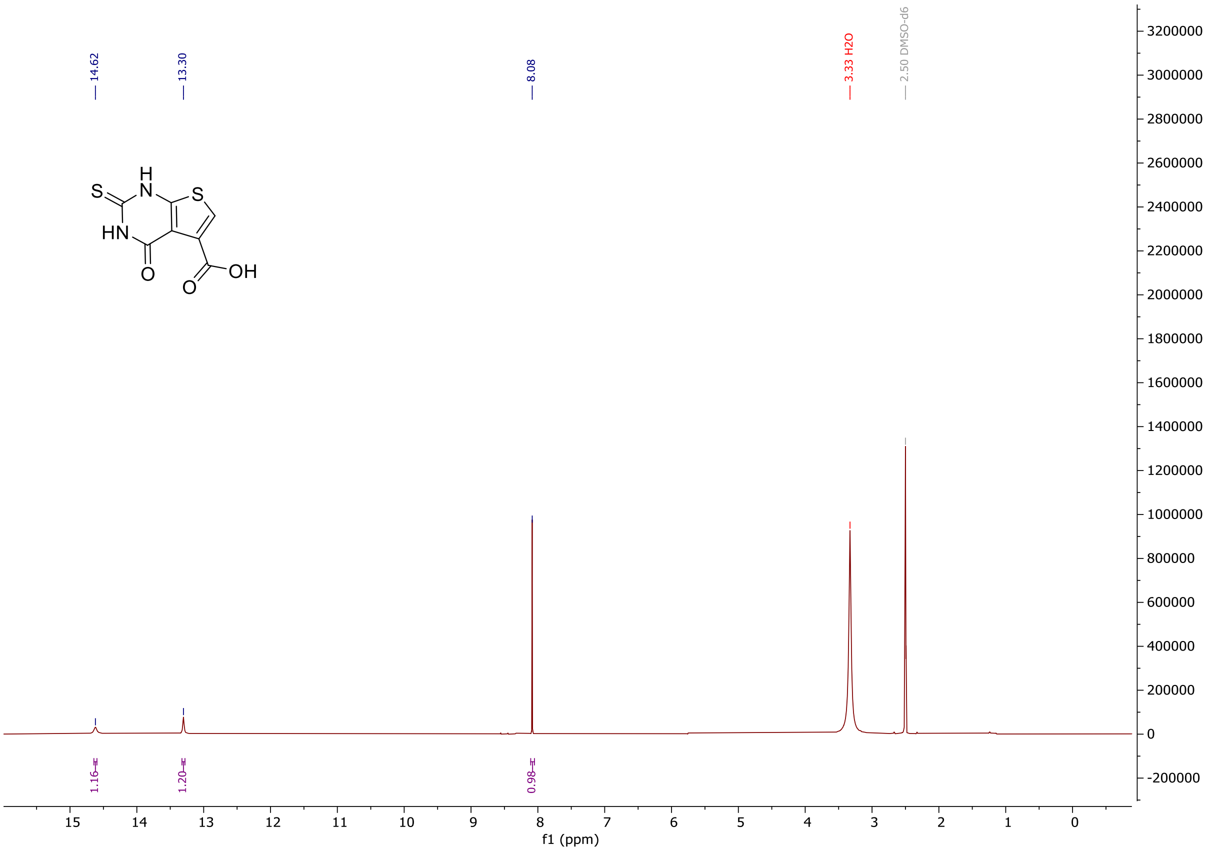

^{13}C-NMR

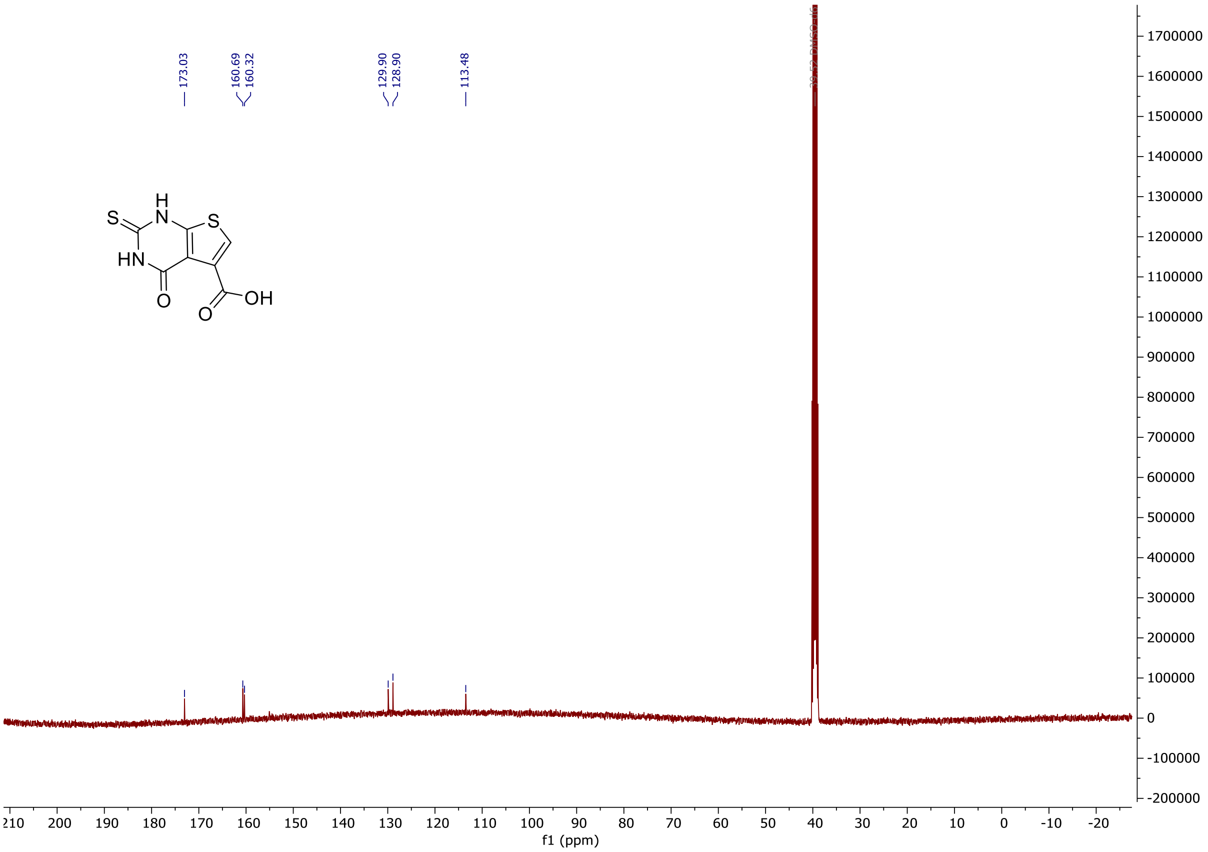

4-oxo-2-thioxo-1,2,3,4-tetrahydrothieno[2,3-d]pyrimidine-5-carboxamide 31

^{1}H-NMR

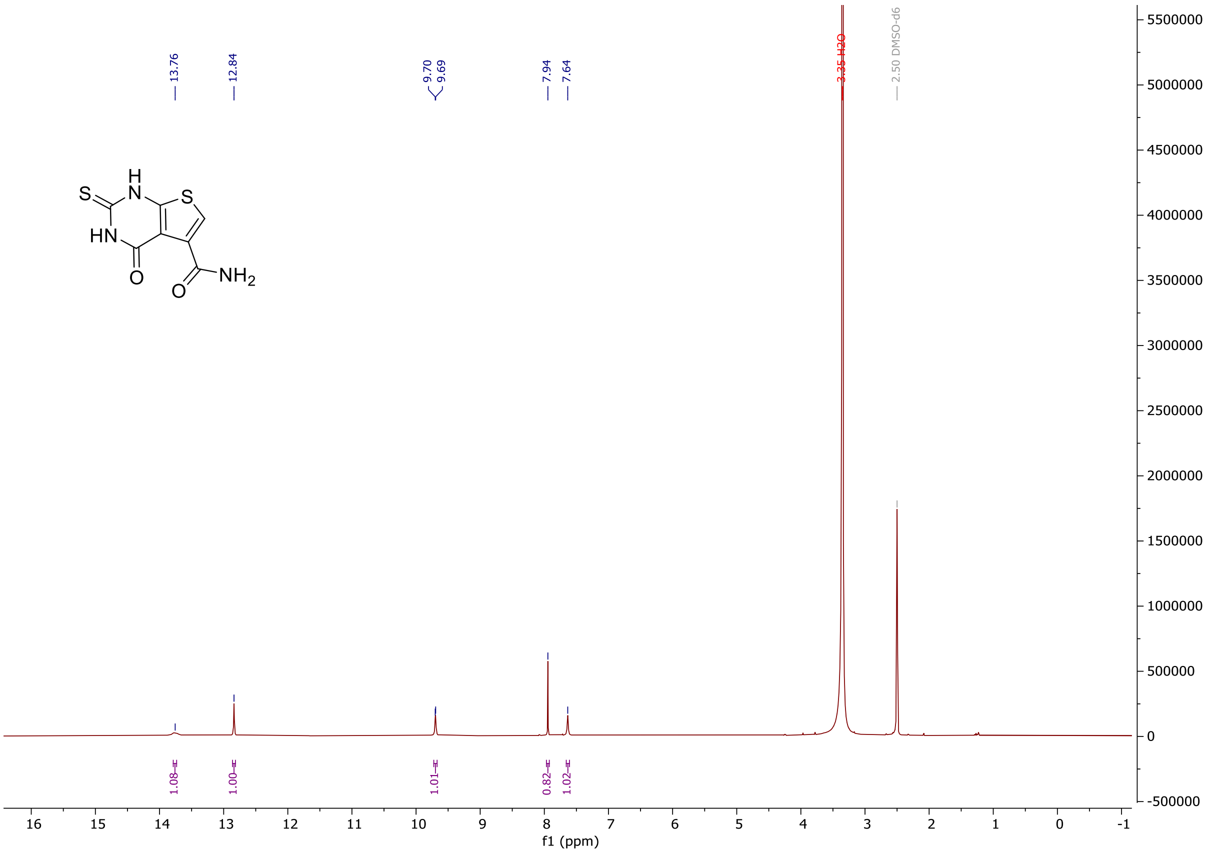

^{13}C-NMR

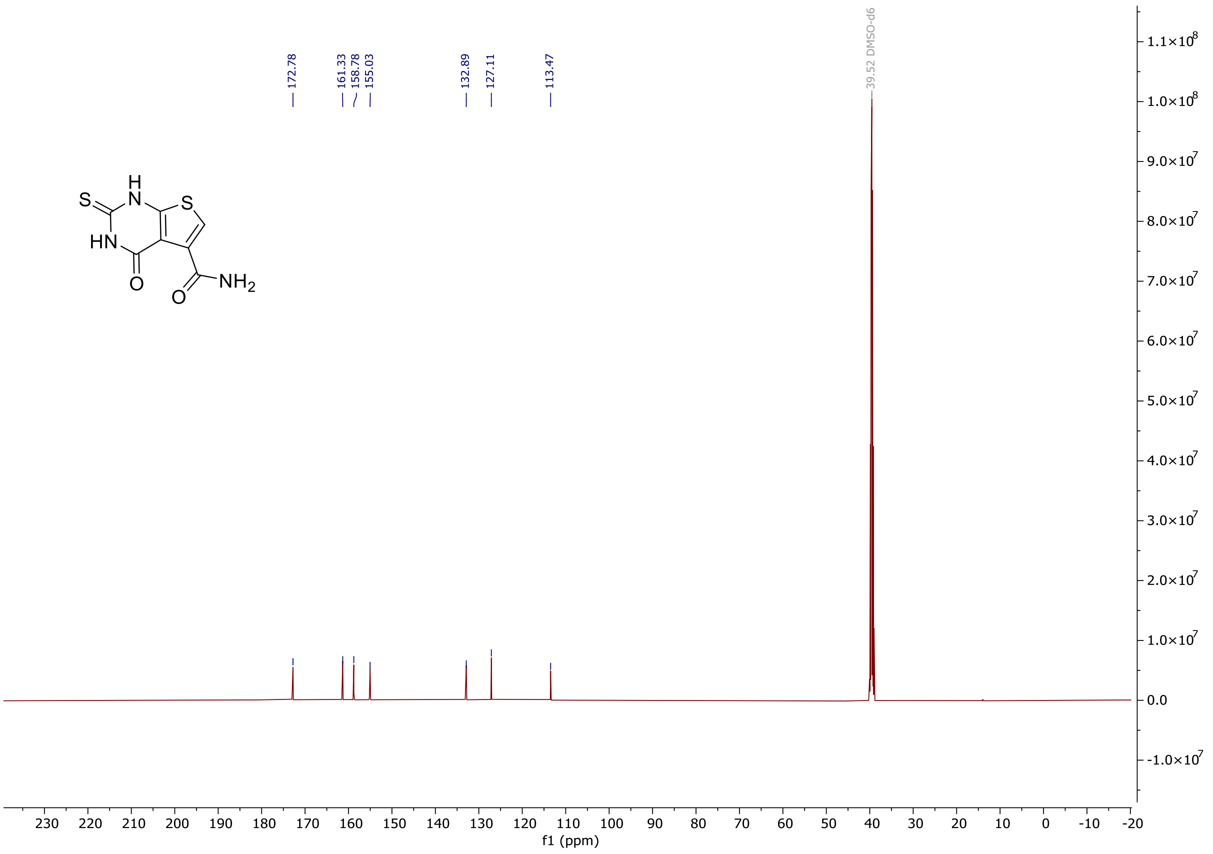

HPLC TRACES OF FINAL COMPOUNDS

5-phenyl-2-thioxo-2,3-dihydrothieno[2,3-d]pyrimidin-4(1H)-one S74

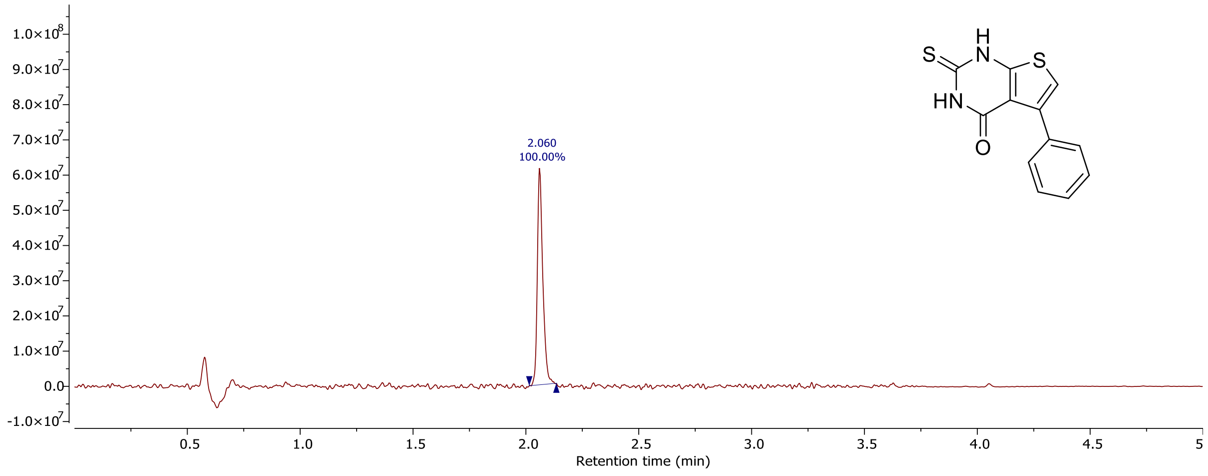

methyl 4-(4-oxo-2-thioxo-1,2,3,4-tetrahydrothieno[2,3-d]pyrimidin-5-yl)benzoate 17

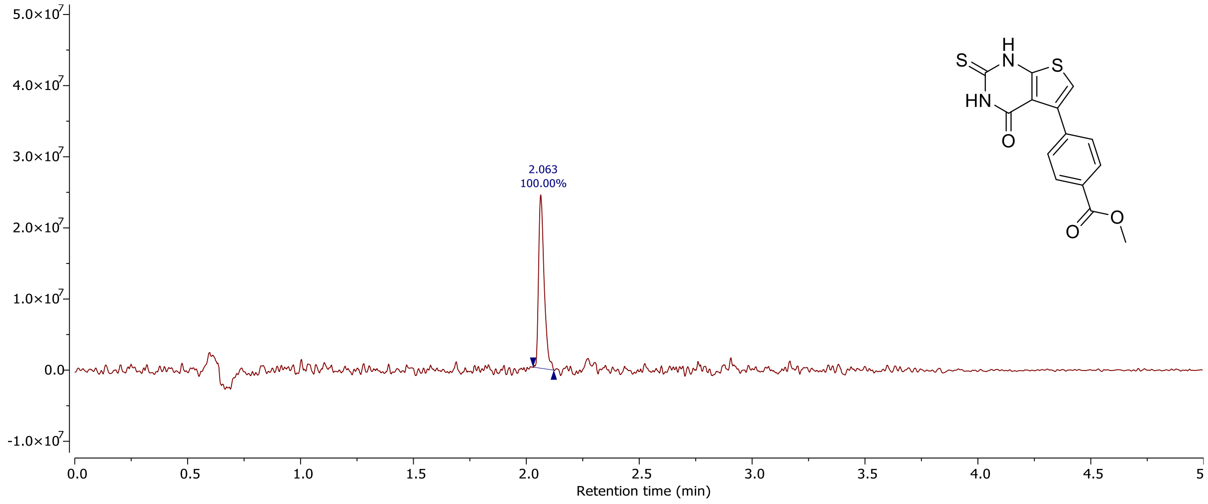

4-(4-oxo-2-thioxo-1,2,3,4-tetrahydrothieno[2,3-d]pyrimidin-5-yl)benzoic acid 18

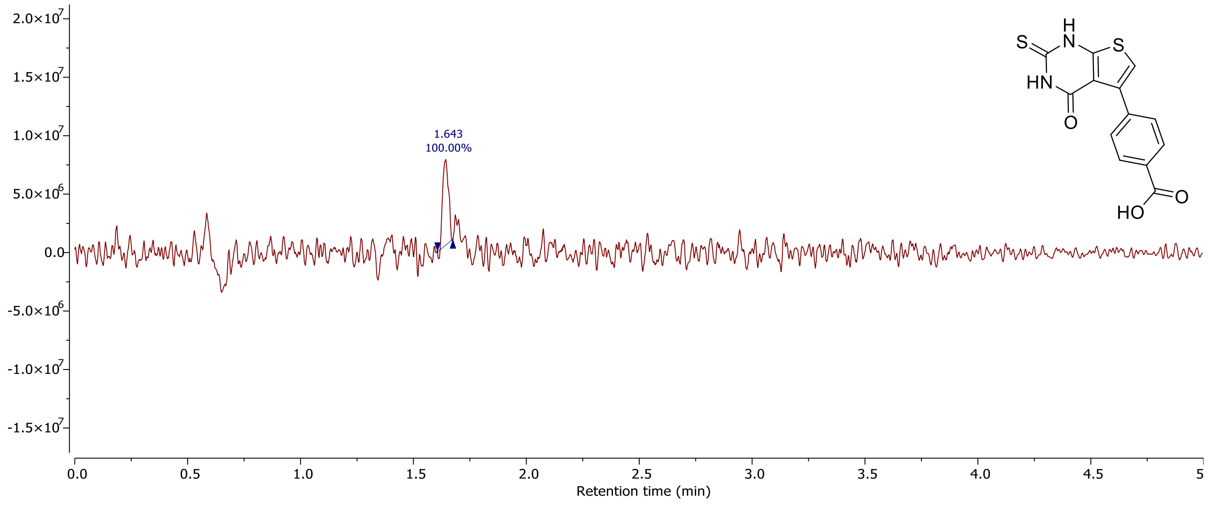

5-(4-bromophenyl)-2-thioxo-2,3-dihydrothieno[2,3-d]pyrimidin-4(1H)-one S75

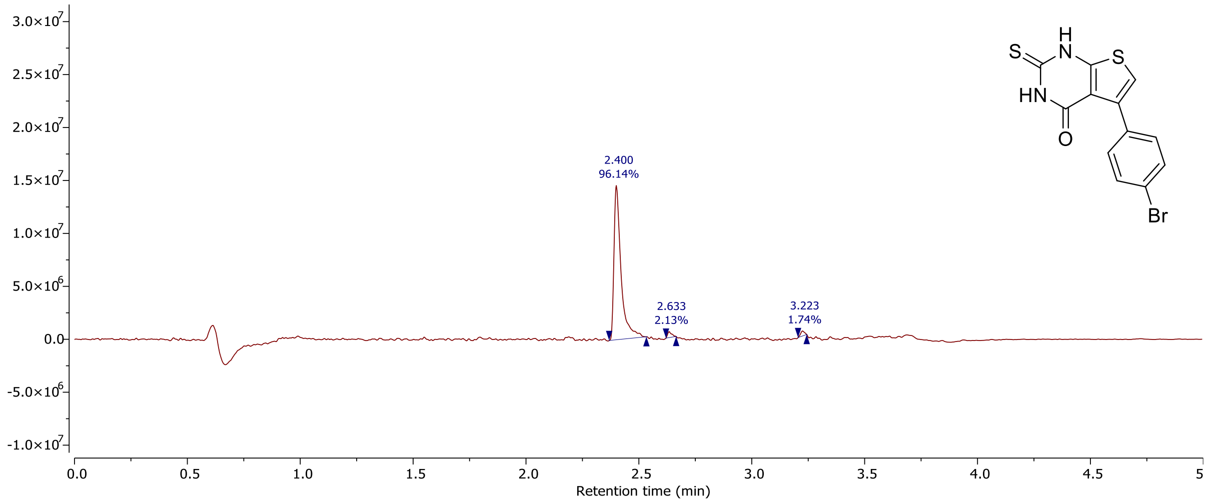

ethyl 2-(3-benzoylthioureido)-4-(4-methoxyphenyl)thiophene-3-carboxylate S76

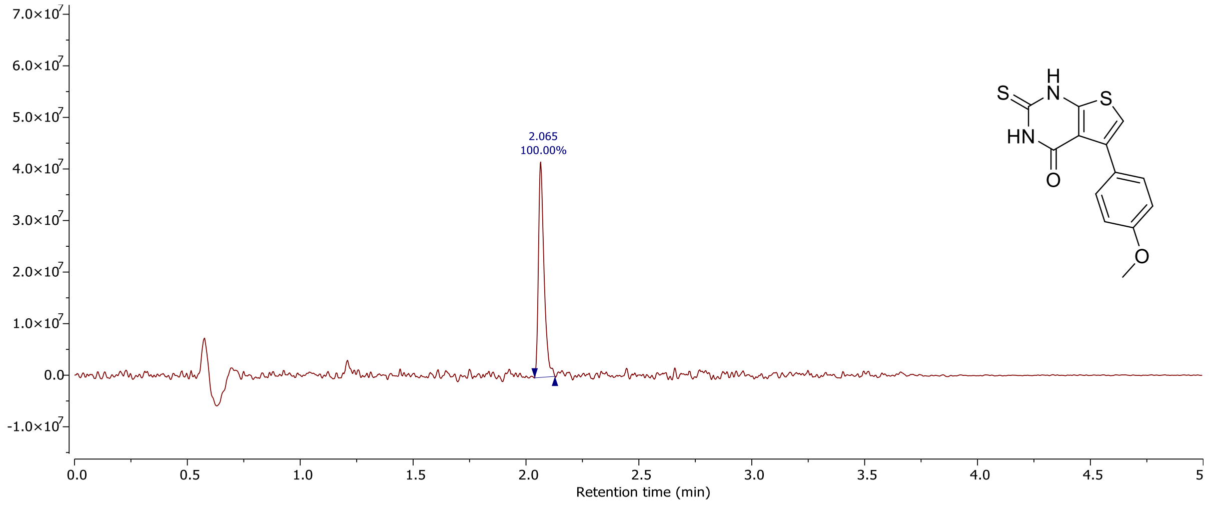

5-(4-hydroxyphenyl)-2-thioxo-2,3-dihydrothieno[2,3-d]pyrimidin-4(1H)-one 19

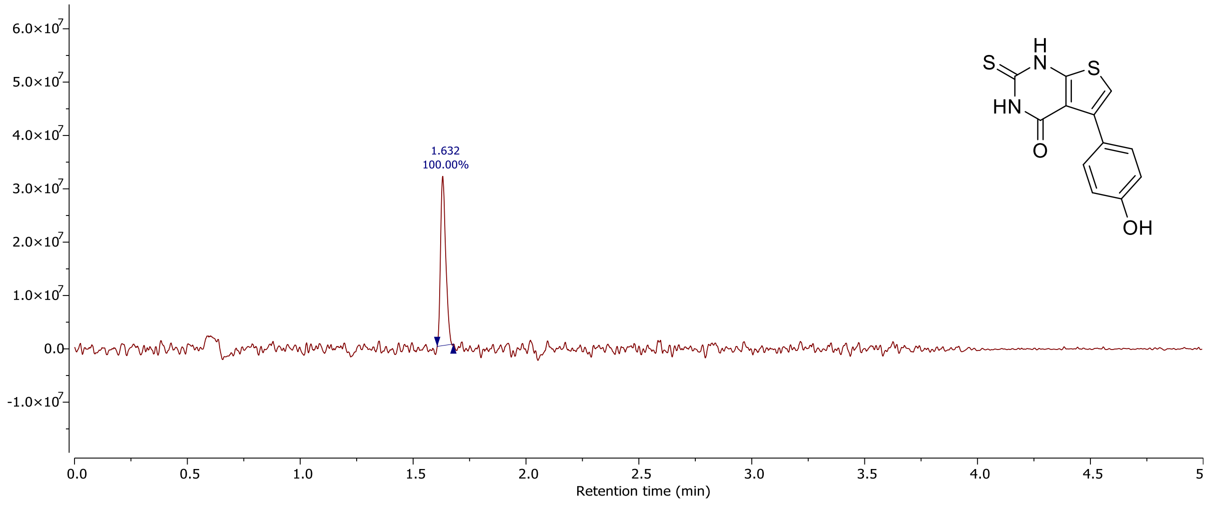

5-cyclohexyl-2-thioxo-2,3-dihydrothieno[2,3-d]pyrimidin-4(1H)-one S77

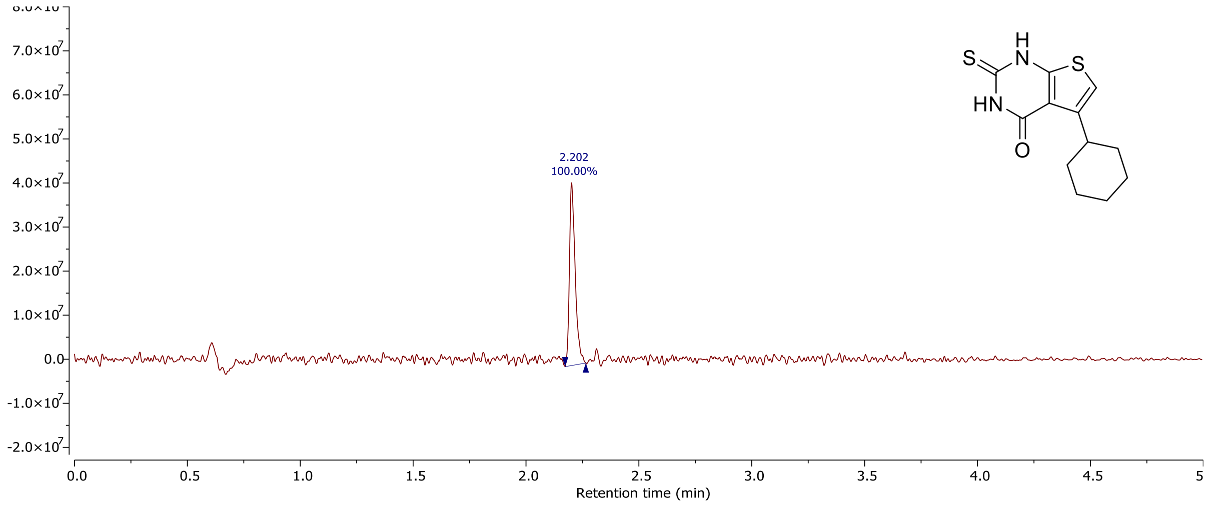

6-methyl-2-thioxo-2,3-dihydrothieno[2,3-d]pyrimidin-4(1H)-one S78

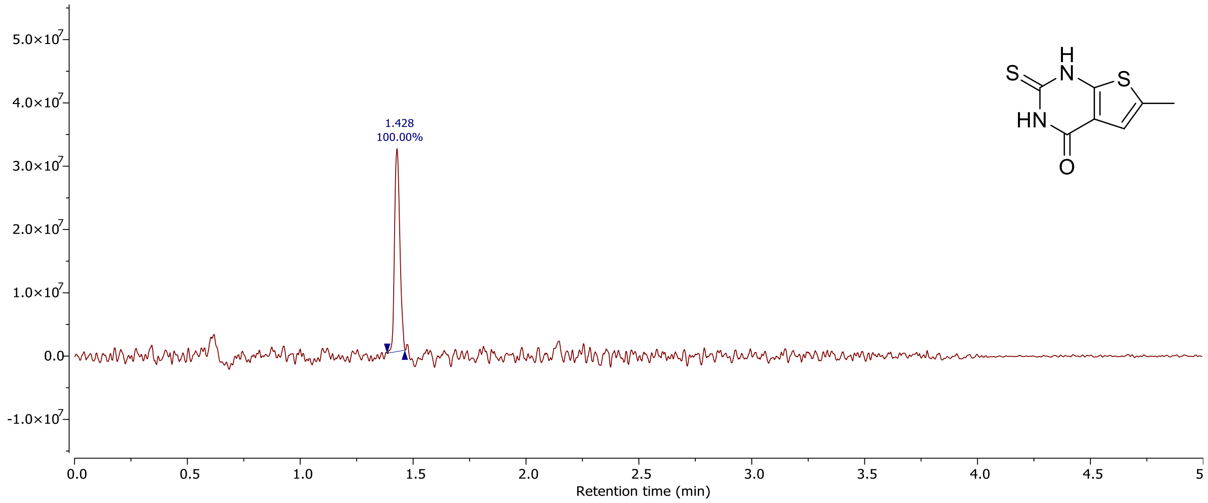

6-acetyl-5-methyl-2-thioxo-2,3-dihydrothieno[2,3-d]pyrimidin-4(1H)-one 20

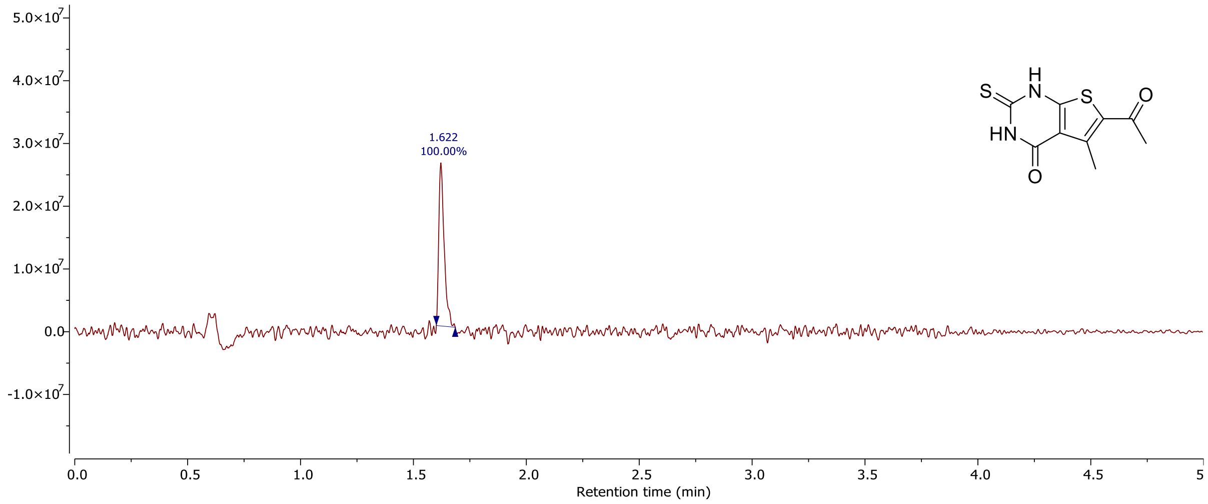

6-(1-hydroxyethyl)-5-methyl-2-thioxo-2,3-dihydrothieno[2,3-d]pyrimidin-4(1H)-one S79

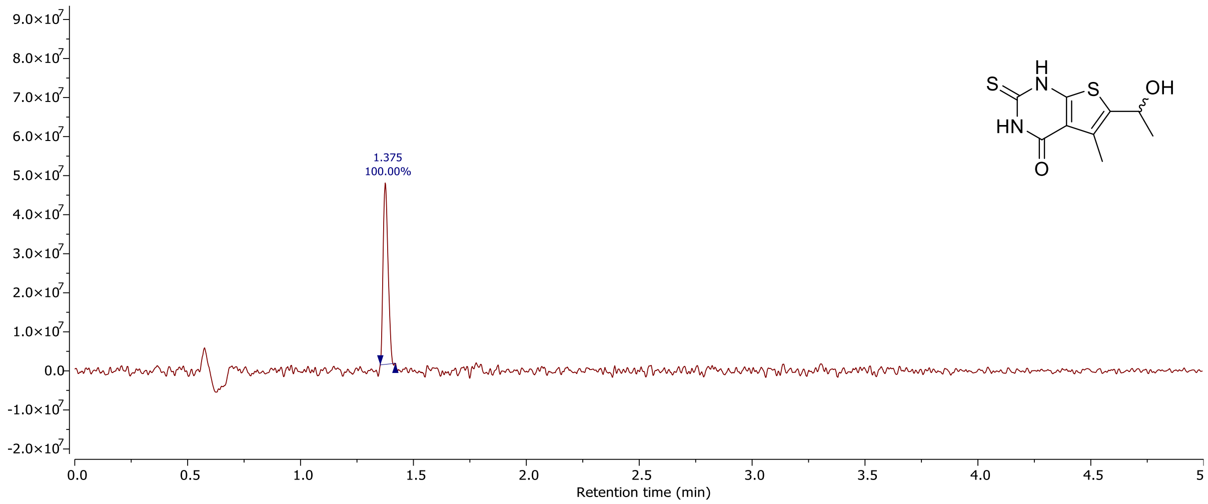

ethyl 5-methyl-4-oxo-2-thioxo-1,2,3,4-tetrahydrothieno[2,3-d]pyrimidine-6-carboxylate 21

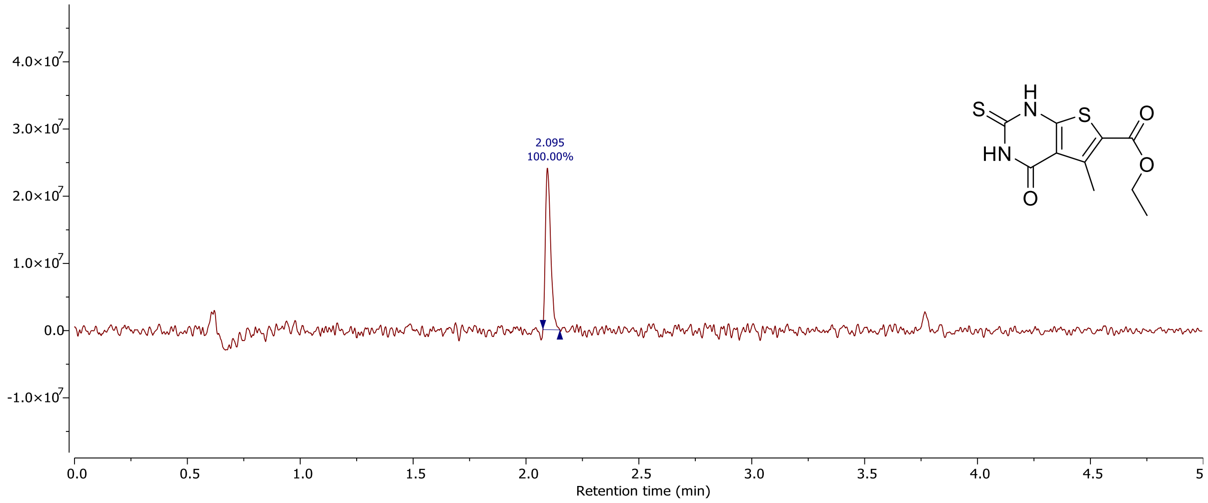

5-methyl-4-oxo-2-thioxo-1,2,3,4-tetrahydrothieno[2,3-d]pyrimidine-6-carboxylic acid S80

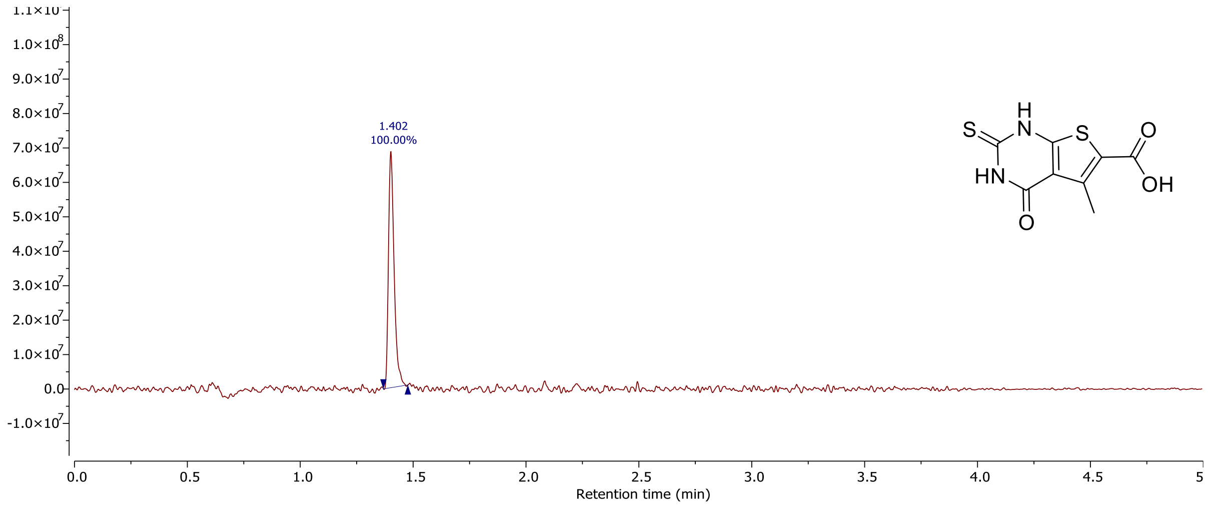

***N-benzyl-5-methyl-4-oxo-2-thioxo-1,2,3,4-tetrahydrothieno[2,3-d]pyrimidine-6-carboxamide* 22**

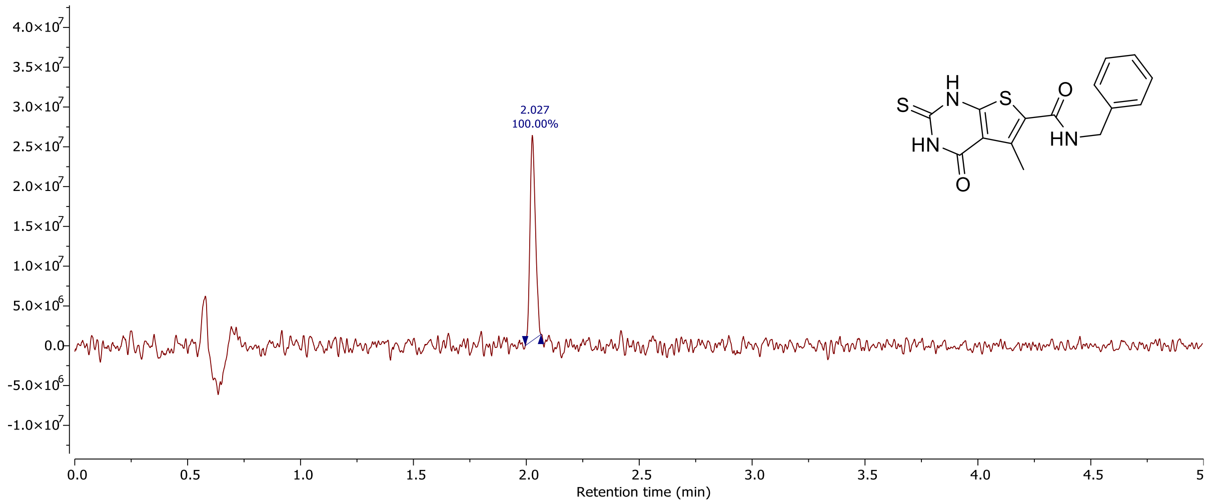

5-methyl-4-oxo-N-(pyridin-3-ylmethyl)-2-thioxo-1,2,3,4-tetrahydrothieno[2,3-d]pyrimidine-6-carboxamide 23

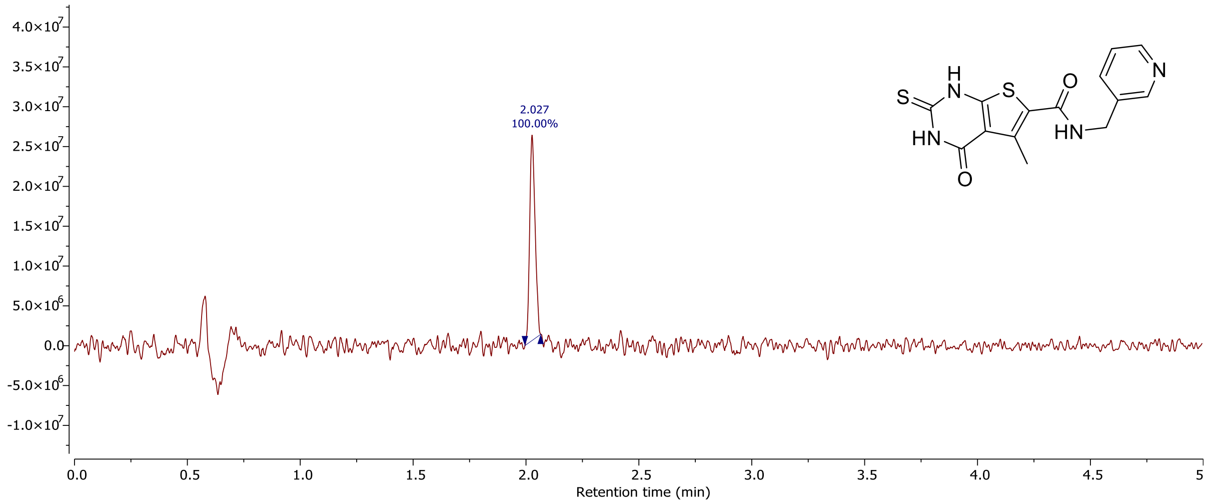

tert-butyl 3-((5-methyl-4-oxo-2-thioxo-1,2,3,4-tetrahydrothieno[2,3-d]pyrimidine-6-carboxamido)methyl)benzoate S81

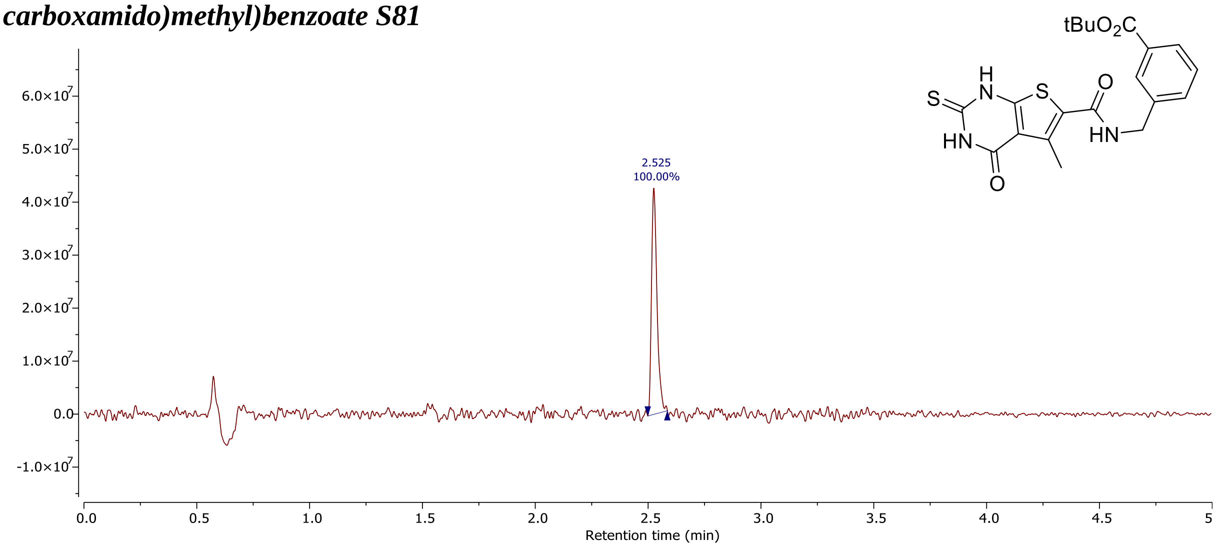

3-((5-methyl-4-oxo-2-thioxo-1,2,3,4-tetrahydrothieno[2,3-d]pyrimidine-6-carboxamido)methyl)benzoic acid 24

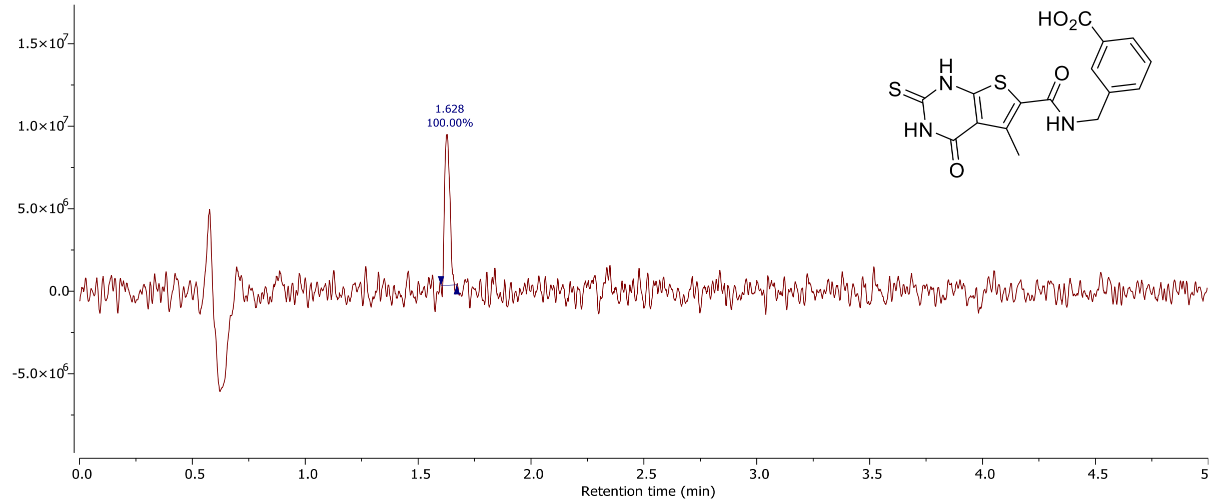

tert-butyl 4-(5-methyl-4-oxo-2-thioxo-1,2,3,4-tetrahydrothieno[2,3-d]pyrimidine-6-carboxamido)butanoate S82

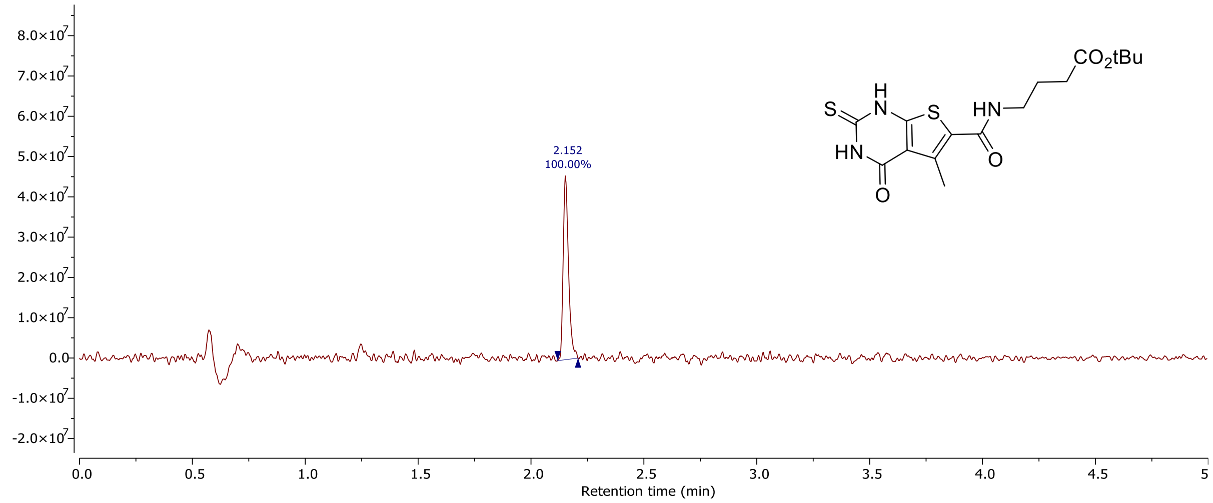

4-(5-methyl-4-oxo-2-thioxo-1,2,3,4-tetrahydrothieno[2,3-d]pyrimidine-6-carboxamido)butanoic acid 25

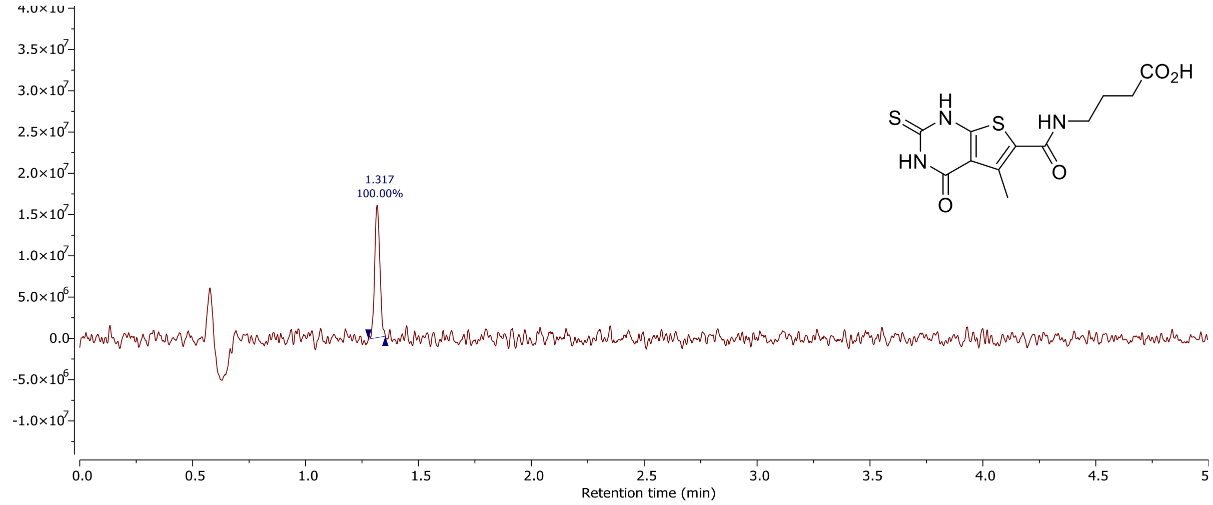

N-(4-hydroxybutyl)-5-methyl-4-oxo-2-thioxo-1,2,3,4-tetrahydrothieno[2,3-d]pyrimidine-6-carboxamide S83

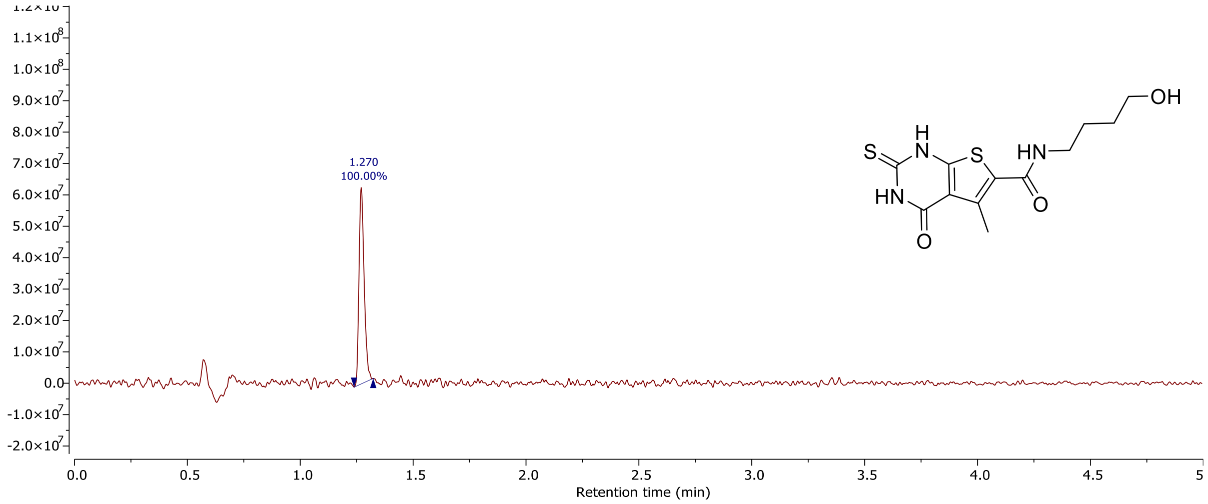

6-phenyl-2-thioxo-2,3-dihydrothieno[2,3-d]pyrimidin-4(1H)-one S84

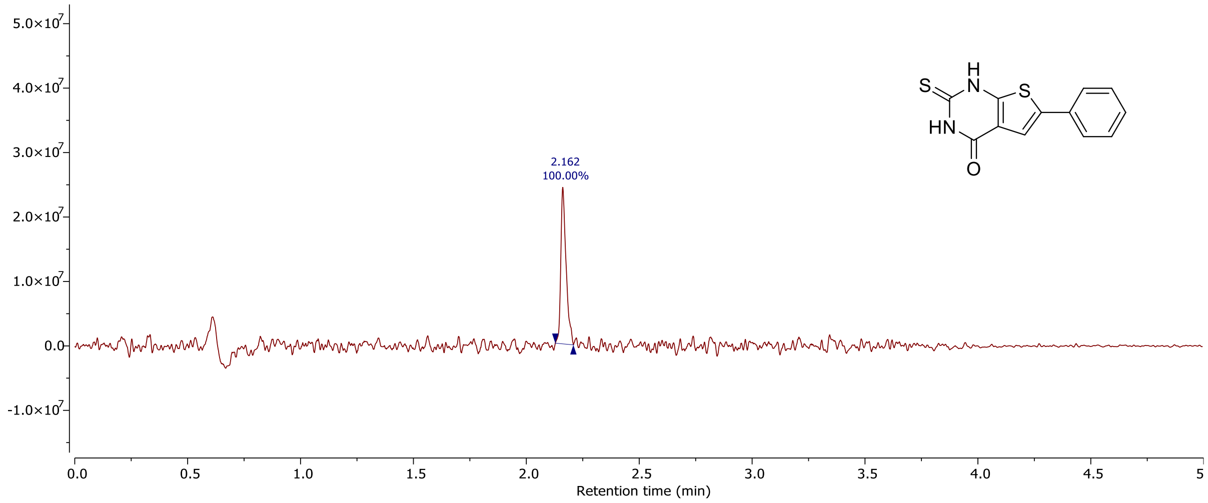

5-methyl-6-(4-nitrophenyl)-2-thioxo-2,3-dihydrothieno[2,3-d]pyrimidin-4(1H)-one 26

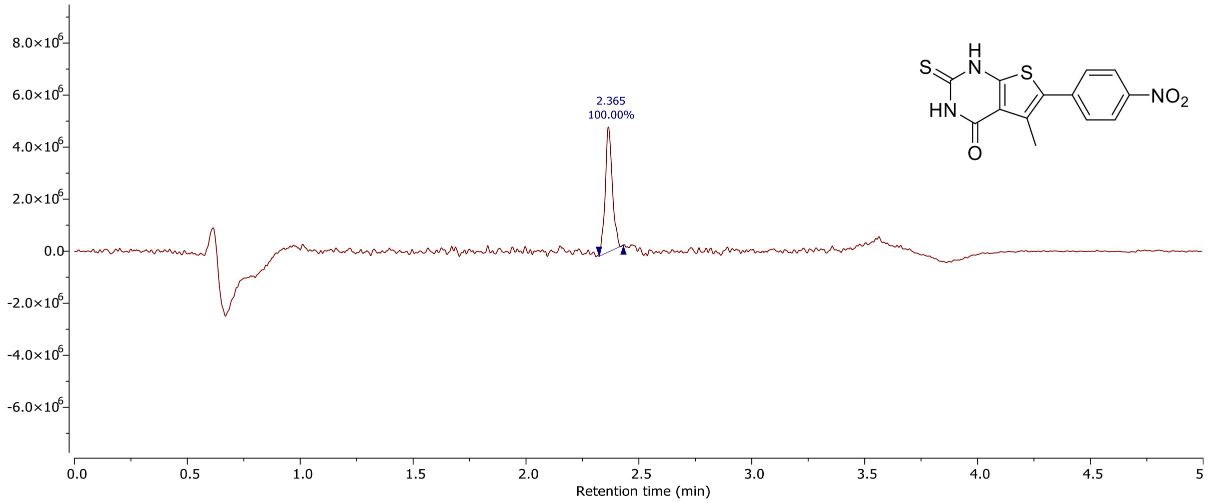

6-(4-aminophenyl)-5-methyl-2-thioxo-2,3-dihydrothieno[2,3-d]pyrimidin-4(1H)-one 27

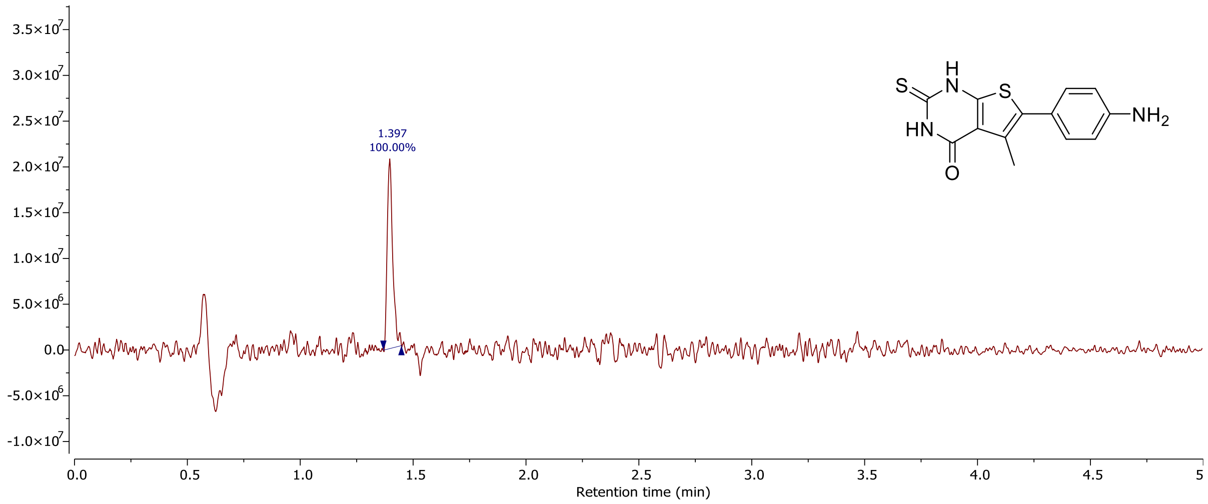

6-(4-methoxyphenyl)-2-thioxo-2,3-dihydrothieno[2,3-d]pyrimidin-4(1H)-one 28

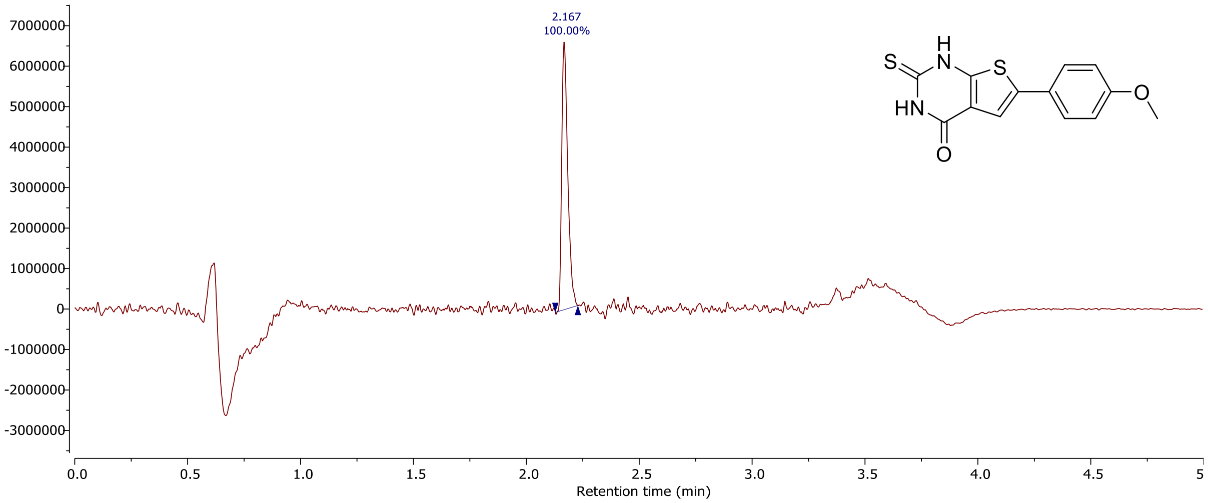

ethyl 4-(4-oxo-2-thioxo-1,2,3,4-tetrahydrothieno[2,3-d]pyrimidin-6-yl)benzoate 29

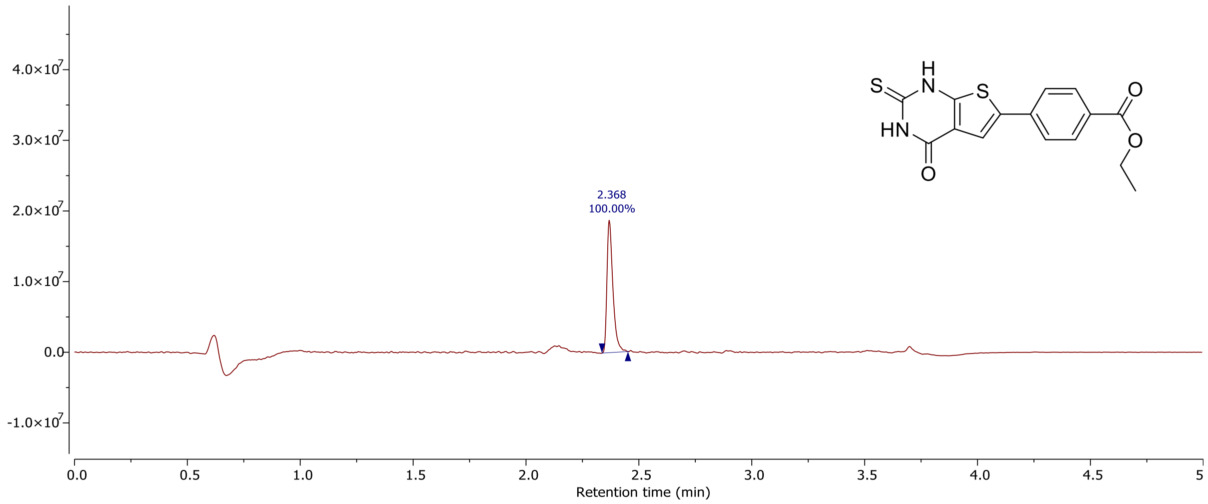

4-(4-oxo-2-thioxo-1,2,3,4-tetrahydrothieno[2,3-d]pyrimidin-6-yl)benzoic acid 30

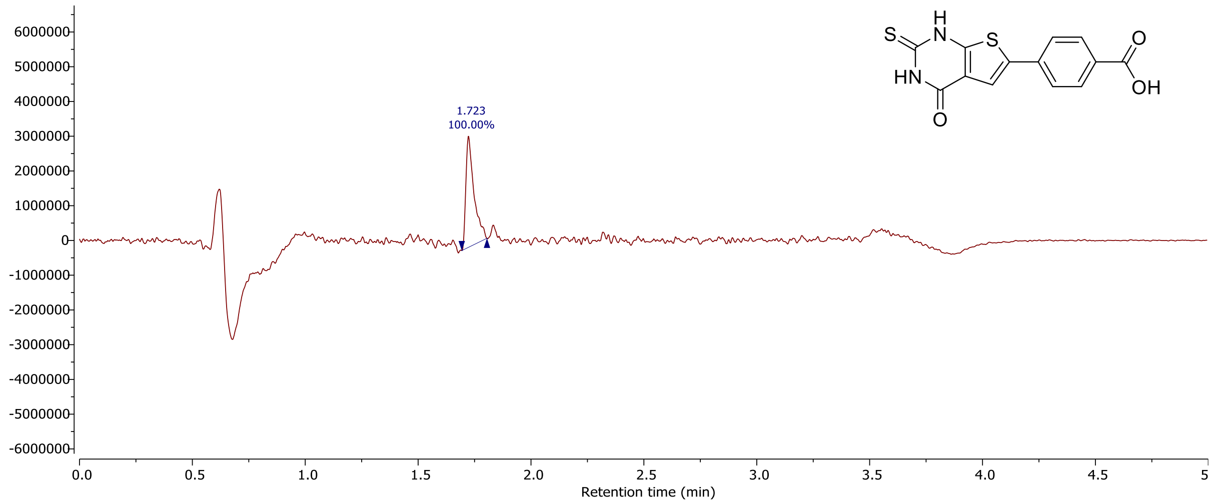

6-benzyl-2-thioxo-2,3-dihydrothieno[2,3-d]pyrimidin-4(1H)-one S85

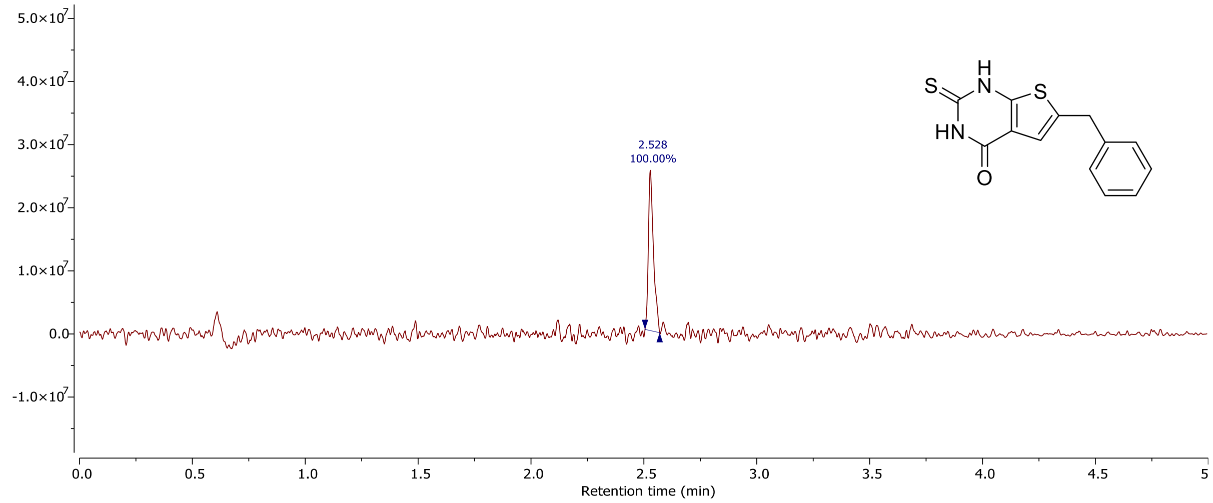

ethyl 4-((4-oxo-2-thioxo-1,2,3,4-tetrahydrothieno[2,3-d]pyrimidin-6-yl)methyl)benzoate S86

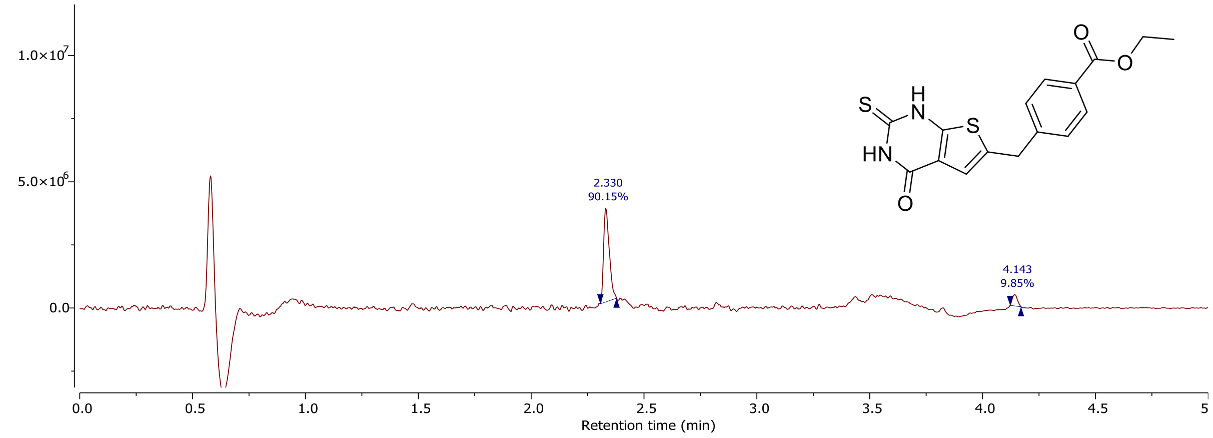

4-((4-oxo-2-thioxo-1,2,3,4-tetrahydrothieno[2,3-d]pyrimidin-6-yl)methyl)benzoic acid S87

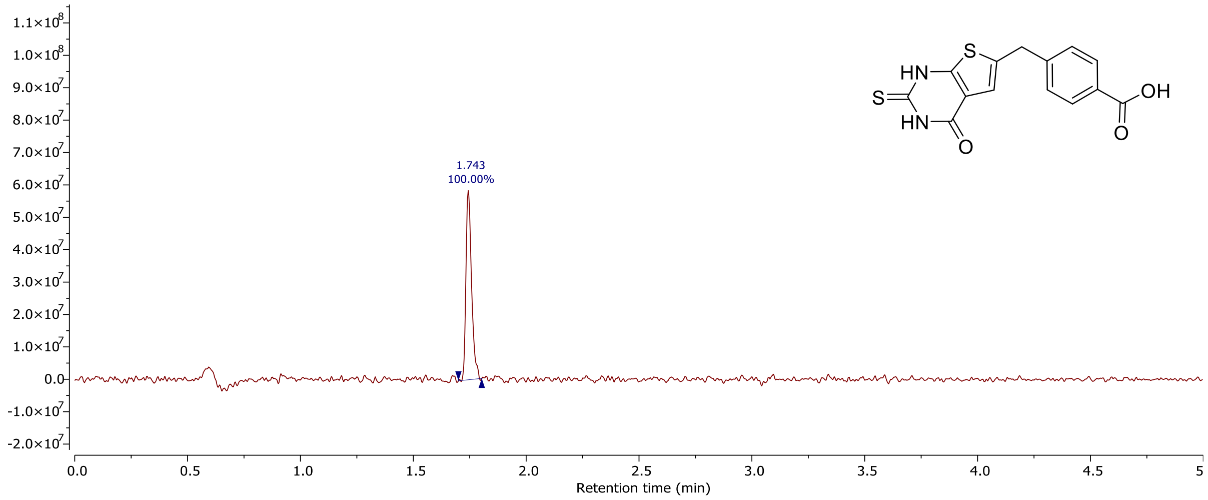

5-methyl-2-thioxo-2,3-dihydrothieno[2,3-d]pyrimidin-4(1H)-one S88

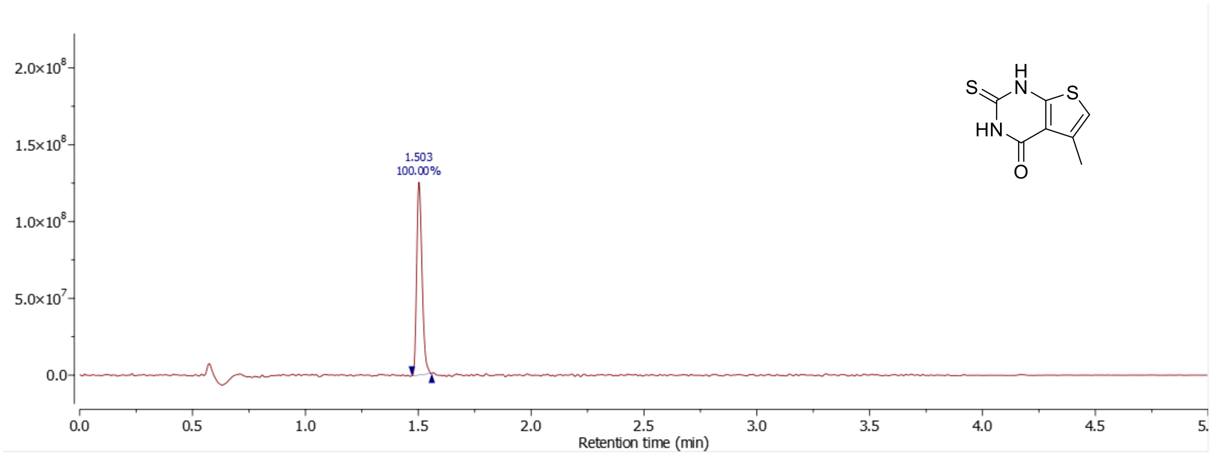

ethyl 4-oxo-2-thioxo-1,2,3,4-tetrahydrothieno[2,3-d]pyrimidine-5-carboxylate S89

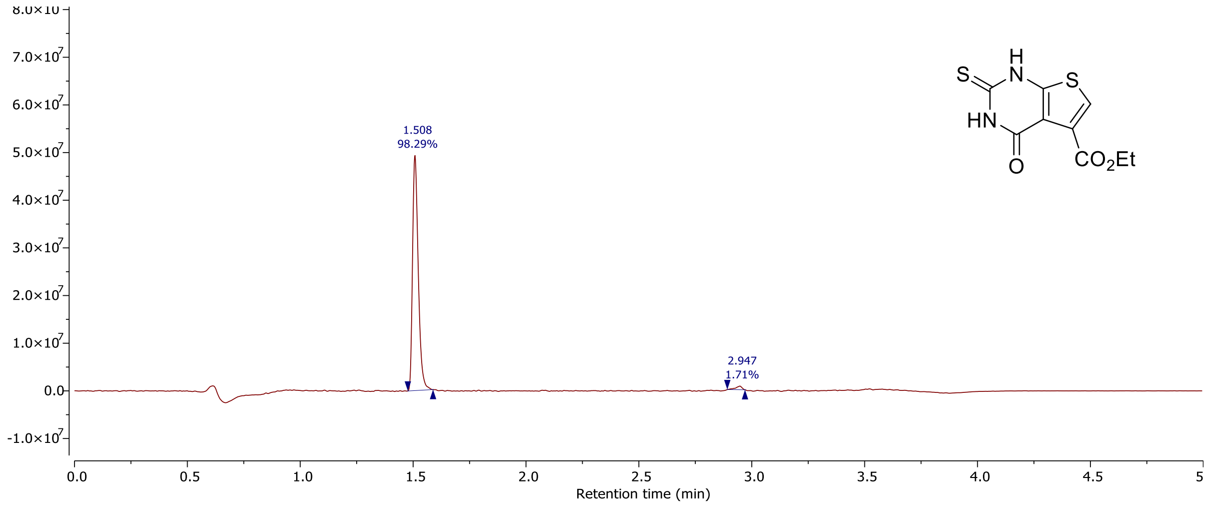

4-oxo-2-thioxo-1,2,3,4-tetrahydrothieno[2,3-d]pyrimidine-5-carboxylic acid S90

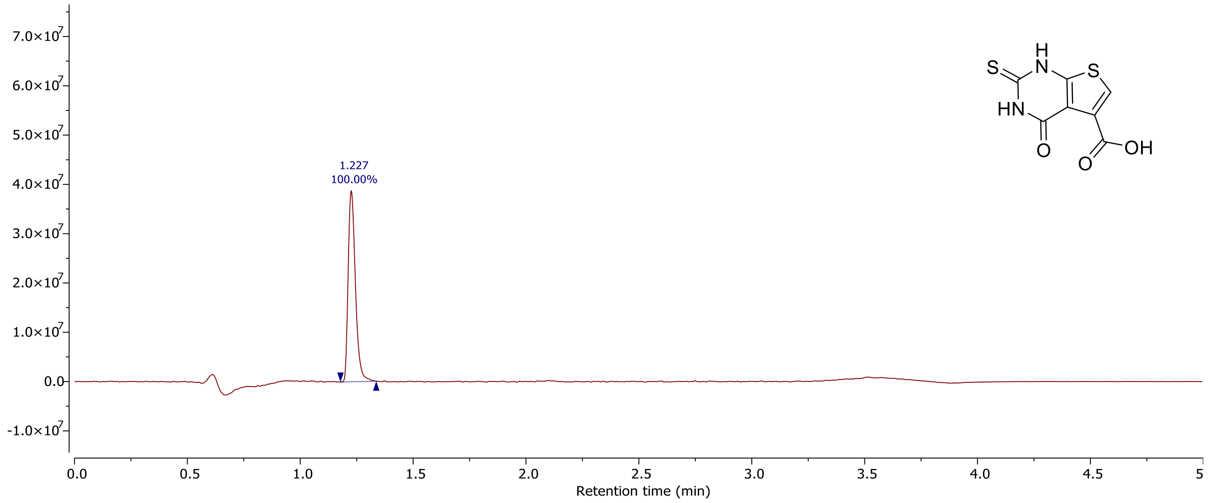

4-oxo-2-thioxo-1,2,3,4-tetrahydrothieno[2,3-d]pyrimidine-5-carboxamide 31

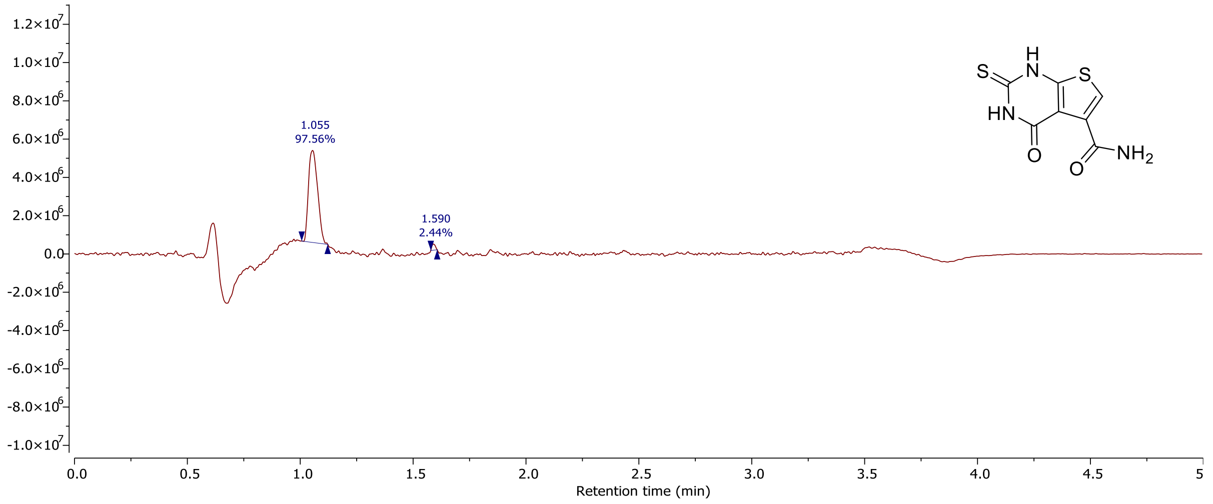

Supplement: Supplementary file 1 [file bg5c00099_si_001.pdf]
